# Global, Regional, and National Trends in Incidence and Mortality of Primary Liver Cancer and Its Underlying Etiologies from 1990 to 2019: Results from the Global Burden of Disease Study 2019

**DOI:** 10.1007/s44197-023-00109-0

**Published:** 2023-05-13

**Authors:** Guiying Cao, Jue Liu, Min Liu

**Affiliations:** Department of Epidemiology and Biostatistics, School of Public Health, Peking University, No. 38 Xueyuan Road, Haidian District, Beijing, 100191 China

**Keywords:** Primary liver cancer, Hepatitis B, Hepatitis C, Alcohol use, Nonalcoholic steatohepatitis, Global burden of disease study

## Abstract

**Objective:**

Primary liver cancer is not only one of the most common causes of cancer deaths but also the second most common cause of premature death worldwide. Understanding the trends in incidence and mortality of primary liver cancer and its etiologies is crucial for development of effective prevention and mitigation strategies. This study aimed to quantify the trends in incidence and mortality of primary liver cancer and its etiologies at the global, regional and national levels using data from Global Burden of Disease (GBD) study.

**Method:**

Annual incident cases, deaths, age-standardized incidence rates (ASIRs), and age-standardized mortality rates (ASMRs) of primary liver cancer and its etiologies, including hepatitis B, hepatitis C, alcohol use, nonalcoholic steatohepatitis, and other causes, between 1990 and 2019 were collected from GBD study 2019. Percentage changes in incident cases and deaths and estimated annual percentage changes (EAPCs) in ASIRs and ASMRs of primary liver cancer and its etiologies were calculated to quantify their temporal trends. Correlations of EAPC in ASIRs and ASMRs with socio-demographic index (SDI) and universal health coverage index (UHCI) in 2019 were separately evaluated by Pearson correlation analyses.

**Results:**

Globally, the incident cases and deaths of primary liver cancer increased by 43.11% from 373 393 in 1990 to 534 365 in 2019 and 32.68% from 365 213 in 1990 to 484 584 in 2019, respectively. ASIR and ASMR of primary liver cancer decreased by an average of 2.23% (95% CI 1.83%, 2.63%) and 1.93% (95% CI 1.55%, 2.31%) per year between 1990 and 2019 worldwide, respectively. ASIRs and ASMRs of primary liver cancer varied between regions, with an increasing trend in ASIR (EAPC = 0.91; 95% CI 0.47, 1.35) and a stable trend in ASMR (EAPC = 0.42, 95% CI − 0.01, 0.85) of primary liver cancer in high SDI region between 1990 and 2019. Nearly half (91/204) of the countries suffered an increasing trend in ASIR of primary liver cancer and more than one-third (71/204) of the countries suffered an increasing trend in ASIRs of primary liver cancer from all etiologies between 1990 and 2019 worldwide. Positive correlations of EAPC in ASIR and ASMR of primary liver cancer with SDI and UHCI were observed in nations with SDI ≥ 0.7 or UHCI ≥ 70.

**Conclusion:**

Primary liver cancer remains a major public health concern globally, with an increasing trend in the numbers of incident cases and deaths in the past three decades. We observed an increasing trend in ASIR of primary liver cancer in nearly half of the countries and an increasing trend in ASIRs of primary liver cancer by etiology in more than one-third of the countries worldwide. In line with the Sustainable Development Goals, the identification and elimination of risk factors for primary liver cancer will be required to achieve a sustained reduction in liver cancer burden.

**Supplementary Information:**

The online version contains supplementary material available at 10.1007/s44197-023-00109-0.

## Introduction

Primary liver cancer was the third leading cause of cancer deaths worldwide in 2020 after lung and colorectum cancer [1]. In addition, primary liver cancer also ranked as the second most common cause of premature death from cancer in 2020 [2]. The exogenous risk factors for primary liver cancer include chronic infection with hepatitis B virus (HBV) or hepatitis C virus (HCV), excessive alcohol consumption, cigarette smoking, liver flukes, aflatoxin-contaminated foods, and the related conditions of diabetes, obesity, and non-alcoholic fatty liver disease [3–5]. It was estimated that approximately 56% of incident cases of primary liver cancer were related to chronic HBV infection and 20% were related to chronic HCV infection in 2012 worldwide [6]. Although chronic HBV and HCV infections constitute the most important exogenous risk factors for primary liver cancer, other risk factors for primary liver cancer have also become prominent, and multiple risk factors may be attributed to the same cases or deaths [4, 5, 7]. The major risks of primary liver cancer vary by geographic region, resulting in marked differences in the burden of primary liver cancer across regions [8, 9].

Prevention and control for noncommunicable diseases (including cancers) and combating viral hepatitis as part of the UN Sustainable Development Goal (SDG) and World Health Organization (WHO) targets, its strategies and practices include eliminating viral hepatitis as a public health threat by 2030, reducing tobacco use and harmful use of alcohol, and controlling diabetes and obesity [10–12]. Thus, primary liver cancer can be prevented by eliminating or reducing its modifiable risk factors under the targets of SDG and WHO for noncommunicable diseases and viral hepatitis. However, even best-case scenarios of these prevention approaches could be unlikely to reduce the number of patients with primary liver cancer in the foreseeable future due to the lag between risk factor exposure and the development of liver cancer [9]. In addition, there were marked disparities in the burden of major risk factors for primary liver cancer and progress to prevent and control noncommunicable diseases and viral hepatitis of the SDG and WHO targets across the world [8, 13, 14]. For example, the prevalence of chronic HBV infections varied widely across regions, with the highest prevalence in Western Pacific region (7.1%) and the lowest prevalence in Europe (1.1%) in 2019, and many countries had not met the WHO Global Health Sector Strategy on Viral Hepatitis 2020 interim targets for new cases of HBV infection by 2019 [15]. Finally, the major risk factors for primary liver cancer appear to be in transition, with the prevalence of HBV and HCV declining and excess body weight and diabetes increasing in many regions [16]. Thus, understanding the pattern of incidence and mortality of primary liver cancer as well as the temporal trends by location is crucial for the development of effective prevention and mitigation strategies, and can serve as one of the considerations toward progress on SDG and WHO relating targets to non-communicable diseases.

To our knowledge, no prior study has provided estimates for the temporal trends in the incidence and mortality of primary liver cancer and its specific etiologies at the global, regional, and national levels. Prior studies analyzing the burden of primary liver cancer have either focused on a subset of the most common etiologies, such as HBV and HCV infection or alcohol use, single countries or regions, or single years without temporal trends [7, 9, 17–19]. In this study, we retrieved detailed data on the global, regional, and national incidence and mortality of primary liver cancer and its underlying etiologies from the Global Burden of Disease (GBD) Study 2019 to determine their trends in the past three decades and their associations with socioeconomic status at the national level to provide a more comprehensive perspective to develop effective prevention and mitigation strategies worldwide.

## Methods

### Data Source

We used data on annual incident cases, deaths, age-standardized incidence rates (ASIRs), and age-standardized mortality rates (ASMRs) of primary liver cancer and its underlying etiologies from 1990 to 2019 by etiology and location, which were estimated by GBD using the Bayesian meta-regression tool DisMod-MR 2.1 [20]. Data were available at the Global Health Data Exchange (GHDx) query tool from a total of 204 countries and territories, and these were categorized into 5 regions in terms of socio-demographic index (SDI) and 21 GBD regions according to geographical contiguity [21]. In the GBD study, five kinds of underlying etiologies for primary liver cancer were hepatitis B, hepatitis C, alcohol use, nonalcoholic steatohepatitis (NASH), and other causes, which included remaining etiologies such as liver flukes, obesity, and aflatoxins. The incident cases and deaths of primary liver cancer were defined by ICD-10 C22 in the GBD study [17]. Specific methods of GBD study estimation for the incidence and mortality of primary liver cancer have been described elsewhere [9, 17]. Data on SDI values and universal health coverage index (UHCI) in 204 countries and territories in 2019 used in this study were also collected from the GHDx query tool [16]. The SDI and UHCI values of 204 countries and terroirs in 2019 are shown in Table S1.

### SDI

The SDI is a composite indicator of development status strongly correlated with health outcomes [22]. It is the geometric mean of 0–1 indices of lag distributed income per capita, average years of schooling for those ages 15 and older, and total fertility rate under the age of 25. A location with an SDI of 0 indicates a theoretical minimum level of development status relevant to health outcomes, while a location with an SDI of 1 indicates a theoretical maximum level [22].

### UHCI

The UHCI developed following GBD 2019 is comprised of 23 indicators drawn across a range of health service areas and is meant to represent healthcare needs over the life course [23]. The indicators of UHCI involved either direct measures of intervention coverage (e.g., antiretroviral therapy coverage) or outcome-based indicators, such as mortality-to-incidence ratios, to approximate access to quality care [24]. The UHCI indicators are reported on a scale of 0–100 [24].

### Statistical Analysis

We presented the incident cases, deaths, ASIR, and ASMR of primary liver cancer with 95% uncertainty intervals (UIs) to quantify the disease burden. We calculated the proportion of annual incident cases and deaths of primary liver cancer by etiology, location, and year. The percentage changes in incident cases and deaths and the estimated annual percentage changes (EAPCs) in the ASIR and ASMR with 95% confidence intervals (CIs) were calculated to reflect the temporal trends in the incidence and mortality of primary liver cancer. The percentage changes in incident cases and deaths of primary liver cancer from 1990 to 2019 were calculated by the equation: Percentage changes = $\frac{\mathrm{Incedent\, cases}/\mathrm{Deaths\, in\, }2019 -\mathrm{ Incident\, cases}/\mathrm{Deaths\, in\, }1990}{\mathrm{Incident\, cases}/\mathrm{Deaths\, in\, }1990}\times$ 100%. The EAPC is a summary and widely used measure of the age-standardized rate (ASR) tend over a specified time interval. A regression line was fitted to the natural logarithm of the rate, i.e., y = α + βx + ε, where y = ln (ASR) and x = calendar year. The EAPC was calculated as $100\times {(e}^{\beta }-1)$ and its 95% CI was calculated to reflect the temporal trend in ASR. The EAPC in ASIR and ASMR of primary liver cancer were calculated by this method. The trend in ASIR and ASMR is reflected in EAPC value and its 95% CI: rate is in an upward trend when the EAPC and the lower boundary of the 95% CI are positive; conversely, rate is in a downward trend when EAPC and the upper boundary of the 95% CI are negative. Moreover, the correlations of EAPC in ASIR and ASMR of primary liver cancer with SDI values (2019) and UHCI values (2019) in 204 countries and territories were evaluated by Pearson correlation analyses to define the potential factors affecting EAPC. The polynomial curves were also modelled. All analyses were conducted with SAS 9.4 (SAS Institute, Inc., Cary, NC) and Origin 2019b. A two-tailed *p* value less than 0.05 was considered statistically significant.

### Patient and Public Involvement

Patients and the public were not involved in the design, conduct or dissemination of this study.

## Results

### Global Trends in the Incidence and Mortality of Primary Liver Cancer and Its Underlying Etiologies

**Fig. 1:**
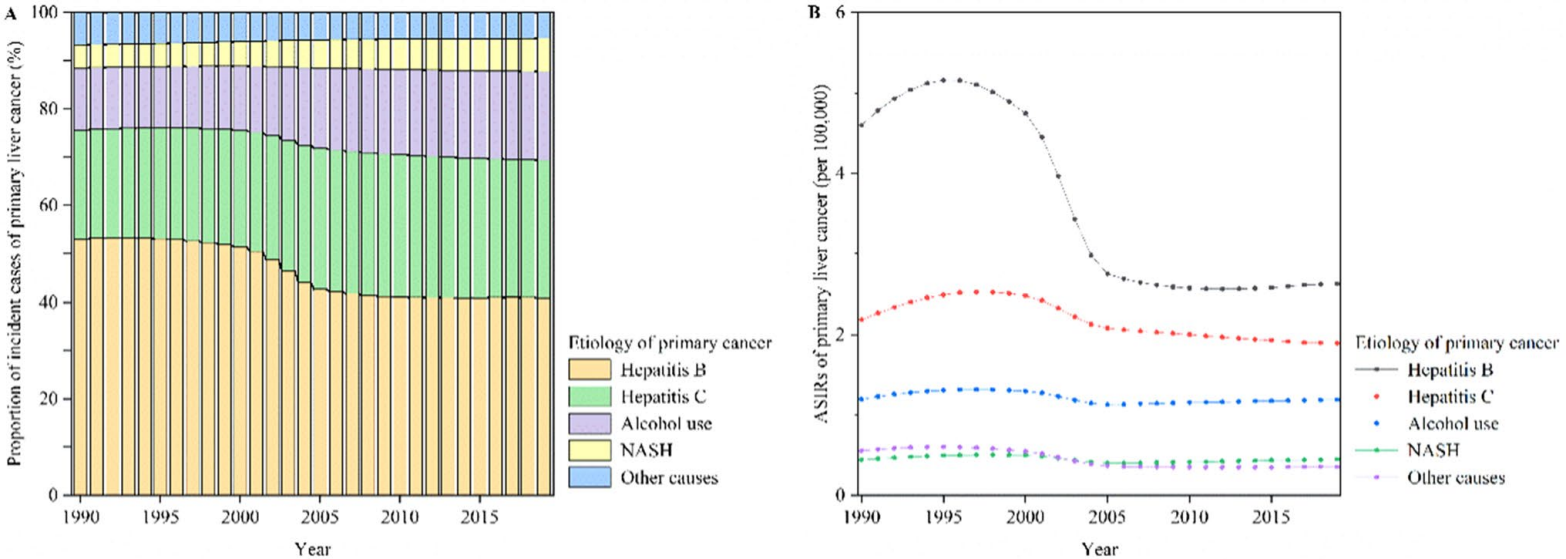
Global proportions of primary liver cancer incident cases by etiology and ASIRs of primary liver cancer by etiology between 1990 and 2019. **A** Global proportions of primary liver cancer incident cases by etiology between 1990 and 2019; **B** Global ASIRs of primary liver cancer by etiology between 1990 and 2019. Note: *ASIR* age-standardized incidence rate; *NASH* nonalcoholic steatohepatitis

Globally, the incident cases of primary liver cancer increased by 43.11% from 373,393 in 1990 to 534,365 in 2019 and the ASIR of primary liver cancer decreased by an average of 1.93% (95% CI 1.55%, 2.31%) per year between 1990 and 2019 (from 8.98 per 100,000 in 1990 to 6.51 per 100,000 in 2019) (Table 1). The global incident cases of primary liver cancer by etiology from 1990 to 2019 increased by 10.47% for hepatitis B, 80.68% for hepatitis C, 103.69% for alcohol use, 105.34% for NASH, and 13.94% for other causes (Table 1). Among the global incident cases of primary liver cancer in each year from 1990 to 2019, more than two-thirds were due to hepatitis B and hepatitis C, and less than one-third were due to alcohol use, NASH, and other causes (Fig. 1A). Among the global incident cases of primary liver cancer between 1990 and 2019, the proportions of incident cases decreased from 53.06% 1990 to 40.95% in 2019 for hepatitis B and 6.59% in 1990 to 5.33% in 2019 for other causes, whereas the proportions of incident cases increased from 22.56% 1990 to 28.49% in 2019 for hepatitis C, 12.95% in 1990 to 18.43% in 2019 for alcohol use, and 4.47% in 1990 and 6.80% in 2019 for NASH (Fig. 1A). The highest ASIR of primary liver cancer by etiology between 1990 and 2019 worldwide was hepatitis B, followed by hepatitis C and alcohol use (Table 1 and Fig. 1B). The ASIRs of primary liver cancer due to hepatitis B, hepatitis C, alcohol use, NASH, and other causes were 2.62 per 100,000, 1.90 per 100,000, 1.19 per 100,000, 0.45 per 100,000, and 0.35 per 100,000 in 2019, respectively (Table 1). The ASIRs of primary liver cancer due to hepatitis B, hepatitis C, alcohol use, NASH, and other causes decreased by an average of 3.10% (95% CI 2.53%, 3.66%), 1.04% (95% CI 0.81%, 1.27%), 0.40% (95% CI 0.22%, 0.57%), 0.51% (95% CI 0.23%, 0.78%), and 2.42% (95% CI 1.99%, 2.85%) per year between 1990 and 2019, respectively (Table 1 and Fig. 1B).

**Table 1:** The incident cases and ASIR of primary liver cancer in 1990 and 2019 and their change trends from 1990 to 2019

Characteristics	1990		2019		1990–2019	
	Incident cases No. × 10^3^ (95% UI)	ASIR per 100,000 No. (95% UI)	Incident cases No. × 10^3^ (95% UI)	ASIR per 100,000 No. (95% UI)	Percentage change in incident cases (%)	EAPC in ASIR No. (95% CI)
Overall	373.39 (335.89, 415.75)	8.98 (8.10, 9.97)	534.36 (486.55, 588.64)	6.51 (5.95, 7.16)	43.11	− 1.93 (− 2.31, − 1.55)
Etiology						
Hepatitis B	198.11 (165.91, 232.20)	4.60 (3.86, 5.39)	218.86 (186.49, 254.89)	2.62 (2.24, 3.05)	10.47	− 3.10 (− 3.66, − 2.53)
Hepatitis C	84.25 (72.74, 96.02)	2.19 (1.90, 2.49)	152.23 (131.58, 174.63)	1.90 (1.64, 2.17)	80.68	− 1.04 (− 1.27, − 0.81)
Alcohol use	48.34 (38.84, 59.09)	1.20 (0.96, 1.47)	98.46 (79.03, 120.13)	1.19 (0.96, 1.45)	103.69	− 0.40 (− 0.57, − 0.22)
NASH	17.70 (14.53, 21.17)	0.44 (0.36, 0.53)	36.34 (29.49, 44.86)	0.45 (0.37, 0.55)	105.34	− 0.51 (− 0.78, − 0.23)
Other causes	25.00 (20.96, 29.80)	0.56 (0.47, 0.67)	28.48 (23.57, 34.08)	0.35 (0.29, 0.42)	13.94	− 2.42 (− 2.85, − 1.99)
SDI region						
Low	10.25 (9.00, 11.59)	4.08 (3.58, 4.63)	20.29 (17.77, 22.83)	3.69 (3.27, 4.11)	97.99	− 0.46 (− 0.52, − 0.40)
Low-middle	34.72 (31.28, 38.61)	5.36 (4.85, 5.93)	56.30 (51.00, 62.61)	4.05 (3.67, 4.51)	62.17	− 1.59 (− 1.90, − 1.27)
Middle	166.09 (144.08, 193.62)	14.73 (12.80, 17.04)	210.55 (184.18, 242.24)	8.28 (7.24, 9.47)	26.77	− 2.98 (− 3.56, − 2.39)
Middle-high	108.73 (95.24, 123.11)	9.92 (8.73, 11.20)	106.88 (94.23, 121.00)	5.34 (4.70, 6.05)	− 1.70	− 3.33 (− 3.89, − 2.77)
High	53.49 (51.83, 54.71)	5.27 (5.11, 5.39)	140.14 (125.50, 154.01)	7.61 (6.88, 8.36)	161.98	0.91 (0.47, 1.35)
GBD region						
High-income Asia Pacific	28.21 (27.27, 29.08)	13.77 (13.30, 14.19)	67.95 (58.13, 77.64)	15.56 (13.46, 17.74)	140.84	− 0.17 (− 0.73, 0.39)
Central Asia	1.49 (1.33, 1.66)	3.13 (2.79, 3.47)	6.11 (5.30, 7.00)	8.27 (7.22, 9.41)	309.21	2.78 (2.23, 3.32)
East Asia	241.53 (204.19, 284.76)	25.26 (21.46, 29.74)	217.17 (181.40, 257.46)	10.43 (8.76, 12.30)	− 10.09	− 4.60 (− 5.39, − 3.80)
South Asia	15.68 (13.21, 18.05)	2.66 (2.20, 3.08)	37.73 (32.78, 43.28)	2.66 (2.30, 3.05)	140.66	− 0.02 (− 0.10, 0.06)
Southeast Asia	17.31 (15.37, 18.98)	6.43 (5.69, 7.06)	42.80 (35.22, 52.13)	7.07 (5.87, 8.61)	147.29	0.31 (0.25, 0.37)
Australasia	0.48 (0.46, 0.49)	2.05 (1.97, 2.13)	2.16 (1.75, 2.67)	4.59 (3.72, 5.68)	354.16	3.14 (2.87, 3.41)
Caribbean	1.56 (1.45, 1.66)	5.94 (5.52, 6.30)	1.63 (1.35, 1.94)	3.16 (2.63, 3.77)	4.17	− 2.09 (− 2.89, − 1.28)
Central Europe	7.70 (7.44, 7.90)	5.25 (5.06, 5.39)	6.91 (5.99, 7.99)	3.29 (2.85, 3.81)	− 10.30	− 1.32 (− 1.68, − 0.95)
Eastern Europe	4.14 (3.97, 4.33)	1.52 (1.45, 1.59)	9.41 (8.20, 10.74)	2.84 (2.46, 3.24)	127.26	2.52 (2.27, 2.78)
Western Europe	20.18 (19.49, 20.70)	3.55 (3.43, 3.64)	45.86 (39.84, 52.74)	5.31 (4.59, 6.12)	127.21	1.37 (1.18, 1.55)
Andean Latin America	1.04 (0.91, 1.17)	4.90 (4.31, 5.52)	1.74 (1.42, 2.11)	3.11 (2.55, 3.78)	67.47	− 2.01 (− 2.45, − 1.57)
Central Latin America	2.97 (2.76, 3.12)	3.49 (3.24, 3.68)	7.99 (6.88, 9.27)	3.43 (2.96, 3.97)	168.89	0.11 (− 0.22, 0.44)
Southern Latin America	0.72 (0.65, 0.79)	1.56 (1.41, 1.71)	1.94 (1.52, 2.42)	2.33 (1.83, 2.92)	168.80	2.05 (1.81, 2.30)
Tropical Latin America	1.85 (1.77, 1.90)	1.97 (1.88, 2.04)	5.67 (5.34, 5.96)	2.36 (2.22, 2.48)	206.92	1.05 (0.90, 1.20)
North Africa and Middle East	10.73 (9.45, 12.00)	6.08 (5.28, 6.81)	27.55 (22.11, 33.84)	6.29 (5.13, 7.71)	156.62	0.49 (0.32, 0.67)
High-income North America	7.53 (7.28, 7.70)	2.20 (2.14, 2.25)	31.01 (25.71, 36.96)	5.18 (4.28, 6.18)	311.64	2.99 (2.79, 3.20)
Oceania	0.11 (0.10, 0.13)	3.65 (3.08, 4.25)	0.23 (0.20, 0.28)	3.28 (2.77, 3.89)	106.30	− 0.22 (− 0.28, − 0.16)
Central Sub-Saharan Africa	0.69 (0.56, 0.83)	2.63 (2.20, 3.08)	1.36 (1.08, 1.71)	2.30 (1.84, 2.86)	98.73	− 0.61 (− 0.68, − 0.53)
Eastern Sub-Saharan Africa	2.44 (1.99, 3.03)	2.93 (2.44, 3.70)	5.44 (4.46, 6.71)	3.13 (2.60, 3.81)	122.59	− 0.03 (− 0.15, 0.10)
Southern Sub-Saharan Africa	1.91 (1.34, 3.15)	6.47 (4.52, 10.72)	4.02 (3.58, 4.52)	6.77 (6.07, 7.61)	110.47	− 0.43 (− 1.00, 0.14)
Western Sub-Saharan Africa	5.11 (4.33, 5.96)	5.45 (4.60, 6.32)	9.71 (8.16, 11.42)	4.93 (4.19, 5.72)	89.84	− 0.47 (− 0.54, − 0.39)

*ASIR* age-standardized incidence rate; *CI* confidence interval; *GBD* Global Burden of Disease; *NASH* nonalcoholic steatohepatitis; *SDI* socio-demographic index; *UI* uncertainty interval

**Fig. 2:**
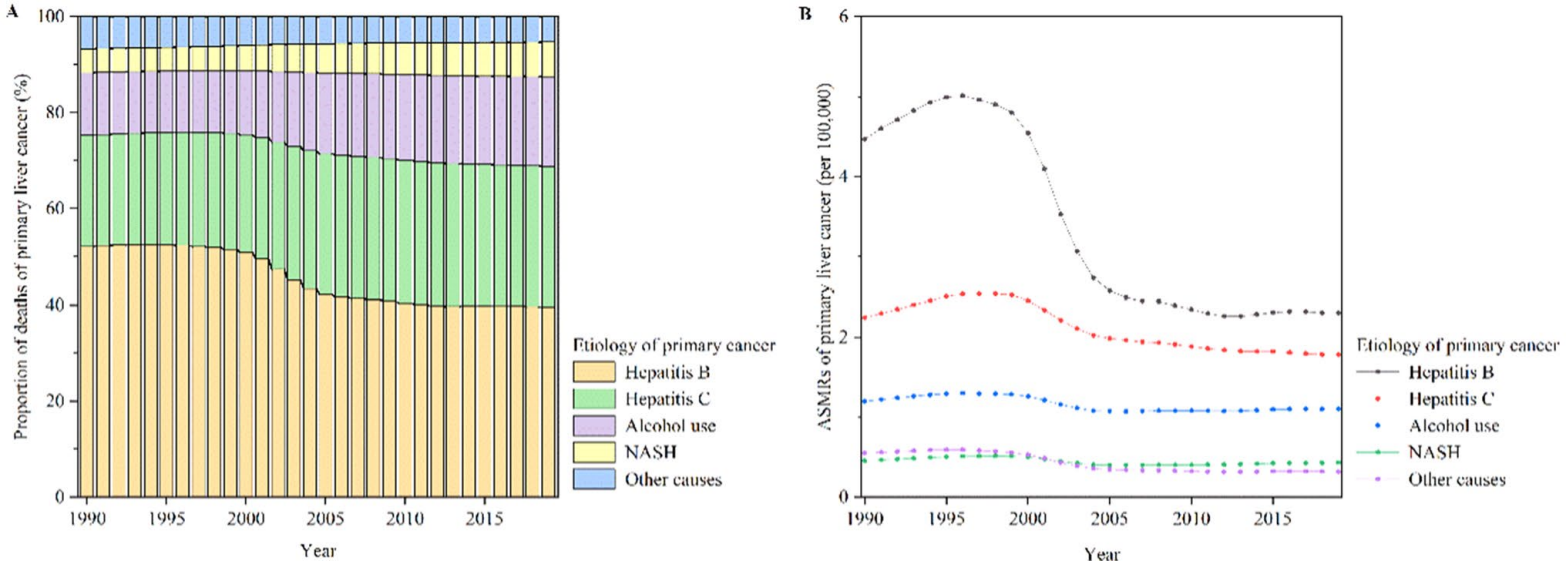
Global proportions of primary liver cancer deaths by etiology and ASMRs of primary liver cancer by etiology between 1990 and 2019. **A** Global proportions of primary liver cancer deaths by etiology between 1990 and 2019; **B** Global ASMRs of primary liver cancer by etiology between 1990 and 2019. Note: *ASMR* age-standardized mortality rate; *NASH* nonalcoholic steatohepatitis

The global deaths of primary liver cancer increased by 32.68% from 365 213 in 1990 to 484 584 in 2019 and the ASMR of primary liver cancer decreased by an average of 2.23% (95% CI 1.83%, 2.63%) between 1990 and 2019 worldwide (Table 2). The global deaths of primary liver cancer by etiology from 1990 to 2019 increased by 0.76% for hepatitis B, 67.50% for hepatitis C, 89.60% for alcohol use, 95.10% for NASH, and 3.91% for other causes (Table 2). In each year from 1990 to 2019, the deaths of primary liver cancer due to hepatitis B and hepatitis C accounted for more than two-thirds of global primary liver cancer deaths, and deaths of primary liver cancer due to alcohol use, NASH, and other causes accounted for less than one-third of global primary liver cancer deaths (Fig. 2A). Similar to the changes in the proportions of incident cases of primary liver cancer by etiology from 1990 to 2019, a decreased proportion of deaths due to hepatitis B and other causes but an increased proportion of deaths due to hepatitis C, alcohol use, and NASH among all deaths of primary liver cancer were observed in this period (Fig. 1B). In 2019, the deaths of primary liver cancer due to hepatitis B, hepatitis C, alcohol use, NASH, and other causes accounted for 39.57%, 29.26%, 18.73%, 7.17%, and 5.27% of the global deaths, respectively (Fig. 2A). The ASMR of primary liver cancer due to hepatitis B was the highest among all etiologies of primary liver cancer worldwide between 1990 and 2019, followed by hepatitis C and alcohol use (Fig. 2B). The ASMRs of primary liver cancer due to hepatitis B, hepatitis C, alcohol use, NASH, and other causes were 2.31 per 100,000, 1.78 per 100,000, 1.10 per 100,000, 0.43 per 100,000, and 0.23 per 100,000 in 2019, respectively (Table 2). The ASMRs of primary liver cancer due to hepatitis B, hepatitis C, alcohol use, NASH, and other causes decreased by an average of 3.46% (95% CI 2.87%, 4.04%), 1.35% (95% CI 1.10%, 1.60%), 0.68% (95% CI 0.48%, 0.88%), 0.74% (95% CI 0.44%, 1.03%), and 2.74% (95% CI 2.29%, 3.20%), respectively (Table 2 and Fig. 2B).

**Table 2:** The deaths and ASMR of primary liver cancer in 1990 and 2019 and their change trends from 1990 to 2019

Characteristics	1990		2019		1990–2019	
	Deaths No. × 10^3^ (95% UI)	ASMR per 100,000 No. (95% UI)	Deaths No. × 10^3^ (95% UI)	ASMR per 100,000 No. (95% UI)	Percentage change in deaths (%)	EAPC in ASMR No. (95% CI)
Overall	365.21 (329.97, 405.77)	8.93 (8.09, 9.90)	484.58 (444.09, 525.80)	5.95 (5.44, 6.44)	32.68	− 2.23 (− 2.63, − 1.83)
Etiology						
Hepatitis B	190.29 (162.33, 222.45)	4.47 (3.82, 5.22)	191.74 (161.86, 223.73)	2.31 (1.95, 2.69)	0.76	− 3.46 (− 4.04, − 2.87)
Hepatitis C	84.67 (73.80, 96.59)	2.25 (1.97, 2.54)	141.81 (121.79, 161.83)	1.78 (1.53, 2.04)	67.50	− 1.35 (− 1.60, − 1.10)
Alcohol use	47.86 (38.59, 58.61)	1.20 (0.97, 1.46)	90.74 (73.35, 109.40)	1.10 (0.89, 1.33)	89.60	− 0.68 (− 0.88, − 0.48)
NASH	17.80 (14.65, 21.51)	0.46 (0.38, 0.55)	34.73 (28.39, 43.18)	0.43 (0.35, 0.53)	95.10	− 0.74 (− 1.03, − 0.44)
Other causes	24.60 (20.58, 29.47)	0.55 (0.46, 0.66)	25.56 (21.23, 30.49)	0.32 (0.27, 0.38)	3.91	− 2.74 (− 3.20, − 2.29)
SDI region						
Low	10.56 (9.24, 11.90)	4.37 (3.80, 4.97)	20.76 (18.22, 23.33)	3.93 (3.49, 4.38)	96.50	− 0.47 (− 0.54, − 0.40)
Low-middle	34.78 (31.45, 38.18)	5.57 (5.05, 6.13)	57.24 (52.13, 63.45)	4.23 (3.86, 4.68)	64.58	− 1.55 (− 1.86, − 1.24)
Middle	163.81 (142.72, 189.46)	15.00 (13.15, 17.26)	196.96 (172.83, 223.21)	7.92 (6.97, 8.93)	20.24	− 3.16 (− 3.73, − 2.58)
Middle-high	107.83 (94.48, 122.72)	9.96 (8.75, 11.27)	97.19 (87.23, 108.11)	4.83 (4.34, 5.38)	− 9.87	− 3.69 (− 4.25, − 3.13)
High	48.13 (46.47, 49.30)	4.69 (4.54, 4.81)	112.24 (102.49, 118.74)	5.89 (5.44, 6.21)	133.20	0.42 (− 0.01, 0.85)
GBD region						
High-income Asia Pacific	23.59 (22.76, 24.37)	11.62 (11.18, 12.00)	49.68 (43.78, 53.50)	10.78 (9.77, 11.53)	110.62	− 0.86 (− 1.49, − 0.22)
Central Asia	1.51 (1.35, 1.66)	3.24 (2.89, 3.58)	6.19 (5.39, 7.08)	8.72 (7.63, 9.88)	310.70	2.93 (2.39, 3.47)
East Asia	237.01 (202.34, 279.89)	25.52 (21.98, 29.94)	193.86 (163.85, 228.76)	9.39 (7.98, 11.03)	− 18.20	− 4.98 (− 5.77, − 4.18)
South Asia	15.85 (13.38, 18.09)	2.82 (2.33, 3.27)	38.65 (33.52, 44.56)	2.81 (2.43, 3.24)	143.79	− 0.06 (− 0.17, 0.04)
Southeast Asia	17.57 (15.68, 19.28)	6.76 (6.03, 7.45)	42.86 (35.33, 51.52)	7.33 (6.08, 8.79)	143.90	0.28 (0.20, 0.36)
Australasia	0.46 (0.44, 0.48)	1.98 (1.90, 2.06)	2.01 (1.83, 2.17)	4.12 (3.80, 4.46)	332.62	2.88 (2.66, 3.10)
Caribbean	1.64 (1.52, 1.74)	6.29 (5.85, 6.68)	1.69 (1.42, 2.01)	3.29 (2.76, 3.89)	3.58	− 2.16 (− 3.01, − 1.31)
Central Europe	8.11 (7.83, 8.32)	5.60 (5.38, 5.75)	7.20 (6.22, 8.33)	3.36 (2.90, 3.90)	− 11.24	− 1.49 (− 1.89, − 1.09)
Eastern Europe	4.22 (4.06, 4.41)	1.55 (1.49, 1.62)	9.68 (8.51, 11.12)	2.87 (2.51, 3.29)	129.05	2.52 (2.26, 2.78)
Western Europe	19.88 (19.16, 20.42)	3.43 (3.31, 3.52)	40.30 (37.22, 42.88)	4.41 (4.10, 4.68)	102.67	0.80 (0.65, 0.95)
Andean Latin America	1.07 (0.95, 1.21)	5.23 (4.60, 5.87)	1.84 (1.51, 2.23)	3.34 (2.73, 4.03)	71.18	− 1.94 (− 2.39, − 1.49)
Central Latin America	3.07 (2.86, 3.23)	3.74 (3.46, 3.94)	8.42 (7.36, 9.75)	3.65 (3.18, 4.22)	173.76	0.04 (− 0.29, 0.37)
Southern Latin America	0.76 (0.68, 0.83)	1.65 (1.49, 1.81)	2.03 (1.90, 2.15)	2.41 (2.26, 2.56)	168.33	2.00 (1.77, 2.24)
Tropical Latin America	1.88 (1.80, 1.95)	2.09 (1.99, 2.17)	5.94 (5.54, 6.24)	2.50 (2.32, 2.62)	215.17	1.06 (0.91, 1.22)
North Africa and Middle East	10.91 (9.58, 12.23)	6.39 (5.55, 7.19)	26.43 (21.21, 32.61)	6.20 (5.06, 7.62)	142.20	0.25 (0.10, 0.40)
High-income North America	7.07 (6.78, 7.25)	2.03 (1.95, 2.07)	26.48 (23.64, 28.91)	4.29 (3.83, 4.68)	274.32	2.66 (2.51, 2.81)
Oceania	0.11 (0.09, 0.13)	3.85 (3.25, 4.47)	0.23 (0.19, 0.28)	3.46 (2.93, 4.09)	106.99	− 0.22 (− 0.29, − 0.16)
Central Sub-Saharan Africa	0.72 (0.60, 0.85)	2.85 (2.43, 3.29)	1.39 (1.11, 1.75)	2.47 (1.99, 3.07)	94.59	− 0.64 (− 0.71, − 0.57)
Eastern Sub-Saharan Africa	2.54 (2.08, 3.15)	3.15 (2.63, 3.99)	5.68 (4.68, 6.92)	3.41 (2.85, 4.15)	123.76	0.08 (− 0.05, 0.22)
Southern Sub-Saharan Africa	1.91 (1.35, 3.14)	6.74 (4.71, 11.07)	4.04 (3.62, 4.54)	7.05 (6.31, 7.91)	111.14	− 0.43 (− 1.04, 0.19)
Western Sub-Saharan Africa	5.31 (4.55, 6.17)	5.81 (4.99, 6.76)	9.97 (8.36, 11.56)	5.29 (4.48, 6.04)	87.85	− 0.44 (− 0.52, − 0.35)

*ASMR* age-standardized mortality rate; *CI* confidence interval; *EAPC* estimated annual percentage changes; *GBD* Global Burden of Disease; *NASH* nonalcoholic steatohepatitis; *SDI* socio-demographic index; *UI* uncertainty interval

### Regional Trends in the Incidence and Mortality of Primary Liver Cancer and Its Underlying Etiologies

**Fig. 3:**
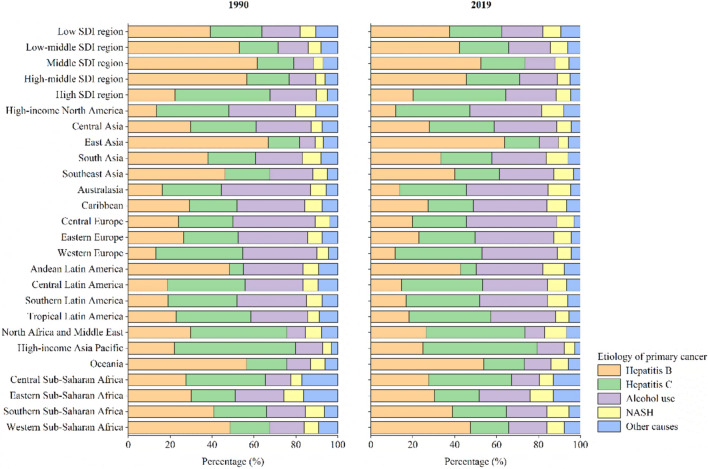
Contribution of hepatitis B, hepatitis C, alcohol use, NASH and other causes to absolute incident cases of primary liver cancer by region in 1990 and 2019. Note: *NASH* nonalcoholic steatohepatitis, *SDI* socio-demographic index

The incident cases of primary liver cancer by SDI region decreased by 1.70% in middle-high SDI region but increased by 97.99%, 62.17%, 26.77%, and 161.98% in low, low-middle, middle- and high SDI regions from 1990 to 2019, respectively (Table 1). The ASIR of primary liver cancer decreased in low (EAPC = − 0.46; 95% CI − 0.52, − 0.40), low-middle (EAPC = − 1.59; 95% CI − 1.90, − 1.27), middle (EAPC = − 2.98; 95% CI − 3.56, − 2.39), and middle-high (EAPC = − 3.33; 95% CI − 3.89, − 2.77) SDI regions, but increased in high SDI region by an average of 0.91% (95% CI 0.47%, 1.35%) between 1990 and 2019 (Table 1). In low, low-middle, middle, and middle-high SDI regions, hepatitis B was the major etiology for the incident cases of primary liver cancer in 1990 and 2019, accounting for 37.63%, 42.28%, 52.38%, and 45.59% of the incident cases of all etiologies in these regions in 2019, respectively (Fig. 3A and B). Hepatitis C was the major etiology for the incident cases of primary liver cancer in high SDI region in 1990 and 2019, accounting for 44.21% of the incident cases of all etiologies in 2019 (Fig. 3A and B).

The incident cases of primary liver cancers by GBD region decreased in Central Europe (10.30%) and East Asia (10.09%), but increased in more than 90% (19/21) of the regions, with the largest increase in Australasia (354.16%), followed by High-income North America (311.64%) and Central Asia (309.21%) (Table 1). High-income Asia Pacific suffered the severest threat of primary liver cancer in 2019 (ASIR: 15.56 per 100,000), followed by East Asia (ASIR: 10.43 per 100,000) and Central Asia (ASIR: 8.27 per 100,000). The ASIR of primary liver cancer increased in more than two-fifths (9/21) of GBD regions, with the largest increase in Australasia (EAPC = 3.14; 95% CI 2.87, 3.41), followed by High-income North America (EAPC = 2.99; 95% CI 2.79, 3.20) and Central Asia (EAPC = 2.78; 95% CI 2.23, 3.32) (Table 1). The major etiology for the incident cases of primary liver cancer varied considerably across GBD regions (Fig. 3A and B). The major etiology for the incident cases of primary liver cancer varied considerably across GBD regions, with hepatitis B as the major etiology in East Asia, South Asia, Southeast Asia, Andean Latin America, Oceania, Eastern Sub-Saharan Africa, Southern Sub-Saharan Africa, and Western Sub-Saharan Africa, and hepatitis C as the major etiology in High-income North America, Central Asia, Western Europe, Central Latin America, Tropical Latin America, North Africa and Middle East, High-income Asia Pacific, and Central Sub-Saharan Africa in 1990 and 2019 (Fig. 3A and B). In 2019, the incident cases of primary liver cancer due to hepatitis B accounted for 63.78%, 33.28%, 40.04%, 42.67%, 53.79%, 30.26%, 38.83%, and 47.52% of the incident cases of all etiologies in East Asia, South Asia, Southeast Asia, Andean Latin America, Oceania, Eastern Sub-Saharan Africa, Southern Sub-Saharan Africa, and Western Sub-Saharan Africa, respectively (Fig. 3B). The incident cases of primary liver cancer due to hepatitis C accounted for 35.44%, 31.00%, 41.32%, 38.78%, 39.14%, 47.02%, 54.63%, and 39.72% of the incident cases of all etiologies in High-income North America, Central Asia, Western Europe, Central Latin America, Tropical Latin America, North Africa and Middle East, High-income Asia Pacific, and Central Sub-Saharan Africa in 2019, respectively (Fig. 3B). In Southern Latin America, the major etiology of the incident cases of primary liver cancer was alcohol use in 1990 and hepatitis C in 2019, accounting for 33.16% and 35.10% of incident cases of all etiologies, respectively (Fig. 3A and B). Alcohol use was the major etiology of incident cases of primary liver cancer in Australasia, Caribbean, Central Europe and Eastern Europe in 1990 and 2019, accounting for 39.10%, 34.94%, 43.23%, and 37.79% of the incident cases of all etiologies in these regions in 2019, respectively (Fig. 3A and B). The proportion of incident cases of primary liver cancer due to NASH increased from 1990 to 2019 in several GBD regions and exceeded 10% in 2019, such as Eastern Sub-Saharan Africa, Australasia, and South Asia (Fig. 3A and B). The trends in incident cases and ASIRs of primary liver cancer by etiology varied considerably across regions between 1990 and 2019, including SDI and GBD regions (Tables S2–S6).

**Fig. 4:**
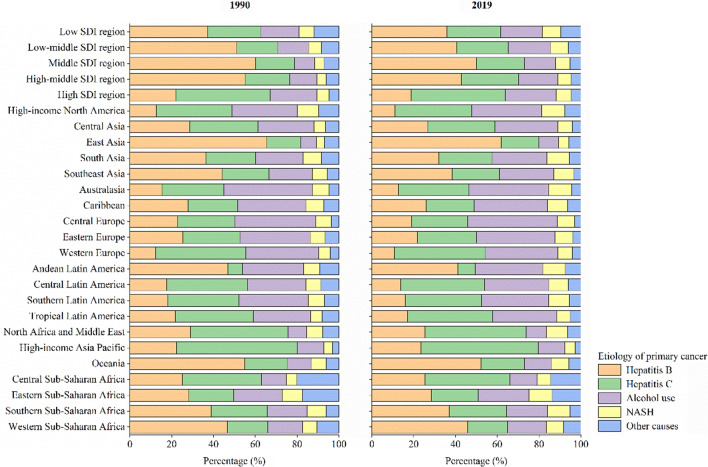
Contribution of hepatitis B, hepatitis C, alcohol use, NASH and other causes to absolute deaths of primary liver cancer by region in 1990 and 2019. Note: *NASH* nonalcoholic steatohepatitis, *SDI* socio-demographic index

Across the 5 SDI regions, the deaths of primary liver cancer decreased in middle-high SDI region (9.87%) from 1990 to 2019, but increased in low, low-middle, middle, and high SDI regions in this period, with increases of 96.50%, 64.58%, 20.24%, and 133.20%, respectively (Table 2). The ASMR of primary liver cancer decreased by an average of 0.47% (95% CI: 0.40%, 0.54%), 1.55% (95% CI: 1.24%, 1.86%), 3.16% (95% CI: 2.58%, 3.73%), and 3.69% (95% CI: 3.13%, 4.25%), but was stable in high SDI region (EAPC = 0.42, 95% CI − 0.01, 0.85) (Table 2). The major cause of deaths from primary liver cancer in 1990 and 2019 was hepatitis B in low, low-middle, middle, and middle-high SDI regions, accounting for 37.05%, 28.46%, 25.43%, and 52.28% of death of all etiologies in 2019, respectively, whereas hepatitis C was the major cause of deaths from primary liver cancer in high SDI region, accounting for 56.28% of deaths of all etiologies in 2019 (Fig. 4A and B).

Across 21 SDI regions, the deaths of primary liver cancer decreased in Central Europe (25.65%), East Asia (14.11%), and Caribbean (2.09%) from 1990 to 2019, but increased in the remaining 18 GBD regions, with the largest increase in Australasia (282.73%), followed by Central Asia (282.60%) and High-income North America (258.34%) in this period (Table 2). Among all GBD regions, the largest ASMR of primary liver cancer was observed in High-income Asia Pacific (ASMR: 10.78 per 100,000 in 2019), followed by East Asia (ASMR: 9.39 per 100,000 in 2019) and Central Asia (ASMR: 8.27 per 100,000 in 2019) (Table 2). The ASMR of primary liver cancer increased in 9 GBD regions, with the largest increase in Central Asia (EAPC = 2.93; 95% CI 2.39, 3.47), followed by Australasia (EAPC = 2.88; 95% CI 2.66, 3.10) and High-income North America (EAPC = 2.66; 95% CI 2.51, 2.81) (Table 2). Similar to the major etiology for the incident cases of primary liver cancer, the major etiology for the deaths of primary liver cancer was also hepatitis B or hepatitis C in more than four-fifths (17/21) of GBD regions in 1990 and 2019, such as GBD regions in Asia, Latin America, and Sub-Saharan Africa (Fig. 4A and B). In Australasia, Caribbean, Central Europe and Eastern Europe, alcohol use was the major cause of primary liver cancer deaths in 1990 and 2019, accounting for 38.14%, 35.07%, 42.94%, and 37.63% of the deaths of all etiologies in these regions in 2019, respectively (Fig. 4A and B). Similar to the trends in incident cases and ASIRs of primary liver cancer by etiology, the trends in deaths and ASMRs also varied considerably across regions between 1990 and 2019, including SDI and GBD regions (Tables S7–S11).

### National Trends in the Incidence and Mortality of Primary Liver Cancer and Its Underlying Etiologies

**Fig. 5:**
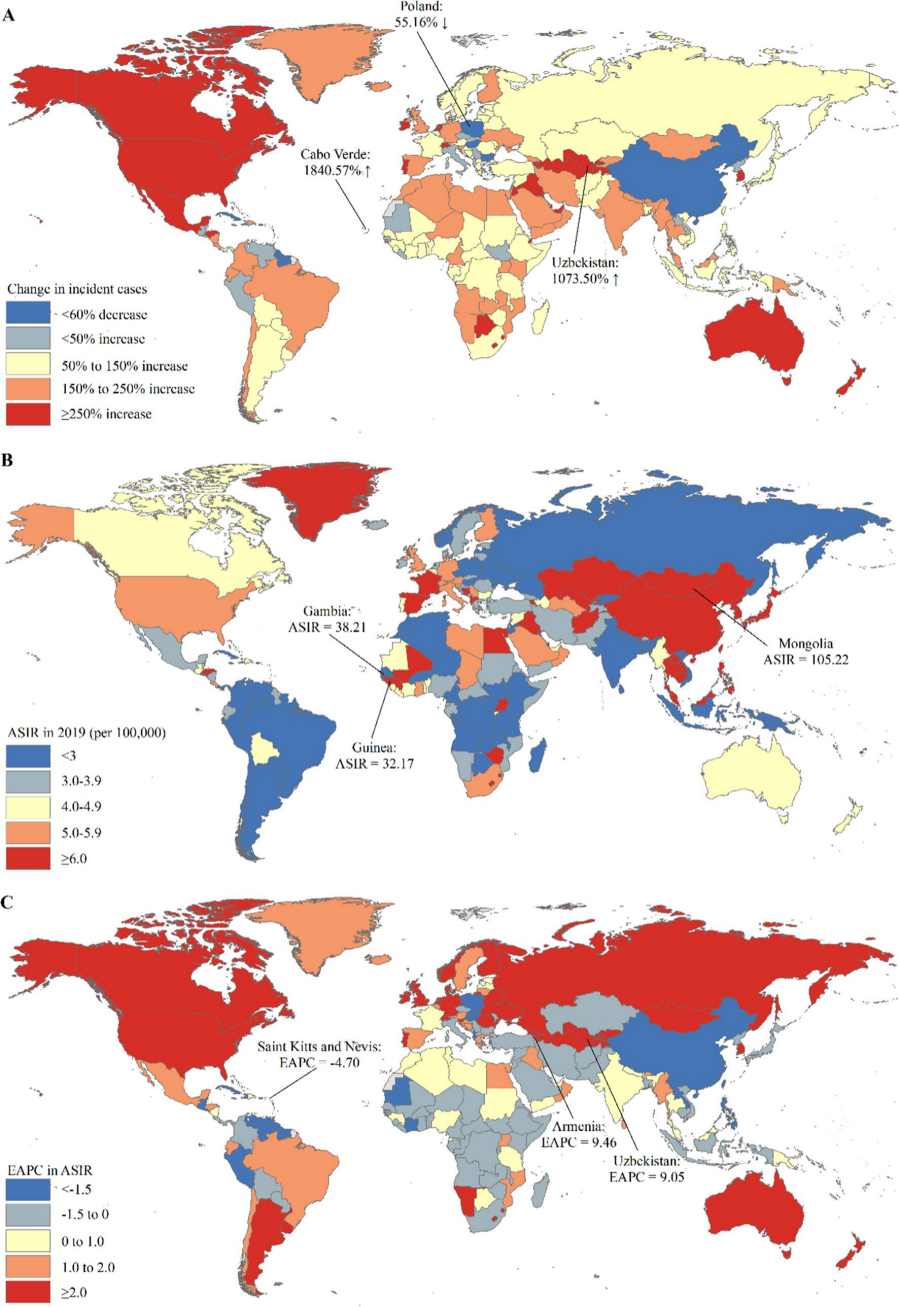
The global trends in the incidence of primary liver cancer in 204 countries and territories. **A** The percentage change in incident cases of primary liver cancer between 1990 and 2019; **B** The ASIR of primary liver cancer in 2019; **C** The EAPCs in ASIR of primary liver cancer from 1990 to 2019. Note: *ASIR* age-standardized incidence rate; *EAPC* estimated annual percentage change

Among 204 countries and territories, the absolute number of incident cases of primary liver cancer in China (210,462) accounted for 39.39% of the global incident cases (534,364) in 2019 (Table S12). The country with the most pronounced increase in the incident cases of primary liver cancer was Cabo Verde (1840.57%) followed by Uzbekistan (1073.50%), and Poland had the most pronounced decrease in the incident cases of primary liver cancer (Table S12 and Fig. 5A). The ASIR of primary liver cancer varied considerably across the world, with the largest ASIR in Mongolia (105.22 per 100,000), followed by Gambia (38.21 per 100,000) and Guinea (32.17 per 100,000) in 2019 (Table S12 and Fig. 5B). Mongolia was also the country suffering the largest burden of primary liver cancer by etiology, with the highest ASIR of primary liver cancer by etiology in 2019 (Tables S13–S17 and Figs. S1–S5). The ASIR of primary liver cancer was deemed to be in a decreasing trend in 89 countries or territories, with the largest decrease in Saint Kitts and Nevis (EAPC = − 4.70; 95% CI − 5.77, − 3.62) (Table S12 and Fig. 5C). The ASIR of primary liver cancer was deemed to be in an increasing trend in 91 countries or territories, with the largest increase in Armenia (EAPC = 9.46; 95% CI 7.92, 11.02) and Uzbekistan (EAPC = 9.05; 95% CI 7.82, 10.28) (Table S12 and Fig. 5C). The ASIR of primary liver cancer remained stable in 24 countries or territories, such as Djibouti, Nigeria, and Botswana (Table S12). Across 204 countries and territories, the trends in incident cases and ASIRs of primary liver cancer by etiology varied considerably between 1990 and 2019 (Tables S13–S17 and Figs. S1–S5). An increasing trend in ASIRs of primary liver cancer by etiology between 1990 and 2019 was observed in 71 countries and territories, such as Armenia, Australia, Canada, and the United States of America (USA) (Tables S13–S17 and Figs. S1–S5). The ASIRs of primary liver cancer by etiology between 1990 and 2019 were deemed to be in a decreasing trend in 54 countries and territories, such as Poland, China, Belize, and Dominica (Tables S13–S17 and Figs. S1–S5). The ASIR of primary liver cancer by etiology remained stable in Jamaica and Kenya (Tables S13-S17 and Figs. S1–S5). Different trends in ASIRs of primary liver cancer by etiology were observed in the remaining 77 countries and territories, such as Malaysia, Pakistan, Qatar, and Thailand (Tables S13–S17 and Figs. S1–S5).

**Fig. 6:**
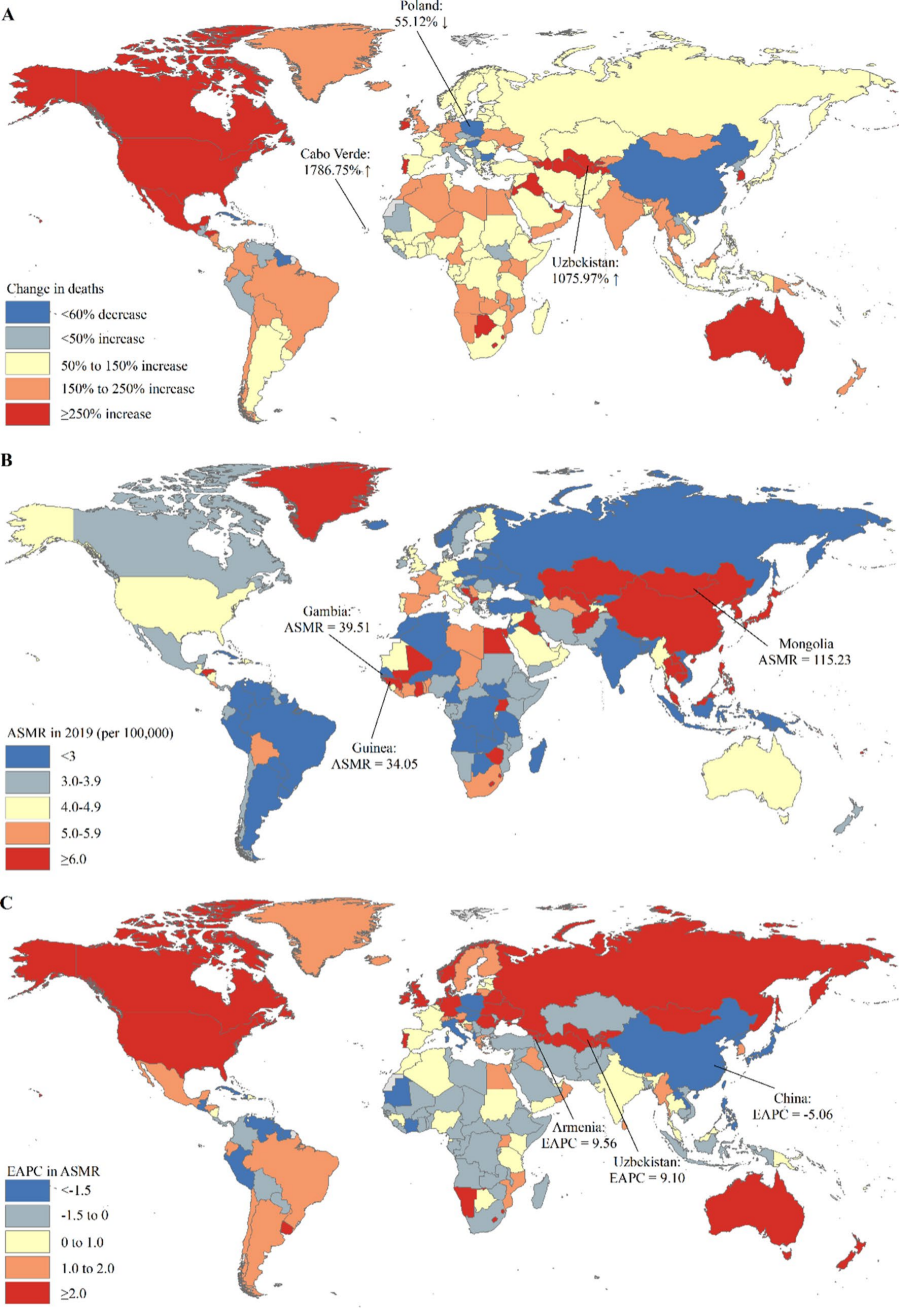
The global trends in the mortality of primary liver cancer in 204 countries and territories. **A** The percentage change in deaths of primary liver cancer between 1990 and 2019; **B** The ASMR of primary liver cancer in 2019; **C** The EAPCs in ASMR of primary liver cancer from 1990 to 2019. Note: *ASMR*: age-standardized mortality rate; *EAPC* estimated annual percentage change

For the deaths of primary liver cancer among 204 countries and territories in 2019, China had the largest deaths of primary liver cancer (187,700), which accounted for 38.73% of the global deaths in 2019 (Table S18). The country with the most pronounced increase in the deaths of primary liver cancer was Cabo Verde (1786.75%) followed by Uzbekistan (1075.97%), and Poland had the most pronounced decrease in the deaths of primary liver cancer (Table S18 and Fig. 6A). Across 204 countries and territories, Mongolia (115.23 per 100,000) had the largest ASMR of primary liver cancer in 2019, followed by Gambia (39.51 per 100,000) and Guinea (34.05 per 100,000) in 2019 (Table S18 and Fig. 6B). Mongolia also suffered the highest ASMR of primary liver cancer by etiology in 2019 (Tables S19–S23 and Figs. S6–S10). The ASMR of primary liver cancer was deemed to be in a decreasing trend in 91 countries or territories, with the largest decrease in China (EAPC = − 5.60; 95% CI − 5.8, − 4.24) (Table S18 and Fig. 6C). The ASMR of primary liver cancer was deemed to be in an increasing trend in 79 countries or territories, with the largest increase in Armenia (EAPC = 9.56; 95% CI 7.95, 11.20) and Uzbekistan (EAPC = 9.10; 95% CI 7.95, 10.27) (Table S18 and Fig. 6C). The ASMR of primary liver cancer remained stable in 34 countries or territories, such as Israel, United Arab Emirates, and Kenya (Table S18). Similar to the trends in incident cases and ASIRs of primary liver cancer by etiology at the national level, the trends in deaths and ASMRs of primary liver cancer by etiology varied considerably in 204 countries and territories (Tables S19–S23 and Figs. S6–S10).

### Associations of SDI and UHCI with EAPC in ASIR and ASMR of Primary Liver Cancer and Its Underlying Etiologies

**Fig. 7:**
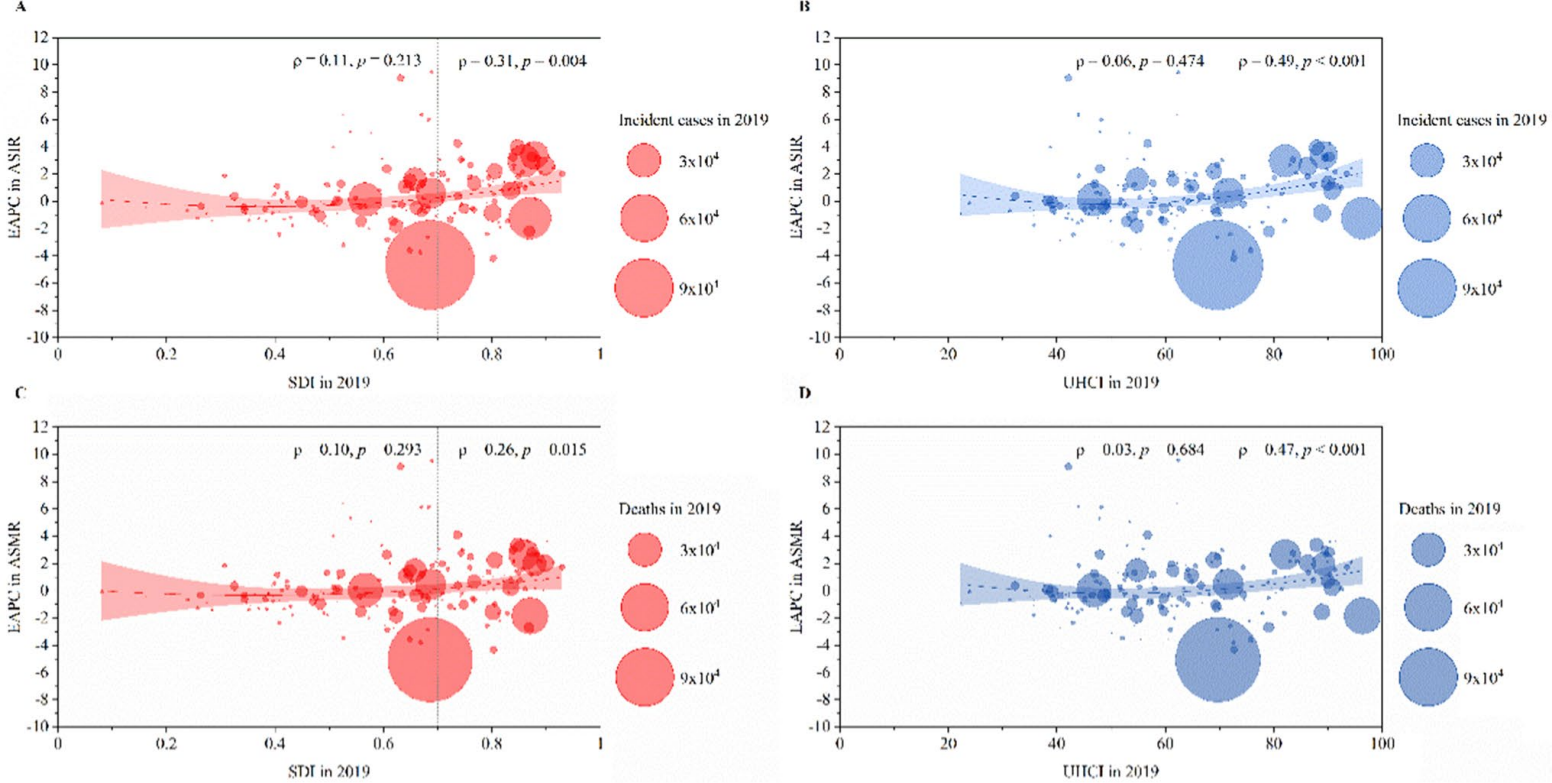
The EAPCs in ASIRs and ASMRs of primary liver cancer at the national levels. **A** The correlation of EAPC in ASIRs between 1990 and 2019 with SDI in 2019. **B** The correlation of EAPC in ASIRs between 1990 and 2019 with UHCI in 2019. **C** The correlation of EAPC in ASMRs between 1990 and 2019 with SDI in 2019. **D** The correlation of EAPC in ASMRs between 1990 and 2019 with UHCI in 2019. The incident cases and deaths of primary liver cancer from 204 countries and territories in 2019 are represented by circles. The size of the circles increased with the incident cases and deaths. The ρ indices and p values were derived from Pearson correlation analysis. Note: *ASIR* age-standardized incidence rate; *ASMR* age-standardized mortality rate; *EAPC* estimated annual percentage change

We found a significant positive correlation of SDI in 2019 with EAPC in ASIR (ρ = 0.31, *p* = 0.004) and EAPC in ASMR (ρ = 0.26, *p* = 0.015) of primary liver cancer in the countries and territories with an SDI value ≥ 0.7, and a nonsignificant correlation in the countries and territories with an SDI value < 0.7 (Fig. 7A and C). A significant positive correlation of UHCI in 2019 with EAPC in ASIR (ρ = 0.49, *p* < 0.001) and EAPC in ASMR (ρ = 0.47, *p* < 0.001) of primary liver cancer was observed in the countries and territories with a UHCI value ≥ 70, while there was a nonsignificant correlation in the countries and territories with a UHCI < 70 (Fig. 7B and D). Similar to the correlations of SDI and UHCI in 2019 with ASIR and ASMR of primary liver cancer, a positive correlation of SDI and UHCI in 2019 with ASIR and ASMR of primary liver cancer by etiology was observed in the countries and territories with an SDI value ≥ 0.7 or a UHCI value ≥ 70, respectively (Figs. S11–S15).

## Discussion

This study used data from the GBD study 2019 to comprehensively assess the landscape and long-term trends in the incidence and mortality of primary liver cancer and its underlying etiologies, including hepatitis B, hepatitis C, alcohol use, NASH, and other causes, as well as the associations of the trends in incidence and mortality with socioeconomic status at the national level. We found an increasing trend in both the incident cases and deaths of primary liver cancer and its underlying etiologies and a decreasing trend in both ASIR and ASMR of primary liver cancer and its underlying etiologies worldwide between 1990 and 2019. The dominant etiology of the global incident cases and deaths of primary liver cancer was hepatitis B, followed by hepatitis C, alcohol use, NASH, and other causes in 1990 and 2019. However, the largest increase in the percentage change in global incident cases and deaths of primary liver cancer by etiology from 1990 to 2019 was primary liver cancer due to NASH, followed by alcohol use, hepatitis C, other causes, and hepatitis B. We found that the temporal trends in the incidence and mortality of primary liver cancer and its underlying etiologies varied considerably between regions and countries. For example, high SDI region had both the largest increase in the percentage change in incident cases and deaths of primary liver cancer and suffered an increasing trend in ASIR and a stable trend in ASMR of primary liver cancer between 1990 and 2019. More than two-fifths of GBD regions suffered an increase in both ASIR and ASMR of primary live cancer between 1990 and 2019, with the largest increase in ASIR in Australasia and the largest increase in ASMR in Central Asia. Notably, we found a positive correlation of EAPC in ASIRs and ASMRs of primary liver cancer with SDI values and UHCI values in countries or territories with a high SDI value (≥ 0.7) or a high UHCI value (≥ 70).

This study found an increase of 43.11% for the absolute numbers of incident cases and 32.68% for the absolute numbers of deaths of primary liver cancer from 1990 to 2019 worldwide. The increased trends in incident cases and deaths of primary liver cancer could be mainly driven by both aging and growth of the population worldwide [7, 25–27]. We found that both the incident cases and deaths of primary liver cancer related to hepatitis C, alcohol use, and NASH increased markedly from 1990 to 2019, leading to an increase in their proportions in the global incident cases and deaths of primary liver cancer, respectively. However, the global predominant cause of primary liver cancer remained hepatitis B, accounting for 40.95% of the incident cases and 39.57% of deaths of primary liver cancer in 2019. Hepatitis C was the second leading cause of primary liver cancer after hepatitis B, accounting for 28.49% of the incident cases and 29.26% of deaths of primary liver cancer in 2019. Thus, although the relevance of nonviral risk factors such as alcohol use and NASH is becoming more important for the burden of primary liver cancer, elimination of viral hepatitis remains the key strategy for primary prevention of primary liver cancer globally. Effective strategies for blocking the transmission of hepatitis B include infant vaccination, screening of blood donations, and safer sex practices [28, 29]. The WHO reported that the coverage for the third dose of hepatitis B vaccine reached 85% by the end of 2019, up from only 30% in 2000; the coverage rate of the timely birth dose of hepatitis B vaccine in 2019 was 43%; and the blood donations screened with quality assurance accounted for 97% of all donations in 2015 [29]. Currently, a cure for chronic HCV infection can be achieved with direct-acting antivirals, but there is no effective vaccine against hepatitis C [30, 31]. The effective measures for HCV control include screening of transfusions, prevention of mother-to-child transmission, provision of clean needles, and infection control in health care facilities [32]. In this study, we found a decreasing trend in both ASIR and ASMR of primary liver cancer between 1990 and 2019, as well as a decreasing trend in all ASIRs and ASMRs of primary liver cancer by etiology worldwide. The decreased trend in ASIR of primary liver cancer was contributed by advances in the prevention and control of its risk factors, such hepatitis B and hepatitis C [13, 14]. Due to the implementation of HBV interventions, such as vaccination, testing, and treatment, the chronic HBV prevalence was 4.1% in 2019 worldwide, with a 31.3% decrease from 6.0% in 1990 [13]. The global age-standardized prevalence rate of HCV infection decreased by an average of 0.67% per year between 1990 and 2019 [14]. In addition to the reduction in the incidence of primary liver cancer, advances in the early detection and effective treatment of primary liver cancer globally during the past decades also possibly contributed to the decreasing trend in ASMR of primary liver cancer [33].

In line with previous studies [7, 9, 17], we found that the most common etiology for primary liver cancer varied between regions. Across 5 SDI regions, the predominant etiology for primary liver cancer both in 1990 and 2019 was hepatitis B in low, low-middle, middle, and middle-high SDI regions, but hepatitis C in high SDI region. Across 21 GBD regions, the predominant etiology of primary liver cancer in 2019 was hepatitis B in East Asia, South Asia, Southeast Asia, Andean Latin America, Oceania, Eastern Sub-Saharan Africa, Southern Sub-Saharan Africa, and Western Sub-Saharan Africa; hepatitis C in High-income North America, Central Asia, Western Europe, Central Latin America, Tropical Latin America, Southern Latin America, North Africa and Middle East, High-income Asia Pacific, and Central Sub-Saharan Africa; and alcohol use in Australasia, Caribbean, Central Europe, and Eastern Europe. The disparities in the major etiology of primary liver cancer from region to region largely reflected a heterogeneous pattern in risk factor exposures across the world [4, 8, 12–14, 34]. For example, the regions with hepatitis B as the etiology for primary liver cancer were usually regions with high levels of HBV infection, such as East Asia, South Asia, and Southeast Asia [13, 35]. Thus, elimination of viral hepatitis remains the key strategy for reducing the burden of primary liver cancer at the regional level, especially in the predominant etiology of hepatitis B or hepatitis C. In addition to the efforts to eliminate viral hepatitis, additional nonviral causes of primary liver cancer must also be incorporated into planning for primary liver cancer control in various regions. For example, in 2019, alcohol use was associated with more than 35% of the incident cases in Europe and Australasia, and NASH was associated with more than 10% of the incident cases of primary liver cancer in several regions such as Eastern Sub-Saharan Africa and Australasia, yet cost-effective policies exist to reduce consumption in the population [36].

The current study found an increasing trend in ASIR of primary liver cancer between 1990 and 2019 in nearly half (91/204) of the countries and territories included in the GBD study. More than one-third (71/204) of the countries and territories included in the GBD study also suffered an increasing trend in ASIRs of primary liver cancer by etiology, such as the USA, Australia, and Canada. In addition, a significant positive correlation was detected between SDI/UHCI in 2019 and EAPC in ASIR of primary liver cancer among the countries or territories with an SDI ≥ 0.7 or UHCI ≥ 70. The main explanation could be that increasing age could be directly correlated with liver cancer incidence in most populations worldwide [37]. Furthermore, migration has likely influenced the incidence of primary liver cancer among ethnic minorities in Western countries, as observed in the USA, Australia, and Canada, where the highest incidence was among migrants from high-risk countries [38–41]. We also found a decreasing trend in ASIRs of primary liver cancer by etiology in more than a quarter of the countries and territories included in the GBD study, such as Poland, China, and Belize. The favorable decreasing trends in ASIRs of primary liver cancer by etiology in these countries reflected the achievements in the prevention and control for the related risk factors, such as viral hepatitis, alcohol use, and metabolic conditions (including obesity, diabetes, and non-alcoholic fatty liver disease). For example, China has changed from a highly endemic area into an intermediate endemic area for HBV infection based on remarkable progress in HBV prevention and control and critical progress in hepatitis B treatment [42–44]. Despite the ASIRs of primary liver cancer and its etiologies significantly declining in the past three decades, China had the largest numbers of incident cases and deaths of primary liver cancer in 2019 across the world. Recently, one study predicted that while the impact of HBV and HCV elimination efforts is only beginning to be reflected in the burden of primary liver cancer today, the increasing prevalence of other risk factors might drive future changes in incidence of primary liver cancer [7]. To reduce the burden of primary liver cancer, control efforts for its causes should be prioritized at the national level. In addition, public health officials must prepare for an increase in demand for resources to manage the care of patients with primary liver cancer throughout the cancer pathway.

This current study comprehensively assessed the global landscape and long-term trends in the incidence and mortality of primary liver cancer and its underlying etiologies as well as the associations of the trends in incidence and mortality with socioeconomic status at the national level over the past three decades. However, several limitations should be noted. First, the availability of data and the quality of available data limited the accuracy and robustness of the estimates of the incidence and mortality of primary liver cancer and its etiologies in the modeling [9], which might bias when national surveillance and population-based studies were lacking. Second, EAPC in ASIRs and ASMRs as well as relative change in the incident cases and deaths of primary liver cancer and its etiologies were used to assess its long-term trends from 1990 to 2019, which might mask the recent short-term trends that reflected the effectiveness of the recent prevention interventions.

In summary, primary liver cancer remains a major public health concern globally. Although the trend in ASIR and ASMR of primary liver cancer decreased between 1990 and 2019, both the trend in the numbers of incident cases and deaths increased from 1990 to 2019. In addition, the trends in ASIR and ASMRs of primary liver cancer and its etiologies varied between regions and countries. In high SDI region, there was an increasing trend in ASIR and a stable trend in ASMR of primary liver cancer in the past three decades. There was an increasing trend in ASIR of primary liver cancer in nearly half of the countries and an increasing trend in ASIRs of primary liver cancer by etiology in more than one-third of the countries worldwide. In line with the SDGs, the identification and elimination of risk factors for primary liver cancer will be required to achieve a sustained reduction in liver cancer burden.

## Supplementary Information

Below is the link to the electronic supplementary material.

Supplementary file1 (DOCX 6625 KB)

## Data Availability

The data are available from an open access database, the GHDx query tool (https://vizhub.healthdata.org/gbd-results/).

## Abbreviations

ASIR: Age-standardized incidence rate
ASMR: Age-standardized mortality rates
CI: Confidence interval
EAPC: Estimated annual percentage change
GBD: Global burden of disease
HBV: Hepatitis B virus
HCV: Hepatitis C virus
NASH: Nonalcoholic steatohepatitis
SDI: Socio-demographic index
SDG: Sustainable development goal
UHCI: Universal health coverage index
UI: Uncertainty interval
WHO: World Health Organization
