# Supplementary material for: Global, Regional, and National Trends in Incidence and Mortality of Primary Liver Cancer and Its Underlying Etiologies from 1990 to 2019: Results from the Global Burden of Disease Study 2019
Source: J Epidemiol Glob Health. 2023 May 13;13(2):344–60. doi: 10.1007/s44197-023-00109-0 (PMC10271958; doi:10.1007/s44197-023-00109-0)
Supplement: Supplementary file 1 — Supplementary file1 (DOCX 6625 KB) [file 44197_2023_109_MOESM1_ESM.docx]

**Supplementary appendix**

Table S1. The SDI value and UHCI of 204 countries and territories in 2019.

Table S2. The incident cases and ASIR of primary liver cancer due to hepatitis B in 1990 and 2019 and their change trends from 1990 to 2019.

Table S3. The incident cases and ASIR of primary liver cancer due to hepatitis C in 1990 and 2019 and their change trends from 1990 to 2019.

Table S4. The incident cases and ASIR of primary liver cancer due to alcohol use in 1990 and 2019 and their change trends from 1990 to 2019.

Table S5. The incident cases and ASIR of primary liver cancer due to NASH in 1990 and 2019 and their change trends from 1990 to 2019.

Table S6. The incident cases and ASIR of primary liver cancer due to other causes in 1990 and 2019 and their change trends from 1990 to 2019.

Table S7. The deaths and ASMR of primary liver cancer due to hepatitis B in 1990 and 2019 and their change trends from 1990 to 2019.

Table S8. The deaths and ASMR of primary liver cancer due to hepatitis C in 1990 and 2019 and their change trends from 1990 to 2019.

Table S9. The deaths and ASMR of primary liver cancer due to alcohol use in 1990 and 2019 and their change trends from 1990 to 2019.

Table S10. The deaths and ASMR of primary liver cancer due to NASH in 1990 and 2019 and their change trends from 1990 to 2019.

Table S11. The deaths and ASMR of primary liver cancer due to other causes in 1990 and 2019 and their change trends from 1990 to 2019.

Table S12. The incident cases and ASIR of primary liver cancer in 1990 and 2019 and their change trends from 1990 to 2019 at national level.

Table S13. The incident cases and ASIR of primary liver cancer due to hepatitis B in 1990 and 2019 and their change trends from 1990 to 2019 at national level.

Figure S1. The global trends in the incidence of primary liver cancer due to hepatitis B in 204 countries and territories. (A) The percentage change in incident cases of primary liver cancer between 1990 and 2019; (B) The ASIR of primary liver cancer in 2019; (C) The EAPCs in ASIR of primary liver cancer from 1990 to 2019.

Table S14. The incident cases and ASIR of primary liver cancer due to hepatitis C in 1990 and 2019 and their change trends from 1990 to 2019 at national level.

Figure S2. The global trends in the incidence of primary liver cancer due to hepatitis C in 204 countries and territories. (A) The percentage change in incident cases of primary liver cancer due to hepatitis C between 1990 and 2019; (B) The ASIR of primary liver cancer due to hepatitis C in 2019; (C) The EAPCs in ASIR of primary liver cancer due to hepatitis C from 1990 to 2019.

Table S15. The incident cases and ASIR of primary liver cancer due to alcohol use in 1990 and 2019 and their change trends from 1990 to 2019 at national level.

Figure S3. The global trends in the incidence of primary liver cancer due to alcohol use in 204 countries and territories. (A) The percentage change in incident cases of primary liver cancer due to alcohol use between 1990 and 2019; (B) The ASIR of primary liver cancer due to alcohol use in 2019; (C) The EAPCs in ASIR of primary liver cancer due to alcohol use from 1990 to 2019.

Table S16. The incident cases and ASIR of primary liver cancer due to NASH in 1990 and 2019 and their change trends from 1990 to 2019 at national level.

Figure S4. The global trends in the incidence of primary liver cancer due to NASH in 204 countries and territories. (A) The percentage change in incident cases of primary liver cancer due to NASH between 1990 and 2019; (B) The ASIR of primary liver cancer due to NASH in 2019; (C) The EAPCs in ASIR of primary liver cancer due to NASH from 1990 to 2019.

Table S17. The incident cases and ASIR of primary liver cancer due to other causes in 1990 and 2019 and their change trends from 1990 to 2019 at national level.

Figure S5. The global trends in the incidence of primary liver cancer due to other causes in 204 countries and territories. (A) The percentage change in incident cases of primary liver cancer due to other causes between 1990 and 2019; (B) The ASIR of primary liver cancer due to other causes in 2019; (C) The EAPCs in ASIR of primary liver cancer due to other causes from 1990 to 2019.

Table S18. The deaths and ASMR of primary liver cancer in 1990 and 2019 and their change trends from 1990 to 2019 at national level.

Table S19. The deaths and ASMR of primary liver cancer due to hepatitis B in 1990 and 2019 and their change trends from 1990 to 2019 at national level.

Figure S6. The global trends in the mortality of primary liver cancer due to hepatitis B in 204 countries and territories. (A) The percentage change in deaths of primary liver cancer due to hepatitis B between 1990 and 2019; (B) The ASMR of primary liver cancer due to hepatitis B in 2019; (C) The EAPCs in ASMR of primary liver cancer due to hepatitis B from 1990 to 2019.

Table S20. The deaths and ASMR of primary liver cancer due to hepatitis C in 1990 and 2019 and their change trends from 1990 to 2019 at national level.

Figure S7. The global trends in the mortality of primary liver cancer due to hepatitis C in 204 countries and territories. (A) The percentage change in deaths of primary liver cancer due to hepatitis C between 1990 and 2019; (B) The ASMR of primary liver cancer due to hepatitis C in 2019; (C) The EAPCs in ASMR of primary liver cancer due to hepatitis C from 1990 to 2019.

Table S21. The deaths and ASMR of primary liver cancer due to alcohol use in 1990 and 2019 and their change trends from 1990 to 2019 at national level.

Figure S8. The global trends in the mortality of primary liver cancer due to alcohol use in 204 countries and territories. (A) The percentage change in deaths of primary liver cancer due to alcohol use between 1990 and 2019; (B) The ASMR of primary liver cancer due to alcohol use in 2019; (C) The EAPCs in ASMR of primary liver cancer due to alcohol use from 1990 to 2019.

Table S22. The deaths and ASMR of primary liver cancer due to NASH in 1990 and 2019 and their change trends from 1990 to 2019 at national level.

Figure S9. The global trends in the mortality of primary liver cancer due to NASH in 204 countries and territories. (A) The percentage change in deaths of primary liver cancer due to NASH between 1990 and 2019; (B) The ASMR of primary liver cancer due to NASH in 2019; (C) The EAPCs in ASMR of primary liver cancer due to NASH from 1990 to 2019.

Table S23. The deaths and ASMR of primary liver cancer due to other causes in 1990 and 2019 and their change trends from 1990 to 2019 at national level.

Figure S10. The global trends in the mortality of primary liver cancer due to other causes in 204 countries and territories. (A) The percentage change in deaths of primary liver cancer due to other causes 1990 and 2019; (B) The ASMR of primary liver cancer due to other causes in 2019; (C) The EAPCs in ASMR of primary liver cancer due to other causes from 1990 to 2019.

Figure S11. The EAPCs in ASIRs and ASMRs of primary liver cancer due to hepatitis B at the national levels. (A) The correlation of EAPC in ASIRs between 1990 and 2019 with SDI in 2019. (B) The correlation of EAPC in ASIRs between 1990 and 2019 with UHCI in 2019. (C) The correlation of EAPC in ASMRs between 1990 and 2019 with SDI in 2019. (D) The correlation of EAPC in ASMRs between 1990 and 2019 with UHCI in 2019.

Figure S12. The EAPCs in ASIRs and ASMRs of primary liver cancer due to hepatitis C at the national levels. (A) The correlation of EAPC in ASIRs between 1990 and 2019 with SDI in 2019. (B) The correlation of EAPC in ASIRs between 1990 and 2019 with UHCI in 2019. (C) The correlation of EAPC in ASMRs between 1990 and 2019 with SDI in 2019. (D) The correlation of EAPC in ASMRs between 1990 and 2019 with UHCI in 2019.

Figure S13. The EAPCs in ASIRs and ASMRs of primary liver cancer due to alcohol use at the national levels. (A) The correlation of EAPC in ASIRs between 1990 and 2019 with SDI in 2019. (B) The correlation of EAPC in ASIRs between 1990 and 2019 with UHCI in 2019. (C) The correlation of EAPC in ASMRs between 1990 and 2019 with SDI in 2019. (D) The correlation of EAPC in ASMRs between 1990 and 2019 with UHCI in 2019.

Figure S14. The EAPCs in ASIRs and ASMRs of primary liver cancer due to NASH at the national levels. (A) The correlation of EAPC in ASIRs between 1990 and 2019 with SDI in 2019. (B) The correlation of EAPC in ASIRs between 1990 and 2019 with UHCI in 2019. (C) The correlation of EAPC in ASMRs between 1990 and 2019 with SDI in 2019. (D) The correlation of EAPC in ASMRs between 1990 and 2019 with UHCI in 2019.

Figure S15. The EAPCs in ASIRs and ASMRs of primary liver cancer due to other causes at the national levels. (A) The correlation of EAPC in ASIRs between 1990 and 2019 with SDI in 2019. (B) The correlation of EAPC in ASIRs between 1990 and 2019 with UHCI in 2019. (C) The correlation of EAPC in ASMRs between 1990 and 2019 with SDI in 2019. (D) The correlation of EAPC in ASMRs between 1990 and 2019 with UHCI in 2019. The incident cases and deaths of primary liver cancer due to other causes from 204 countries and territories in 2019 are represented by circles. The size of the circles increased with the incident cases and deaths. The ρ indices and p values were derived from Pearson correlation analysis.

**Table S1. The SDI value and UHCI of 204 countries and territories in 2019.**

| Region | SDI value | UHCI value |
| --- | --- | --- |
| Afghanistan | 0.343 | 39.295 |
| Albania | 0.681 | 69.626 |
| Algeria | 0.652 | 64.855 |
| American Samoa | 0.712 | 53.197 |
| Andorra | 0.894 | 91.748 |
| Angola | 0.470 | 39.158 |
| Antigua and Barbuda | 0.743 | 59.630 |
| Argentina | 0.708 | 61.158 |
| Armenia | 0.689 | 62.435 |
| Australia | 0.839 | 89.423 |
| Austria | 0.849 | 86.370 |
| Azerbaijan | 0.683 | 48.181 |
| Bahamas | 0.796 | 60.565 |
| Bahrain | 0.751 | 70.577 |
| Bangladesh | 0.483 | 53.883 |
| Barbados | 0.742 | 61.207 |
| Belarus | 0.745 | 70.462 |
| Belgium | 0.851 | 87.301 |
| Belize | 0.603 | 54.280 |
| Benin | 0.352 | 44.624 |
| Bermuda | 0.813 | 77.549 |
| Bhutan | 0.455 | 51.301 |
| Bolivia | 0.566 | 52.399 |
| Bosnia and Herzegovina | 0.718 | 64.184 |
| Botswana | 0.634 | 57.518 |
| Brazil | 0.640 | 64.828 |
| Brunei | 0.823 | 65.532 |
| Bulgaria | 0.764 | 62.557 |
| Burkina Faso | 0.257 | 41.797 |
| Burundi | 0.284 | 49.940 |
| Cambodia | 0.469 | 57.079 |
| Cameroon | 0.490 | 42.290 |
| Canada | 0.873 | 90.302 |
| Cape Verde | 0.525 | 62.188 |
| Central African Republic | 0.274 | 22.300 |
| Chad | 0.238 | 31.372 |
| Chile | 0.759 | 74.350 |
| China | 0.686 | 69.712 |
| Colombia | 0.633 | 74.397 |
| Comoros | 0.455 | 48.139 |
| Congo | 0.568 | 43.904 |
| Cook Islands | 0.764 | 62.228 |
| Costa Rica | 0.680 | 79.015 |
| Cote d'Ivoire | 0.408 | 43.040 |
| Croatia | 0.794 | 78.935 |
| Cuba | 0.668 | 72.592 |
| Cyprus | 0.841 | 79.602 |
| Czech Republic | 0.828 | 81.944 |
| Democratic Republic of the Congo | 0.382 | 45.169 |
| Denmark | 0.890 | 84.140 |
| Djibouti | 0.459 | 45.287 |
| Dominica | 0.729 | 51.809 |
| Dominican Republic | 0.592 | 52.500 |
| Ecuador | 0.640 | 64.454 |
| Egypt | 0.658 | 54.797 |
| El Salvador | 0.573 | 61.678 |
| Equatorial Guinea | 0.685 | 49.994 |
| Eritrea | 0.396 | 42.275 |
| Estonia | 0.835 | 82.039 |
| Eswatini | 0.577 | 53.397 |
| Ethiopia | 0.343 | 46.522 |
| Federated States of Micronesia | 0.580 | 34.474 |
| Fiji | 0.664 | 45.176 |
| Finland | 0.856 | 91.349 |
| France | 0.834 | 90.766 |
| Gabon | 0.656 | 53.050 |
| Gambia | 0.399 | 48.065 |
| Georgia | 0.702 | 55.953 |
| Germany | 0.898 | 86.249 |
| Ghana | 0.557 | 49.139 |
| Greece | 0.794 | 80.140 |
| Greenland | 0.761 | 68.725 |
| Grenada | 0.669 | 50.484 |
| Guam | 0.813 | 63.773 |
| Guatemala | 0.526 | 52.099 |
| Guinea | 0.325 | 32.335 |
| Guinea-Bissau | 0.355 | 35.707 |
| Guyana | 0.618 | 40.622 |
| Haiti | 0.432 | 35.812 |
| Honduras | 0.496 | 54.284 |
| Hungary | 0.791 | 72.028 |
| Iceland | 0.869 | 95.307 |
| India | 0.566 | 46.826 |
| Indonesia | 0.660 | 48.728 |
| Iran | 0.670 | 69.515 |
| Iraq | 0.671 | 57.725 |
| Ireland | 0.867 | 90.346 |
| Israel | 0.803 | 81.385 |
| Italy | 0.801 | 88.895 |
| Jamaica | 0.684 | 56.840 |
| Japan | 0.870 | 96.341 |
| Jordan | 0.731 | 69.967 |
| Kazakhstan | 0.723 | 59.237 |
| Kenya | 0.508 | 51.647 |
| Kiribati | 0.527 | 35.736 |
| Kuwait | 0.851 | 81.833 |
| Kyrgyzstan | 0.596 | 52.952 |
| Lao | 0.490 | 43.855 |
| Latvia | 0.820 | 69.788 |
| Lebanon | 0.708 | 74.533 |
| Lesotho | 0.507 | 38.737 |
| Liberia | 0.370 | 47.600 |
| Libya | 0.709 | 66.328 |
| Lithuania | 0.843 | 70.352 |
| Luxembourg | 0.895 | 91.455 |
| Macedonia | 0.744 | 60.746 |
| Madagascar | 0.396 | 39.691 |
| Malawi | 0.384 | 55.521 |
| Malaysia | 0.737 | 66.574 |
| Maldives | 0.562 | 66.857 |
| Mali | 0.263 | 40.661 |
| Malta | 0.801 | 82.881 |
| Marshall Islands | 0.544 | 44.004 |
| Mauritania | 0.496 | 53.278 |
| Mauritius | 0.705 | 55.809 |
| Mexico | 0.649 | 61.437 |
| Moldova | 0.696 | 62.194 |
| Monaco | 0.902 | 91.353 |
| Mongolia | 0.606 | 47.907 |
| Montenegro | 0.791 | 65.959 |
| Morocco | 0.548 | 58.032 |
| Mozambique | 0.307 | 44.044 |
| Myanmar | 0.521 | 46.954 |
| Namibia | 0.612 | 62.169 |
| Nauru | 0.618 | 42.002 |
| Nepal | 0.422 | 47.280 |
| Netherlands | 0.883 | 89.588 |
| New Zealand | 0.840 | 82.978 |
| Nicaragua | 0.517 | 57.159 |
| Niger | 0.162 | 35.026 |
| Nigeria | 0.515 | 38.339 |
| Niue | 0.711 | 49.010 |
| North Korea | 0.558 | 52.838 |
| Northern Mariana Islands | 0.771 | 60.411 |
| Norway | 0.913 | 94.241 |
| Oman | 0.783 | 71.221 |
| Pakistan | 0.449 | 39.168 |
| Palau | 0.738 | 45.067 |
| Palestine | 0.588 | 61.234 |
| Panama | 0.686 | 71.155 |
| Papua New Guinea | 0.394 | 37.767 |
| Paraguay | 0.638 | 63.351 |
| Peru | 0.648 | 75.759 |
| Philippines | 0.623 | 54.712 |
| Poland | 0.802 | 72.656 |
| Portugal | 0.743 | 83.533 |
| Puerto Rico | 0.814 | 75.531 |
| Qatar | 0.830 | 80.403 |
| Romania | 0.760 | 69.585 |
| Russia | 0.805 | 68.974 |
| Rwanda | 0.429 | 59.359 |
| Saint Kitts and Nevis | 0.746 | 52.885 |
| Saint Lucia | 0.670 | 59.140 |
| Saint Vincent and the Grenadines | 0.627 | 49.494 |
| Samoa | 0.641 | 49.796 |
| San Marino | 0.884 | 92.716 |
| Sao Tome and Principe | 0.502 | 54.755 |
| Saudi Arabia | 0.805 | 64.196 |
| Senegal | 0.389 | 49.610 |
| Serbia | 0.767 | 63.349 |
| Seychelles | 0.724 | 61.517 |
| Sierra Leone | 0.347 | 42.120 |
| Singapore | 0.861 | 92.440 |
| Slovakia | 0.812 | 77.983 |
| Slovenia | 0.840 | 89.834 |
| Solomon Islands | 0.407 | 39.333 |
| Somalia | 0.081 | 23.940 |
| South Africa | 0.678 | 59.727 |
| South Korea | 0.878 | 89.162 |
| South Sudan | 0.363 | 41.694 |
| Spain | 0.767 | 90.006 |
| Sri Lanka | 0.690 | 65.563 |
| Sudan | 0.515 | 51.835 |
| Suriname | 0.636 | 50.134 |
| Sweden | 0.872 | 90.361 |
| Switzerland | 0.929 | 93.498 |
| Syrian Arab Republic | 0.619 | 57.565 |
| Taiwan (Province of China) | 0.868 | 79.081 |
| Tajikistan | 0.539 | 47.878 |
| Tanzania | 0.423 | 55.249 |
| Thailand | 0.687 | 71.600 |
| Timor-Leste | 0.514 | 45.954 |
| Togo | 0.417 | 42.809 |
| Tokelau | 0.626 | 52.643 |
| Tonga | 0.636 | 52.417 |
| Trinidad and Tobago | 0.757 | 55.517 |
| Tunisia | 0.672 | 68.106 |
| Turkey | 0.748 | 69.210 |
| Turkmenistan | 0.670 | 44.013 |
| Tuvalu | 0.589 | 39.570 |
| Uganda | 0.404 | 52.748 |
| Ukraine | 0.736 | 56.752 |
| United Arab Emirates | 0.880 | 63.357 |
| United Kingdom | 0.847 | 87.900 |
| United States of America | 0.859 | 82.138 |
| United States Virgin Islands | 0.799 | 53.715 |
| Uruguay | 0.697 | 68.530 |
| Uzbekistan | 0.631 | 42.185 |
| Vanuatu | 0.485 | 34.084 |
| Venezuela | 0.607 | 60.968 |
| Viet Nam | 0.617 | 59.707 |
| Yemen | 0.412 | 49.046 |
| Zambia | 0.505 | 52.694 |
| Zimbabwe | 0.476 | 54.461 |

SDI: socio-demographic index; UHCI: universal health coverage index.

**Table S2. The incident cases and ASIR of primary liver cancer due to hepatitis B in 1990 and 2019 and their change trends from 1990 to 2019.**

| Region | 1990 | |  | 2019 | |  | 1990-2019 | |
| --- | --- | --- | --- | --- | --- | --- | --- | --- |
|  | Incident cases  No. x 10^3^ (95% UI) | ASIR per 100,000  No. (95% UI) |  | Incident cases  No. x 10^3^ (95% UI) | ASIR per 100,000  No. (95% UI) |  | Percentage change in incident cases (%) | EAPC in ASIR  No. (95% CI) |
| SDI region |  |  |  |  |  |  |  |  |
| Low | 4.01 (3.25, 4.89) | 1.51 (1.21, 1.84) |  | 7.64 (6.09, 9.36) | 1.28 (1.01, 1.57) |  | 90.40 | -0.71 (-0.81, -0.62) |
| Low-middle | 18.39 (15.70, 21.35) | 2.65 (2.25, 3.08) |  | 23.80 (20.49, 27.68) | 1.63 (1.39, 1.89) |  | 29.43 | -2.59 (-3.04, -2.14) |
| Middle | 102.25 (84.26, 121.90) | 8.58 (7.06, 10.26) |  | 110.29 (91.43, 132.42) | 4.18 (3.48, 5.01) |  | 7.86 | -3.68 (-4.36, -3.00) |
| Middle-high | 61.44 (50.81, 73.28) | 5.49 (4.55, 6.53) |  | 48.73 (39.27, 58.41) | 2.46 (1.98, 2.95) |  | -20.69 | -4.35 (-5.08, -3.62) |
| High | 11.98 (10.49, 13.76) | 1.23 (1.08, 1.40) |  | 28.34 (23.48, 33.71) | 1.71 (1.43, 2.02) |  | 136.58 | 0.91 (0.37, 1.46) |
| GBD region |  |  |  |  |  |  |  |  |
| High-income Asia Pacific | 6.23 (5.47, 7.07) | 3.00 (2.63, 3.40) |  | 16.84 (13.56, 20.45) | 4.73 (3.84, 5.69) |  | 170.22 | 1.59 (0.86, 2.33) |
| Central Asia | 0.44 (0.33, 0.61) | 0.90 (0.65, 1.22) |  | 1.70 (1.21, 2.27) | 2.05 (1.47, 2.78) |  | 282.60 | 2.14 (1.56, 2.71) |
| East Asia | 161.26 (132.17, 194.34) | 16.16 (13.32, 19.41) |  | 138.51 (112.43, 167.40) | 6.57 (5.35, 7.95) |  | -14.11 | -4.69 (-5.51, -3.87) |
| South Asia | 5.98 (4.87, 7.12) | 0.92 (0.73, 1.10) |  | 12.56 (10.25, 15.24) | 0.84 (0.68, 1.02) |  | 109.96 | -0.25 (-0.37, -0.12) |
| Southeast Asia | 7.99 (6.57, 9.56) | 2.73 (2.19, 3.29) |  | 17.14 (12.89, 22.48) | 2.64 (1.97, 3.45) |  | 114.41 | -0.25 (-0.31, -0.18) |
| Australasia | 0.08 (0.06, 0.10) | 0.34 (0.25, 0.44) |  | 0.30 (0.20, 0.42) | 0.68 (0.47, 0.97) |  | 282.73 | 2.65 (2.36, 2.95) |
| Caribbean | 0.45 (0.34, 0.59) | 1.68 (1.25, 2.22) |  | 0.44 (0.31, 0.62) | 0.86 (0.60, 1.20) |  | -2.09 | -2.26 (-3.08, -1.44) |
| Central Europe | 1.85 (1.45, 2.31) | 1.25 (0.99, 1.57) |  | 1.37 (0.98, 1.91) | 0.70 (0.51, 0.96) |  | -25.65 | -1.83 (-2.12, -1.55) |
| Eastern Europe | 1.10 (0.93, 1.29) | 0.40 (0.34, 0.46) |  | 2.16 (1.72, 2.69) | 0.68 (0.55, 0.85) |  | 97.34 | 2.16 (1.87, 2.45) |
| Western Europe | 2.64 (2.02, 3.41) | 0.50 (0.38, 0.63) |  | 5.34 (3.87, 7.41) | 0.71 (0.52, 0.97) |  | 102.12 | 1.14 (0.98, 1.31) |
| Andean Latin America | 0.50 (0.40, 0.62) | 2.29 (1.80, 2.85) |  | 0.74 (0.54, 0.97) | 1.31 (0.95, 1.71) |  | 47.63 | -2.42 (-2.82, -2.01) |
| Central Latin America | 0.55 (0.42, 0.71) | 0.58 (0.43, 0.77) |  | 1.17 (0.86, 1.61) | 0.48 (0.36, 0.67) |  | 111.55 | -0.62 (-0.97, -0.27) |
| Southern Latin America | 0.14 (0.10, 0.19) | 0.29 (0.21, 0.41) |  | 0.33 (0.21, 0.49) | 0.40 (0.26, 0.61) |  | 139.04 | 1.68 (1.49, 1.87) |
| Tropical Latin America | 0.42 (0.37, 0.48) | 0.41 (0.35, 0.47) |  | 1.03 (0.87, 1.21) | 0.42 (0.35, 0.49) |  | 143.32 | 0.35 (0.23, 0.47) |
| North Africa and Middle East | 3.20 (2.54, 3.97) | 1.69 (1.32, 2.13) |  | 7.26 (5.42, 9.47) | 1.53 (1.14, 2.01) |  | 126.64 | -0.34 (-0.47, -0.21) |
| High-income North America | 1.02 (0.88, 1.18) | 0.31 (0.27, 0.36) |  | 3.66 (2.79, 4.74) | 0.66 (0.50, 0.85) |  | 258.34 | 2.68 (2.42, 2.94) |
| Oceania | 0.06 (0.05, 0.08) | 1.88 (1.45, 2.35) |  | 0.13 (0.10, 0.16) | 1.59 (1.22, 2.04) |  | 96.62 | -0.42 (-0.49, -0.35) |
| Central Sub-Saharan Africa | 0.19 (0.13, 0.26) | 0.66 (0.46, 0.91) |  | 0.38 (0.26, 0.54) | 0.53 (0.36, 0.78) |  | 99.20 | -0.85 (-0.97, -0.73) |
| Eastern Sub-Saharan Africa | 0.73 (0.55, 1.00) | 0.83 (0.61, 1.15) |  | 1.65 (1.20, 2.20) | 0.83 (0.61, 1.13) |  | 124.51 | -0.29 (-0.46, -0.13) |
| Southern Sub-Saharan Africa | 0.78 (0.52, 1.35) | 2.39 (1.56, 4.14) |  | 1.56 (1.30, 1.87) | 2.39 (1.98, 2.85) |  | 100.83 | -0.72 (-1.33, -0.12) |
| Western Sub-Saharan Africa | 2.49 (1.98, 3.05) | 2.57 (2.03, 3.15) |  | 4.61 (3.60, 5.78) | 2.17 (1.67, 2.72) |  | 85.38 | -0.72 (-0.82, -0.62) |

ASIR: age-standardized incidence rate; CI: confidence interval; GBD: Global Burden of Disease; SDI: socio-demographic index; UI: uncertainty interval.

**Table S3. The incident cases and ASIR of primary liver cancer due to hepatitis C in 1990 and 2019 and their change trends from 1990 to 2019.**

| Region | 1990 | |  | 2019 | |  | 1990-2019 | |
| --- | --- | --- | --- | --- | --- | --- | --- | --- |
|  | Incident cases  No. x 10^3^ (95% UI) | ASIR per 100,000  No. (95% UI) |  | Incident cases  No. x 10^3^ (95% UI) | ASIR per 100,000  No. (95% UI) |  | Percentage change in incident cases (%) | EAPC in ASIR  No. (95% CI) |
| SDI region |  |  |  |  |  |  |  |  |
| Low | 2.52 (1.92, 3.14) | 1.19 (0.91, 1.47) |  | 5.01 (3.88, 6.12) | 1.06 (0.83, 1.28) |  | 98.49 | -0.51 (-0.57, -0.45) |
| Low-middle | 6.46 (5.22, 7.84) | 1.20 (0.99, 1.45) |  | 13.23 (10.65, 15.82) | 1.03 (0.84, 1.23) |  | 104.74 | -0.83 (-1.00, -0.65) |
| Middle | 29.05 (24.23, 34.40) | 3.03 (2.56, 3.58) |  | 44.75 (36.83, 54.10) | 1.87 (1.56, 2.25) |  | 54.03 | -2.34 (-2.78, -1.90) |
| Middle-high | 21.95 (18.62, 25.39) | 2.11 (1.80, 2.42) |  | 27.23 (23.25, 31.62) | 1.34 (1.14, 1.55) |  | 24.04 | -2.36 (-2.73, -2.00) |
| High | 24.23 (22.07, 26.43) | 2.35 (2.14, 2.57) |  | 61.95 (52.88, 71.34) | 3.13 (2.69, 3.60) |  | 155.65 | 0.45 (-0.05, 0.94) |
| GBD region |  |  |  |  |  |  |  |  |
| High-income Asia Pacific | 16.30 (15.27, 17.27) | 8.00 (7.50, 8.47) |  | 37.12 (30.23, 43.42) | 7.54 (6.29, 8.91) |  | 127.72 | -1.01 (-1.57, -0.45) |
| Central Asia | 0.47 (0.33, 0.61) | 1.04 (0.74, 1.34) |  | 1.89 (1.33, 2.49) | 2.81 (2.04, 3.63) |  | 306.44 | 3.14 (2.57, 3.71) |
| East Asia | 36.42 (29.96, 44.39) | 4.48 (3.72, 5.41) |  | 35.91 (29.63, 42.90) | 1.79 (1.48, 2.12) |  | -1.41 | -4.59 (-5.29, -3.89) |
| South Asia | 3.55 (2.75, 4.41) | 0.73 (0.56, 0.90) |  | 9.25 (7.52, 11.10) | 0.70 (0.58, 0.84) |  | 160.23 | -0.21 (-0.29, -0.13) |
| Southeast Asia | 3.69 (2.80, 4.65) | 1.60 (1.24, 1.99) |  | 9.15 (6.79, 12.02) | 1.67 (1.25, 2.17) |  | 147.91 | 0.16 (0.12, 0.21) |
| Australasia | 0.13 (0.10, 0.17) | 0.57 (0.44, 0.72) |  | 0.69 (0.49, 0.95) | 1.39 (0.99, 1.91) |  | 414.06 | 3.39 (3.17, 3.61) |
| Caribbean | 0.36 (0.25, 0.47) | 1.40 (0.99, 1.86) |  | 0.35 (0.24, 0.50) | 0.68 (0.46, 0.96) |  | -0.82 | -2.41 (-3.21, -1.61) |
| Central Europe | 2.01 (1.59, 2.48) | 1.40 (1.12, 1.71) |  | 1.78 (1.30, 2.39) | 0.81 (0.59, 1.08) |  | -11.50 | -1.60 (-1.99, -1.21) |
| Eastern Europe | 1.08 (0.92, 1.23) | 0.39 (0.34, 0.44) |  | 2.51 (2.07, 3.01) | 0.71 (0.59, 0.85) |  | 133.15 | 2.53 (2.30, 2.76) |
| Western Europe | 8.40 (7.13, 9.78) | 1.43 (1.22, 1.67) |  | 18.95 (15.19, 23.28) | 2.05 (1.63, 2.55) |  | 125.53 | 1.16 (0.97, 1.34) |
| Andean Latin America | 0.07 (0.04, 0.10) | 0.36 (0.22, 0.53) |  | 0.13 (0.08, 0.20) | 0.25 (0.15, 0.38) |  | 96.23 | -1.76 (-2.18, -1.34) |
| Central Latin America | 1.10 (0.90, 1.31) | 1.41 (1.16, 1.68) |  | 3.10 (2.49, 3.79) | 1.35 (1.08, 1.65) |  | 181.09 | 0.01 (-0.29, 0.30) |
| Southern Latin America | 0.24 (0.17, 0.31) | 0.52 (0.39, 0.68) |  | 0.68 (0.46, 0.93) | 0.80 (0.55, 1.10) |  | 186.64 | 2.17 (1.93, 2.41) |
| Tropical Latin America | 0.66 (0.58, 0.73) | 0.78 (0.69, 0.87) |  | 2.22 (1.94, 2.49) | 0.94 (0.82, 1.05) |  | 237.08 | 1.14 (0.93, 1.35) |
| North Africa and Middle East | 4.93 (3.89, 6.01) | 2.98 (2.37, 3.63) |  | 12.95 (9.36, 17.37) | 3.08 (2.28, 4.07) |  | 162.52 | 0.71 (0.47, 0.96) |
| High-income North America | 2.60 (2.33, 2.85) | 0.74 (0.67, 0.82) |  | 10.99 (8.87, 13.36) | 1.77 (1.43, 2.15) |  | 322.48 | 3.09 (2.82, 3.36) |
| Oceania | 0.02 (0.01, 0.03) | 0.88 (0.60, 1.19) |  | 0.05 (0.03, 0.07) | 0.80 (0.55, 1.10) |  | 111.21 | -0.18 (-0.25, -0.12) |
| Central Sub-Saharan Africa | 0.26 (0.19, 0.34) | 1.27 (0.97, 1.60) |  | 0.54 (0.38, 0.72) | 1.12 (0.82, 1.47) |  | 107.37 | -0.59 (-0.66, -0.51) |
| Eastern Sub-Saharan Africa | 0.52 (0.36, 0.69) | 0.77 (0.56, 1.02) |  | 1.17 (0.86, 1.50) | 0.83 (0.63, 1.06) |  | 126.40 | 0.06 (-0.03, 0.15) |
| Southern Sub-Saharan Africa | 0.48 (0.33, 0.72) | 1.86 (1.27, 2.79) |  | 1.04 (0.84, 1.25) | 1.96 (1.62, 2.35) |  | 115.67 | -0.24 (-0.73, 0.25) |
| Western Sub-Saharan Africa | 0.96 (0.68, 1.27) | 1.20 (0.86, 1.56) |  | 1.77 (1.27, 2.30) | 1.07 (0.78, 1.37) |  | 84.15 | -0.49 (-0.53, -0.45) |

ASIR: age-standardized incidence rate; CI: confidence interval; GBD: Global Burden of Disease; SDI: socio-demographic index; UI: uncertainty interval.

**Table S4. The incident cases and ASIR of primary liver cancer due to alcohol use in 1990 and 2019 and their change trends from 1990 to 2019.**

| Region | 1990 | |  | 2019 | |  | 1990-2019 | |
| --- | --- | --- | --- | --- | --- | --- | --- | --- |
|  | Incident cases  No. x 10^3^ (95% UI) | ASIR per 100,000  No. (95% UI) |  | Incident cases  No. x 10^3^ (95% UI) | ASIR per 100,000  No. (95% UI) |  | Percentage change in incident cases (%) | EAPC in ASIR  No. (95% CI) |
| SDI region |  |  |  |  |  |  |  |  |
| Low | 1.87 (1.39, 2.45) | 0.80 (0.60, 1.05) |  | 4.01 (2.99, 5.17) | 0.78 (0.59, 1.00) |  | 114.56 | -0.16 (-0.22, -0.10) |
| Low-middle | 4.99 (3.88, 6.26) | 0.83 (0.65, 1.04) |  | 11.18 (8.84, 13.85) | 0.82 (0.65, 1.01) |  | 124.20 | -0.32 (-0.50, -0.14) |
| Middle | 15.71 (11.86, 20.23) | 1.47 (1.13, 1.89) |  | 30.25 (23.06, 38.46) | 1.19 (0.91, 1.51) |  | 92.58 | -1.29 (-1.70, -0.89) |
| Middle-high | 14.00 (11.27, 17.04) | 1.28 (1.04, 1.56) |  | 19.21 (15.44, 23.37) | 0.93 (0.75, 1.13) |  | 37.22 | -1.64 (-1.92, -1.37) |
| High | 11.74 (9.85, 13.65) | 1.14 (0.96, 1.32) |  | 33.75 (27.37, 40.57) | 1.86 (1.51, 2.24) |  | 187.41 | 1.54 (1.27, 1.81) |
| GBD region |  |  |  |  |  |  |  |  |
| High-income Asia Pacific | 3.66 (3.04, 4.34) | 1.76 (1.47, 2.08) |  | 8.72 (6.69, 11.07) | 2.07 (1.58, 2.67) |  | 138.14 | -0.07 (-0.54, 0.40) |
| Central Asia | 0.39 (0.27, 0.52) | 0.85 (0.59, 1.12) |  | 1.83 (1.29, 2.49) | 2.47 (1.77, 3.26) |  | 365.58 | 2.80 (2.24, 3.37) |
| East Asia | 17.94 (13.02, 23.87) | 1.92 (1.42, 2.55) |  | 19.77 (14.72, 26.28) | 0.92 (0.69, 1.21) |  | 10.16 | -4.04 (-4.87, -3.20) |
| South Asia | 3.51 (2.73, 4.39) | 0.63 (0.48, 0.78) |  | 9.74 (7.74, 12.00) | 0.69 (0.55, 0.85) |  | 177.35 | 0.32 (0.25, 0.38) |
| Southeast Asia | 3.55 (2.61, 4.68) | 1.37 (1.02, 1.81) |  | 11.13 (7.77, 15.45) | 1.84 (1.30, 2.54) |  | 213.56 | 1.12 (1.03, 1.20) |
| Australasia | 0.20 (0.16, 0.24) | 0.86 (0.68, 1.02) |  | 0.84 (0.62, 1.13) | 1.79 (1.31, 2.42) |  | 317.96 | 2.98 (2.69, 3.27) |
| Caribbean | 0.51 (0.37, 0.65) | 1.94 (1.43, 2.49) |  | 0.57 (0.41, 0.77) | 1.10 (0.79, 1.49) |  | 12.17 | -1.85 (-2.64, -1.05) |
| Central Europe | 3.01 (2.49, 3.50) | 2.02 (1.67, 2.34) |  | 2.99 (2.32, 3.70) | 1.40 (1.09, 1.74) |  | -0.87 | -0.89 (-1.28, -0.51) |
| Eastern Europe | 1.37 (1.19, 1.57) | 0.48 (0.42, 0.55) |  | 3.55 (2.92, 4.24) | 1.04 (0.86, 1.24) |  | 158.70 | 2.98 (2.66, 3.31) |
| Western Europe | 7.13 (5.80, 8.39) | 1.25 (1.02, 1.47) |  | 16.56 (12.86, 20.52) | 1.94 (1.50, 2.40) |  | 132.28 | 1.57 (1.37, 1.77) |
| Andean Latin America | 0.29 (0.21, 0.39) | 1.49 (1.05, 1.99) |  | 0.55 (0.38, 0.76) | 1.00 (0.69, 1.38) |  | 86.46 | -1.76 (-2.29, -1.23) |
| Central Latin America | 0.83 (0.64, 1.02) | 1.00 (0.77, 1.23) |  | 2.47 (1.91, 3.11) | 1.06 (0.82, 1.33) |  | 198.76 | 0.41 (0.04, 0.79) |
| Southern Latin America | 0.24 (0.17, 0.31) | 0.51 (0.37, 0.66) |  | 0.63 (0.42, 0.87) | 0.75 (0.50, 1.04) |  | 163.01 | 2.03 (1.71, 2.35) |
| Tropical Latin America | 0.50 (0.43, 0.57) | 0.54 (0.47, 0.62) |  | 1.75 (1.51, 1.99) | 0.72 (0.62, 0.82) |  | 248.74 | 1.41 (1.25, 1.57) |
| North Africa and Middle East | 0.94 (0.62, 1.34) | 0.55 (0.37, 0.78) |  | 2.62 (1.68, 4.01) | 0.61 (0.40, 0.91) |  | 177.50 | 0.71 (0.51, 0.90) |
| High-income North America | 2.39 (2.12, 2.65) | 0.69 (0.62, 0.77) |  | 10.67 (8.40, 13.36) | 1.77 (1.39, 2.22) |  | 345.98 | 3.17 (3.05, 3.29) |
| Oceania | 0.01 (0.01, 0.02) | 0.44 (0.28, 0.65) |  | 0.03 (0.02, 0.04) | 0.44 (0.28, 0.64) |  | 128.74 | 0.20 (0.13, 0.26) |
| Central Sub-Saharan Africa | 0.08 (0.05, 0.12) | 0.36 (0.24, 0.51) |  | 0.18 (0.11, 0.27) | 0.33 (0.21, 0.48) |  | 119.02 | -0.41 (-0.48, -0.34) |
| Eastern Sub-Saharan Africa | 0.57 (0.40, 0.81) | 0.76 (0.55, 1.09) |  | 1.32 (0.93, 1.85) | 0.83 (0.58, 1.14) |  | 133.11 | 0.04 (-0.11, 0.19) |
| Southern Sub-Saharan Africa | 0.36 (0.21, 0.69) | 1.26 (0.75, 2.41) |  | 0.78 (0.62, 0.99) | 1.34 (1.07, 1.67) |  | 118.61 | -0.49 (-1.12, 0.14) |
| Western Sub-Saharan Africa | 0.84 (0.59, 1.13) | 0.96 (0.69, 1.30) |  | 1.77 (1.28, 2.34) | 0.98 (0.71, 1.29) |  | 110.55 | -0.08 (-0.18, 0.02) |

ASIR: age-standardized incidence rate; CI: confidence interval; GBD: Global Burden of Disease; SDI: socio-demographic index; UI: uncertainty interval.

**Table S5. The incident cases and ASIR of primary liver cancer due to NASH in 1990 and 2019 and their change trends from 1990 to 2019.**

| Region | 1990 | |  | 2019 | |  | 1990-2019 | |
| --- | --- | --- | --- | --- | --- | --- | --- | --- |
|  | Incident cases  No. x 10^3^ (95% UI) | ASIR per 100,000  No. (95% UI) |  | Incident cases  No. x 10^3^ (95% UI) | ASIR per 100,000  No. (95% UI) |  | Percentage change in incident cases (%) | EAPC in ASIR  No. (95% CI) |
| SDI region |  |  |  |  |  |  |  |  |
| Low | 0.75 (0.58, 0.98) | 0.33 (0.25, 0.43) |  | 1.75 (1.34, 2.27) | 0.34 (0.26, 0.45) |  | 131.95 | 0.02 (-0.02, 0.07) |
| Low-middle | 2.09 (1.71, 2.56) | 0.35 (0.29, 0.43) |  | 4.64 (3.75, 5.74) | 0.35 (0.28, 0.43) |  | 121.97 | -0.42 (-0.63, -0.21) |
| Middle | 7.32 (5.88, 8.84) | 0.70 (0.56, 0.85) |  | 13.92 (11.07, 17.47) | 0.57 (0.46, 0.72) |  | 90.05 | -1.41 (-1.89, -0.93) |
| Middle-high | 4.63 (3.79, 5.57) | 0.43 (0.35, 0.52) |  | 6.36 (5.19, 7.87) | 0.32 (0.26, 0.39) |  | 37.50 | -1.99 (-2.46, -1.52) |
| High | 2.89 (2.37, 3.54) | 0.28 (0.23, 0.34) |  | 9.65 (7.60, 12.30) | 0.51 (0.41, 0.64) |  | 233.54 | 1.89 (1.48, 2.29) |
| GBD region |  |  |  |  |  |  |  |  |
| High-income Asia Pacific | 1.13 (0.94, 1.33) | 0.56 (0.47, 0.66) |  | 3.36 (2.56, 4.36) | 0.71 (0.55, 0.92) |  | 197.49 | 0.30 (-0.28, 0.89) |
| Central Asia | 0.08 (0.05, 0.11) | 0.17 (0.12, 0.25) |  | 0.42 (0.29, 0.59) | 0.61 (0.43, 0.87) |  | 439.92 | 4.01 (3.48, 4.55) |
| East Asia | 9.02 (7.07, 11.09) | 1.00 (0.79, 1.22) |  | 10.46 (8.18, 12.96) | 0.51 (0.40, 0.63) |  | 15.93 | -3.79 (-4.59, -2.98) |
| South Asia | 1.38 (1.11, 1.69) | 0.25 (0.20, 0.31) |  | 3.97 (3.23, 4.91) | 0.29 (0.24, 0.36) |  | 187.09 | 0.41 (0.32, 0.49) |
| Southeast Asia | 1.21 (0.91, 1.60) | 0.49 (0.36, 0.64) |  | 3.97 (2.82, 5.48) | 0.70 (0.49, 0.97) |  | 228.14 | 1.33 (1.25, 1.42) |
| Australasia | 0.04 (0.03, 0.05) | 0.15 (0.11, 0.20) |  | 0.23 (0.16, 0.33) | 0.47 (0.32, 0.67) |  | 545.75 | 4.24 (3.90, 4.59) |
| Caribbean | 0.13 (0.09, 0.18) | 0.50 (0.35, 0.68) |  | 0.15 (0.11, 0.22) | 0.30 (0.20, 0.42) |  | 19.55 | -1.59 (-2.43, -0.75) |
| Central Europe | 0.55 (0.43, 0.69) | 0.38 (0.30, 0.48) |  | 0.55 (0.40, 0.77) | 0.26 (0.19, 0.35) |  | 1.48 | -1.05 (-1.48, -0.62) |
| Eastern Europe | 0.29 (0.24, 0.34) | 0.10 (0.09, 0.12) |  | 0.77 (0.63, 0.93) | 0.23 (0.19, 0.27) |  | 169.45 | 3.16 (2.92, 3.40) |
| Western Europe | 1.10 (0.84, 1.45) | 0.19 (0.14, 0.24) |  | 3.01 (2.18, 4.14) | 0.33 (0.24, 0.45) |  | 174.55 | 2.02 (1.79, 2.24) |
| Andean Latin America | 0.08 (0.05, 0.11) | 0.39 (0.27, 0.55) |  | 0.18 (0.12, 0.25) | 0.33 (0.22, 0.46) |  | 129.39 | -1.15 (-1.57, -0.73) |
| Central Latin America | 0.21 (0.16, 0.27) | 0.25 (0.19, 0.33) |  | 0.73 (0.57, 0.96) | 0.32 (0.24, 0.41) |  | 250.67 | 1.02 (0.71, 1.33) |
| Southern Latin America | 0.05 (0.04, 0.08) | 0.12 (0.08, 0.17) |  | 0.19 (0.12, 0.27) | 0.22 (0.15, 0.32) |  | 248.66 | 2.90 (2.67, 3.13) |
| Tropical Latin America | 0.10 (0.09, 0.12) | 0.11 (0.09, 0.13) |  | 0.36 (0.30, 0.42) | 0.15 (0.13, 0.18) |  | 256.81 | 1.54 (1.32, 1.77) |
| North Africa and Middle East | 0.81 (0.59, 1.14) | 0.48 (0.35, 0.68) |  | 2.87 (2.00, 4.13) | 0.68 (0.48, 0.97) |  | 253.90 | 1.46 (1.27, 1.65) |
| High-income North America | 0.73 (0.62, 0.85) | 0.20 (0.18, 0.24) |  | 3.18 (2.50, 3.94) | 0.52 (0.41, 0.64) |  | 337.25 | 3.37 (3.12, 3.61) |
| Oceania | 0.01 (0.01, 0.01) | 0.28 (0.19, 0.41) |  | 0.02 (0.01, 0.03) | 0.30 (0.21, 0.43) |  | 144.93 | 0.27 (0.20, 0.34) |
| Central Sub-Saharan Africa | 0.04 (0.03, 0.05) | 0.16 (0.11, 0.23) |  | 0.09 (0.06, 0.13) | 0.16 (0.11, 0.24) |  | 143.26 | -0.17 (-0.25, -0.09) |
| Eastern Sub-Saharan Africa | 0.23 (0.17, 0.32) | 0.32 (0.24, 0.44) |  | 0.60 (0.44, 0.81) | 0.38 (0.28, 0.52) |  | 156.94 | 0.39 (0.28, 0.50) |
| Southern Sub-Saharan Africa | 0.17 (0.12, 0.28) | 0.61 (0.40, 1.00) |  | 0.42 (0.34, 0.51) | 0.75 (0.61, 0.92) |  | 143.03 | 0.20 (-0.37, 0.77) |
| Western Sub-Saharan Africa | 0.35 (0.26, 0.47) | 0.41 (0.30, 0.55) |  | 0.80 (0.59, 1.07) | 0.45 (0.33, 0.61) |  | 127.88 | 0.16 (0.08, 0.24) |

ASIR: age-standardized incidence rate; CI: confidence interval; GBD: Global Burden of Disease; NASH: nonalcoholic steatohepatitis; SDI: socio-demographic index; UI: uncertainty interval.

**Table S6. The incident cases and ASIR of primary liver cancer due to other causes in 1990 and 2019 and their change trends from 1990 to 2019.**

| Region | 1990 | |  | 2019 | |  | 1990-2019 | |
| --- | --- | --- | --- | --- | --- | --- | --- | --- |
|  | Incident cases  No. x 10^3^ (95% UI) | ASIR per 100,000  No. (95% UI) |  | Incident cases  No. x 10^3^ (95% UI) | ASIR per 100,000  No. (95% UI) |  | Percentage change in incident cases (%) | EAPC in ASIR  No. (95% CI) |
| SDI region |  |  |  |  |  |  |  |  |
| Low | 1.09 (0.83, 1.40) | 0.25 (0.19, 0.32) |  | 1.88 (1.47, 2.37) | 0.23 (0.18, 0.28) |  | 72.80 | -0.40 (-0.43, -0.37) |
| Low-middle | 2.79 (2.32, 3.36) | 0.33 (0.27, 0.39) |  | 3.45 (2.83, 4.14) | 0.23 (0.19, 0.28) |  | 23.72 | -1.99 (-2.35, -1.61) |
| Middle | 11.75 (9.74, 14.38) | 0.94 (0.77, 1.16) |  | 11.34 (9.26, 13.87) | 0.45 (0.37, 0.55) |  | -3.53 | -3.57 (-4.21, -2.93) |
| Middle-high | 6.72 (5.58, 8.04) | 0.61 (0.50, 0.73) |  | 5.35 (4.39, 6.46) | 0.30 (0.25, 0.35) |  | -20.27 | -3.71 (-4.31, -3.10) |
| High | 2.64 (2.21, 3.11) | 0.28 (0.24, 0.33) |  | 6.45 (5.13, 7.96) | 0.41 (0.33, 0.49) |  | 143.81 | 1.04 (0.72, 1.35) |
| GBD region |  |  |  |  |  |  |  |  |
| High-income Asia Pacific | 0.89 (0.75, 1.04) | 0.45 (0.38, 0.52) |  | 1.91 (1.46, 2.40) | 0.50 (0.39, 0.62) |  | 114.60 | -0.22 (-0.72, 0.29) |
| Central Asia | 0.11 (0.08, 0.16) | 0.17 (0.12, 0.24) |  | 0.26 (0.19, 0.37) | 0.33 (0.24, 0.46) |  | 136.80 | 2.01 (1.66, 2.36) |
| East Asia | 16.89 (13.81, 20.86) | 1.69 (1.38, 2.09) |  | 12.53 (10.14, 15.48) | 0.64 (0.52, 0.78) |  | -25.81 | -4.84 (-5.64, -4.04) |
| South Asia | 1.25 (1.04, 1.52) | 0.13 (0.11, 0.16) |  | 2.22 (1.85, 2.75) | 0.14 (0.12, 0.17) |  | 77.48 | -0.01 (-0.06, 0.05) |
| Southeast Asia | 0.86 (0.66, 1.13) | 0.24 (0.18, 0.30) |  | 1.41 (1.05, 1.91) | 0.23 (0.17, 0.31) |  | 63.37 | -0.21 (-0.25, -0.17) |
| Australasia | 0.03 (0.02, 0.03) | 0.13 (0.10, 0.16) |  | 0.10 (0.07, 0.14) | 0.25 (0.18, 0.34) |  | 277.25 | 2.65 (2.43, 2.87) |
| Caribbean | 0.12 (0.09, 0.15) | 0.41 (0.30, 0.55) |  | 0.11 (0.08, 0.15) | 0.22 (0.16, 0.30) |  | -7.98 | -2.09 (-2.80, -1.37) |
| Central Europe | 0.29 (0.23, 0.36) | 0.21 (0.17, 0.26) |  | 0.22 (0.15, 0.30) | 0.13 (0.09, 0.17) |  | -24.63 | -1.42 (-1.75, -1.08) |
| Eastern Europe | 0.31 (0.26, 0.38) | 0.14 (0.12, 0.18) |  | 0.41 (0.32, 0.53) | 0.17 (0.12, 0.25) |  | 33.94 | 0.93 (0.73, 1.13) |
| Western Europe | 0.92 (0.72, 1.16) | 0.19 (0.15, 0.23) |  | 2.01 (1.46, 2.69) | 0.28 (0.22, 0.37) |  | 118.85 | 1.48 (1.31, 1.65) |
| Andean Latin America | 0.09 (0.07, 0.12) | 0.37 (0.26, 0.50) |  | 0.14 (0.09, 0.18) | 0.24 (0.16, 0.32) |  | 42.32 | -1.94 (-2.32, -1.55) |
| Central Latin America | 0.28 (0.24, 0.33) | 0.24 (0.19, 0.29) |  | 0.52 (0.41, 0.65) | 0.22 (0.17, 0.27) |  | 84.79 | -0.08 (-0.35, 0.20) |
| Southern Latin America | 0.05 (0.04, 0.07) | 0.11 (0.09, 0.15) |  | 0.12 (0.08, 0.17) | 0.15 (0.10, 0.21) |  | 113.05 | 1.54 (1.34, 1.73) |
| Tropical Latin America | 0.16 (0.15, 0.19) | 0.13 (0.12, 0.15) |  | 0.31 (0.27, 0.36) | 0.14 (0.12, 0.16) |  | 91.04 | 0.48 (0.36, 0.60) |
| North Africa and Middle East | 0.84 (0.64, 1.08) | 0.37 (0.28, 0.48) |  | 1.85 (1.32, 2.57) | 0.38 (0.27, 0.54) |  | 118.96 | 0.51 (0.32, 0.69) |
| High-income North America | 0.79 (0.70, 0.88) | 0.25 (0.22, 0.28) |  | 2.51 (2.00, 3.10) | 0.47 (0.38, 0.57) |  | 217.17 | 2.12 (1.93, 2.32) |
| Oceania | 0.01 (0.01, 0.01) | 0.17 (0.12, 0.23) |  | 0.01 (0.01, 0.02) | 0.15 (0.10, 0.21) |  | 94.67 | -0.29 (-0.34, -0.24) |
| Central Sub-Saharan Africa | 0.12 (0.07, 0.19) | 0.18 (0.13, 0.25) |  | 0.18 (0.11, 0.29) | 0.15 (0.10, 0.22) |  | 50.66 | -0.72 (-0.76, -0.67) |
| Eastern Sub-Saharan Africa | 0.40 (0.27, 0.57) | 0.25 (0.18, 0.34) |  | 0.71 (0.52, 0.94) | 0.25 (0.19, 0.32) |  | 78.83 | -0.16 (-0.25, -0.08) |
| Southern Sub-Saharan Africa | 0.12 (0.09, 0.18) | 0.34 (0.24, 0.53) |  | 0.22 (0.18, 0.27) | 0.34 (0.27, 0.42) |  | 80.91 | -0.41 (-0.92, 0.10) |
| Western Sub-Saharan Africa | 0.47 (0.36, 0.60) | 0.30 (0.23, 0.38) |  | 0.75 (0.56, 0.98) | 0.26 (0.20, 0.34) |  | 59.80 | -0.53 (-0.65, -0.40) |

ASIR: age-standardized incidence rate; CI: confidence interval; GBD: Global Burden of Disease; SDI: socio-demographic index; UI: uncertainty interval.

**Table S7. The deaths and ASMR of primary liver cancer due to hepatitis B in 1990 and 2019 and their change trends from 1990 to 2019.**

| Region | 1990 | |  | 2019 | |  | 1990-2019 | |
| --- | --- | --- | --- | --- | --- | --- | --- | --- |
|  | Deaths  No. x 10^3^ (95% UI) | ASMR per 100,000  No. (95% UI) |  | Deaths  No. x 10^3^ (95% UI) | ASMR per 100,000  No. (95% UI) |  | Percentage change in deaths (%) | EAPC in ASMR  No. (95% CI) |
| SDI region |  |  |  |  |  |  |  |  |
| Low | 3.94 (3.18, 4.81) | 1.55 (1.24, 1.90) |  | 7.46 (5.92, 9.16) | 1.30 (1.03, 1.62) |  | 89.19 | -0.73 (-0.84, -0.63) |
| Low-middle | 17.80 (15.27, 20.62) | 2.64 (2.27, 3.04) |  | 23.19 (19.84, 26.87) | 1.62 (1.38, 1.88) |  | 30.26 | -2.59 (-3.04, -2.14) |
| Middle | 98.51 (82.69, 118.01) | 8.47 (7.11, 10.09) |  | 98.32 (81.73, 117.89) | 3.79 (3.15, 4.54) |  | -0.19 | -3.95 (-4.63, -3.28) |
| Middle-high | 59.35 (49.16, 70.50) | 5.34 (4.44, 6.33) |  | 41.65 (34.36, 50.27) | 2.08 (1.72, 2.51) |  | -29.83 | -4.85 (-5.58, -4.11) |
| High | 10.65 (9.20, 12.34) | 1.08 (0.94, 1.25) |  | 21.06 (17.52, 24.91) | 1.22 (1.03, 1.44) |  | 97.81 | 0.13 (-0.41, 0.67) |
| GBD region |  |  |  |  |  |  |  |  |
| High-income Asia Pacific | 5.27 (4.59, 6.03) | 2.54 (2.21, 2.90) |  | 11.62 (9.71, 13.64) | 3.10 (2.62, 3.60) |  | 120.41 | 0.62 (-0.18, 1.44) |
| Central Asia | 0.43 (0.32, 0.59) | 0.89 (0.64, 1.22) |  | 1.65 (1.18, 2.21) | 2.05 (1.47, 2.81) |  | 282.81 | 2.25 (1.68, 2.83) |
| East Asia | 155.31 (128.85, 186.54) | 15.90 (13.23, 19.08) |  | 120.12 (97.66, 146.17) | 5.71 (4.68, 6.92) |  | -22.66 | -5.11 (-5.92, -4.29) |
| South Asia | 5.79 (4.76, 6.88) | 0.93 (0.74, 1.11) |  | 12.36 (10.14, 14.96) | 0.84 (0.69, 1.03) |  | 113.57 | -0.28 (-0.42, -0.14) |
| Southeast Asia | 7.78 (6.34, 9.33) | 2.75 (2.20, 3.33) |  | 16.44 (12.18, 21.72) | 2.60 (1.93, 3.43) |  | 111.29 | -0.29 (-0.38, -0.21) |
| Australasia | 0.07 (0.05, 0.10) | 0.31 (0.23, 0.41) |  | 0.25 (0.18, 0.35) | 0.56 (0.40, 0.77) |  | 256.37 | 2.29 (2.07, 2.51) |
| Caribbean | 0.45 (0.34, 0.60) | 1.70 (1.26, 2.25) |  | 0.44 (0.31, 0.61) | 0.85 (0.60, 1.18) |  | -2.95 | -2.35 (-3.20, -1.48) |
| Central Europe | 1.86 (1.46, 2.36) | 1.27 (1.00, 1.60) |  | 1.37 (0.97, 1.91) | 0.68 (0.50, 0.94) |  | -26.45 | -2.00 (-2.31, -1.68) |
| Eastern Europe | 1.07 (0.90, 1.27) | 0.38 (0.33, 0.45) |  | 2.12 (1.68, 2.65) | 0.65 (0.52, 0.81) |  | 97.74 | 2.17 (1.86, 2.49) |
| Western Europe | 2.44 (1.85, 3.19) | 0.45 (0.34, 0.58) |  | 4.30 (3.13, 5.82) | 0.53 (0.39, 0.71) |  | 76.39 | 0.46 (0.33, 0.59) |
| Andean Latin America | 0.50 (0.40, 0.62) | 2.37 (1.84, 2.98) |  | 0.76 (0.55, 1.00) | 1.35 (0.97, 1.78) |  | 50.34 | -2.38 (-2.80, -1.96) |
| Central Latin America | 0.54 (0.41, 0.70) | 0.59 (0.44, 0.78) |  | 1.16 (0.86, 1.60) | 0.48 (0.36, 0.67) |  | 115.08 | -0.70 (-1.05, -0.35) |
| Southern Latin America | 0.14 (0.09, 0.19) | 0.29 (0.20, 0.41) |  | 0.32 (0.22, 0.47) | 0.39 (0.27, 0.56) |  | 137.53 | 1.64 (1.44, 1.84) |
| Tropical Latin America | 0.41 (0.35, 0.47) | 0.41 (0.35, 0.47) |  | 1.02 (0.86, 1.20) | 0.41 (0.35, 0.49) |  | 149.28 | 0.38 (0.26, 0.50) |
| North Africa and Middle East | 3.15 (2.48, 3.94) | 1.72 (1.34, 2.17) |  | 6.74 (5.02, 8.81) | 1.46 (1.08, 1.92) |  | 113.62 | -0.58 (-0.70, -0.46) |
| High-income North America | 0.90 (0.77, 1.04) | 0.27 (0.23, 0.31) |  | 2.91 (2.35, 3.59) | 0.50 (0.41, 0.62) |  | 225.05 | 2.34 (2.14, 2.53) |
| Oceania | 0.06 (0.05, 0.08) | 1.91 (1.46, 2.40) |  | 0.12 (0.09, 0.16) | 1.62 (1.23, 2.08) |  | 96.72 | -0.43 (-0.51, -0.35) |
| Central Sub-Saharan Africa | 0.18 (0.13, 0.24) | 0.66 (0.47, 0.92) |  | 0.35 (0.24, 0.52) | 0.53 (0.36, 0.77) |  | 97.57 | -0.89 (-1.00, -0.77) |
| Eastern Sub-Saharan Africa | 0.72 (0.53, 0.98) | 0.85 (0.62, 1.17) |  | 1.62 (1.19, 2.16) | 0.86 (0.62, 1.16) |  | 125.66 | -0.20 (-0.38, -0.03) |
| Southern Sub-Saharan Africa | 0.74 (0.50, 1.30) | 2.36 (1.56, 4.13) |  | 1.50 (1.25, 1.79) | 2.35 (1.95, 2.81) |  | 101.30 | -0.72 (-1.38, -0.05) |
| Western Sub-Saharan Africa | 2.47 (1.97, 3.03) | 2.65 (2.10, 3.26) |  | 4.56 (3.57, 5.70) | 2.26 (1.74, 2.82) |  | 84.51 | -0.69 (-0.80, -0.58) |

ASMR: age-standardized mortality rate; CI: confidence interval; EAPC: estimated annual percentage changes; GBD: Global Burden of Disease; SDI: socio-demographic index; UI: uncertainty interval.

**Table S8. The deaths and ASMR of primary liver cancer due to hepatitis C in 1990 and 2019 and their change trends from 1990 to 2019.**

| Region | 1990 | |  | 2019 | |  | 1990-2019 | |
| --- | --- | --- | --- | --- | --- | --- | --- | --- |
|  | Deaths  No. x 10^3^ (95% UI) | ASMR per 100,000  No. (95% UI) |  | Deaths  No. x 10^3^ (95% UI) | ASMR per 100,000  No. (95% UI) |  | Percentage change in deaths (%) | EAPC in ASMR  No. (95% CI) |
| SDI region |  |  |  |  |  |  |  |  |
| Low | 2.66 (2.02, 3.32) | 1.33 (1.01, 1.65) |  | 14.20 (11.42, 16.83) | 1.18 (0.94, 1.41) |  | 99.75 | -0.50 (-0.56, -0.45) |
| Low-middle | 6.83 (5.56, 8.25) | 1.34 (1.09, 1.59) |  | 45.17 (37.16, 54.31) | 1.15 (0.93, 1.35) |  | 107.83 | -0.84 (-1.01, -0.66) |
| Middle | 30.46 (25.85, 36.00) | 3.32 (2.83, 3.87) |  | 26.55 (22.66, 30.48) | 1.95 (1.61, 2.32) |  | 48.28 | -2.51 (-2.95, -2.07) |
| Middle-high | 23.05 (19.77, 26.67) | 2.26 (1.94, 2.59) |  | 50.52 (43.62, 56.77) | 1.31 (1.12, 1.50) |  | 15.14 | -2.70 (-3.08, -2.32) |
| High | 21.63 (19.59, 23.69) | 2.08 (1.89, 2.28) |  | 27.96 (23.73, 30.94) | 2.48 (2.16, 2.78) |  | 133.62 | 0.10 (-0.39, 0.60) |
| GBD region |  |  |  |  |  |  |  |  |
| High-income Asia Pacific | 13.59 (12.74, 14.36) | 6.76 (6.33, 7.14) |  | 1.99 (1.42, 2.60) | 5.42 (4.70, 5.99) |  | 105.75 | -1.54 (-2.16, -0.92) |
| Central Asia | 0.49 (0.35, 0.64) | 1.12 (0.82, 1.43) |  | 34.88 (28.70, 41.39) | 3.08 (2.25, 3.95) |  | 303.21 | 3.28 (2.72, 3.84) |
| East Asia | 38.16 (31.68, 46.29) | 4.92 (4.14, 5.89) |  | 9.90 (8.09, 11.94) | 1.78 (1.49, 2.10) |  | -8.60 | -4.92 (-5.62, -4.21) |
| South Asia | 3.74 (2.89, 4.60) | 0.81 (0.63, 1.01) |  | 9.71 (7.33, 12.70) | 0.78 (0.64, 0.94) |  | 164.69 | -0.26 (-0.37, -0.15) |
| Southeast Asia | 3.93 (2.99, 4.96) | 1.78 (1.39, 2.21) |  | 0.68 (0.51, 0.86) | 1.84 (1.39, 2.39) |  | 147.09 | 0.16 (0.08, 0.23) |
| Australasia | 0.14 (0.10, 0.17) | 0.59 (0.45, 0.73) |  | 0.39 (0.26, 0.54) | 1.33 (1.00, 1.69) |  | 392.76 | 3.17 (2.98, 3.35) |
| Caribbean | 0.39 (0.28, 0.52) | 1.56 (1.11, 2.07) |  | 1.93 (1.41, 2.57) | 0.75 (0.51, 1.04) |  | -0.68 | -2.46 (-3.31, -1.61) |
| Central Europe | 2.20 (1.75, 2.71) | 1.57 (1.27, 1.90) |  | 2.72 (2.28, 3.21) | 0.86 (0.63, 1.15) |  | -12.56 | -1.77 (-2.19, -1.34) |
| Eastern Europe | 1.16 (1.00, 1.32) | 0.43 (0.37, 0.48) |  | 17.57 (14.48, 20.79) | 0.77 (0.65, 0.91) |  | 134.20 | 2.49 (2.26, 2.71) |
| Western Europe | 8.58 (7.31, 9.93) | 1.45 (1.24, 1.68) |  | 0.15 (0.09, 0.23) | 1.81 (1.49, 2.15) |  | 104.73 | 0.65 (0.50, 0.80) |
| Andean Latin America | 0.08 (0.05, 0.11) | 0.41 (0.25, 0.61) |  | 3.39 (2.74, 4.11) | 0.28 (0.18, 0.43) |  | 101.96 | -1.64 (-2.06, -1.23) |
| Central Latin America | 1.19 (0.97, 1.41) | 1.57 (1.29, 1.85) |  | 0.74 (0.54, 0.93) | 1.49 (1.21, 1.80) |  | 184.54 | -0.05 (-0.35, 0.24) |
| Southern Latin America | 0.26 (0.19, 0.34) | 0.58 (0.43, 0.75) |  | 2.42 (2.11, 2.70) | 0.87 (0.64, 1.10) |  | 186.51 | 2.11 (1.88, 2.34) |
| Tropical Latin America | 0.70 (0.63, 0.79) | 0.86 (0.77, 0.96) |  | 12.74 (9.25, 17.06) | 1.03 (0.90, 1.15) |  | 243.54 | 1.14 (0.92, 1.35) |
| North Africa and Middle East | 5.11 (4.04, 6.23) | 3.20 (2.53, 3.89) |  | 9.75 (8.36, 11.23) | 3.11 (2.31, 4.09) |  | 149.33 | 0.47 (0.25, 0.68) |
| High-income North America | 2.55 (2.28, 2.80) | 0.72 (0.65, 0.79) |  | 0.05 (0.03, 0.07) | 1.53 (1.32, 1.77) |  | 282.39 | 2.75 (2.54, 2.96) |
| Oceania | 0.02 (0.01, 0.03) | 0.99 (0.67, 1.32) |  | 0.57 (0.40, 0.75) | 0.90 (0.62, 1.23) |  | 112.32 | -0.20 (-0.27, -0.12) |
| Central Sub-Saharan Africa | 0.27 (0.20, 0.35) | 1.41 (1.08, 1.74) |  | 1.28 (0.95, 1.64) | 1.24 (0.92, 1.60) |  | 107.76 | -0.60 (-0.68, -0.53) |
| Eastern Sub-Saharan Africa | 0.55 (0.39, 0.73) | 0.86 (0.63, 1.13) |  | 1.10 (0.91, 1.31) | 0.96 (0.73, 1.21) |  | 133.37 | 0.21 (0.11, 0.30) |
| Southern Sub-Saharan Africa | 0.51 (0.35, 0.78) | 2.07 (1.41, 3.10) |  | 1.91 (1.37, 2.46) | 2.16 (1.80, 2.57) |  | 114.50 | -0.26 (-0.78, 0.26) |
| Western Sub-Saharan Africa | 1.03 (0.75, 1.35) | 1.34 (0.99, 1.72) |  | 14.20 (11.42, 16.83) | 1.20 (0.88, 1.51) |  | 84.54 | -0.45 (-0.50, -0.40) |

ASMR: age-standardized mortality rate; CI: confidence interval; EAPC: estimated annual percentage changes; GBD: Global Burden of Disease; SDI: socio-demographic index; UI: uncertainty interval.

**Table S9. The deaths and ASMR of primary liver cancer due to alcohol use in 1990 and 2019 and their change trends from 1990 to 2019.**

| Characteristics | 1990 | |  | 2019 | |  | 1990-2019 | |
| --- | --- | --- | --- | --- | --- | --- | --- | --- |
|  | Deaths  No. x 10^3^ (95% UI) | ASMR per 100,000  No. (95% UI) |  | Deaths  No. x 10^3^ (95% UI) | ASMR per 100,000  No. (95% UI) |  | Percentage change in deaths (%) | EAPC in ASMR  No. (95% CI) |
| SDI region |  |  |  |  |  |  |  |  |
| Low | 1.94 (1.43, 2.55) | 0.86 (0.64, 1.12) |  | 4.16 (3.12, 5.31) | 0.84 (0.64, 1.06) |  | 114.68 | -0.17 (-0.23, -0.10) |
| Low-middle | 5.10 (4.02, 6.43) | 0.87 (0.69, 1.10) |  | 11.57 (9.17, 14.15) | 0.86 (0.68, 1.05) |  | 126.76 | -0.33 (-0.50, -0.15) |
| Middle | 15.83 (12.22, 20.31) | 1.52 (1.19, 1.94) |  | 29.45 (22.18, 37.36) | 1.18 (0.90, 1.50) |  | 86.00 | -1.41 (-1.81, -1.01) |
| Middle-high | 14.13 (11.43, 17.24) | 1.31 (1.07, 1.60) |  | 18.20 (14.80, 21.80) | 0.89 (0.72, 1.06) |  | 28.80 | -1.90 (-2.19, -1.61) |
| High | 10.83 (9.03, 12.56) | 1.04 (0.87, 1.21) |  | 27.32 (22.49, 32.05) | 1.47 (1.21, 1.72) |  | 152.28 | 1.03 (0.78, 1.27) |
| GBD region |  |  |  |  |  |  |  |  |
| High-income Asia Pacific | 3.04 (2.53, 3.61) | 1.47 (1.23, 1.74) |  | 6.22 (4.96, 7.73) | 1.42 (1.13, 1.77) |  | 104.17 | -0.78 (-1.33, -0.22) |
| Central Asia | 0.40 (0.28, 0.53) | 0.88 (0.61, 1.16) |  | 1.86 (1.31, 2.51) | 2.60 (1.86, 3.39) |  | 361.47 | 2.94 (2.39, 3.49) |
| East Asia | 17.91 (13.36, 24.17) | 1.96 (1.47, 2.62) |  | 17.97 (13.15, 23.27) | 0.84 (0.62, 1.08) |  | 0.34 | -4.40 (-5.22, -3.57) |
| South Asia | 3.59 (2.79, 4.44) | 0.66 (0.51, 0.82) |  | 10.08 (8.09, 12.26) | 0.73 (0.59, 0.89) |  | 180.46 | 0.27 (0.17, 0.37) |
| Southeast Asia | 3.62 (2.68, 4.77) | 1.44 (1.08, 1.88) |  | 11.18 (7.88, 15.39) | 1.90 (1.34, 2.59) |  | 208.50 | 1.07 (0.96, 1.19) |
| Australasia | 0.20 (0.15, 0.24) | 0.83 (0.66, 0.99) |  | 0.77 (0.60, 0.93) | 1.58 (1.24, 1.93) |  | 288.29 | 2.65 (2.41, 2.89) |
| Caribbean | 0.54 (0.40, 0.69) | 2.06 (1.53, 2.64) |  | 0.59 (0.43, 0.80) | 1.15 (0.83, 1.54) |  | 11.05 | -1.93 (-2.77, -1.08) |
| Central Europe | 3.16 (2.63, 3.67) | 2.13 (1.78, 2.47) |  | 3.09 (2.40, 3.85) | 1.43 (1.11, 1.77) |  | -2.10 | -1.05 (-1.46, -0.63) |
| Eastern Europe | 1.42 (1.22, 1.60) | 0.50 (0.43, 0.57) |  | 3.64 (3.00, 4.38) | 1.06 (0.87, 1.28) |  | 157.31 | 2.97 (2.64, 3.29) |
| Western Europe | 6.92 (5.61, 8.17) | 1.19 (0.97, 1.40) |  | 14.02 (11.22, 16.81) | 1.58 (1.27, 1.88) |  | 102.76 | 0.97 (0.82, 1.13) |
| Andean Latin America | 0.31 (0.22, 0.42) | 1.61 (1.15, 2.14) |  | 0.59 (0.41, 0.82) | 1.09 (0.75, 1.51) |  | 89.10 | -1.70 (-2.22, -1.17) |
| Central Latin America | 0.86 (0.66, 1.06) | 1.06 (0.82, 1.31) |  | 2.59 (2.00, 3.26) | 1.12 (0.86, 1.41) |  | 200.56 | 0.33 (-0.05, 0.70) |
| Southern Latin America | 0.25 (0.18, 0.33) | 0.54 (0.39, 0.71) |  | 0.65 (0.47, 0.83) | 0.77 (0.56, 0.98) |  | 159.54 | 1.96 (1.66, 2.26) |
| Tropical Latin America | 0.52 (0.45, 0.59) | 0.58 (0.50, 0.65) |  | 1.82 (1.57, 2.07) | 0.76 (0.65, 0.86) |  | 252.03 | 1.41 (1.25, 1.56) |
| North Africa and Middle East | 0.97 (0.64, 1.38) | 0.58 (0.39, 0.82) |  | 2.57 (1.65, 3.91) | 0.61 (0.39, 0.92) |  | 165.52 | 0.50 (0.33, 0.67) |
| High-income North America | 2.22 (1.97, 2.47) | 0.64 (0.56, 0.70) |  | 8.86 (7.23, 10.43) | 1.44 (1.18, 1.69) |  | 298.38 | 2.78 (2.72, 2.85) |
| Oceania | 0.01 (0.01, 0.02) | 0.47 (0.30, 0.69) |  | 0.03 (0.02, 0.04) | 0.46 (0.30, 0.68) |  | 128.49 | 0.19 (0.11, 0.27) |
| Central Sub-Saharan Africa | 0.08 (0.06, 0.12) | 0.39 (0.26, 0.55) |  | 0.18 (0.12, 0.27) | 0.35 (0.23, 0.51) |  | 116.44 | -0.46 (-0.54, -0.38) |
| Eastern Sub-Saharan Africa | 0.59 (0.42, 0.84) | 0.82 (0.59, 1.16) |  | 1.38 (0.98, 1.93) | 0.89 (0.64, 1.23) |  | 134.33 | 0.11 (-0.04, 0.27) |
| Southern Sub-Saharan Africa | 0.36 (0.22, 0.70) | 1.31 (0.79, 2.50) |  | 0.80 (0.63, 0.99) | 1.40 (1.11, 1.71) |  | 118.93 | -0.50 (-1.20, 0.21) |
| Western Sub-Saharan Africa | 0.88 (0.64, 1.21) | 1.03 (0.75, 1.42) |  | 1.85 (1.33, 2.45) | 1.06 (0.77, 1.40) |  | 110.60 | -0.04 (-0.14, 0.07) |

ASMR: age-standardized mortality rate; CI: confidence interval; EAPC: estimated annual percentage changes; GBD: Global Burden of Disease; SDI: socio-demographic index; UI: uncertainty interval.

**Table S10. The deaths and ASMR of primary liver cancer due to NASH in 1990 and 2019 and their change trends from 1990 to 2019.**

| Region | 1990 | |  | 2019 | |  | 1990-2019 | |
| --- | --- | --- | --- | --- | --- | --- | --- | --- |
|  | Deaths  No. x 10^3^ (95% UI) | ASMR per 100,000  No. (95% UI) |  | Deaths  No. x 10^3^ (95% UI) | ASMR per 100,000  No. (95% UI) |  | Percentage change in deaths (%) | EAPC in ASMR  No. (95% CI) |
| SDI region |  |  |  |  |  |  |  |  |
| Low | 0.77 (0.59, 1.01) | 0.36 (0.27, 0.47) |  | 1.80 (1.37, 2.37) | 0.37 (0.29, 0.49) |  | 133.08 | 0.03 (-0.02, 0.09) |
| Low-middle | 2.12 (1.74, 2.62) | 0.38 (0.30, 0.47) |  | 4.84 (3.89, 6.06) | 0.38 (0.30, 0.47) |  | 128.20 | -0.38 (-0.58, -0.18) |
| Middle | 7.41 (6.03, 9.08) | 0.74 (0.60, 0.91) |  | 13.68 (10.95, 17.31) | 0.58 (0.47, 0.73) |  | 84.58 | -1.54 (-2.01, -1.06) |
| Middle-high | 4.75 (3.87, 5.77) | 0.45 (0.37, 0.55) |  | 6.15 (4.98, 7.56) | 0.30 (0.25, 0.37) |  | 29.57 | -2.25 (-2.71, -1.78) |
| High | 2.74 (2.22, 3.38) | 0.26 (0.21, 0.32) |  | 8.24 (6.48, 10.35) | 0.42 (0.33, 0.53) |  | 200.58 | 1.45 (1.07, 1.83) |
| GBD region |  |  |  |  |  |  |  |  |
| High-income Asia Pacific | 0.96 (0.81, 1.14) | 0.48 (0.40, 0.57) |  | 2.52 (1.92, 3.19) | 0.51 (0.39, 0.64) |  | 162.15 | -0.34 (-0.99, 0.31) |
| Central Asia | 0.08 (0.06, 0.12) | 0.18 (0.13, 0.27) |  | 0.44 (0.31, 0.62) | 0.67 (0.47, 0.95) |  | 433.18 | 4.14 (3.62, 4.67) |
| East Asia | 9.08 (7.22, 11.29) | 1.05 (0.84, 1.29) |  | 9.79 (7.87, 12.10) | 0.49 (0.39, 0.60) |  | 7.89 | -4.10 (-4.90, -3.29) |
| South Asia | 1.40 (1.12, 1.72) | 0.28 (0.21, 0.34) |  | 4.16 (3.35, 5.05) | 0.32 (0.25, 0.39) |  | 196.11 | 0.40 (0.28, 0.51) |
| Southeast Asia | 1.24 (0.94, 1.63) | 0.52 (0.39, 0.70) |  | 4.08 (2.90, 5.63) | 0.75 (0.53, 1.03) |  | 227.83 | 1.31 (1.20, 1.42) |
| Australasia | 0.04 (0.03, 0.05) | 0.15 (0.11, 0.21) |  | 0.22 (0.16, 0.30) | 0.45 (0.33, 0.59) |  | 521.72 | 4.02 (3.72, 4.32) |
| Caribbean | 0.14 (0.10, 0.19) | 0.54 (0.38, 0.75) |  | 0.17 (0.11, 0.23) | 0.32 (0.22, 0.45) |  | 19.39 | -1.67 (-2.56, -0.76) |
| Central Europe | 0.60 (0.47, 0.75) | 0.42 (0.33, 0.53) |  | 0.60 (0.43, 0.82) | 0.27 (0.20, 0.37) |  | 0.26 | -1.24 (-1.70, -0.78) |
| Eastern Europe | 0.30 (0.26, 0.36) | 0.11 (0.09, 0.13) |  | 0.82 (0.68, 1.00) | 0.24 (0.20, 0.29) |  | 171.28 | 3.14 (2.89, 3.38) |
| Western Europe | 1.11 (0.84, 1.47) | 0.19 (0.14, 0.25) |  | 2.75 (2.01, 3.73) | 0.29 (0.21, 0.38) |  | 147.11 | 1.49 (1.31, 1.67) |
| Andean Latin America | 0.08 (0.06, 0.12) | 0.44 (0.30, 0.62) |  | 0.20 (0.13, 0.28) | 0.37 (0.25, 0.52) |  | 137.03 | -1.05 (-1.46, -0.63) |
| Central Latin America | 0.22 (0.17, 0.28) | 0.27 (0.21, 0.36) |  | 0.78 (0.61, 1.01) | 0.34 (0.27, 0.44) |  | 258.20 | 0.95 (0.64, 1.26) |
| Southern Latin America | 0.06 (0.04, 0.08) | 0.13 (0.09, 0.19) |  | 0.20 (0.14, 0.29) | 0.24 (0.16, 0.33) |  | 247.50 | 2.83 (2.61, 3.06) |
| Tropical Latin America | 0.10 (0.09, 0.12) | 0.12 (0.10, 0.14) |  | 0.38 (0.32, 0.45) | 0.16 (0.14, 0.19) |  | 267.04 | 1.54 (1.31, 1.77) |
| North Africa and Middle East | 0.83 (0.60, 1.17) | 0.52 (0.37, 0.73) |  | 2.72 (1.89, 3.93) | 0.67 (0.47, 0.94) |  | 226.93 | 1.11 (0.96, 1.27) |
| High-income North America | 0.72 (0.61, 0.85) | 0.20 (0.17, 0.23) |  | 2.89 (2.35, 3.50) | 0.45 (0.37, 0.55) |  | 300.11 | 3.03 (2.83, 3.23) |
| Oceania | 0.01 (0.01, 0.01) | 0.31 (0.21, 0.44) |  | 0.02 (0.01, 0.03) | 0.33 (0.23, 0.47) |  | 146.59 | 0.26 (0.20, 0.32) |
| Central Sub-Saharan Africa | 0.04 (0.02, 0.05) | 0.17 (0.12, 0.25) |  | 0.09 (0.06, 0.13) | 0.18 (0.12, 0.26) |  | 143.74 | -0.18 (-0.26, -0.09) |
| Eastern Sub-Saharan Africa | 0.24 (0.18, 0.33) | 0.35 (0.26, 0.48) |  | 0.63 (0.46, 0.84) | 0.43 (0.31, 0.57) |  | 163.00 | 0.52 (0.41, 0.63) |
| Southern Sub-Saharan Africa | 0.18 (0.12, 0.29) | 0.66 (0.43, 1.07) |  | 0.43 (0.35, 0.53) | 0.80 (0.65, 0.97) |  | 144.75 | 0.18 (-0.42, 0.79) |
| Western Sub-Saharan Africa | 0.37 (0.27, 0.49) | 0.45 (0.33, 0.60) |  | 0.84 (0.63, 1.12) | 0.50 (0.37, 0.66) |  | 128.41 | 0.20 (0.11, 0.29) |

ASMR: age-standardized mortality rate; CI: confidence interval; EAPC: estimated annual percentage changes; GBD: Global Burden of Disease; NASH: nonalcoholic steatohepatitis; SDI: socio-demographic index; UI: uncertainty interval.

**Table S11. The deaths and ASMR of primary liver cancer due to other causes in 1990 and 2019 and their change trends from 1990 to 2019.**

| Region | 1990 | |  | 2019 | |  | 1990-2019 | |
| --- | --- | --- | --- | --- | --- | --- | --- | --- |
|  | Deaths  No. x 10^3^ (95% UI) | ASMR per 100,000  No. (95% UI) |  | Deaths  No. x 10^3^ (95% UI) | ASMR per 100,000  No. (95% UI) |  | Percentage change in deaths (%) | EAPC in ASMR  No. (95% CI) |
| SDI region |  |  |  |  |  |  |  |  |
| Low |  | 0.27 (0.21, 0.35) |  | 2.02 (1.58, 2.51) | 0.24 (0.19, 0.30) |  | 61.72 | -0.48 (-0.52, -0.44) |
| Low-middle | 1.25 (0.98, 1.60) | 0.35 (0.29, 0.42) |  | 3.45 (2.87, 4.14) | 0.23 (0.19, 0.28) |  | 17.91 | -2.02 (-2.38, -1.65) |
| Middle | 2.92 (2.44, 3.56) | 0.95 (0.78, 1.16) |  | 10.35 (8.37, 12.55) | 0.42 (0.34, 0.51) |  | -10.75 | -3.81 (-4.44, -3.18) |
| Middle-high | 11.59 (9.54, 14.07) | 0.60 (0.49, 0.72) |  | 4.64 (3.82, 5.57) | 0.25 (0.21, 0.30) |  | -29.01 | -4.26 (-4.88, -3.64) |
| High | 6.54 (5.36, 7.91) | 0.23 (0.20, 0.28) |  | 5.10 (4.12, 6.23) | 0.29 (0.24, 0.35) |  | 122.84 | 0.53 (0.23, 0.82) |
| GBD region |  |  |  |  |  |  |  |  |
| High-income Asia Pacific | 2.29 (1.89, 2.73) | 0.36 (0.31, 0.42) |  | 1.36 (1.07, 1.69) | 0.32 (0.26, 0.39) |  | 89.39 | -1.04 (-1.64, -0.44) |
| Central Asia | 0.72 (0.60, 0.85) | 0.16 (0.12, 0.21) |  | 0.25 (0.18, 0.35) | 0.33 (0.23, 0.45) |  | 158.41 | 2.37 (1.97, 2.76) |
| East Asia | 0.10 (0.07, 0.12) | 1.70 (1.38, 2.08) |  | 11.10 (8.92, 13.55) | 0.57 (0.46, 0.68) |  | -32.92 | -5.27 (-6.07, -4.46) |
| South Asia | 16.55 (13.47, 20.34) | 0.14 (0.12, 0.16) |  | 2.15 (1.81, 2.52) | 0.14 (0.12, 0.16) |  | 62.08 | -0.18 (-0.26, -0.11) |
| Southeast Asia | 1.33 (1.14, 1.53) | 0.27 (0.21, 0.35) |  | 1.46 (1.09, 1.93) | 0.25 (0.18, 0.33) |  | 46.29 | -0.34 (-0.39, -0.30) |
| Australasia | 1.00 (0.76, 1.35) | 0.10 (0.08, 0.13) |  | 0.09 (0.06, 0.12) | 0.20 (0.15, 0.26) |  | 290.30 | 2.63 (2.43, 2.84) |
| Caribbean | 0.02 (0.02, 0.03) | 0.42 (0.31, 0.56) |  | 0.11 (0.07, 0.15) | 0.21 (0.15, 0.30) |  | -9.69 | -2.24 (-3.01, -1.46) |
| Central Europe | 0.12 (0.09, 0.16) | 0.21 (0.17, 0.26) |  | 0.22 (0.15, 0.30) | 0.12 (0.08, 0.15) |  | -26.53 | -1.85 (-2.23, -1.47) |
| Eastern Europe | 0.29 (0.23, 0.36) | 0.13 (0.11, 0.14) |  | 0.38 (0.31, 0.46) | 0.15 (0.11, 0.18) |  | 37.56 | 0.71 (0.52, 0.90) |
| Western Europe | 0.28 (0.25, 0.31) | 0.16 (0.13, 0.20) |  | 1.65 (1.22, 2.21) | 0.20 (0.16, 0.26) |  | 98.22 | 0.86 (0.73, 1.00) |
| Andean Latin America | 0.83 (0.64, 1.08) | 0.39 (0.28, 0.53) |  | 0.14 (0.10, 0.19) | 0.25 (0.17, 0.34) |  | 41.49 | -1.95 (-2.32, -1.57) |
| Central Latin America | 0.10 (0.08, 0.13) | 0.24 (0.19, 0.30) |  | 0.50 (0.39, 0.63) | 0.21 (0.17, 0.27) |  | 88.31 | -0.20 (-0.49, 0.09) |
| Southern Latin America | 0.27 (0.23, 0.31) | 0.11 (0.08, 0.15) |  | 0.11 (0.08, 0.15) | 0.14 (0.10, 0.19) |  | 113.96 | 1.45 (1.24, 1.66) |
| Tropical Latin America | 0.05 (0.04, 0.07) | 0.13 (0.11, 0.15) |  | 0.30 (0.26, 0.34) | 0.13 (0.11, 0.15) |  | 99.07 | 0.45 (0.32, 0.58) |
| North Africa and Middle East | 0.15 (0.14, 0.17) | 0.38 (0.28, 0.50) |  | 1.66 (1.19, 2.33) | 0.36 (0.25, 0.50) |  | 95.89 | 0.15 (-0.00, 0.31) |
| High-income North America | 0.85 (0.65, 1.10) | 0.21 (0.18, 0.23) |  | 2.06 (1.71, 2.44) | 0.36 (0.30, 0.42) |  | 202.86 | 1.90 (1.76, 2.04) |
| Oceania | 0.68 (0.59, 0.77) | 0.17 (0.12, 0.24) |  | 0.01 (0.01, 0.02) | 0.15 (0.11, 0.21) |  | 94.93 | -0.29 (-0.35, -0.23) |
| Central Sub-Saharan Africa | 0.01 (0.01, 0.01) | 0.21 (0.15, 0.28) |  | 0.20 (0.12, 0.32) | 0.16 (0.11, 0.24) |  | 40.57 | -0.86 (-0.91, -0.81) |
| Eastern Sub-Saharan Africa | 0.14 (0.10, 0.21) | 0.28 (0.20, 0.36) |  | 0.77 (0.58, 1.00) | 0.27 (0.21, 0.35) |  | 73.82 | -0.09 (-0.18, -0.00) |
| Southern Sub-Saharan Africa | 0.45 (0.32, 0.62) | 0.34 (0.24, 0.53) |  | 0.21 (0.17, 0.26) | 0.34 (0.28, 0.42) |  | 83.50 | -0.40 (-0.91, 0.11) |
| Western Sub-Saharan Africa | 0.12 (0.08, 0.18) | 0.33 (0.26, 0.42) |  | 0.82 (0.62, 1.05) | 0.28 (0.21, 0.37) |  | 46.30 | -0.65 (-0.81, -0.50) |

ASMR: age-standardized mortality rate; CI: confidence interval; EAPC: estimated annual percentage changes; GBD: Global Burden of Disease; SDI: socio-demographic index; UI: uncertainty interval.

**Table S12**. **The incident cases and ASIR of primary liver cancer in 1990 and 2019 and their change trends from 1990 to 2019 at national level.**

| Nation | 1990 | |  | 2019 | |  | 1990-2019 | |
| --- | --- | --- | --- | --- | --- | --- | --- | --- |
|  | Incident cases  No. (95% UI) | ASIR per 100,000  No. (95% UI) |  | Incident cases  No. x 10^3^ (95% UI) | ASIR per 100,000  No. (95% UI) |  | Percentage change in incident cases  No. (%) | EAPC in ASIR  No. (95% CI) |
| Afghanistan | 843.13 (646.19, 1082.40) | 11.48 (8.93, 14.53) |  | 1407.83 (1056.52, 1848.10) | 9.82 (7.63, 12.44) |  | 66.98 | -0.60 (-0.70, -0.50) |
| Albania | 224.03 (209.00, 239.59) | 10.85 (10.10, 11.60) |  | 281.18 (204.76, 376.33) | 6.58 (4.82, 8.76) |  | 25.51 | -2.66 (-3.17, -2.15) |
| Algeria | 207.30 (167.77, 255.89) | 1.68 (1.38, 2.06) |  | 720.08 (561.62, 908.00) | 2.21 (1.73, 2.78) |  | 247.35 | 0.94 (0.79, 1.10) |
| American Samoa | 1.34 (1.01, 1.65) | 5.47 (4.19, 6.72) |  | 3.41 (2.80, 4.19) | 6.88 (5.69, 8.35) |  | 154.79 | 1.16 (0.97, 1.34) |
| Andorra | 5.25 (3.89, 7.20) | 9.57 (7.16, 12.99) |  | 15.08 (11.35, 19.69) | 10.95 (8.23, 14.37) |  | 187.10 | 0.51 (0.48, 0.53) |
| Angola | 112.31 (84.27, 146.93) | 2.54 (1.94, 3.28) |  | 293.55 (225.80, 376.88) | 2.39 (1.92, 2.99) |  | 161.37 | -0.37 (-0.45, -0.28) |
| Antigua and Barbuda | 4.09 (3.71, 4.51) | 7.70 (6.99, 8.51) |  | 2.68 (2.27, 3.14) | 2.69 (2.29, 3.13) |  | -34.42 | -3.98 (-4.99, -2.95) |
| Argentina | 453.09 (394.60, 514.45) | 1.40 (1.22, 1.59) |  | 1105.03 (867.29, 1380.78) | 2.05 (1.61, 2.56) |  | 143.89 | 2.01 (1.78, 2.25) |
| Armenia | 29.52 (27.45, 32.42) | 1.09 (1.02, 1.18) |  | 271.07 (225.36, 323.14) | 6.60 (5.47, 7.85) |  | 818.11 | 9.46 (7.92, 11.02) |
| Australia | 383.65 (367.58, 400.18) | 1.98 (1.89, 2.06) |  | 1799.34 (1387.71, 2309.22) | 4.52 (3.48, 5.84) |  | 369.01 | 3.24 (2.96, 3.53) |
| Austria | 397.97 (378.52, 418.25) | 3.48 (3.31, 3.66) |  | 939.40 (752.63, 1173.18) | 5.50 (4.41, 6.89) |  | 136.05 | 1.84 (1.49, 2.18) |
| Azerbaijan | 51.02 (45.96, 57.77) | 0.98 (0.89, 1.10) |  | 358.38 (273.84, 471.96) | 4.07 (3.11, 5.46) |  | 602.47 | 6.00 (4.01, 8.03) |
| Bahamas | 13.05 (11.49, 14.56) | 8.15 (7.18, 9.12) |  | 12.21 (9.98, 15.02) | 3.10 (2.56, 3.81) |  | -6.45 | -3.61 (-4.50, -2.71) |
| Bahrain | 8.96 (7.47, 10.63) | 5.62 (4.72, 6.58) |  | 44.42 (34.42, 57.48) | 5.53 (4.39, 6.91) |  | 395.83 | -0.84 (-1.24, -0.45) |
| Bangladesh | 1741.95 (1383.58, 2137.96) | 3.11 (2.55, 3.77) |  | 3404.73 (2661.33, 4240.00) | 2.58 (2.03, 3.21) |  | 95.46 | -1.09 (-1.23, -0.95) |
| Barbados | 8.01 (5.94, 10.33) | 2.78 (2.05, 3.58) |  | 12.59 (10.45, 15.00) | 2.63 (2.17, 3.14) |  | 57.09 | -0.37 (-0.60, -0.13) |
| Belarus | 163.16 (153.79, 173.12) | 1.29 (1.22, 1.38) |  | 380.53 (274.53, 512.22) | 2.49 (1.81, 3.34) |  | 133.22 | 3.01 (2.36, 3.66) |
| Belgium | 443.21 (418.46, 466.89) | 2.89 (2.74, 3.04) |  | 927.59 (733.80, 1171.29) | 4.27 (3.36, 5.42) |  | 109.29 | 1.23 (0.92, 1.54) |
| Belize | 5.66 (5.13, 6.25) | 5.93 (5.36, 6.56) |  | 8.44 (7.21, 9.75) | 3.04 (2.60, 3.52) |  | 49.17 | -2.56 (-3.15, -1.96) |
| Benin | 138.22 (111.08, 175.49) | 6.54 (5.24, 8.36) |  | 249.08 (182.28, 337.19) | 4.78 (3.57, 6.37) |  | 80.20 | -1.41 (-1.55, -1.26) |
| Bermuda | 4.40 (3.95, 4.86) | 7.07 (6.36, 7.81) |  | 2.67 (2.21, 3.27) | 2.09 (1.73, 2.56) |  | -39.37 | -4.11 (-5.17, -3.04) |
| Bhutan | 5.90 (3.81, 8.67) | 2.18 (1.47, 3.20) |  | 16.97 (11.68, 24.00) | 3.04 (2.11, 4.29) |  | 187.79 | 1.22 (1.13, 1.30) |
| Bolivia | 162.44 (122.43, 212.79) | 4.92 (3.72, 6.43) |  | 399.34 (296.15, 520.26) | 4.58 (3.41, 5.93) |  | 145.85 | -0.34 (-0.45, -0.23) |
| Bosnia and Herzegovina | 228.56 (212.35, 245.66) | 5.50 (5.11, 5.90) |  | 452.01 (359.24, 569.63) | 7.53 (5.99, 9.46) |  | 97.76 | 1.54 (1.21, 1.87) |
| Botswana | 5.84 (2.84, 13.38) | 0.97 (0.47, 2.17) |  | 22.24 (15.47, 30.52) | 1.47 (1.07, 1.95) |  | 280.90 | 0.57 (-0.19, 1.35) |
| Brazil | 1794.07 (1725.95, 1850.22) | 1.96 (1.88, 2.03) |  | 5555.06 (5239.30, 5841.00) | 2.37 (2.23, 2.49) |  | 209.63 | 1.09 (0.93, 1.24) |
| Brunei | 9.64 (7.79, 11.59) | 9.68 (7.88, 11.49) |  | 38.25 (32.09, 45.74) | 11.75 (9.96, 13.78) |  | 296.86 | 1.43 (0.94, 1.91) |
| Bulgaria | 782.15 (732.38, 836.43) | 6.19 (5.81, 6.61) |  | 613.86 (489.38, 757.89) | 4.50 (3.58, 5.58) |  | -21.52 | -0.50 (-0.99, -0.01) |
| Burkina Faso | 143.02 (112.21, 179.18) | 2.97 (2.38, 3.62) |  | 233.21 (174.37, 302.12) | 2.18 (1.66, 2.75) |  | 63.06 | -1.24 (-1.43, -1.06) |
| Burundi | 93.72 (68.15, 124.12) | 3.69 (2.71, 4.83) |  | 141.42 (96.53, 221.01) | 2.89 (1.98, 4.53) |  | 50.89 | -1.41 (-1.59, -1.23) |
| Cambodia | 621.92 (485.76, 805.20) | 10.86 (8.82, 13.11) |  | 1143.79 (887.21, 1413.26) | 9.20 (7.26, 11.26) |  | 83.91 | -0.83 (-0.92, -0.73) |
| Cameroon | 30.08 (21.02, 44.75) | 0.65 (0.45, 0.97) |  | 84.95 (60.02, 116.24) | 0.65 (0.47, 0.87) |  | 182.39 | -0.17 (-0.41, 0.06) |
| Canada | 656.55 (627.53, 682.49) | 2.05 (1.96, 2.13) |  | 3108.27 (2397.06, 3996.99) | 4.75 (3.62, 6.11) |  | 373.43 | 3.27 (3.08, 3.45) |
| Cape Verde | 2.60 (2.23, 2.98) | 1.09 (0.94, 1.27) |  | 50.42 (41.40, 61.07) | 11.56 (9.52, 13.94) |  | 1840.57 | 6.37 (4.15, 8.63) |
| Central African Republic | 46.51 (31.97, 65.40) | 3.55 (2.50, 4.86) |  | 73.80 (46.49, 111.23) | 3.18 (2.08, 4.66) |  | 58.68 | -0.87 (-1.10, -0.63) |
| Chad | 182.32 (140.90, 234.13) | 6.16 (4.75, 7.91) |  | 317.44 (237.21, 412.08) | 5.31 (4.05, 6.84) |  | 74.12 | -0.67 (-0.73, -0.60) |
| Chile | 215.11 (200.60, 231.13) | 2.14 (1.99, 2.30) |  | 713.59 (554.17, 899.33) | 2.98 (2.31, 3.75) |  | 231.73 | 1.78 (1.46, 2.11) |
| China | 236824.63 (199322.36, 280115.93) | 25.71 (21.73, 30.35) |  | 210462.35 (174831.65, 251195.06) | 10.46 (8.74, 12.42) |  | -11.13 | -4.67 (-5.50, -3.84) |
| Colombia | 509.13 (482.98, 534.89) | 2.84 (2.68, 2.98) |  | 1317.95 (1001.54, 1689.85) | 2.51 (1.91, 3.22) |  | 158.86 | -0.22 (-0.70, 0.26) |
| Comoros | 7.19 (3.74, 12.10) | 3.09 (1.69, 5.16) |  | 13.75 (9.40, 21.44) | 2.79 (1.94, 4.33) |  | 91.32 | -0.56 (-0.72, -0.41) |
| Congo | 45.80 (33.73, 62.76) | 3.97 (2.99, 5.38) |  | 76.91 (54.81, 106.67) | 2.89 (2.11, 3.94) |  | 67.92 | -1.48 (-1.61, -1.35) |
| Cook Islands | 1.74 (1.44, 2.06) | 13.25 (11.01, 15.61) |  | 2.78 (2.23, 3.40) | 11.37 (9.15, 13.93) |  | 59.59 | -0.47 (-0.60, -0.35) |
| Costa Rica | 95.76 (88.64, 102.15) | 5.42 (5.02, 5.80) |  | 261.38 (201.64, 331.02) | 5.14 (3.97, 6.51) |  | 172.94 | -0.41 (-0.96, 0.15) |
| Cote d'Ivoire | 331.10 (247.42, 425.51) | 7.46 (5.69, 9.39) |  | 534.04 (383.55, 737.98) | 4.73 (3.50, 6.43) |  | 61.29 | -2.44 (-2.74, -2.13) |
| Croatia | 199.99 (184.97, 215.70) | 3.13 (2.90, 3.37) |  | 320.61 (251.99, 403.62) | 3.73 (2.92, 4.75) |  | 60.31 | 1.22 (0.81, 1.64) |
| Cuba | 659.16 (624.36, 689.64) | 6.35 (6.01, 6.65) |  | 441.60 (355.36, 547.85) | 2.34 (1.89, 2.90) |  | -33.01 | -3.76 (-4.99, -2.51) |
| Cyprus | 25.04 (20.63, 29.41) | 3.15 (2.67, 3.65) |  | 70.06 (59.71, 82.23) | 3.69 (3.16, 4.32) |  | 179.75 | 0.85 (0.63, 1.07) |
| Czech Republic | 596.26 (565.68, 624.34) | 4.32 (4.10, 4.52) |  | 614.55 (503.61, 754.66) | 2.96 (2.42, 3.65) |  | 3.07 | -1.46 (-1.66, -1.26) |
| Democratic Republic of the Congo | 455.66 (366.73, 563.89) | 2.47 (2.02, 2.96) |  | 869.70 (658.75, 1142.34) | 2.14 (1.65, 2.79) |  | 90.87 | -0.56 (-0.63, -0.50) |
| Denmark | 190.66 (179.88, 201.02) | 2.41 (2.28, 2.54) |  | 433.72 (341.22, 553.17) | 3.98 (3.11, 5.10) |  | 127.48 | 2.09 (1.86, 2.32) |
| Djibouti | 5.09 (3.23, 9.02) | 3.15 (2.06, 5.44) |  | 19.89 (12.01, 33.55) | 3.22 (2.07, 5.23) |  | 290.80 | -0.03 (-0.17, 0.10) |
| Dominica | 6.15 (5.40, 6.93) | 8.59 (7.50, 9.72) |  | 2.84 (2.31, 3.53) | 3.18 (2.58, 3.95) |  | -53.75 | -3.57 (-4.36, -2.77) |
| Dominican Republic | 155.46 (136.14, 176.28) | 3.91 (3.38, 4.45) |  | 434.55 (294.22, 660.24) | 4.64 (3.16, 6.99) |  | 179.53 | 0.82 (0.32, 1.32) |
| Ecuador | 154.87 (139.22, 171.76) | 2.82 (2.50, 3.13) |  | 504.83 (400.08, 652.63) | 3.40 (2.72, 4.37) |  | 225.97 | 1.11 (0.79, 1.43) |
| Egypt | 5143.28 (4090.43, 6179.05) | 16.75 (13.10, 20.20) |  | 13840.91 (9844.18, 19063.53) | 20.92 (15.09, 28.51) |  | 169.11 | 1.63 (1.25, 2.02) |
| El Salvador | 102.87 (95.21, 110.56) | 3.37 (3.10, 3.64) |  | 117.63 (88.92, 152.95) | 1.99 (1.50, 2.59) |  | 14.35 | -2.00 (-2.69, -1.31) |
| Equatorial Guinea | 6.62 (4.86, 8.65) | 3.03 (2.27, 3.96) |  | 16.11 (9.21, 24.20) | 3.17 (1.87, 4.63) |  | 143.41 | 0.07 (-0.14, 0.28) |
| Eritrea | 35.49 (23.65, 54.24) | 3.21 (2.17, 4.86) |  | 87.35 (59.78, 128.33) | 3.10 (2.17, 4.41) |  | 146.12 | -0.47 (-0.63, -0.30) |
| Estonia | 41.76 (38.91, 44.73) | 2.06 (1.92, 2.21) |  | 94.23 (72.01, 118.21) | 3.71 (2.84, 4.71) |  | 125.65 | 0.98 (0.48, 1.48) |
| Eswatini | 17.80 (11.29, 34.68) | 5.64 (3.67, 10.81) |  | 118.92 (34.02, 219.98) | 18.43 (5.61, 32.97) |  | 568.27 | 5.02 (3.71, 6.34) |
| Ethiopia | 642.46 (445.74, 925.82) | 2.91 (2.10, 3.99) |  | 1155.19 (929.55, 1475.70) | 2.73 (2.21, 3.47) |  | 79.81 | -0.41 (-0.50, -0.33) |
| Federated States of Micronesia | 5.49 (4.05, 7.19) | 11.16 (8.30, 14.48) |  | 7.88 (5.02, 11.54) | 10.37 (6.93, 14.51) |  | 43.53 | -0.31 (-0.44, -0.17) |
| Fiji | 22.36 (18.02, 27.96) | 5.72 (4.62, 7.07) |  | 47.28 (35.91, 61.11) | 6.04 (4.67, 7.62) |  | 111.40 | 0.73 (0.35, 1.12) |
| Finland | 236.05 (220.96, 250.54) | 3.32 (3.11, 3.53) |  | 670.20 (529.71, 857.19) | 5.54 (4.35, 7.14) |  | 183.92 | 2.14 (1.96, 2.31) |
| France | 3975.71 (3786.31, 4158.99) | 4.98 (4.75, 5.21) |  | 8449.28 (6481.85, 10931.63) | 6.66 (5.05, 8.72) |  | 112.52 | 0.79 (0.54, 1.03) |
| Gabon | 19.68 (14.31, 25.42) | 3.35 (2.48, 4.27) |  | 34.36 (22.68, 48.65) | 3.27 (2.15, 4.67) |  | 74.56 | -0.30 (-0.42, -0.18) |
| Gambia | 121.37 (88.63, 162.12) | 29.75 (22.26, 39.05) |  | 409.16 (292.11, 540.99) | 38.21 (27.53, 49.67) |  | 237.12 | 0.51 (0.34, 0.68) |
| Georgia | 68.38 (61.70, 75.46) | 1.14 (1.03, 1.25) |  | 199.72 (164.42, 243.86) | 3.52 (2.87, 4.32) |  | 192.06 | 3.06 (1.77, 4.37) |
| Germany | 3171.06 (2930.52, 3424.81) | 2.55 (2.36, 2.75) |  | 9299.52 (7292.47, 11872.22) | 5.07 (3.96, 6.44) |  | 193.26 | 2.60 (2.15, 3.04) |
| Ghana | 392.50 (299.18, 526.01) | 5.83 (4.49, 7.75) |  | 979.95 (724.55, 1259.87) | 5.68 (4.31, 7.20) |  | 149.67 | -0.37 (-0.58, -0.15) |
| Greece | 346.17 (324.04, 368.05) | 2.28 (2.14, 2.42) |  | 802.46 (642.53, 1002.39) | 3.52 (2.78, 4.42) |  | 131.81 | 1.73 (1.61, 1.85) |
| Greenland | 1.70 (1.46, 2.01) | 4.48 (3.91, 5.18) |  | 4.44 (3.48, 5.66) | 6.20 (4.93, 7.74) |  | 160.71 | 1.16 (1.07, 1.26) |
| Grenada | 6.37 (5.72, 7.04) | 8.92 (7.97, 9.89) |  | 3.29 (2.88, 3.76) | 2.96 (2.61, 3.36) |  | -48.26 | -4.11 (-5.07, -3.15) |
| Guam | 3.03 (2.56, 3.59) | 3.75 (3.21, 4.37) |  | 11.34 (9.15, 13.97) | 5.89 (4.80, 7.19) |  | 273.99 | 2.11 (1.95, 2.27) |
| Guatemala | 344.16 (305.12, 387.90) | 9.33 (8.27, 10.50) |  | 498.41 (394.15, 628.44) | 4.47 (3.55, 5.60) |  | 44.82 | -3.21 (-4.60, -1.81) |
| Guinea | 1031.88 (858.80, 1242.51) | 29.91 (24.86, 35.83) |  | 1881.15 (1308.12, 2447.14) | 32.17 (22.33, 41.90) |  | 82.30 | 0.39 (0.33, 0.45) |
| Guinea-Bissau | 35.31 (25.07, 50.78) | 8.07 (5.85, 11.34) |  | 47.89 (34.06, 66.43) | 6.12 (4.45, 8.28) |  | 35.63 | -1.07 (-1.14, -1.00) |
| Guyana | 25.88 (22.23, 29.57) | 6.52 (5.61, 7.45) |  | 19.02 (14.62, 24.35) | 3.03 (2.36, 3.84) |  | -26.52 | -2.90 (-3.72, -2.07) |
| Haiti | 219.70 (136.77, 300.00) | 6.65 (4.16, 9.25) |  | 289.92 (175.81, 431.87) | 4.14 (2.49, 6.26) |  | 31.97 | -1.74 (-1.97, -1.52) |
| Honduras | 231.32 (91.63, 333.40) | 11.06 (4.16, 16.11) |  | 879.29 (407.57, 1299.01) | 14.80 (6.80, 21.70) |  | 280.12 | 1.22 (1.10, 1.35) |
| Hungary | 951.97 (909.20, 992.65) | 6.47 (6.18, 6.74) |  | 486.03 (399.92, 592.11) | 2.58 (2.11, 3.16) |  | -48.94 | -2.42 (-3.14, -1.70) |
| Iceland | 5.69 (5.20, 6.25) | 2.03 (1.86, 2.23) |  | 18.74 (16.21, 21.58) | 3.52 (3.06, 4.05) |  | 229.48 | 1.82 (1.70, 1.94) |
| India | 11776.65 (9852.31, 13688.90) | 2.54 (2.09, 2.97) |  | 29967.57 (25243.79, 35612.30) | 2.61 (2.20, 3.10) |  | 154.47 | 0.13 (0.05, 0.21) |
| Indonesia | 2472.02 (2136.55, 2836.51) | 2.43 (2.10, 2.80) |  | 4504.14 (3796.46, 5157.77) | 2.23 (1.90, 2.51) |  | 82.20 | -0.47 (-0.56, -0.37) |
| Iran | 1067.70 (881.37, 1245.36) | 4.26 (3.45, 5.11) |  | 2721.52 (2474.84, 2991.34) | 3.85 (3.50, 4.25) |  | 154.90 | -0.83 (-1.50, -0.15) |
| Iraq | 353.23 (272.92, 439.12) | 4.38 (3.37, 5.43) |  | 1406.61 (1068.91, 1782.50) | 6.09 (4.71, 7.57) |  | 298.21 | 1.35 (0.91, 1.79) |
| Ireland | 60.09 (55.90, 64.33) | 1.46 (1.36, 1.56) |  | 295.26 (227.32, 374.31) | 4.00 (3.07, 5.10) |  | 391.38 | 4.24 (3.89, 4.58) |
| Israel | 121.91 (109.07, 134.87) | 2.49 (2.23, 2.74) |  | 337.46 (263.12, 428.66) | 2.97 (2.31, 3.79) |  | 176.81 | 0.54 (0.44, 0.63) |
| Italy | 5982.34 (5811.08, 6129.91) | 6.77 (6.58, 6.94) |  | 7927.98 (6450.13, 9653.50) | 5.97 (4.81, 7.37) |  | 32.52 | -0.85 (-1.06, -0.64) |
| Jamaica | 69.75 (65.28, 74.63) | 3.89 (3.64, 4.15) |  | 77.03 (60.96, 96.31) | 2.61 (2.07, 3.25) |  | 10.44 | -0.45 (-1.39, 0.50) |
| Japan | 24465.36 (23600.80, 25148.05) | 14.22 (13.71, 14.62) |  | 46659.56 (37994.64, 55120.07) | 12.71 (10.51, 14.98) |  | 90.72 | -1.24 (-1.74, -0.73) |
| Jordan | 40.08 (31.83, 49.48) | 3.01 (2.36, 3.78) |  | 154.27 (124.47, 191.27) | 2.46 (1.98, 3.04) |  | 284.88 | -0.91 (-1.04, -0.78) |
| Kazakhstan | 450.19 (419.32, 482.17) | 3.48 (3.24, 3.71) |  | 1100.68 (946.15, 1272.44) | 6.30 (5.43, 7.24) |  | 144.49 | -1.35 (-2.49, -0.21) |
| Kenya | 218.69 (151.29, 383.95) | 2.48 (1.70, 4.37) |  | 663.49 (431.64, 982.39) | 2.90 (1.92, 4.25) |  | 203.39 | -0.15 (-0.55, 0.26) |
| Kiribati | 5.83 (4.50, 7.34) | 13.92 (10.80, 17.27) |  | 9.23 (7.12, 11.78) | 11.69 (9.09, 14.66) |  | 58.39 | -0.63 (-0.67, -0.60) |
| Kuwait | 18.25 (16.32, 20.34) | 2.78 (2.47, 3.10) |  | 65.83 (51.80, 82.36) | 2.75 (2.17, 3.42) |  | 260.79 | 0.58 (0.33, 0.84) |
| Kyrgyzstan | 40.23 (36.27, 44.59) | 1.27 (1.16, 1.38) |  | 114.29 (97.00, 132.30) | 2.54 (2.16, 2.91) |  | 184.09 | 3.14 (2.81, 3.47) |
| Lao | 219.65 (150.34, 299.84) | 9.99 (6.95, 13.51) |  | 304.68 (223.29, 395.43) | 6.72 (5.06, 8.54) |  | 38.71 | -1.63 (-1.75, -1.51) |
| Latvia | 58.04 (54.24, 62.06) | 1.65 (1.54, 1.77) |  | 101.05 (83.59, 121.95) | 2.68 (2.22, 3.26) |  | 74.10 | 0.76 (0.01, 1.51) |
| Lebanon | 78.87 (64.02, 97.33) | 3.43 (2.84, 4.20) |  | 191.92 (143.13, 265.83) | 3.67 (2.74, 5.09) |  | 143.35 | 0.28 (0.12, 0.44) |
| Lesotho | 51.92 (31.53, 110.07) | 5.13 (3.17, 10.64) |  | 194.85 (77.80, 308.83) | 14.44 (6.05, 22.70) |  | 275.27 | 3.98 (3.27, 4.69) |
| Liberia | 77.56 (61.09, 100.76) | 6.78 (5.37, 8.83) |  | 106.68 (75.10, 154.39) | 4.92 (3.60, 7.24) |  | 37.54 | -1.28 (-1.42, -1.14) |
| Libya | 100.02 (75.74, 129.52) | 5.18 (3.90, 6.71) |  | 270.61 (199.76, 373.77) | 5.18 (3.88, 7.05) |  | 170.55 | 0.08 (-0.05, 0.22) |
| Lithuania | 73.96 (69.38, 78.67) | 1.66 (1.56, 1.78) |  | 172.24 (135.71, 211.01) | 3.20 (2.50, 3.95) |  | 132.90 | 1.43 (1.12, 1.74) |
| Luxembourg | 15.99 (14.77, 17.34) | 2.95 (2.73, 3.20) |  | 39.21 (30.36, 51.20) | 4.03 (3.14, 5.25) |  | 145.20 | 1.12 (0.82, 1.41) |
| Macedonia | 162.88 (145.88, 178.88) | 8.65 (7.72, 9.49) |  | 270.29 (213.45, 344.42) | 8.39 (6.65, 10.65) |  | 65.94 | -0.23 (-0.35, -0.11) |
| Madagascar | 163.71 (117.69, 274.22) | 2.88 (1.99, 5.02) |  | 285.28 (192.94, 429.79) | 2.44 (1.66, 3.72) |  | 74.25 | -0.84 (-0.95, -0.74) |
| Malawi | 160.34 (118.67, 221.59) | 3.25 (2.46, 4.60) |  | 240.78 (187.37, 303.07) | 2.82 (2.23, 3.54) |  | 50.17 | -1.45 (-1.80, -1.09) |
| Malaysia | 523.27 (448.16, 611.98) | 5.51 (4.70, 6.44) |  | 1641.04 (1243.31, 2134.11) | 6.08 (4.65, 7.83) |  | 213.61 | 0.39 (0.15, 0.63) |
| Maldives | 6.00 (4.04, 9.26) | 6.80 (4.73, 10.09) |  | 16.85 (13.20, 20.68) | 5.47 (4.31, 6.70) |  | 180.77 | -0.65 (-0.81, -0.48) |
| Mali | 695.56 (570.71, 837.79) | 15.12 (12.43, 18.25) |  | 1358.18 (996.92, 1802.93) | 14.46 (10.75, 18.98) |  | 95.26 | -0.34 (-0.44, -0.25) |
| Malta | 6.88 (6.23, 7.55) | 1.61 (1.46, 1.76) |  | 22.18 (18.70, 26.13) | 2.50 (2.11, 2.95) |  | 222.36 | 1.48 (1.27, 1.70) |
| Marshall Islands | 2.04 (1.58, 2.64) | 11.66 (8.89, 15.25) |  | 3.87 (2.72, 5.29) | 10.22 (7.38, 13.84) |  | 89.89 | -0.38 (-0.44, -0.32) |
| Mauritania | 72.21 (58.44, 88.41) | 6.91 (5.59, 8.42) |  | 86.47 (62.59, 113.61) | 4.10 (3.03, 5.29) |  | 19.75 | -1.81 (-1.96, -1.67) |
| Mauritius | 13.52 (12.50, 14.52) | 1.81 (1.68, 1.94) |  | 33.13 (25.42, 42.79) | 1.93 (1.49, 2.49) |  | 145.04 | 1.44 (1.02, 1.86) |
| Mexico | 969.85 (935.38, 997.29) | 2.24 (2.14, 2.30) |  | 3973.10 (3435.71, 4549.71) | 3.46 (2.99, 3.96) |  | 309.66 | 1.55 (1.40, 1.70) |
| Moldova | 73.24 (68.21, 78.26) | 1.65 (1.54, 1.76) |  | 131.51 (111.41, 154.68) | 2.30 (1.95, 2.70) |  | 79.57 | -1.06 (-2.66, 0.57) |
| Monaco | 2.30 (1.80, 2.84) | 3.47 (2.71, 4.29) |  | 8.04 (6.36, 9.89) | 9.04 (7.13, 11.28) |  | 249.19 | 3.97 (3.11, 4.83) |
| Mongolia | 679.84 (543.82, 826.85) | 64.22 (51.72, 77.51) |  | 2311.05 (1766.56, 2994.15) | 105.22 (82.57, 131.46) |  | 239.94 | 2.39 (1.93, 2.86) |
| Montenegro | 37.89 (31.20, 43.92) | 6.05 (4.99, 7.00) |  | 58.42 (46.87, 71.61) | 5.97 (4.82, 7.31) |  | 54.20 | -0.13 (-0.34, 0.09) |
| Morocco | 249.05 (183.33, 306.67) | 1.92 (1.38, 2.37) |  | 631.62 (480.99, 777.89) | 2.16 (1.65, 2.63) |  | 153.61 | 0.24 (-0.00, 0.48) |
| Mozambique | 132.73 (94.26, 214.36) | 2.10 (1.51, 3.39) |  | 440.58 (303.25, 585.03) | 3.67 (2.55, 4.85) |  | 231.94 | 1.87 (1.65, 2.09) |
| Myanmar | 643.20 (457.62, 925.27) | 2.78 (2.01, 3.95) |  | 1868.01 (1542.68, 2236.98) | 4.11 (3.46, 4.88) |  | 190.42 | 1.28 (1.14, 1.42) |
| Namibia | 13.82 (8.48, 24.75) | 1.86 (1.16, 3.30) |  | 48.06 (35.56, 63.15) | 3.28 (2.50, 4.20) |  | 247.88 | 2.02 (1.61, 2.44) |
| Nauru | 0.46 (0.31, 0.62) | 10.54 (7.58, 13.38) |  | 0.46 (0.31, 0.67) | 9.17 (6.50, 12.59) |  | 0.57 | -0.67 (-0.95, -0.39) |
| Nepal | 178.66 (137.11, 231.60) | 1.80 (1.40, 2.27) |  | 459.74 (331.23, 662.43) | 2.13 (1.54, 3.05) |  | 157.33 | 0.63 (0.51, 0.76) |
| Netherlands | 283.86 (268.58, 297.99) | 1.46 (1.39, 1.54) |  | 1074.16 (836.46, 1349.94) | 3.33 (2.58, 4.19) |  | 278.41 | 3.21 (3.05, 3.37) |
| New Zealand | 92.04 (85.44, 98.93) | 2.41 (2.24, 2.59) |  | 361.04 (296.67, 437.40) | 4.96 (4.09, 6.02) |  | 292.27 | 2.67 (2.50, 2.85) |
| Nicaragua | 49.90 (43.79, 56.35) | 3.04 (2.65, 3.48) |  | 164.65 (132.89, 202.41) | 3.79 (3.09, 4.63) |  | 229.97 | 0.77 (0.33, 1.22) |
| Niger | 20.13 (15.62, 25.16) | 0.67 (0.52, 0.83) |  | 51.19 (37.20, 68.40) | 0.61 (0.45, 0.80) |  | 154.32 | -0.44 (-0.52, -0.36) |
| Nigeria | 1559.30 (1183.76, 1976.12) | 3.30 (2.46, 4.16) |  | 2785.56 (2157.33, 3527.04) | 3.25 (2.56, 4.04) |  | 78.64 | 0.04 (-0.01, 0.08) |
| Niue | 0.17 (0.14, 0.22) | 8.10 (6.43, 10.14) |  | 0.16 (0.12, 0.20) | 7.37 (5.65, 9.46) |  | -8.56 | -0.44 (-0.48, -0.40) |
| North Korea | 2589.05 (1965.02, 3409.54) | 14.62 (11.35, 18.97) |  | 3352.93 (2492.28, 4401.89) | 10.23 (7.66, 13.36) |  | 29.50 | -1.47 (-1.58, -1.36) |
| Northern Mariana Islands | 1.48 (1.10, 1.92) | 6.80 (5.35, 8.40) |  | 4.56 (3.66, 5.72) | 7.97 (6.58, 9.70) |  | 207.97 | 0.82 (0.66, 0.98) |
| Norway | 105.50 (100.14, 109.93) | 1.64 (1.57, 1.71) |  | 261.98 (218.19, 315.68) | 2.91 (2.42, 3.51) |  | 148.33 | 2.43 (2.24, 2.61) |
| Oman | 30.80 (21.78, 41.42) | 4.25 (2.94, 5.65) |  | 98.16 (78.98, 121.59) | 5.27 (4.47, 6.30) |  | 218.70 | 1.52 (1.14, 1.91) |
| Pakistan | 1975.61 (1345.19, 2622.33) | 3.27 (2.16, 4.42) |  | 3883.60 (3101.33, 4736.40) | 3.27 (2.56, 4.02) |  | 96.58 | -0.05 (-0.21, 0.10) |
| Palau | 1.01 (0.69, 1.41) | 9.64 (6.66, 13.30) |  | 2.34 (1.77, 3.10) | 9.97 (7.68, 12.94) |  | 131.00 | 0.09 (0.02, 0.16) |
| Palestine | 71.99 (52.57, 96.13) | 8.42 (6.28, 11.14) |  | 148.94 (124.66, 180.32) | 6.56 (5.52, 7.88) |  | 106.90 | -0.89 (-0.97, -0.82) |
| Panama | 49.94 (45.98, 53.61) | 3.27 (3.01, 3.52) |  | 120.64 (91.65, 155.16) | 2.93 (2.23, 3.77) |  | 141.57 | -0.07 (-0.52, 0.38) |
| Papua New Guinea | 24.68 (18.99, 31.59) | 1.40 (1.09, 1.78) |  | 65.25 (49.80, 85.41) | 1.53 (1.19, 1.96) |  | 164.43 | 0.37 (0.34, 0.39) |
| Paraguay | 52.35 (45.38, 59.82) | 2.32 (2.00, 2.66) |  | 111.97 (83.92, 145.95) | 2.05 (1.54, 2.67) |  | 113.87 | -0.38 (-0.93, 0.17) |
| Peru | 718.84 (618.55, 828.90) | 5.84 (5.02, 6.72) |  | 831.07 (618.03, 1102.85) | 2.60 (1.93, 3.45) |  | 15.61 | -3.62 (-4.20, -3.04) |
| Philippines | 3166.65 (2421.57, 3894.91) | 9.64 (7.27, 11.92) |  | 5300.68 (4244.35, 6574.04) | 6.38 (5.16, 7.84) |  | 67.39 | -1.80 (-2.08, -1.52) |
| Poland | 3007.42 (2891.93, 3100.73) | 6.95 (6.66, 7.17) |  | 1348.50 (1131.27, 1599.32) | 1.95 (1.63, 2.31) |  | -55.16 | -4.18 (-5.51, -2.84) |
| Portugal | 254.57 (241.31, 269.38) | 1.86 (1.77, 1.96) |  | 1058.27 (825.34, 1339.01) | 4.90 (3.79, 6.26) |  | 315.72 | 3.06 (2.74, 3.38) |
| Puerto Rico | 240.56 (225.30, 253.79) | 6.57 (6.15, 6.93) |  | 188.41 (140.96, 243.66) | 2.76 (2.06, 3.59) |  | -21.68 | -1.88 (-2.55, -1.21) |
| Qatar | 14.61 (11.09, 19.18) | 16.14 (12.14, 21.04) |  | 108.14 (75.75, 146.58) | 17.39 (12.83, 22.91) |  | 640.09 | 0.85 (0.66, 1.03) |
| Romania | 483.28 (432.83, 542.58) | 1.75 (1.56, 1.95) |  | 1064.11 (865.39, 1294.33) | 3.02 (2.45, 3.70) |  | 120.18 | 2.68 (2.21, 3.15) |
| Russia | 3043.97 (2910.49, 3207.25) | 1.72 (1.64, 1.80) |  | 6597.54 (5591.15, 7845.07) | 2.91 (2.46, 3.44) |  | 116.74 | 2.21 (1.94, 2.48) |
| Rwanda | 158.18 (113.78, 219.56) | 5.05 (3.67, 6.79) |  | 279.23 (211.75, 368.76) | 4.40 (3.48, 5.65) |  | 76.53 | -1.19 (-1.43, -0.95) |
| Saint Kitts and Nevis | 4.75 (4.31, 5.25) | 12.71 (11.56, 14.03) |  | 2.45 (2.01, 2.95) | 3.73 (3.12, 4.44) |  | -48.35 | -4.70 (-5.77, -3.62) |
| Saint Lucia | 5.36 (4.92, 5.84) | 6.05 (5.55, 6.61) |  | 4.59 (3.82, 5.50) | 2.16 (1.80, 2.59) |  | -14.39 | -3.85 (-4.88, -2.80) |
| Saint Vincent and the Grenadines | 5.23 (4.81, 5.68) | 7.15 (6.57, 7.76) |  | 4.01 (3.45, 4.66) | 2.97 (2.57, 3.44) |  | -23.37 | -3.10 (-4.01, -2.18) |
| Samoa | 5.53 (4.32, 7.04) | 6.01 (4.76, 7.60) |  | 7.67 (5.78, 9.80) | 4.99 (3.82, 6.29) |  | 38.57 | -0.66 (-0.74, -0.59) |
| San Marino | 0.72 (0.60, 0.85) | 2.21 (1.85, 2.62) |  | 1.92 (1.46, 2.56) | 3.22 (2.45, 4.29) |  | 166.82 | 1.87 (1.67, 2.07) |
| Sao Tome and Principe | 1.61 (1.23, 1.96) | 2.44 (1.89, 2.96) |  | 3.01 (2.01, 4.04) | 2.64 (1.77, 3.48) |  | 86.49 | 0.24 (0.12, 0.35) |
| Saudi Arabia | 290.93 (211.77, 373.30) | 5.03 (3.66, 6.43) |  | 855.48 (644.99, 1124.01) | 5.29 (4.17, 6.69) |  | 194.05 | -0.00 (-0.21, 0.21) |
| Senegal | 76.87 (61.06, 91.65) | 2.23 (1.78, 2.65) |  | 162.90 (120.07, 207.98) | 2.07 (1.55, 2.60) |  | 111.92 | -0.12 (-0.26, 0.01) |
| Serbia | 681.71 (568.36, 789.31) | 5.99 (5.00, 6.87) |  | 862.30 (677.58, 1086.55) | 5.42 (4.28, 6.82) |  | 26.49 | -0.64 (-0.91, -0.37) |
| Seychelles | 4.33 (3.76, 5.01) | 7.69 (6.67, 8.90) |  | 6.22 (5.18, 7.42) | 5.56 (4.68, 6.58) |  | 43.69 | -0.97 (-1.23, -0.71) |
| Sierra Leone | 115.99 (87.01, 149.87) | 5.83 (4.43, 7.54) |  | 176.06 (130.26, 234.99) | 4.62 (3.50, 6.03) |  | 51.79 | -0.87 (-0.95, -0.78) |
| Singapore | 189.49 (178.05, 201.44) | 8.44 (7.90, 8.99) |  | 881.22 (707.07, 1109.76) | 11.50 (9.24, 14.41) |  | 365.05 | 1.00 (0.74, 1.26) |
| Slovakia | 272.13 (255.47, 292.51) | 4.55 (4.27, 4.87) |  | 311.09 (242.14, 395.28) | 3.41 (2.66, 4.33) |  | 14.32 | -1.54 (-1.83, -1.26) |
| Slovenia | 71.19 (54.56, 90.60) | 2.91 (2.23, 3.72) |  | 223.27 (171.37, 285.79) | 5.30 (4.04, 6.83) |  | 213.60 | 2.38 (2.08, 2.68) |
| Solomon Islands | 10.96 (8.17, 14.23) | 6.67 (4.95, 8.63) |  | 19.37 (15.24, 24.13) | 5.37 (4.30, 6.55) |  | 76.62 | -0.74 (-0.82, -0.66) |
| Somalia | 98.30 (58.61, 170.34) | 3.53 (2.21, 6.12) |  | 240.03 (150.03, 425.16) | 3.33 (2.15, 5.91) |  | 144.17 | -0.13 (-0.20, -0.06) |
| South Africa | 1306.45 (866.02, 2211.49) | 5.79 (3.76, 9.81) |  | 2599.74 (2291.21, 2945.59) | 5.60 (4.94, 6.35) |  | 98.99 | -0.50 (-1.11, 0.11) |
| South Korea | 3547.65 (2935.49, 4338.57) | 10.99 (9.18, 13.42) |  | 20367.31 (16677.58, 24503.60) | 22.80 (18.72, 27.32) |  | 474.11 | 3.38 (2.06, 4.72) |
| South Sudan | 77.73 (48.71, 141.00) | 3.03 (1.88, 5.53) |  | 109.90 (64.64, 191.78) | 2.74 (1.64, 4.77) |  | 41.39 | -0.35 (-0.38, -0.31) |
| Spain | 2131.62 (2023.01, 2224.80) | 3.92 (3.72, 4.09) |  | 5411.04 (4206.62, 6880.68) | 6.01 (4.63, 7.72) |  | 153.85 | 1.33 (0.95, 1.72) |
| Sri Lanka | 222.06 (193.69, 254.28) | 2.06 (1.81, 2.35) |  | 711.03 (511.50, 953.33) | 2.80 (2.06, 3.72) |  | 220.19 | 1.96 (1.62, 2.30) |
| Sudan | 330.28 (194.85, 495.09) | 3.53 (2.06, 5.36) |  | 673.55 (392.97, 1047.68) | 3.70 (2.19, 5.76) |  | 103.93 | 0.15 (0.02, 0.28) |
| Suriname | 17.02 (15.10, 18.97) | 6.45 (5.76, 7.18) |  | 15.55 (12.45, 19.48) | 2.60 (2.09, 3.23) |  | -8.64 | -2.93 (-3.66, -2.20) |
| Sweden | 350.82 (331.68, 368.94) | 2.41 (2.29, 2.53) |  | 594.66 (501.22, 696.88) | 3.04 (2.56, 3.59) |  | 69.51 | 1.33 (0.72, 1.94) |
| Switzerland | 266.12 (250.68, 280.84) | 2.64 (2.48, 2.78) |  | 959.10 (737.60, 1244.19) | 5.79 (4.44, 7.59) |  | 260.40 | 2.02 (1.44, 2.59) |
| Syrian Arab Republic | 258.81 (195.65, 325.68) | 4.89 (3.64, 6.23) |  | 534.69 (400.38, 711.31) | 4.50 (3.42, 5.90) |  | 106.60 | -0.37 (-0.52, -0.22) |
| Taiwan (Province of China) | 2117.75 (2030.10, 2207.86) | 12.26 (11.74, 12.77) |  | 3356.16 (2609.39, 4335.45) | 8.65 (6.73, 11.15) |  | 58.48 | -2.23 (-3.33, -1.11) |
| Tajikistan | 33.72 (30.35, 38.31) | 1.04 (0.94, 1.15) |  | 173.92 (138.91, 219.34) | 3.70 (2.96, 4.66) |  | 415.83 | 5.12 (4.88, 5.36) |
| Tanzania | 254.64 (202.91, 313.91) | 2.03 (1.67, 2.48) |  | 600.00 (464.25, 764.55) | 2.25 (1.79, 2.80) |  | 135.63 | 0.30 (0.18, 0.42) |
| Thailand | 7868.96 (6829.90, 9009.17) | 20.81 (18.13, 23.74) |  | 24828.49 (18286.70, 33054.99) | 24.18 (17.89, 32.01) |  | 215.52 | 0.61 (0.51, 0.70) |
| Timor-Leste | 23.21 (15.20, 32.86) | 7.47 (5.02, 10.41) |  | 50.88 (33.49, 71.95) | 6.19 (4.18, 8.57) |  | 119.22 | -0.88 (-1.05, -0.70) |
| Togo | 86.55 (68.43, 110.48) | 6.40 (5.11, 8.12) |  | 191.83 (144.41, 251.86) | 4.95 (3.83, 6.37) |  | 121.65 | -1.33 (-1.49, -1.17) |
| Tokelau | 0.11 (0.08, 0.15) | 8.36 (6.20, 10.95) |  | 0.10 (0.07, 0.13) | 7.32 (5.29, 9.90) |  | -12.79 | -0.54 (-0.57, -0.50) |
| Tonga | 13.65 (9.45, 17.84) | 23.33 (16.20, 30.14) |  | 19.66 (14.22, 25.73) | 24.33 (17.65, 31.90) |  | 44.02 | 0.10 (-0.03, 0.22) |
| Trinidad and Tobago | 56.53 (53.01, 60.10) | 6.66 (6.23, 7.09) |  | 46.16 (34.78, 60.51) | 2.51 (1.90, 3.29) |  | -18.33 | -3.50 (-4.54, -2.44) |
| Tunisia | 94.33 (72.87, 120.54) | 1.87 (1.45, 2.37) |  | 240.07 (170.14, 335.89) | 1.94 (1.38, 2.69) |  | 154.51 | 0.27 (0.18, 0.35) |
| Turkey | 1349.21 (1079.85, 1640.10) | 3.72 (2.97, 4.47) |  | 2767.82 (2204.34, 3413.37) | 3.21 (2.55, 3.95) |  | 105.14 | -0.52 (-0.78, -0.26) |
| Turkmenistan | 25.38 (23.36, 28.83) | 1.17 (1.09, 1.27) |  | 234.46 (183.91, 297.99) | 5.55 (4.38, 7.01) |  | 823.81 | 6.35 (5.25, 7.45) |
| Tuvalu | 0.74 (0.55, 0.95) | 10.40 (7.88, 13.47) |  | 0.88 (0.65, 1.19) | 8.47 (6.24, 11.32) |  | 19.28 | -0.80 (-0.93, -0.67) |
| Uganda | 317.50 (255.04, 387.75) | 4.48 (3.60, 5.42) |  | 962.52 (744.42, 1217.45) | 6.06 (4.81, 7.51) |  | 203.15 | 1.12 (0.90, 1.34) |
| Ukraine | 685.04 (650.68, 720.33) | 1.00 (0.94, 1.07) |  | 1929.62 (1640.63, 2284.99) | 2.67 (2.27, 3.19) |  | 181.68 | 4.25 (3.68, 4.82) |
| United Arab Emirates | 20.51 (11.10, 36.50) | 4.28 (2.15, 8.14) |  | 219.40 (92.71, 496.49) | 4.73 (2.01, 10.82) |  | 969.60 | 0.19 (-0.06, 0.45) |
| United Kingdom | 1787.33 (1722.06, 1835.84) | 2.04 (1.97, 2.10) |  | 6201.49 (5143.40, 7417.01) | 5.08 (4.20, 6.11) |  | 246.97 | 3.95 (3.72, 4.19) |
| United States of America | 6874.50 (6639.15, 7028.71) | 2.22 (2.15, 2.27) |  | 27895.01 (22784.69, 33512.92) | 5.23 (4.28, 6.29) |  | 305.78 | 2.97 (2.74, 3.19) |
| United States Virgin Islands | 3.49 (2.87, 4.11) | 4.07 (3.35, 4.76) |  | 4.61 (3.81, 5.45) | 2.49 (2.06, 2.95) |  | 32.11 | -1.78 (-2.39, -1.16) |
| Uruguay | 53.07 (46.65, 59.82) | 1.36 (1.20, 1.53) |  | 120.13 (93.79, 152.59) | 2.28 (1.77, 2.90) |  | 126.36 | 2.15 (2.00, 2.31) |
| Uzbekistan | 114.69 (99.29, 134.44) | 0.91 (0.82, 1.00) |  | 1345.91 (1098.10, 1614.72) | 6.34 (5.27, 7.46) |  | 1073.50 | 9.05 (7.82, 10.28) |
| Vanuatu | 6.41 (4.13, 9.63) | 9.22 (5.88, 13.77) |  | 16.50 (11.16, 23.46) | 9.05 (6.21, 12.85) |  | 157.34 | -0.03 (-0.11, 0.06) |
| Venezuela | 617.31 (588.47, 646.15) | 6.36 (6.04, 6.68) |  | 653.76 (501.85, 855.23) | 2.29 (1.77, 2.97) |  | 5.90 | -2.80 (-3.96, -1.64) |
| Viet Nam | 1499.65 (1119.01, 1892.91) | 3.70 (2.77, 4.63) |  | 2335.33 (1786.64, 2945.04) | 2.61 (2.03, 3.26) |  | 55.72 | -1.47 (-1.67, -1.27) |
| Yemen | 155.61 (98.42, 229.18) | 3.19 (2.03, 4.67) |  | 415.82 (300.68, 568.50) | 3.20 (2.32, 4.33) |  | 167.22 | 0.05 (-0.04, 0.14) |
| Zambia | 75.99 (56.45, 108.09) | 2.37 (1.78, 3.43) |  | 195.43 (149.30, 246.95) | 2.75 (2.14, 3.41) |  | 157.16 | -0.18 (-0.55, 0.19) |
| Zimbabwe | 512.13 (397.42, 796.21) | 11.76 (9.21, 17.91) |  | 1031.93 (760.18, 1405.34) | 13.64 (10.27, 18.13) |  | 101.50 | -0.65 (-1.19, -0.11) |

ASIR: age-standardized incidence rate; CI: confidence interval; EAPC: estimated annual percentage change; UI: uncertainty interval.

**Table S13**. **The incident cases and ASIR of primary liver cancer due to hepatitis B in 1990 and 2019 and their change trends from 1990 to 2019 at national level.**

| Nation | 1990 | |  | 2019 | |  | 1990-2019 | |
| --- | --- | --- | --- | --- | --- | --- | --- | --- |
|  | Incident cases  No. (95% UI) | ASIR per 100,000  No. (95% UI) |  | Incident cases  No. x 10^3^ (95% UI) | ASIR per 100,000  No. (95% UI) |  | Percentage change in incident cases  No. (%) | EAPC in ASIR  No. (95% CI) |
| Afghanistan | 339.40 (236.62, 472.88) | 4.41 (3.12, 6.09) |  | 605.86 (424.39, 850.48) | 3.60 (2.49, 4.97) |  | 78.51 | -0.74 (-0.85, -0.63) |
| Albania | 70.49 (52.34, 92.39) | 3.15 (2.29, 4.16) |  | 67.89 (42.46, 102.09) | 1.66 (1.06, 2.45) |  | -3.69 | -3.26 (-3.84, -2.68) |
| Algeria | 79.12 (56.67, 106.04) | 0.59 (0.42, 0.80) |  | 251.80 (175.69, 350.14) | 0.71 (0.49, 0.99) |  | 218.26 | 0.60 (0.39, 0.81) |
| American Samoa | 0.76 (0.52, 1.00) | 2.83 (1.96, 3.74) |  | 1.88 (1.42, 2.48) | 3.64 (2.78, 4.75) |  | 148.34 | 1.26 (1.11, 1.42) |
| Andorra | 0.76 (0.45, 1.17) | 1.30 (0.80, 2.00) |  | 1.94 (1.21, 3.01) | 1.46 (0.92, 2.22) |  | 156.70 | 0.39 (0.33, 0.44) |
| Angola | 31.93 (21.40, 46.19) | 0.64 (0.42, 0.93) |  | 79.76 (53.40, 114.96) | 0.54 (0.36, 0.78) |  | 149.80 | -0.73 (-0.84, -0.63) |
| Antigua and Barbuda | 1.24 (0.90, 1.66) | 2.42 (1.76, 3.25) |  | 0.72 (0.50, 1.01) | 0.69 (0.48, 0.96) |  | -42.21 | -4.75 (-5.76, -3.73) |
| Argentina | 81.12 (55.75, 115.05) | 0.25 (0.17, 0.35) |  | 187.10 (121.68, 284.20) | 0.36 (0.23, 0.54) |  | 130.64 | 1.83 (1.64, 2.01) |
| Armenia | 7.15 (5.32, 9.33) | 0.25 (0.18, 0.33) |  | 56.33 (37.34, 81.32) | 1.39 (0.93, 1.98) |  | 688.17 | 9.11 (7.55, 10.69) |
| Australia | 60.29 (42.26, 83.94) | 0.32 (0.22, 0.43) |  | 237.75 (148.20, 357.83) | 0.65 (0.41, 0.98) |  | 294.34 | 2.70 (2.36, 3.03) |
| Austria | 31.27 (21.32, 44.57) | 0.30 (0.21, 0.42) |  | 62.40 (38.44, 97.30) | 0.41 (0.26, 0.62) |  | 99.53 | 1.13 (0.74, 1.53) |
| Azerbaijan | 12.74 (9.43, 16.87) | 0.23 (0.17, 0.31) |  | 93.98 (62.51, 138.17) | 0.92 (0.61, 1.36) |  | 637.43 | 5.91 (3.77, 8.08) |
| Bahamas | 4.18 (3.16, 5.50) | 2.41 (1.78, 3.25) |  | 3.73 (2.64, 5.19) | 0.89 (0.63, 1.24) |  | -10.90 | -3.73 (-4.58, -2.88) |
| Bahrain | 3.55 (2.64, 4.72) | 1.80 (1.29, 2.49) |  | 16.56 (11.35, 23.68) | 1.50 (1.03, 2.16) |  | 366.89 | -1.70 (-2.15, -1.25) |
| Bangladesh | 564.18 (404.06, 761.81) | 0.87 (0.62, 1.18) |  | 808.66 (566.42, 1142.39) | 0.57 (0.39, 0.82) |  | 43.33 | -2.11 (-2.33, -1.89) |
| Barbados | 2.08 (1.31, 3.15) | 0.77 (0.49, 1.16) |  | 2.84 (1.97, 4.07) | 0.61 (0.43, 0.86) |  | 36.93 | -1.13 (-1.36, -0.90) |
| Belarus | 37.46 (27.28, 50.38) | 0.29 (0.22, 0.39) |  | 76.56 (46.11, 120.35) | 0.52 (0.32, 0.80) |  | 104.40 | 2.65 (1.92, 3.39) |
| Belgium | 57.40 (39.65, 80.60) | 0.40 (0.28, 0.55) |  | 112.21 (71.15, 170.05) | 0.59 (0.37, 0.87) |  | 95.50 | 1.11 (0.89, 1.32) |
| Belize | 1.63 (1.23, 2.15) | 1.65 (1.21, 2.22) |  | 2.51 (1.85, 3.38) | 0.82 (0.59, 1.12) |  | 53.35 | -2.81 (-3.37, -2.24) |
| Benin | 71.62 (52.96, 95.45) | 3.31 (2.44, 4.44) |  | 121.69 (83.09, 169.77) | 2.15 (1.49, 3.00) |  | 69.91 | -1.85 (-2.01, -1.69) |
| Bermuda | 1.13 (0.82, 1.56) | 1.77 (1.29, 2.45) |  | 0.63 (0.43, 0.91) | 0.52 (0.36, 0.74) |  | -44.23 | -4.01 (-5.10, -2.91) |
| Bhutan | 1.93 (1.09, 3.15) | 0.63 (0.36, 1.03) |  | 4.44 (2.57, 6.97) | 0.74 (0.43, 1.15) |  | 129.80 | 0.48 (0.36, 0.60) |
| Bolivia | 79.94 (56.85, 108.24) | 2.34 (1.66, 3.18) |  | 175.98 (120.52, 245.53) | 1.96 (1.34, 2.71) |  | 120.15 | -0.80 (-0.89, -0.70) |
| Bosnia and Herzegovina | 70.27 (50.67, 94.37) | 1.57 (1.14, 2.09) |  | 97.18 (62.97, 147.13) | 1.67 (1.11, 2.50) |  | 38.30 | 0.41 (0.13, 0.70) |
| Botswana | 2.35 (0.98, 6.30) | 0.35 (0.14, 0.92) |  | 9.17 (5.92, 13.74) | 0.52 (0.34, 0.76) |  | 291.09 | 0.31 (-0.58, 1.20) |
| Brazil | 413.29 (357.56, 473.82) | 0.41 (0.35, 0.47) |  | 1008.03 (853.80, 1187.51) | 0.42 (0.35, 0.49) |  | 143.90 | 0.36 (0.23, 0.49) |
| Brunei | 5.19 (3.90, 6.70) | 4.38 (3.21, 5.78) |  | 20.29 (15.18, 26.27) | 5.29 (3.96, 6.89) |  | 290.65 | 1.57 (1.10, 2.04) |
| Bulgaria | 198.17 (140.34, 270.25) | 1.60 (1.17, 2.13) |  | 125.00 (82.74, 187.69) | 1.02 (0.68, 1.52) |  | -36.92 | -1.11 (-1.52, -0.69) |
| Burkina Faso | 59.46 (42.59, 78.49) | 1.25 (0.89, 1.66) |  | 91.75 (64.35, 124.99) | 0.84 (0.59, 1.17) |  | 54.29 | -1.52 (-1.67, -1.36) |
| Burundi | 27.00 (17.97, 38.38) | 1.00 (0.66, 1.44) |  | 43.77 (26.37, 73.70) | 0.78 (0.45, 1.30) |  | 62.11 | -1.46 (-1.65, -1.27) |
| Cambodia | 241.49 (181.34, 315.71) | 4.00 (2.95, 5.38) |  | 409.65 (291.24, 573.15) | 2.99 (2.11, 4.16) |  | 69.64 | -1.26 (-1.38, -1.14) |
| Cameroon | 15.80 (10.15, 24.75) | 0.32 (0.21, 0.50) |  | 43.20 (28.53, 61.91) | 0.30 (0.20, 0.43) |  | 173.48 | -0.43 (-0.73, -0.13) |
| Canada | 55.18 (38.44, 79.47) | 0.17 (0.12, 0.25) |  | 214.30 (130.94, 333.44) | 0.36 (0.23, 0.55) |  | 288.35 | 2.87 (2.69, 3.06) |
| Cape Verde | 1.19 (0.92, 1.52) | 0.52 (0.40, 0.65) |  | 24.35 (18.47, 31.42) | 5.27 (3.95, 6.83) |  | 1941.05 | 6.21 (3.88, 8.59) |
| Central African Republic | 14.64 (9.03, 21.66) | 0.99 (0.61, 1.48) |  | 22.49 (12.73, 37.18) | 0.81 (0.46, 1.31) |  | 53.68 | -1.15 (-1.41, -0.88) |
| Chad | 96.51 (67.95, 129.58) | 3.20 (2.26, 4.35) |  | 168.83 (118.58, 229.05) | 2.66 (1.87, 3.63) |  | 74.94 | -0.79 (-0.87, -0.71) |
| Chile | 44.89 (32.03, 62.69) | 0.43 (0.30, 0.59) |  | 118.46 (74.65, 181.73) | 0.50 (0.32, 0.76) |  | 163.90 | 1.13 (0.85, 1.41) |
| China | 158410.02 (129498.89, 191318.15) | 16.48 (13.52, 19.86) |  | 135027.64 (108923.81, 164319.86) | 6.63 (5.36, 8.07) |  | -14.76 | -4.75 (-5.59, -3.91) |
| Colombia | 114.74 (86.39, 151.70) | 0.57 (0.42, 0.78) |  | 232.44 (146.02, 349.03) | 0.44 (0.28, 0.67) |  | 102.59 | -0.77 (-1.24, -0.30) |
| Comoros | 2.40 (1.01, 4.52) | 1.00 (0.45, 1.85) |  | 4.35 (2.47, 7.58) | 0.82 (0.47, 1.42) |  | 80.94 | -0.97 (-1.16, -0.77) |
| Congo | 13.09 (8.40, 19.84) | 1.00 (0.63, 1.53) |  | 20.85 (13.46, 32.01) | 0.65 (0.41, 0.99) |  | 59.23 | -1.89 (-2.05, -1.74) |
| Cook Islands | 0.96 (0.72, 1.25) | 6.94 (5.22, 9.07) |  | 1.32 (0.98, 1.77) | 5.50 (4.07, 7.32) |  | 38.57 | -0.79 (-0.99, -0.59) |
| Costa Rica | 17.08 (12.60, 22.95) | 0.89 (0.64, 1.23) |  | 40.26 (26.05, 61.35) | 0.78 (0.50, 1.18) |  | 135.66 | -0.81 (-1.31, -0.31) |
| Cote d'Ivoire | 189.71 (132.78, 254.45) | 3.93 (2.82, 5.26) |  | 279.23 (185.84, 398.84) | 2.21 (1.49, 3.19) |  | 47.19 | -2.96 (-3.29, -2.61) |
| Croatia | 47.65 (33.91, 64.15) | 0.74 (0.53, 0.99) |  | 64.89 (41.81, 97.32) | 0.82 (0.54, 1.22) |  | 36.19 | 0.78 (0.42, 1.13) |
| Cuba | 186.42 (139.51, 244.55) | 1.80 (1.34, 2.35) |  | 107.11 (72.56, 155.07) | 0.58 (0.40, 0.84) |  | -42.54 | -4.25 (-5.58, -2.91) |
| Cyprus | 3.76 (2.46, 5.53) | 0.46 (0.31, 0.67) |  | 8.68 (5.85, 12.78) | 0.47 (0.32, 0.67) |  | 131.10 | 0.20 (-0.02, 0.42) |
| Czech Republic | 130.90 (92.60, 181.46) | 0.97 (0.70, 1.34) |  | 107.63 (70.65, 162.15) | 0.56 (0.38, 0.82) |  | -17.77 | -2.05 (-2.38, -1.72) |
| Democratic Republic of the Congo | 122.13 (84.80, 170.07) | 0.61 (0.42, 0.87) |  | 238.87 (156.09, 355.15) | 0.50 (0.32, 0.76) |  | 95.59 | -0.75 (-0.87, -0.62) |
| Denmark | 24.97 (16.95, 35.94) | 0.34 (0.24, 0.48) |  | 58.95 (35.99, 91.27) | 0.61 (0.38, 0.93) |  | 136.09 | 2.37 (2.13, 2.60) |
| Djibouti | 1.86 (1.01, 3.63) | 0.99 (0.54, 1.93) |  | 7.00 (3.75, 12.45) | 0.96 (0.53, 1.69) |  | 275.75 | -0.23 (-0.40, -0.06) |
| Dominica | 1.64 (1.17, 2.27) | 2.45 (1.78, 3.30) |  | 0.73 (0.49, 1.03) | 0.83 (0.56, 1.18) |  | -55.62 | -3.77 (-4.53, -2.99) |
| Dominican Republic | 50.76 (38.40, 65.65) | 1.17 (0.87, 1.54) |  | 127.38 (74.12, 214.61) | 1.30 (0.77, 2.18) |  | 150.93 | 0.51 (-0.04, 1.06) |
| Ecuador | 76.60 (61.92, 92.63) | 1.35 (1.07, 1.65) |  | 215.23 (152.18, 291.26) | 1.42 (1.00, 1.92) |  | 180.98 | 0.52 (0.24, 0.81) |
| Egypt | 789.66 (528.41, 1154.63) | 2.29 (1.52, 3.39) |  | 1879.54 (1117.56, 3027.91) | 2.49 (1.50, 4.03) |  | 138.02 | 0.81 (0.52, 1.10) |
| El Salvador | 20.64 (15.23, 27.50) | 0.63 (0.45, 0.86) |  | 18.01 (11.33, 27.42) | 0.31 (0.19, 0.47) |  | -12.76 | -2.78 (-3.45, -2.11) |
| Equatorial Guinea | 1.89 (1.23, 2.81) | 0.78 (0.52, 1.17) |  | 4.79 (2.55, 7.70) | 0.75 (0.40, 1.22) |  | 153.90 | -0.30 (-0.52, -0.07) |
| Eritrea | 12.88 (7.99, 20.15) | 1.01 (0.62, 1.59) |  | 29.16 (17.35, 45.49) | 0.86 (0.52, 1.35) |  | 126.42 | -0.99 (-1.18, -0.81) |
| Estonia | 10.35 (7.40, 14.24) | 0.52 (0.37, 0.70) |  | 16.67 (10.55, 24.52) | 0.73 (0.47, 1.08) |  | 61.03 | -0.27 (-0.85, 0.31) |
| Eswatini | 7.46 (4.16, 16.13) | 2.08 (1.14, 4.44) |  | 54.43 (12.76, 105.40) | 7.51 (1.83, 14.25) |  | 629.84 | 5.33 (3.87, 6.81) |
| Ethiopia | 157.35 (103.98, 238.00) | 0.66 (0.44, 0.96) |  | 276.62 (206.50, 369.94) | 0.58 (0.43, 0.78) |  | 75.80 | -0.63 (-0.74, -0.53) |
| Federated States of Micronesia | 3.12 (2.14, 4.26) | 5.93 (4.06, 8.21) |  | 4.46 (2.72, 6.77) | 5.35 (3.33, 7.86) |  | 43.18 | -0.40 (-0.57, -0.23) |
| Fiji | 12.61 (9.46, 16.36) | 2.93 (2.16, 3.83) |  | 24.90 (17.75, 34.68) | 2.96 (2.12, 4.08) |  | 97.45 | 0.64 (0.19, 1.11) |
| Finland | 33.02 (23.01, 47.06) | 0.49 (0.34, 0.68) |  | 79.30 (48.82, 125.69) | 0.76 (0.49, 1.17) |  | 140.18 | 1.86 (1.72, 2.00) |
| France | 555.14 (375.47, 785.13) | 0.74 (0.51, 1.03) |  | 1058.33 (646.27, 1654.99) | 0.96 (0.60, 1.47) |  | 90.64 | 0.70 (0.45, 0.94) |
| Gabon | 4.59 (2.96, 6.60) | 0.72 (0.46, 1.03) |  | 8.24 (4.77, 13.13) | 0.67 (0.39, 1.09) |  | 79.69 | -0.39 (-0.57, -0.20) |
| Gambia | 72.01 (50.79, 98.68) | 17.02 (12.08, 23.08) |  | 228.92 (159.30, 323.36) | 20.27 (14.09, 28.56) |  | 217.91 | 0.19 (0.03, 0.36) |
| Georgia | 16.27 (11.57, 22.48) | 0.26 (0.19, 0.36) |  | 46.64 (31.61, 67.22) | 0.89 (0.62, 1.28) |  | 186.67 | 3.30 (1.85, 4.77) |
| Germany | 386.60 (277.01, 529.48) | 0.33 (0.24, 0.45) |  | 887.99 (556.45, 1357.75) | 0.55 (0.35, 0.83) |  | 129.69 | 1.65 (1.18, 2.12) |
| Ghana | 214.78 (154.33, 297.40) | 3.01 (2.15, 4.13) |  | 514.75 (359.32, 699.11) | 2.77 (1.94, 3.78) |  | 139.67 | -0.60 (-0.84, -0.36) |
| Greece | 121.29 (90.67, 153.62) | 0.81 (0.62, 1.01) |  | 269.50 (183.34, 381.02) | 1.31 (0.92, 1.84) |  | 122.20 | 1.86 (1.72, 2.00) |
| Greenland | 0.28 (0.19, 0.41) | 0.62 (0.42, 0.94) |  | 0.58 (0.35, 0.89) | 0.78 (0.48, 1.18) |  | 108.19 | 0.94 (0.87, 1.01) |
| Grenada | 1.73 (1.28, 2.31) | 2.57 (1.91, 3.42) |  | 0.86 (0.60, 1.19) | 0.74 (0.52, 1.02) |  | -50.32 | -4.64 (-5.62, -3.64) |
| Guam | 1.78 (1.40, 2.24) | 1.98 (1.56, 2.53) |  | 6.74 (5.11, 8.67) | 3.49 (2.66, 4.45) |  | 279.52 | 2.61 (2.42, 2.80) |
| Guatemala | 74.08 (53.92, 100.36) | 1.73 (1.21, 2.39) |  | 89.65 (59.55, 131.00) | 0.74 (0.48, 1.10) |  | 21.01 | -3.63 (-4.96, -2.29) |
| Guinea | 567.50 (433.86, 731.18) | 16.25 (12.38, 20.89) |  | 1037.57 (690.36, 1420.30) | 17.00 (11.25, 23.24) |  | 82.83 | 0.33 (0.24, 0.42) |
| Guinea-Bissau | 19.42 (13.12, 28.53) | 4.23 (2.86, 6.06) |  | 25.12 (17.09, 36.11) | 2.90 (1.98, 4.08) |  | 29.36 | -1.37 (-1.45, -1.29) |
| Guyana | 8.07 (6.02, 10.77) | 1.84 (1.32, 2.52) |  | 5.58 (3.73, 8.09) | 0.82 (0.54, 1.18) |  | -30.85 | -3.02 (-3.77, -2.26) |
| Haiti | 68.61 (40.43, 107.97) | 1.91 (1.13, 3.04) |  | 92.16 (50.98, 153.24) | 1.19 (0.66, 2.02) |  | 34.32 | -1.74 (-1.98, -1.50) |
| Honduras | 51.10 (18.67, 84.59) | 2.21 (0.75, 3.78) |  | 157.66 (63.67, 272.78) | 2.46 (0.97, 4.31) |  | 208.52 | 0.35 (0.26, 0.44) |
| Hungary | 209.37 (148.28, 286.77) | 1.46 (1.06, 1.97) |  | 102.28 (68.63, 150.05) | 0.59 (0.40, 0.84) |  | -51.15 | -2.41 (-3.14, -1.66) |
| Iceland | 0.93 (0.66, 1.32) | 0.35 (0.24, 0.48) |  | 2.86 (1.98, 4.12) | 0.58 (0.40, 0.83) |  | 206.67 | 1.67 (1.56, 1.79) |
| India | 5032.10 (4035.67, 6058.52) | 0.99 (0.78, 1.19) |  | 10909.58 (8721.73, 13396.90) | 0.90 (0.72, 1.11) |  | 116.80 | -0.18 (-0.30, -0.05) |
| Indonesia | 603.81 (491.60, 720.92) | 0.50 (0.41, 0.60) |  | 993.35 (767.37, 1255.25) | 0.43 (0.33, 0.53) |  | 64.51 | -0.79 (-0.94, -0.63) |
| Iran | 466.28 (383.71, 549.69) | 1.67 (1.37, 1.98) |  | 1071.95 (918.11, 1244.85) | 1.42 (1.20, 1.66) |  | 129.89 | -1.08 (-1.74, -0.42) |
| Iraq | 135.95 (95.61, 187.19) | 1.59 (1.10, 2.22) |  | 524.67 (359.18, 744.68) | 2.04 (1.38, 2.88) |  | 285.94 | 0.99 (0.60, 1.38) |
| Ireland | 8.13 (5.61, 11.59) | 0.21 (0.14, 0.29) |  | 37.39 (23.36, 57.68) | 0.54 (0.34, 0.82) |  | 360.19 | 4.27 (3.88, 4.66) |
| Israel | 19.71 (13.62, 28.94) | 0.41 (0.29, 0.59) |  | 50.76 (32.62, 78.36) | 0.48 (0.31, 0.73) |  | 157.53 | 0.31 (0.19, 0.43) |
| Italy | 760.70 (637.37, 905.86) | 0.91 (0.78, 1.08) |  | 848.65 (632.81, 1100.92) | 0.75 (0.56, 0.98) |  | 11.56 | -1.27 (-1.54, -0.99) |
| Jamaica | 20.48 (15.10, 26.87) | 1.17 (0.86, 1.53) |  | 21.08 (14.36, 29.52) | 0.71 (0.48, 1.00) |  | 2.94 | -0.76 (-1.70, 0.19) |
| Japan | 3751.84 (3265.76, 4325.05) | 2.20 (1.92, 2.52) |  | 4875.84 (3779.83, 6178.68) | 1.65 (1.30, 2.08) |  | 29.96 | -1.86 (-2.30, -1.41) |
| Jordan | 16.49 (12.08, 22.30) | 1.10 (0.78, 1.51) |  | 56.92 (40.14, 78.41) | 0.80 (0.56, 1.13) |  | 245.05 | -1.41 (-1.56, -1.25) |
| Kazakhstan | 119.48 (87.21, 160.88) | 0.88 (0.63, 1.18) |  | 264.24 (182.10, 375.85) | 1.42 (0.98, 1.99) |  | 121.17 | -2.17 (-3.42, -0.90) |
| Kenya | 63.93 (40.08, 114.19) | 0.66 (0.41, 1.17) |  | 200.11 (123.13, 306.02) | 0.75 (0.46, 1.16) |  | 213.03 | -0.35 (-0.84, 0.14) |
| Kiribati | 3.50 (2.56, 4.65) | 7.75 (5.67, 10.44) |  | 5.35 (3.86, 7.21) | 6.08 (4.38, 8.17) |  | 52.69 | -0.89 (-0.96, -0.83) |
| Kuwait | 8.09 (6.36, 10.16) | 1.05 (0.78, 1.37) |  | 24.05 (16.51, 33.68) | 0.84 (0.56, 1.22) |  | 197.18 | -0.27 (-0.52, -0.03) |
| Kyrgyzstan | 9.57 (6.99, 12.77) | 0.30 (0.21, 0.40) |  | 27.28 (19.01, 37.92) | 0.54 (0.37, 0.76) |  | 184.94 | 2.65 (2.26, 3.06) |
| Lao | 101.01 (62.97, 149.51) | 4.27 (2.70, 6.30) |  | 131.96 (88.52, 186.19) | 2.62 (1.76, 3.67) |  | 30.63 | -1.91 (-2.00, -1.83) |
| Latvia | 14.21 (10.16, 19.42) | 0.41 (0.30, 0.55) |  | 18.65 (12.39, 27.38) | 0.56 (0.38, 0.81) |  | 31.29 | -0.15 (-1.03, 0.74) |
| Lebanon | 42.61 (32.05, 55.17) | 1.78 (1.34, 2.28) |  | 92.05 (64.28, 131.82) | 1.76 (1.23, 2.52) |  | 116.01 | -0.02 (-0.17, 0.14) |
| Lesotho | 21.11 (10.95, 49.96) | 1.93 (1.02, 4.51) |  | 83.58 (28.30, 144.05) | 5.60 (1.97, 9.55) |  | 295.99 | 3.99 (3.14, 4.84) |
| Liberia | 40.15 (29.23, 54.03) | 3.44 (2.51, 4.62) |  | 55.16 (35.91, 87.00) | 2.29 (1.51, 3.54) |  | 37.37 | -1.52 (-1.66, -1.38) |
| Libya | 40.40 (27.50, 55.80) | 1.94 (1.33, 2.71) |  | 105.17 (69.73, 156.02) | 1.80 (1.20, 2.64) |  | 160.30 | -0.33 (-0.42, -0.24) |
| Lithuania | 18.40 (13.44, 24.79) | 0.42 (0.31, 0.56) |  | 33.02 (21.67, 49.06) | 0.69 (0.46, 1.01) |  | 79.47 | 0.50 (0.10, 0.91) |
| Luxembourg | 2.03 (1.37, 2.94) | 0.39 (0.27, 0.56) |  | 4.71 (2.94, 7.20) | 0.51 (0.33, 0.77) |  | 131.74 | 0.81 (0.44, 1.19) |
| Macedonia | 44.40 (31.56, 60.56) | 2.25 (1.60, 3.06) |  | 63.25 (41.37, 95.09) | 1.96 (1.30, 2.89) |  | 42.45 | -0.63 (-0.84, -0.43) |
| Madagascar | 57.73 (36.90, 101.08) | 0.96 (0.58, 1.72) |  | 99.08 (59.59, 163.72) | 0.72 (0.42, 1.23) |  | 71.61 | -1.26 (-1.37, -1.15) |
| Malawi | 45.92 (30.67, 71.02) | 0.99 (0.65, 1.59) |  | 65.27 (43.97, 90.68) | 0.74 (0.50, 1.07) |  | 42.12 | -2.26 (-2.69, -1.82) |
| Malaysia | 320.87 (255.28, 389.59) | 3.21 (2.52, 3.94) |  | 951.34 (677.04, 1261.41) | 3.38 (2.42, 4.47) |  | 196.49 | 0.26 (0.05, 0.47) |
| Maldives | 2.92 (1.79, 4.83) | 2.96 (1.83, 4.75) |  | 7.52 (5.46, 9.96) | 2.12 (1.49, 2.89) |  | 157.86 | -1.11 (-1.30, -0.91) |
| Mali | 285.74 (203.33, 374.58) | 6.01 (4.25, 7.96) |  | 548.76 (356.15, 798.37) | 5.42 (3.46, 7.81) |  | 92.05 | -0.57 (-0.66, -0.48) |
| Malta | 1.03 (0.70, 1.51) | 0.24 (0.17, 0.35) |  | 2.80 (1.84, 4.18) | 0.36 (0.25, 0.52) |  | 171.52 | 1.18 (0.92, 1.43) |
| Marshall Islands | 1.19 (0.86, 1.64) | 6.36 (4.49, 9.01) |  | 2.26 (1.50, 3.26) | 5.38 (3.59, 7.84) |  | 89.81 | -0.46 (-0.52, -0.40) |
| Mauritania | 39.06 (29.42, 50.32) | 3.61 (2.72, 4.65) |  | 43.04 (28.71, 60.50) | 1.93 (1.30, 2.72) |  | 10.19 | -2.18 (-2.33, -2.03) |
| Mauritius | 5.57 (4.29, 7.01) | 0.70 (0.53, 0.88) |  | 11.46 (7.56, 16.35) | 0.65 (0.44, 0.93) |  | 105.91 | 1.00 (0.58, 1.42) |
| Mexico | 142.41 (121.31, 167.60) | 0.30 (0.25, 0.35) |  | 479.95 (377.74, 609.90) | 0.40 (0.31, 0.51) |  | 237.01 | 0.91 (0.71, 1.10) |
| Moldova | 14.89 (10.72, 20.64) | 0.32 (0.23, 0.44) |  | 23.97 (16.31, 34.62) | 0.43 (0.30, 0.61) |  | 60.98 | -1.47 (-3.29, 0.38) |
| Monaco | 0.32 (0.20, 0.49) | 0.56 (0.36, 0.83) |  | 1.01 (0.64, 1.48) | 1.35 (0.88, 1.97) |  | 216.45 | 3.48 (2.66, 4.30) |
| Mongolia | 239.08 (166.85, 330.41) | 21.55 (14.91, 29.84) |  | 715.04 (470.10, 1044.02) | 27.28 (18.01, 39.10) |  | 199.09 | 1.17 (0.83, 1.52) |
| Montenegro | 9.30 (6.46, 12.99) | 1.44 (1.01, 2.03) |  | 12.37 (8.14, 18.28) | 1.31 (0.88, 1.90) |  | 33.12 | -0.32 (-0.69, 0.05) |
| Morocco | 100.04 (70.04, 137.97) | 0.71 (0.49, 0.99) |  | 230.13 (153.63, 327.20) | 0.72 (0.48, 1.02) |  | 130.05 | -0.19 (-0.40, 0.02) |
| Mozambique | 55.60 (34.95, 98.93) | 0.84 (0.52, 1.47) |  | 186.42 (117.42, 266.26) | 1.44 (0.91, 2.07) |  | 235.27 | 1.77 (1.50, 2.04) |
| Myanmar | 248.69 (156.06, 381.88) | 1.00 (0.63, 1.54) |  | 648.53 (459.86, 900.88) | 1.32 (0.93, 1.84) |  | 160.78 | 0.92 (0.77, 1.07) |
| Namibia | 4.98 (2.56, 10.15) | 0.64 (0.33, 1.31) |  | 17.26 (11.34, 24.52) | 1.07 (0.71, 1.55) |  | 246.20 | 1.98 (1.44, 2.51) |
| Nauru | 0.28 (0.18, 0.40) | 5.66 (3.81, 7.69) |  | 0.28 (0.17, 0.42) | 4.66 (3.12, 6.77) |  | -1.50 | -0.88 (-1.19, -0.57) |
| Nepal | 49.88 (33.36, 70.99) | 0.46 (0.31, 0.65) |  | 110.20 (68.22, 173.37) | 0.47 (0.29, 0.74) |  | 120.92 | 0.04 (-0.04, 0.12) |
| Netherlands | 38.07 (27.04, 52.90) | 0.20 (0.15, 0.28) |  | 132.31 (83.98, 203.48) | 0.47 (0.31, 0.70) |  | 247.57 | 3.26 (3.08, 3.45) |
| New Zealand | 16.87 (14.02, 20.15) | 0.45 (0.38, 0.54) |  | 57.56 (43.04, 74.98) | 0.88 (0.66, 1.14) |  | 241.24 | 2.53 (2.36, 2.70) |
| Nicaragua | 10.28 (7.46, 13.87) | 0.55 (0.39, 0.79) |  | 26.26 (17.27, 38.11) | 0.54 (0.35, 0.79) |  | 155.45 | -0.05 (-0.53, 0.43) |
| Niger | 11.43 (8.30, 15.22) | 0.36 (0.26, 0.47) |  | 27.18 (18.03, 37.86) | 0.30 (0.20, 0.42) |  | 137.88 | -0.60 (-0.68, -0.53) |
| Nigeria | 650.78 (468.77, 858.71) | 1.34 (0.96, 1.79) |  | 1119.36 (828.77, 1492.57) | 1.23 (0.91, 1.62) |  | 72.00 | -0.21 (-0.27, -0.14) |
| Niue | 0.09 (0.06, 0.12) | 4.25 (3.08, 5.58) |  | 0.08 (0.06, 0.11) | 3.69 (2.61, 5.06) |  | -10.67 | -0.63 (-0.71, -0.55) |
| North Korea | 1646.23 (1185.34, 2253.01) | 8.76 (6.38, 11.82) |  | 2013.90 (1393.53, 2795.39) | 6.05 (4.19, 8.45) |  | 22.33 | -1.53 (-1.65, -1.42) |
| Northern Mariana Islands | 0.92 (0.64, 1.24) | 3.59 (2.59, 4.74) |  | 2.73 (2.03, 3.64) | 4.47 (3.39, 5.79) |  | 196.18 | 1.13 (0.94, 1.32) |
| Norway | 16.13 (13.54, 18.92) | 0.28 (0.24, 0.33) |  | 35.95 (27.63, 46.55) | 0.45 (0.35, 0.58) |  | 122.89 | 2.04 (1.80, 2.27) |
| Oman | 13.41 (8.44, 19.51) | 1.63 (1.02, 2.39) |  | 42.10 (29.02, 58.48) | 1.76 (1.26, 2.47) |  | 213.83 | 0.95 (0.56, 1.35) |
| Pakistan | 333.33 (219.61, 463.10) | 0.51 (0.33, 0.72) |  | 725.41 (555.47, 943.89) | 0.51 (0.39, 0.67) |  | 117.63 | -0.04 (-0.23, 0.16) |
| Palau | 0.64 (0.42, 0.92) | 5.82 (3.75, 8.30) |  | 1.45 (1.03, 2.01) | 5.84 (4.24, 7.86) |  | 125.05 | -0.06 (-0.13, 0.00) |
| Palestine | 25.07 (16.91, 36.54) | 2.74 (1.85, 3.99) |  | 54.10 (39.86, 71.91) | 2.06 (1.49, 2.81) |  | 115.77 | -1.12 (-1.25, -1.00) |
| Panama | 9.77 (7.13, 13.31) | 0.60 (0.42, 0.83) |  | 18.77 (12.07, 28.37) | 0.45 (0.29, 0.68) |  | 92.21 | -0.83 (-1.24, -0.42) |
| Papua New Guinea | 12.66 (8.97, 17.60) | 0.66 (0.46, 0.91) |  | 32.51 (22.56, 45.52) | 0.69 (0.47, 0.96) |  | 156.74 | 0.21 (0.18, 0.24) |
| Paraguay | 9.54 (6.70, 13.21) | 0.40 (0.27, 0.56) |  | 20.81 (12.92, 31.79) | 0.36 (0.22, 0.56) |  | 118.02 | -0.18 (-0.68, 0.32) |
| Peru | 344.97 (269.60, 435.58) | 2.70 (2.06, 3.43) |  | 349.18 (236.59, 494.79) | 1.08 (0.73, 1.54) |  | 1.22 | -3.99 (-4.54, -3.44) |
| Philippines | 1653.14 (1228.72, 2056.64) | 4.53 (3.29, 5.74) |  | 2457.90 (1903.21, 3172.43) | 2.73 (2.11, 3.52) |  | 48.68 | -2.20 (-2.47, -1.93) |
| Poland | 698.74 (595.27, 816.29) | 1.61 (1.38, 1.86) |  | 252.01 (193.79, 324.72) | 0.38 (0.30, 0.49) |  | -63.93 | -4.93 (-6.17, -3.67) |
| Portugal | 36.15 (25.25, 50.42) | 0.28 (0.20, 0.38) |  | 137.39 (83.74, 212.76) | 0.74 (0.46, 1.14) |  | 280.06 | 3.06 (2.69, 3.43) |
| Puerto Rico | 63.89 (46.35, 85.83) | 1.76 (1.29, 2.35) |  | 44.11 (27.78, 65.96) | 0.71 (0.46, 1.06) |  | -30.96 | -1.96 (-2.60, -1.32) |
| Qatar | 6.33 (4.28, 9.24) | 5.26 (3.43, 7.90) |  | 43.83 (27.90, 66.68) | 4.73 (2.91, 7.33) |  | 592.25 | -0.10 (-0.24, 0.04) |
| Romania | 118.72 (84.22, 160.77) | 0.43 (0.31, 0.57) |  | 209.28 (140.05, 306.35) | 0.65 (0.44, 0.94) |  | 76.29 | 2.05 (1.57, 2.52) |
| Russia | 836.59 (716.51, 981.74) | 0.46 (0.40, 0.54) |  | 1526.95 (1187.67, 1985.88) | 0.70 (0.55, 0.91) |  | 82.52 | 1.69 (1.40, 1.98) |
| Rwanda | 47.21 (30.92, 71.27) | 1.38 (0.91, 2.05) |  | 81.68 (52.60, 118.40) | 1.11 (0.73, 1.61) |  | 73.00 | -1.54 (-1.80, -1.28) |
| Saint Kitts and Nevis | 1.33 (0.95, 1.76) | 3.78 (2.82, 4.91) |  | 0.73 (0.49, 1.05) | 1.02 (0.69, 1.45) |  | -44.78 | -5.17 (-6.22, -4.10) |
| Saint Lucia | 1.48 (1.11, 1.95) | 1.64 (1.22, 2.16) |  | 1.18 (0.83, 1.64) | 0.54 (0.38, 0.75) |  | -20.43 | -3.98 (-4.98, -2.97) |
| Saint Vincent and the Grenadines | 1.58 (1.17, 2.10) | 2.16 (1.60, 2.85) |  | 1.11 (0.79, 1.50) | 0.81 (0.59, 1.09) |  | -30.21 | -3.47 (-4.27, -2.66) |
| Samoa | 3.16 (2.29, 4.18) | 3.32 (2.38, 4.37) |  | 4.34 (3.10, 5.91) | 2.70 (1.94, 3.68) |  | 37.08 | -0.73 (-0.82, -0.64) |
| San Marino | 0.10 (0.07, 0.15) | 0.32 (0.22, 0.48) |  | 0.24 (0.15, 0.38) | 0.46 (0.29, 0.70) |  | 139.10 | 1.69 (1.49, 1.88) |
| Sao Tome and Principe | 0.83 (0.58, 1.08) | 1.23 (0.87, 1.58) |  | 1.54 (0.96, 2.22) | 1.21 (0.76, 1.73) |  | 85.49 | -0.08 (-0.18, 0.03) |
| Saudi Arabia | 127.03 (84.56, 176.77) | 1.96 (1.28, 2.79) |  | 334.28 (224.72, 476.71) | 1.66 (1.11, 2.39) |  | 163.15 | -0.95 (-1.12, -0.78) |
| Senegal | 46.31 (35.82, 57.44) | 1.33 (1.03, 1.65) |  | 95.84 (67.22, 128.49) | 1.18 (0.84, 1.57) |  | 106.96 | -0.36 (-0.48, -0.24) |
| Serbia | 164.94 (113.60, 231.13) | 1.40 (0.97, 1.93) |  | 161.48 (101.17, 246.66) | 1.09 (0.70, 1.65) |  | -2.10 | -1.20 (-1.57, -0.82) |
| Seychelles | 1.83 (1.39, 2.40) | 3.26 (2.46, 4.30) |  | 2.46 (1.79, 3.30) | 2.07 (1.51, 2.79) |  | 34.40 | -1.56 (-1.85, -1.26) |
| Sierra Leone | 59.56 (41.06, 81.01) | 2.94 (2.04, 4.01) |  | 87.58 (60.21, 124.15) | 2.14 (1.48, 3.07) |  | 47.05 | -1.15 (-1.24, -1.05) |
| Singapore | 122.87 (105.04, 141.78) | 5.23 (4.39, 6.10) |  | 498.92 (364.35, 651.88) | 6.36 (4.61, 8.31) |  | 306.06 | 0.58 (0.31, 0.84) |
| Slovakia | 64.66 (46.78, 87.02) | 1.09 (0.79, 1.46) |  | 61.53 (38.72, 93.15) | 0.70 (0.45, 1.04) |  | -4.83 | -2.16 (-2.53, -1.79) |
| Slovenia | 17.60 (11.44, 26.03) | 0.72 (0.47, 1.07) |  | 47.08 (29.57, 71.08) | 1.21 (0.76, 1.81) |  | 167.56 | 1.86 (1.44, 2.28) |
| Solomon Islands | 6.47 (4.61, 8.58) | 3.72 (2.61, 4.97) |  | 10.83 (8.10, 14.33) | 2.75 (2.06, 3.61) |  | 67.33 | -1.04 (-1.13, -0.95) |
| Somalia | 38.19 (21.10, 73.83) | 1.21 (0.67, 2.31) |  | 89.94 (50.79, 167.30) | 1.09 (0.62, 2.02) |  | 135.53 | -0.35 (-0.41, -0.29) |
| South Africa | 517.70 (334.20, 909.04) | 2.07 (1.30, 3.70) |  | 995.18 (831.11, 1182.84) | 1.96 (1.64, 2.32) |  | 92.23 | -0.61 (-1.27, 0.04) |
| South Korea | 2353.62 (1877.74, 2934.99) | 6.83 (5.43, 8.45) |  | 11449.42 (8884.66, 14577.82) | 12.82 (9.97, 16.31) |  | 386.46 | 3.15 (1.75, 4.56) |
| South Sudan | 26.25 (14.57, 52.29) | 0.98 (0.54, 1.98) |  | 38.48 (20.27, 72.55) | 0.86 (0.45, 1.63) |  | 46.58 | -0.49 (-0.55, -0.43) |
| Spain | 240.84 (166.75, 339.50) | 0.47 (0.33, 0.65) |  | 579.49 (361.63, 899.31) | 0.74 (0.47, 1.14) |  | 140.61 | 1.41 (1.00, 1.82) |
| Sri Lanka | 99.04 (75.94, 125.92) | 0.85 (0.64, 1.10) |  | 263.84 (170.27, 385.75) | 1.01 (0.67, 1.47) |  | 166.39 | 1.47 (1.07, 1.87) |
| Sudan | 127.44 (75.26, 197.58) | 1.30 (0.76, 2.03) |  | 247.12 (132.74, 406.59) | 1.24 (0.68, 2.05) |  | 93.91 | -0.19 (-0.30, -0.09) |
| Suriname | 5.38 (4.04, 7.09) | 1.92 (1.42, 2.56) |  | 4.41 (3.02, 6.23) | 0.71 (0.49, 1.00) |  | -18.17 | -3.26 (-3.94, -2.57) |
| Sweden | 20.66 (16.04, 26.93) | 0.16 (0.13, 0.21) |  | 33.67 (24.02, 44.97) | 0.20 (0.15, 0.27) |  | 62.94 | 1.36 (0.72, 2.00) |
| Switzerland | 35.26 (24.13, 50.74) | 0.38 (0.26, 0.53) |  | 126.06 (77.82, 194.54) | 0.84 (0.53, 1.29) |  | 257.53 | 2.02 (1.37, 2.67) |
| Syrian Arab Republic | 105.17 (75.34, 145.00) | 1.80 (1.24, 2.54) |  | 194.05 (128.58, 282.08) | 1.47 (0.98, 2.12) |  | 84.51 | -0.87 (-1.05, -0.69) |
| Taiwan (Province of China) | 1199.06 (1019.81, 1372.33) | 6.67 (5.66, 7.69) |  | 1464.39 (1044.22, 2002.70) | 3.84 (2.75, 5.24) |  | 22.13 | -3.04 (-4.02, -2.04) |
| Tajikistan | 5.93 (4.42, 7.91) | 0.18 (0.13, 0.25) |  | 38.41 (26.64, 53.81) | 0.62 (0.42, 0.88) |  | 548.03 | 4.82 (4.58, 5.06) |
| Tanzania | 71.49 (50.50, 99.75) | 0.56 (0.39, 0.81) |  | 163.43 (111.42, 234.97) | 0.57 (0.38, 0.82) |  | 128.60 | -0.08 (-0.22, 0.05) |
| Thailand | 3988.19 (3146.15, 4910.43) | 9.72 (7.66, 12.07) |  | 10263.62 (6986.55, 14642.73) | 9.97 (6.88, 14.14) |  | 157.35 | 0.00 (-0.06, 0.07) |
| Timor-Leste | 11.73 (7.28, 18.28) | 3.25 (2.01, 5.05) |  | 21.55 (11.70, 33.11) | 2.49 (1.38, 3.82) |  | 83.75 | -1.11 (-1.28, -0.94) |
| Togo | 46.76 (34.76, 62.22) | 3.24 (2.34, 4.31) |  | 99.59 (69.69, 138.80) | 2.31 (1.64, 3.19) |  | 112.98 | -1.58 (-1.78, -1.39) |
| Tokelau | 0.06 (0.04, 0.08) | 4.30 (2.90, 6.05) |  | 0.05 (0.03, 0.07) | 3.80 (2.50, 5.41) |  | -9.47 | -0.49 (-0.53, -0.45) |
| Tonga | 7.98 (5.17, 11.08) | 13.12 (8.52, 18.26) |  | 10.74 (7.29, 15.17) | 13.05 (8.89, 18.45) |  | 34.53 | -0.06 (-0.13, 0.01) |
| Trinidad and Tobago | 16.59 (12.47, 21.83) | 1.88 (1.39, 2.49) |  | 11.88 (7.60, 17.81) | 0.64 (0.42, 0.95) |  | -28.39 | -3.85 (-4.85, -2.84) |
| Tunisia | 31.15 (20.98, 44.65) | 0.58 (0.39, 0.83) |  | 66.93 (39.82, 104.22) | 0.52 (0.31, 0.80) |  | 114.85 | -0.30 (-0.41, -0.20) |
| Turkey | 677.52 (515.38, 861.27) | 1.77 (1.33, 2.26) |  | 1155.66 (844.16, 1572.07) | 1.29 (0.94, 1.76) |  | 70.57 | -1.18 (-1.34, -1.02) |
| Turkmenistan | 5.94 (4.53, 7.77) | 0.27 (0.20, 0.37) |  | 67.10 (45.43, 95.83) | 1.45 (0.99, 2.05) |  | 1028.80 | 6.86 (5.52, 8.22) |
| Tuvalu | 0.42 (0.28, 0.57) | 5.57 (3.88, 7.64) |  | 0.48 (0.33, 0.69) | 4.46 (3.10, 6.41) |  | 16.17 | -0.81 (-0.94, -0.67) |
| Uganda | 101.59 (70.29, 144.09) | 1.36 (0.94, 1.95) |  | 297.54 (201.33, 426.14) | 1.63 (1.10, 2.36) |  | 192.88 | 0.57 (0.33, 0.82) |
| Ukraine | 163.47 (138.49, 193.08) | 0.24 (0.20, 0.27) |  | 465.71 (371.71, 578.55) | 0.68 (0.55, 0.86) |  | 184.89 | 4.64 (3.94, 5.35) |
| United Arab Emirates | 10.32 (5.20, 19.66) | 1.55 (0.71, 3.18) |  | 115.43 (47.64, 264.41) | 1.73 (0.69, 4.15) |  | 1018.95 | 0.17 (0.04, 0.31) |
| United Kingdom | 246.02 (205.50, 292.82) | 0.31 (0.26, 0.36) |  | 803.83 (609.77, 1030.62) | 0.75 (0.57, 0.95) |  | 226.73 | 3.86 (3.62, 4.09) |
| United States of America | 965.27 (839.35, 1109.78) | 0.32 (0.28, 0.37) |  | 3442.79 (2613.25, 4474.02) | 0.69 (0.53, 0.89) |  | 256.67 | 2.69 (2.41, 2.96) |
| United States Virgin Islands | 1.10 (0.76, 1.50) | 1.18 (0.81, 1.63) |  | 1.17 (0.79, 1.68) | 0.66 (0.46, 0.94) |  | 5.99 | -2.11 (-2.76, -1.46) |
| Uruguay | 10.65 (7.33, 15.38) | 0.28 (0.20, 0.40) |  | 21.11 (13.55, 31.91) | 0.43 (0.29, 0.66) |  | 98.21 | 1.76 (1.58, 1.93) |
| Uzbekistan | 27.23 (20.39, 35.81) | 0.22 (0.16, 0.29) |  | 387.37 (271.25, 548.31) | 1.51 (1.05, 2.13) |  | 1322.84 | 9.16 (7.77, 10.57) |
| Vanuatu | 3.81 (2.39, 5.84) | 5.11 (3.09, 7.91) |  | 9.40 (6.00, 14.06) | 4.86 (3.10, 7.20) |  | 146.50 | -0.15 (-0.26, -0.05) |
| Venezuela | 110.68 (78.88, 152.88) | 1.04 (0.71, 1.47) |  | 102.19 (63.99, 159.11) | 0.34 (0.22, 0.53) |  | -7.67 | -3.11 (-4.23, -1.99) |
| Viet Nam | 704.55 (482.10, 968.24) | 1.69 (1.15, 2.32) |  | 952.78 (648.49, 1340.81) | 0.99 (0.68, 1.41) |  | 35.23 | -2.16 (-2.43, -1.90) |
| Yemen | 55.50 (32.37, 91.00) | 1.03 (0.61, 1.69) |  | 139.00 (87.71, 211.12) | 0.96 (0.59, 1.46) |  | 150.45 | -0.30 (-0.38, -0.23) |
| Zambia | 23.13 (15.29, 35.27) | 0.68 (0.45, 1.06) |  | 61.67 (41.99, 86.73) | 0.75 (0.51, 1.07) |  | 166.62 | -0.45 (-0.90, -0.00) |
| Zimbabwe | 222.88 (150.05, 379.96) | 4.65 (3.11, 7.83) |  | 399.79 (264.66, 612.45) | 4.58 (3.01, 7.03) |  | 79.37 | -1.47 (-2.08, -0.86) |

ASIR: age-standardized incidence rate; CI: confidence interval; EAPC: estimated annual percentage change; UI: uncertainty interval.

**
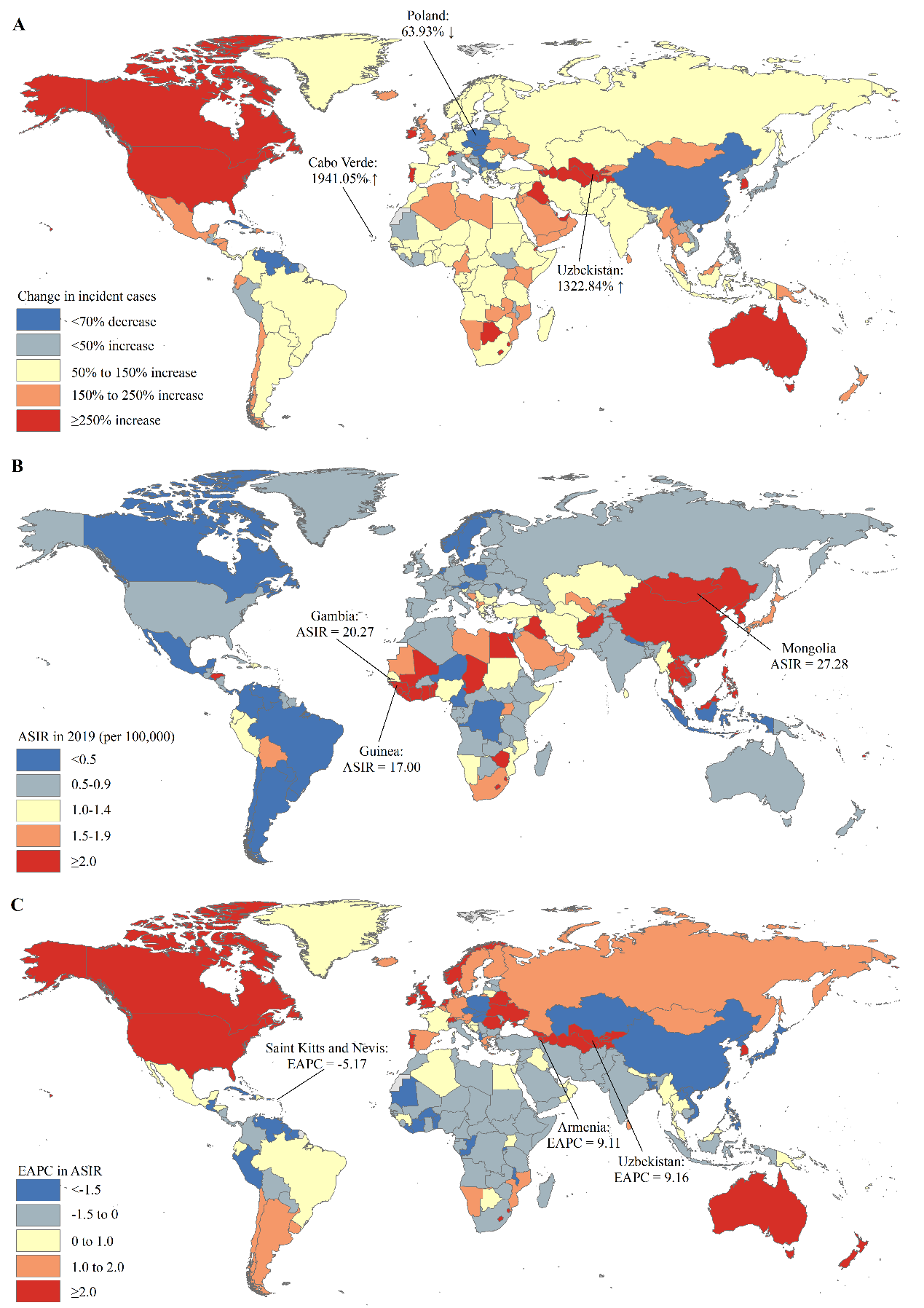
**

**Figure S1.** The global trends in the incidence of primary liver cancer due to hepatitis B in 204 countries and territories. (A) The percentage change in incident cases of primary liver cancer between 1990 and 2019; (B) The ASIR of primary liver cancer in 2019; (C) The EAPCs in ASIR of primary liver cancer from 1990 to 2019.

Note: ASIR: age-standardized incidence rate; EAPC: estimated annual percentage change.

**Table S14**. **The incident cases and ASIR of primary liver cancer due to hepatitis C in 1990 and 2019 and their change trends from 1990 to 2019 at national level.**

| Nation | 1990 | |  | 2019 | |  | 1990-2019 | |
| --- | --- | --- | --- | --- | --- | --- | --- | --- |
|  | Incident cases  No. (95% UI) | ASIR per 100,000  No. (95% UI) |  | Incident cases  No. x 10^3^ (95% UI) | ASIR per 100,000  No. (95% UI) |  | Percentage change in incident cases  No. (%) | EAPC in ASIR  No. (95% CI) |
| Afghanistan | 304.11 (204.50, 427.62) | 4.43 (3.04, 6.04) |  | 438.78 (290.61, 622.47) | 3.79 (2.62, 5.31) |  | 44.29 | -0.63 (-0.75, -0.51) |
| Albania | 61.27 (43.32, 80.80) | 3.23 (2.31, 4.22) |  | 74.17 (46.52, 111.42) | 1.69 (1.08, 2.52) |  | 21.06 | -3.32 (-3.89, -2.75) |
| Algeria | 69.32 (47.26, 95.62) | 0.66 (0.47, 0.87) |  | 259.90 (180.68, 356.52) | 0.87 (0.61, 1.18) |  | 274.91 | 0.92 (0.77, 1.08) |
| American Samoa | 0.25 (0.16, 0.35) | 1.24 (0.81, 1.72) |  | 0.63 (0.41, 0.89) | 1.40 (0.95, 1.91) |  | 158.37 | 0.82 (0.60, 1.04) |
| Andorra | 1.69 (1.05, 2.58) | 3.25 (2.12, 4.91) |  | 5.44 (3.56, 7.67) | 3.84 (2.50, 5.45) |  | 222.26 | 0.69 (0.59, 0.78) |
| Angola | 43.27 (29.73, 60.27) | 1.25 (0.88, 1.69) |  | 109.56 (76.88, 148.71) | 1.13 (0.82, 1.48) |  | 153.22 | -0.59 (-0.68, -0.49) |
| Antigua and Barbuda | 0.96 (0.66, 1.30) | 1.75 (1.19, 2.38) |  | 0.60 (0.40, 0.84) | 0.63 (0.43, 0.88) |  | -37.93 | -3.90 (-4.90, -2.89) |
| Argentina | 148.71 (107.45, 201.66) | 0.47 (0.34, 0.63) |  | 384.51 (263.29, 529.38) | 0.70 (0.48, 0.97) |  | 158.56 | 2.12 (1.89, 2.36) |
| Armenia | 9.86 (7.30, 12.35) | 0.40 (0.30, 0.50) |  | 103.07 (73.25, 137.40) | 2.49 (1.78, 3.27) |  | 945.47 | 9.60 (8.02, 11.19) |
| Australia | 109.57 (79.29, 142.84) | 0.56 (0.41, 0.72) |  | 580.87 (387.80, 824.56) | 1.39 (0.93, 1.97) |  | 430.16 | 3.49 (3.26, 3.73) |
| Austria | 119.50 (87.54, 155.36) | 0.99 (0.73, 1.29) |  | 289.29 (198.41, 411.73) | 1.60 (1.10, 2.30) |  | 142.10 | 2.01 (1.63, 2.38) |
| Azerbaijan | 16.47 (12.28, 20.94) | 0.36 (0.27, 0.45) |  | 121.36 (78.84, 177.68) | 1.52 (1.01, 2.26) |  | 637.07 | 6.06 (3.98, 8.18) |
| Bahamas | 2.34 (1.59, 3.23) | 1.61 (1.10, 2.21) |  | 2.44 (1.58, 3.43) | 0.66 (0.44, 0.91) |  | 4.00 | -3.37 (-4.22, -2.51) |
| Bahrain | 2.88 (1.98, 3.98) | 2.23 (1.60, 2.91) |  | 14.74 (9.99, 20.61) | 2.35 (1.67, 3.14) |  | 411.64 | -0.47 (-0.87, -0.06) |
| Bangladesh | 534.72 (378.73, 730.47) | 1.19 (0.85, 1.61) |  | 1384.46 (973.47, 1845.39) | 1.11 (0.78, 1.46) |  | 158.91 | -0.51 (-0.61, -0.40) |
| Barbados | 1.90 (1.21, 2.81) | 0.62 (0.40, 0.91) |  | 2.88 (1.93, 4.12) | 0.58 (0.39, 0.82) |  | 51.84 | -0.33 (-0.49, -0.17) |
| Belarus | 43.02 (30.68, 56.64) | 0.33 (0.24, 0.43) |  | 93.92 (60.57, 137.33) | 0.58 (0.37, 0.85) |  | 118.31 | 2.46 (1.96, 2.95) |
| Belgium | 175.82 (132.82, 217.94) | 1.11 (0.84, 1.37) |  | 355.59 (248.31, 484.20) | 1.51 (1.05, 2.08) |  | 102.25 | 0.95 (0.56, 1.34) |
| Belize | 1.34 (0.95, 1.81) | 1.49 (1.05, 2.01) |  | 1.69 (1.13, 2.37) | 0.66 (0.45, 0.92) |  | 25.65 | -3.01 (-3.66, -2.36) |
| Benin | 23.50 (14.88, 33.59) | 1.26 (0.81, 1.78) |  | 40.90 (25.43, 58.09) | 0.96 (0.62, 1.35) |  | 74.06 | -1.17 (-1.30, -1.04) |
| Bermuda | 0.91 (0.61, 1.25) | 1.52 (1.04, 2.08) |  | 0.58 (0.37, 0.83) | 0.42 (0.28, 0.61) |  | -36.72 | -4.43 (-5.46, -3.38) |
| Bhutan | 1.48 (0.85, 2.33) | 0.65 (0.39, 1.01) |  | 5.23 (3.22, 8.00) | 1.00 (0.62, 1.54) |  | 252.63 | 1.68 (1.58, 1.77) |
| Bolivia | 11.90 (6.36, 20.36) | 0.42 (0.23, 0.71) |  | 31.69 (17.42, 53.08) | 0.40 (0.22, 0.65) |  | 166.37 | -0.23 (-0.33, -0.14) |
| Bosnia and Herzegovina | 59.23 (40.12, 80.10) | 1.53 (1.07, 2.03) |  | 136.85 (92.84, 194.51) | 2.26 (1.55, 3.18) |  | 131.05 | 1.97 (1.56, 2.39) |
| Botswana | 1.59 (0.76, 3.15) | 0.31 (0.15, 0.60) |  | 5.13 (3.17, 7.48) | 0.41 (0.27, 0.59) |  | 223.48 | 0.41 (-0.13, 0.96) |
| Brazil | 639.76 (569.69, 711.93) | 0.78 (0.69, 0.86) |  | 2177.98 (1906.53, 2436.50) | 0.94 (0.82, 1.05) |  | 240.44 | 1.17 (0.97, 1.38) |
| Brunei | 2.70 (1.77, 3.85) | 3.49 (2.36, 4.77) |  | 10.98 (7.45, 15.24) | 4.19 (3.04, 5.52) |  | 306.13 | 1.22 (0.71, 1.73) |
| Bulgaria | 179.73 (120.98, 245.86) | 1.43 (1.00, 1.92) |  | 141.82 (93.14, 208.83) | 0.97 (0.64, 1.41) |  | -21.09 | -0.71 (-1.23, -0.19) |
| Burkina Faso | 33.21 (22.31, 46.18) | 0.87 (0.59, 1.17) |  | 47.77 (31.76, 65.79) | 0.60 (0.39, 0.82) |  | 43.83 | -1.47 (-1.68, -1.26) |
| Burundi | 17.48 (10.32, 26.93) | 0.81 (0.50, 1.22) |  | 27.76 (16.34, 43.25) | 0.71 (0.44, 1.09) |  | 58.86 | -0.97 (-1.14, -0.81) |
| Cambodia | 180.59 (125.43, 243.29) | 4.27 (3.04, 5.55) |  | 391.93 (273.81, 525.21) | 3.54 (2.54, 4.63) |  | 117.03 | -0.88 (-0.98, -0.77) |
| Cameroon | 4.58 (2.65, 7.30) | 0.12 (0.07, 0.18) |  | 11.30 (6.92, 17.00) | 0.11 (0.07, 0.16) |  | 146.67 | -0.38 (-0.53, -0.23) |
| Canada | 115.54 (80.45, 156.67) | 0.36 (0.25, 0.48) |  | 577.98 (360.81, 851.64) | 0.83 (0.52, 1.22) |  | 400.25 | 3.32 (3.07, 3.56) |
| Cape Verde | 0.57 (0.39, 0.78) | 0.24 (0.16, 0.33) |  | 8.94 (5.97, 12.57) | 2.22 (1.46, 3.17) |  | 1476.92 | 6.05 (4.00, 8.13) |
| Central African Republic | 16.66 (10.38, 24.87) | 1.55 (1.01, 2.25) |  | 27.77 (16.30, 42.88) | 1.46 (0.90, 2.17) |  | 66.63 | -0.71 (-0.93, -0.48) |
| Chad | 34.06 (21.50, 49.11) | 1.28 (0.81, 1.81) |  | 52.93 (32.35, 77.66) | 1.07 (0.68, 1.54) |  | 55.38 | -0.75 (-0.80, -0.70) |
| Chile | 69.73 (50.54, 89.25) | 0.72 (0.52, 0.92) |  | 252.19 (167.53, 352.45) | 1.04 (0.69, 1.45) |  | 261.69 | 1.91 (1.60, 2.23) |
| China | 35446.63 (28972.20, 43242.24) | 4.53 (3.76, 5.48) |  | 34036.44 (27796.29, 40829.00) | 1.75 (1.44, 2.09) |  | -3.98 | -4.75 (-5.51, -3.98) |
| Colombia | 176.95 (134.46, 219.86) | 1.09 (0.83, 1.34) |  | 489.09 (336.89, 684.48) | 0.92 (0.63, 1.29) |  | 176.41 | -0.39 (-0.85, 0.07) |
| Comoros | 1.62 (0.86, 2.65) | 0.79 (0.45, 1.27) |  | 3.40 (2.10, 5.21) | 0.75 (0.48, 1.15) |  | 109.52 | -0.32 (-0.43, -0.22) |
| Congo | 18.57 (12.44, 26.64) | 1.88 (1.32, 2.62) |  | 31.78 (20.95, 46.67) | 1.38 (0.95, 1.97) |  | 71.13 | -1.45 (-1.58, -1.33) |
| Cook Islands | 0.35 (0.23, 0.49) | 2.96 (1.97, 4.05) |  | 0.57 (0.37, 0.81) | 2.27 (1.52, 3.21) |  | 62.03 | -0.86 (-0.93, -0.79) |
| Costa Rica | 33.80 (25.44, 43.13) | 2.01 (1.51, 2.56) |  | 93.30 (62.72, 129.31) | 1.84 (1.24, 2.54) |  | 176.01 | -0.50 (-1.03, 0.04) |
| Cote d'Ivoire | 41.17 (25.17, 61.43) | 1.23 (0.80, 1.80) |  | 73.55 (44.38, 110.58) | 0.84 (0.53, 1.26) |  | 78.66 | -1.81 (-2.01, -1.60) |
| Croatia | 39.80 (26.64, 55.38) | 0.63 (0.43, 0.87) |  | 61.40 (39.11, 88.69) | 0.67 (0.43, 0.96) |  | 54.28 | 0.80 (0.37, 1.24) |
| Cuba | 158.30 (111.05, 210.32) | 1.53 (1.08, 2.03) |  | 100.99 (66.09, 144.09) | 0.52 (0.34, 0.75) |  | -36.20 | -4.12 (-5.30, -2.92) |
| Cyprus | 9.15 (6.31, 12.24) | 1.22 (0.88, 1.60) |  | 26.60 (19.14, 34.48) | 1.41 (1.04, 1.82) |  | 190.85 | 0.87 (0.58, 1.17) |
| Czech Republic | 133.06 (90.66, 178.16) | 0.95 (0.66, 1.27) |  | 141.31 (92.99, 205.46) | 0.65 (0.42, 0.94) |  | 6.20 | -1.41 (-1.61, -1.22) |
| Democratic Republic of the Congo | 171.20 (123.96, 226.25) | 1.19 (0.90, 1.52) |  | 351.78 (244.60, 484.36) | 1.07 (0.76, 1.43) |  | 105.47 | -0.47 (-0.55, -0.39) |
| Denmark | 67.68 (49.60, 87.64) | 0.82 (0.60, 1.05) |  | 152.21 (103.52, 215.24) | 1.32 (0.90, 1.86) |  | 124.90 | 2.05 (1.80, 2.30) |
| Djibouti | 0.91 (0.52, 1.54) | 0.79 (0.48, 1.30) |  | 3.93 (2.26, 6.70) | 0.80 (0.49, 1.29) |  | 330.61 | -0.05 (-0.15, 0.06) |
| Dominica | 1.52 (1.06, 2.08) | 2.03 (1.43, 2.76) |  | 0.64 (0.42, 0.90) | 0.69 (0.46, 0.99) |  | -58.23 | -3.81 (-4.62, -3.00) |
| Dominican Republic | 30.15 (20.99, 40.95) | 0.88 (0.62, 1.18) |  | 87.34 (54.53, 138.70) | 0.98 (0.62, 1.55) |  | 189.65 | 0.70 (0.21, 1.19) |
| Ecuador | 11.30 (7.15, 16.47) | 0.23 (0.15, 0.34) |  | 41.06 (24.39, 62.06) | 0.29 (0.18, 0.44) |  | 263.54 | 1.37 (0.99, 1.75) |
| Egypt | 3294.40 (2484.08, 4067.42) | 11.30 (8.43, 13.90) |  | 8677.72 (5839.76, 12410.66) | 13.64 (9.44, 19.19) |  | 163.41 | 1.59 (1.19, 1.99) |
| El Salvador | 39.58 (30.01, 49.53) | 1.38 (1.05, 1.73) |  | 45.11 (30.41, 63.50) | 0.75 (0.50, 1.06) |  | 13.96 | -2.22 (-2.97, -1.46) |
| Equatorial Guinea | 2.73 (1.81, 3.92) | 1.47 (1.02, 2.03) |  | 5.93 (3.32, 9.29) | 1.42 (0.81, 2.18) |  | 117.62 | -0.27 (-0.50, -0.04) |
| Eritrea | 6.94 (3.68, 12.00) | 0.83 (0.47, 1.38) |  | 18.97 (11.43, 28.55) | 0.87 (0.54, 1.28) |  | 173.39 | -0.10 (-0.23, 0.04) |
| Estonia | 11.18 (7.87, 14.85) | 0.54 (0.39, 0.72) |  | 24.38 (15.79, 34.38) | 0.87 (0.56, 1.26) |  | 118.02 | 0.61 (0.08, 1.14) |
| Eswatini | 4.42 (2.64, 7.71) | 1.69 (1.03, 2.90) |  | 21.62 (7.21, 41.21) | 4.04 (1.43, 7.46) |  | 388.76 | 3.95 (2.96, 4.96) |
| Ethiopia | 191.29 (130.12, 272.35) | 1.09 (0.77, 1.51) |  | 383.66 (301.69, 491.83) | 1.06 (0.83, 1.35) |  | 100.56 | -0.30 (-0.37, -0.22) |
| Federated States of Micronesia | 1.04 (0.62, 1.62) | 2.50 (1.56, 3.74) |  | 1.42 (0.78, 2.33) | 2.29 (1.37, 3.46) |  | 36.19 | -0.34 (-0.49, -0.20) |
| Fiji | 4.21 (2.65, 6.13) | 1.34 (0.88, 1.91) |  | 9.24 (5.88, 13.66) | 1.36 (0.91, 1.97) |  | 119.51 | 0.52 (0.21, 0.84) |
| Finland | 89.98 (66.67, 114.31) | 1.23 (0.93, 1.56) |  | 258.01 (180.87, 358.84) | 2.00 (1.39, 2.76) |  | 186.74 | 1.94 (1.80, 2.09) |
| France | 1544.78 (1143.47, 2013.26) | 1.87 (1.39, 2.46) |  | 3644.99 (2535.72, 5003.29) | 2.68 (1.84, 3.71) |  | 135.96 | 1.06 (0.78, 1.35) |
| Gabon | 8.91 (6.11, 12.06) | 1.67 (1.17, 2.22) |  | 15.12 (9.46, 22.61) | 1.58 (1.02, 2.33) |  | 69.71 | -0.44 (-0.55, -0.32) |
| Gambia | 13.03 (7.84, 19.30) | 4.03 (2.54, 5.88) |  | 49.39 (30.63, 73.25) | 5.47 (3.43, 8.11) |  | 279.16 | 0.77 (0.60, 0.93) |
| Georgia | 24.24 (17.93, 31.17) | 0.41 (0.31, 0.52) |  | 64.88 (46.44, 86.91) | 1.07 (0.76, 1.44) |  | 167.69 | 2.43 (1.11, 3.76) |
| Germany | 957.01 (734.98, 1207.49) | 0.74 (0.57, 0.93) |  | 2725.33 (1844.77, 3793.37) | 1.37 (0.93, 1.90) |  | 184.77 | 2.18 (1.68, 2.68) |
| Ghana | 56.50 (35.11, 82.69) | 1.03 (0.66, 1.49) |  | 128.12 (78.28, 190.39) | 0.90 (0.58, 1.31) |  | 126.77 | -0.64 (-0.79, -0.49) |
| Greece | 64.67 (44.73, 88.05) | 0.42 (0.30, 0.57) |  | 157.69 (104.01, 229.32) | 0.59 (0.38, 0.86) |  | 143.85 | 1.39 (1.30, 1.49) |
| Greenland | 0.41 (0.27, 0.57) | 1.19 (0.82, 1.63) |  | 1.16 (0.73, 1.69) | 1.65 (1.09, 2.39) |  | 184.25 | 0.89 (0.71, 1.08) |
| Grenada | 1.58 (1.10, 2.13) | 2.09 (1.45, 2.80) |  | 0.68 (0.46, 0.96) | 0.65 (0.44, 0.89) |  | -56.77 | -4.24 (-5.22, -3.24) |
| Guam | 0.52 (0.33, 0.75) | 0.82 (0.55, 1.13) |  | 1.73 (1.11, 2.58) | 0.91 (0.59, 1.34) |  | 235.49 | 0.70 (0.57, 0.82) |
| Guatemala | 132.21 (99.08, 166.67) | 4.03 (3.07, 5.00) |  | 200.09 (142.05, 272.89) | 1.90 (1.37, 2.58) |  | 51.34 | -3.27 (-4.70, -1.81) |
| Guinea | 165.22 (106.15, 234.18) | 5.35 (3.51, 7.46) |  | 271.82 (165.10, 404.65) | 5.38 (3.29, 8.00) |  | 64.53 | 0.08 (0.05, 0.12) |
| Guinea-Bissau | 5.50 (3.21, 8.88) | 1.49 (0.90, 2.33) |  | 7.71 (4.55, 12.19) | 1.24 (0.75, 1.89) |  | 40.16 | -0.75 (-0.82, -0.69) |
| Guyana | 5.10 (3.45, 7.27) | 1.46 (1.00, 2.03) |  | 3.85 (2.40, 5.67) | 0.67 (0.43, 0.96) |  | -24.45 | -2.92 (-3.75, -2.08) |
| Haiti | 48.53 (26.49, 79.32) | 1.69 (0.95, 2.74) |  | 60.67 (32.58, 98.78) | 0.98 (0.53, 1.56) |  | 25.02 | -1.93 (-2.15, -1.72) |
| Honduras | 86.26 (29.10, 136.74) | 4.48 (1.48, 7.06) |  | 342.96 (140.18, 558.02) | 6.06 (2.50, 9.75) |  | 297.59 | 1.26 (1.12, 1.40) |
| Hungary | 220.26 (153.32, 292.27) | 1.49 (1.05, 1.95) |  | 119.07 (78.35, 168.04) | 0.59 (0.39, 0.84) |  | -45.94 | -2.42 (-3.13, -1.71) |
| Iceland | 2.17 (1.64, 2.79) | 0.76 (0.57, 0.97) |  | 6.70 (4.90, 8.89) | 1.20 (0.87, 1.59) |  | 208.37 | 1.43 (1.28, 1.57) |
| India | 1934.52 (1516.72, 2422.25) | 0.51 (0.40, 0.63) |  | 5842.04 (4521.48, 7312.91) | 0.55 (0.43, 0.69) |  | 201.99 | 0.23 (0.13, 0.32) |
| Indonesia | 1014.68 (855.53, 1210.69) | 1.16 (0.98, 1.39) |  | 2002.08 (1633.90, 2332.58) | 1.08 (0.89, 1.23) |  | 97.31 | -0.43 (-0.52, -0.34) |
| Iran | 258.26 (197.61, 339.80) | 1.26 (0.96, 1.66) |  | 764.23 (649.14, 891.21) | 1.16 (0.98, 1.35) |  | 195.91 | -0.74 (-1.45, -0.03) |
| Iraq | 120.12 (81.54, 165.85) | 1.65 (1.14, 2.26) |  | 489.83 (330.08, 682.44) | 2.37 (1.64, 3.28) |  | 307.79 | 1.52 (1.05, 2.00) |
| Ireland | 22.63 (17.07, 28.62) | 0.55 (0.42, 0.68) |  | 110.60 (75.26, 153.74) | 1.46 (0.99, 2.03) |  | 388.70 | 4.26 (3.92, 4.60) |
| Israel | 55.83 (41.82, 69.93) | 1.14 (0.87, 1.42) |  | 148.36 (105.06, 203.91) | 1.27 (0.90, 1.74) |  | 165.71 | 0.30 (0.18, 0.42) |
| Italy | 3109.02 (2833.22, 3394.17) | 3.45 (3.14, 3.77) |  | 4437.88 (3567.82, 5463.13) | 3.14 (2.50, 3.92) |  | 42.74 | -0.75 (-0.94, -0.55) |
| Jamaica | 16.24 (11.30, 21.41) | 0.90 (0.62, 1.17) |  | 17.53 (11.57, 25.43) | 0.59 (0.38, 0.85) |  | 7.92 | -0.59 (-1.50, 0.32) |
| Japan | 15780.18 (14838.18, 16675.76) | 9.14 (8.59, 9.66) |  | 33311.75 (26824.34, 39444.35) | 8.60 (7.06, 10.25) |  | 111.10 | -1.08 (-1.62, -0.54) |
| Jordan | 12.11 (8.02, 17.04) | 1.10 (0.72, 1.51) |  | 48.45 (33.07, 66.24) | 0.89 (0.61, 1.20) |  | 300.23 | -0.90 (-1.04, -0.76) |
| Kazakhstan | 140.07 (102.10, 180.77) | 1.14 (0.84, 1.46) |  | 355.89 (247.15, 482.77) | 2.14 (1.50, 2.86) |  | 154.08 | -0.80 (-1.83, 0.24) |
| Kenya | 43.86 (29.88, 75.66) | 0.59 (0.41, 1.01) |  | 133.41 (87.32, 195.43) | 0.70 (0.47, 1.01) |  | 204.18 | 0.02 (-0.31, 0.34) |
| Kiribati | 1.05 (0.68, 1.54) | 3.07 (2.06, 4.33) |  | 1.68 (1.04, 2.46) | 2.72 (1.75, 3.93) |  | 60.55 | -0.37 (-0.43, -0.31) |
| Kuwait | 4.94 (3.49, 6.45) | 0.96 (0.69, 1.22) |  | 21.44 (14.79, 29.21) | 1.03 (0.73, 1.39) |  | 333.99 | 0.93 (0.67, 1.19) |
| Kyrgyzstan | 13.83 (10.28, 17.60) | 0.47 (0.35, 0.59) |  | 39.34 (28.01, 50.97) | 0.95 (0.68, 1.24) |  | 184.45 | 3.19 (2.91, 3.47) |
| Lao | 52.49 (29.62, 82.12) | 2.71 (1.61, 4.10) |  | 68.98 (43.41, 98.34) | 1.77 (1.15, 2.48) |  | 31.41 | -1.76 (-1.90, -1.62) |
| Latvia | 15.68 (11.10, 20.82) | 0.43 (0.31, 0.57) |  | 26.74 (18.15, 37.15) | 0.64 (0.43, 0.89) |  | 70.54 | 0.52 (-0.20, 1.24) |
| Lebanon | 16.19 (10.03, 23.94) | 0.78 (0.50, 1.14) |  | 46.13 (28.67, 72.45) | 0.88 (0.55, 1.38) |  | 184.95 | 0.45 (0.29, 0.61) |
| Lesotho | 14.26 (8.26, 26.06) | 1.58 (0.94, 2.84) |  | 41.59 (18.69, 70.72) | 3.57 (1.68, 5.91) |  | 191.73 | 3.33 (2.83, 3.83) |
| Liberia | 12.89 (8.06, 18.67) | 1.24 (0.81, 1.75) |  | 16.83 (10.16, 25.77) | 0.96 (0.57, 1.44) |  | 30.55 | -1.19 (-1.32, -1.05) |
| Libya | 31.35 (20.52, 46.00) | 1.82 (1.19, 2.64) |  | 86.55 (57.03, 127.95) | 1.83 (1.20, 2.66) |  | 176.04 | 0.16 (0.00, 0.32) |
| Lithuania | 19.90 (14.07, 25.88) | 0.44 (0.31, 0.57) |  | 42.53 (27.82, 58.26) | 0.71 (0.47, 0.98) |  | 113.75 | 0.83 (0.50, 1.15) |
| Luxembourg | 5.66 (4.15, 7.33) | 1.02 (0.75, 1.32) |  | 14.63 (10.07, 20.88) | 1.43 (0.98, 2.06) |  | 158.54 | 1.27 (0.97, 1.58) |
| Macedonia | 41.35 (28.36, 56.01) | 2.30 (1.60, 3.07) |  | 72.75 (47.57, 105.34) | 2.32 (1.56, 3.27) |  | 75.94 | -0.03 (-0.14, 0.08) |
| Madagascar | 30.51 (18.79, 49.52) | 0.65 (0.40, 1.05) |  | 56.31 (34.24, 86.62) | 0.61 (0.39, 0.93) |  | 84.57 | -0.51 (-0.61, -0.42) |
| Malawi | 25.41 (15.83, 38.96) | 0.73 (0.46, 1.08) |  | 41.52 (26.70, 57.81) | 0.68 (0.45, 0.93) |  | 63.42 | -1.05 (-1.34, -0.76) |
| Malaysia | 80.02 (50.94, 114.11) | 0.97 (0.62, 1.36) |  | 251.62 (158.74, 378.21) | 1.03 (0.67, 1.53) |  | 214.46 | 0.20 (-0.10, 0.50) |
| Maldives | 1.28 (0.70, 2.31) | 1.76 (1.00, 2.92) |  | 3.78 (2.51, 5.23) | 1.44 (0.95, 2.01) |  | 195.46 | -0.52 (-0.66, -0.38) |
| Mali | 210.23 (140.84, 292.51) | 5.14 (3.54, 6.98) |  | 408.50 (262.95, 587.57) | 4.90 (3.25, 6.81) |  | 94.31 | -0.38 (-0.49, -0.27) |
| Malta | 2.71 (2.02, 3.47) | 0.64 (0.48, 0.81) |  | 8.60 (6.16, 11.64) | 0.91 (0.66, 1.22) |  | 217.16 | 1.22 (1.01, 1.43) |
| Marshall Islands | 0.38 (0.23, 0.58) | 2.60 (1.64, 3.93) |  | 0.67 (0.38, 1.03) | 2.24 (1.36, 3.33) |  | 77.27 | -0.52 (-0.59, -0.45) |
| Mauritania | 12.68 (7.70, 18.42) | 1.35 (0.84, 1.93) |  | 15.58 (9.80, 22.51) | 0.83 (0.53, 1.19) |  | 22.94 | -1.68 (-1.81, -1.55) |
| Mauritius | 3.35 (2.33, 4.36) | 0.50 (0.36, 0.64) |  | 8.94 (5.85, 13.06) | 0.54 (0.35, 0.77) |  | 166.83 | 1.41 (1.01, 1.82) |
| Mexico | 382.57 (343.59, 419.86) | 0.98 (0.88, 1.07) |  | 1594.84 (1337.30, 1895.02) | 1.42 (1.19, 1.68) |  | 316.88 | 1.34 (1.16, 1.51) |
| Moldova | 14.14 (9.82, 19.36) | 0.33 (0.24, 0.45) |  | 30.16 (20.42, 42.73) | 0.52 (0.35, 0.73) |  | 113.35 | -0.14 (-1.67, 1.41) |
| Monaco | 0.91 (0.62, 1.25) | 1.26 (0.85, 1.74) |  | 3.17 (2.24, 4.24) | 3.28 (2.27, 4.41) |  | 247.21 | 4.02 (3.16, 4.89) |
| Mongolia | 205.58 (137.82, 287.93) | 20.42 (13.90, 28.35) |  | 656.64 (435.48, 924.52) | 35.02 (24.73, 46.77) |  | 219.41 | 2.85 (2.24, 3.47) |
| Montenegro | 8.53 (5.70, 11.87) | 1.40 (0.94, 1.92) |  | 14.43 (9.73, 20.63) | 1.46 (0.99, 2.05) |  | 69.18 | -0.01 (-0.24, 0.23) |
| Morocco | 81.49 (52.80, 113.75) | 0.70 (0.46, 0.95) |  | 216.43 (143.98, 296.55) | 0.80 (0.53, 1.08) |  | 165.59 | 0.33 (0.08, 0.58) |
| Mozambique | 20.10 (11.73, 31.87) | 0.41 (0.25, 0.66) |  | 55.28 (32.86, 82.39) | 0.62 (0.38, 0.92) |  | 175.11 | 1.32 (1.15, 1.49) |
| Myanmar | 182.12 (107.99, 291.29) | 0.90 (0.57, 1.39) |  | 547.54 (372.66, 745.44) | 1.30 (0.90, 1.73) |  | 200.65 | 1.16 (1.01, 1.31) |
| Namibia | 4.09 (2.49, 6.78) | 0.61 (0.38, 0.99) |  | 12.35 (8.01, 17.37) | 0.94 (0.63, 1.30) |  | 201.79 | 1.49 (1.21, 1.76) |
| Nauru | 0.07 (0.04, 0.11) | 2.29 (1.47, 3.27) |  | 0.07 (0.04, 0.11) | 1.99 (1.24, 3.04) |  | -5.15 | -0.60 (-0.86, -0.35) |
| Nepal | 48.99 (31.72, 71.86) | 0.61 (0.41, 0.87) |  | 148.23 (98.48, 232.08) | 0.75 (0.51, 1.14) |  | 202.59 | 0.72 (0.57, 0.86) |
| Netherlands | 91.14 (68.11, 116.77) | 0.45 (0.34, 0.58) |  | 355.36 (247.04, 494.61) | 1.03 (0.71, 1.43) |  | 289.89 | 3.16 (3.02, 3.30) |
| New Zealand | 24.53 (21.15, 28.48) | 0.63 (0.55, 0.73) |  | 108.44 (87.02, 134.14) | 1.39 (1.12, 1.72) |  | 342.14 | 2.87 (2.71, 3.03) |
| Nicaragua | 17.59 (12.80, 22.60) | 1.22 (0.89, 1.56) |  | 62.13 (44.42, 83.55) | 1.52 (1.10, 2.02) |  | 253.28 | 0.66 (0.25, 1.08) |
| Niger | 3.18 (2.03, 4.68) | 0.13 (0.09, 0.19) |  | 8.58 (5.23, 12.60) | 0.13 (0.08, 0.18) |  | 169.63 | -0.32 (-0.40, -0.24) |
| Nigeria | 307.14 (216.24, 419.06) | 0.78 (0.56, 1.06) |  | 557.55 (420.75, 717.92) | 0.75 (0.58, 0.95) |  | 81.53 | -0.03 (-0.12, 0.06) |
| Niue | 0.04 (0.03, 0.06) | 1.81 (1.21, 2.58) |  | 0.03 (0.02, 0.05) | 1.55 (1.01, 2.25) |  | -17.29 | -0.58 (-0.62, -0.55) |
| North Korea | 477.18 (298.79, 690.27) | 3.21 (2.07, 4.53) |  | 716.71 (460.90, 1018.61) | 2.27 (1.48, 3.21) |  | 50.20 | -1.41 (-1.49, -1.32) |
| Northern Mariana Islands | 0.22 (0.14, 0.32) | 1.50 (0.99, 2.08) |  | 0.71 (0.43, 1.06) | 1.45 (0.93, 2.02) |  | 230.11 | 0.05 (-0.11, 0.21) |
| Norway | 42.70 (37.81, 47.61) | 0.62 (0.55, 0.69) |  | 103.82 (83.72, 126.08) | 1.08 (0.87, 1.32) |  | 143.14 | 2.34 (2.18, 2.50) |
| Oman | 9.50 (5.93, 14.25) | 1.62 (1.04, 2.33) |  | 27.25 (18.47, 37.23) | 1.93 (1.35, 2.54) |  | 186.67 | 1.46 (1.10, 1.81) |
| Pakistan | 1034.33 (661.70, 1434.32) | 1.86 (1.18, 2.58) |  | 1868.87 (1399.55, 2382.19) | 1.79 (1.31, 2.24) |  | 80.68 | -0.20 (-0.35, -0.04) |
| Palau | 0.14 (0.08, 0.22) | 1.59 (0.96, 2.41) |  | 0.32 (0.20, 0.48) | 1.59 (1.03, 2.27) |  | 122.44 | 0.07 (0.01, 0.13) |
| Palestine | 28.41 (19.15, 40.29) | 3.61 (2.47, 5.00) |  | 55.37 (39.38, 72.70) | 2.79 (2.04, 3.63) |  | 94.91 | -0.85 (-0.92, -0.78) |
| Panama | 17.74 (13.35, 22.57) | 1.23 (0.92, 1.57) |  | 42.63 (28.82, 60.40) | 1.04 (0.70, 1.48) |  | 140.28 | -0.24 (-0.65, 0.17) |
| Papua New Guinea | 5.72 (3.57, 8.36) | 0.41 (0.26, 0.58) |  | 15.07 (9.65, 22.66) | 0.44 (0.29, 0.64) |  | 163.36 | 0.32 (0.29, 0.34) |
| Paraguay | 18.35 (13.29, 23.61) | 0.87 (0.63, 1.12) |  | 40.37 (27.15, 56.10) | 0.77 (0.52, 1.05) |  | 120.00 | -0.33 (-0.87, 0.22) |
| Peru | 44.70 (27.26, 67.18) | 0.41 (0.25, 0.60) |  | 60.47 (36.46, 95.69) | 0.19 (0.11, 0.30) |  | 35.28 | -3.52 (-4.09, -2.96) |
| Philippines | 546.75 (400.13, 702.92) | 2.07 (1.54, 2.62) |  | 1022.49 (805.69, 1294.69) | 1.42 (1.13, 1.78) |  | 87.01 | -1.55 (-1.86, -1.25) |
| Poland | 857.16 (742.75, 974.16) | 2.02 (1.76, 2.27) |  | 333.09 (268.74, 408.52) | 0.46 (0.37, 0.56) |  | -61.14 | -4.93 (-6.31, -3.53) |
| Portugal | 91.71 (69.25, 117.24) | 0.66 (0.51, 0.83) |  | 387.81 (268.01, 539.12) | 1.64 (1.11, 2.29) |  | 322.87 | 2.93 (2.63, 3.23) |
| Puerto Rico | 54.03 (37.33, 72.56) | 1.48 (1.03, 1.96) |  | 43.69 (27.95, 64.04) | 0.58 (0.37, 0.85) |  | -19.13 | -2.12 (-2.85, -1.39) |
| Qatar | 4.28 (2.75, 6.35) | 6.25 (4.02, 8.87) |  | 31.43 (19.17, 45.78) | 7.09 (4.79, 9.84) |  | 634.13 | 1.20 (0.97, 1.43) |
| Romania | 117.57 (81.44, 159.35) | 0.43 (0.31, 0.57) |  | 260.54 (174.53, 365.22) | 0.69 (0.46, 0.97) |  | 121.60 | 2.37 (1.93, 2.81) |
| Russia | 767.02 (656.94, 877.63) | 0.43 (0.37, 0.49) |  | 1757.36 (1417.55, 2117.11) | 0.74 (0.60, 0.89) |  | 129.12 | 2.38 (2.08, 2.68) |
| Rwanda | 29.87 (17.54, 47.95) | 1.15 (0.70, 1.75) |  | 55.15 (36.55, 78.70) | 1.05 (0.72, 1.48) |  | 84.61 | -0.86 (-1.05, -0.67) |
| Saint Kitts and Nevis | 1.16 (0.80, 1.57) | 3.06 (2.15, 4.07) |  | 0.50 (0.33, 0.74) | 0.84 (0.56, 1.18) |  | -56.83 | -4.68 (-5.74, -3.60) |
| Saint Lucia | 1.15 (0.81, 1.56) | 1.37 (0.98, 1.82) |  | 0.94 (0.63, 1.34) | 0.45 (0.30, 0.64) |  | -18.21 | -4.25 (-5.35, -3.14) |
| Saint Vincent and the Grenadines | 1.19 (0.84, 1.60) | 1.68 (1.19, 2.23) |  | 0.79 (0.53, 1.12) | 0.60 (0.41, 0.84) |  | -33.46 | -3.51 (-4.46, -2.55) |
| Samoa | 0.99 (0.62, 1.49) | 1.18 (0.77, 1.73) |  | 1.38 (0.86, 2.02) | 0.99 (0.63, 1.45) |  | 39.58 | -0.58 (-0.69, -0.48) |
| San Marino | 0.26 (0.18, 0.35) | 0.77 (0.55, 1.03) |  | 0.71 (0.48, 1.02) | 1.08 (0.73, 1.55) |  | 173.65 | 1.77 (1.55, 1.99) |
| Sao Tome and Principe | 0.28 (0.19, 0.39) | 0.47 (0.31, 0.65) |  | 0.43 (0.26, 0.67) | 0.47 (0.28, 0.72) |  | 54.04 | -0.00 (-0.13, 0.12) |
| Saudi Arabia | 95.72 (62.41, 133.94) | 1.91 (1.25, 2.64) |  | 284.18 (187.84, 399.23) | 2.11 (1.46, 2.88) |  | 196.87 | 0.32 (0.06, 0.58) |
| Senegal | 6.10 (3.65, 9.08) | 0.21 (0.13, 0.31) |  | 13.42 (7.88, 20.78) | 0.20 (0.12, 0.31) |  | 119.87 | 0.08 (-0.06, 0.23) |
| Serbia | 211.87 (145.32, 293.86) | 1.93 (1.37, 2.65) |  | 287.89 (194.29, 405.23) | 1.76 (1.22, 2.43) |  | 35.88 | -0.58 (-0.83, -0.33) |
| Seychelles | 1.08 (0.74, 1.46) | 1.91 (1.32, 2.60) |  | 1.36 (0.92, 1.89) | 1.32 (0.90, 1.79) |  | 26.73 | -1.01 (-1.27, -0.74) |
| Sierra Leone | 19.63 (12.33, 29.03) | 1.09 (0.70, 1.59) |  | 29.51 (18.11, 42.90) | 0.93 (0.58, 1.34) |  | 50.32 | -0.60 (-0.65, -0.56) |
| Singapore | 40.70 (27.30, 55.64) | 2.02 (1.37, 2.74) |  | 246.20 (156.20, 355.03) | 3.32 (2.11, 4.72) |  | 504.97 | 1.70 (1.43, 1.97) |
| Slovakia | 60.22 (41.65, 81.36) | 1.00 (0.70, 1.34) |  | 73.45 (46.68, 106.71) | 0.79 (0.51, 1.14) |  | 21.97 | -1.35 (-1.66, -1.03) |
| Slovenia | 18.06 (11.70, 26.67) | 0.73 (0.48, 1.08) |  | 60.30 (39.08, 86.00) | 1.33 (0.86, 1.92) |  | 233.84 | 2.50 (2.22, 2.77) |
| Solomon Islands | 1.69 (0.96, 2.61) | 1.32 (0.80, 1.94) |  | 3.12 (1.95, 4.46) | 1.15 (0.77, 1.61) |  | 85.21 | -0.46 (-0.54, -0.38) |
| Somalia | 18.60 (9.76, 32.32) | 0.86 (0.49, 1.43) |  | 47.72 (26.66, 84.50) | 0.87 (0.51, 1.56) |  | 156.61 | 0.18 (0.10, 0.25) |
| South Africa | 316.52 (203.23, 492.21) | 1.61 (1.02, 2.49) |  | 629.98 (534.14, 751.41) | 1.51 (1.29, 1.81) |  | 99.03 | -0.49 (-1.02, 0.04) |
| South Korea | 476.14 (313.81, 678.89) | 1.81 (1.23, 2.55) |  | 3548.78 (2361.20, 4873.81) | 3.98 (2.69, 5.45) |  | 645.33 | 3.47 (2.31, 4.65) |
| South Sudan | 15.91 (9.02, 27.33) | 0.74 (0.43, 1.26) |  | 22.55 (12.28, 39.14) | 0.68 (0.39, 1.15) |  | 41.74 | -0.23 (-0.28, -0.19) |
| Spain | 1040.87 (823.30, 1278.27) | 1.88 (1.49, 2.30) |  | 2839.26 (2022.48, 3751.14) | 2.97 (2.07, 3.98) |  | 172.78 | 1.48 (1.11, 1.84) |
| Sri Lanka | 49.89 (33.49, 68.44) | 0.53 (0.37, 0.71) |  | 173.27 (109.72, 258.51) | 0.72 (0.46, 1.04) |  | 247.28 | 1.95 (1.64, 2.26) |
| Sudan | 94.57 (51.09, 146.65) | 1.12 (0.61, 1.73) |  | 181.52 (102.36, 291.83) | 1.12 (0.64, 1.78) |  | 91.95 | -0.03 (-0.16, 0.09) |
| Suriname | 3.56 (2.48, 4.80) | 1.45 (1.01, 1.96) |  | 3.25 (2.15, 4.62) | 0.56 (0.38, 0.80) |  | -8.60 | -2.97 (-3.70, -2.23) |
| Sweden | 140.91 (120.15, 162.84) | 0.92 (0.78, 1.07) |  | 217.29 (172.30, 266.23) | 1.04 (0.82, 1.28) |  | 54.20 | 0.95 (0.32, 1.58) |
| Switzerland | 87.54 (63.50, 114.65) | 0.83 (0.60, 1.09) |  | 339.93 (228.52, 477.89) | 1.93 (1.29, 2.73) |  | 288.34 | 2.30 (1.75, 2.86) |
| Syrian Arab Republic | 82.96 (54.21, 118.49) | 1.80 (1.18, 2.55) |  | 186.01 (123.84, 264.18) | 1.73 (1.18, 2.42) |  | 124.22 | -0.19 (-0.32, -0.05) |
| Taiwan (Province of China) | 501.01 (365.06, 646.46) | 3.16 (2.32, 3.99) |  | 1158.80 (796.42, 1592.57) | 2.90 (1.97, 4.00) |  | 131.29 | -1.21 (-2.50, 0.09) |
| Tajikistan | 12.77 (10.04, 15.74) | 0.48 (0.37, 0.59) |  | 75.48 (54.50, 100.62) | 1.86 (1.39, 2.44) |  | 491.09 | 5.62 (5.36, 5.87) |
| Tanzania | 45.50 (30.05, 63.23) | 0.48 (0.33, 0.65) |  | 114.22 (76.82, 159.50) | 0.54 (0.36, 0.74) |  | 151.02 | 0.32 (0.22, 0.42) |
| Thailand | 1219.91 (815.67, 1709.93) | 3.79 (2.60, 5.22) |  | 4112.70 (2572.63, 6174.61) | 4.06 (2.57, 6.04) |  | 237.13 | 0.53 (0.39, 0.68) |
| Timor-Leste | 5.08 (3.00, 7.95) | 2.08 (1.30, 3.15) |  | 13.01 (8.13, 19.72) | 1.73 (1.12, 2.52) |  | 155.96 | -0.84 (-1.01, -0.67) |
| Togo | 13.77 (8.84, 19.55) | 1.26 (0.83, 1.76) |  | 30.99 (19.06, 44.69) | 1.01 (0.65, 1.42) |  | 125.09 | -1.09 (-1.18, -0.99) |
| Tokelau | 0.03 (0.02, 0.04) | 2.05 (1.31, 2.92) |  | 0.02 (0.01, 0.03) | 1.63 (1.06, 2.31) |  | -24.64 | -0.89 (-0.93, -0.85) |
| Tonga | 2.47 (1.53, 3.75) | 4.66 (2.95, 6.94) |  | 3.72 (2.34, 5.47) | 4.77 (3.02, 7.04) |  | 50.66 | 0.10 (-0.03, 0.22) |
| Trinidad and Tobago | 12.66 (8.69, 16.87) | 1.59 (1.11, 2.09) |  | 10.31 (6.49, 15.31) | 0.57 (0.36, 0.83) |  | -18.55 | -3.71 (-4.77, -2.63) |
| Tunisia | 41.23 (28.22, 55.69) | 0.88 (0.61, 1.18) |  | 115.27 (76.75, 168.21) | 0.95 (0.64, 1.37) |  | 179.61 | 0.45 (0.36, 0.53) |
| Turkey | 309.37 (199.93, 438.96) | 0.95 (0.61, 1.33) |  | 772.44 (528.01, 1032.24) | 0.92 (0.63, 1.23) |  | 149.68 | -0.02 (-0.42, 0.39) |
| Turkmenistan | 7.71 (5.75, 9.67) | 0.44 (0.34, 0.55) |  | 66.86 (43.09, 94.32) | 1.71 (1.12, 2.35) |  | 767.06 | 5.43 (4.39, 6.48) |
| Tuvalu | 0.15 (0.09, 0.24) | 2.44 (1.55, 3.62) |  | 0.18 (0.11, 0.27) | 1.87 (1.16, 2.73) |  | 16.29 | -1.06 (-1.20, -0.92) |
| Uganda | 53.28 (34.48, 76.01) | 0.90 (0.59, 1.25) |  | 165.48 (106.96, 233.62) | 1.31 (0.87, 1.85) |  | 210.60 | 1.41 (1.19, 1.63) |
| Ukraine | 205.15 (177.63, 234.04) | 0.29 (0.25, 0.33) |  | 533.84 (436.22, 650.38) | 0.68 (0.56, 0.84) |  | 160.22 | 3.62 (3.16, 4.08) |
| United Arab Emirates | 4.68 (2.32, 9.36) | 1.53 (0.72, 3.09) |  | 46.03 (17.02, 112.63) | 1.58 (0.63, 3.86) |  | 883.91 | -0.05 (-0.43, 0.33) |
| United Kingdom | 669.46 (596.97, 746.79) | 0.73 (0.65, 0.81) |  | 2340.87 (1911.41, 2840.49) | 1.79 (1.46, 2.17) |  | 249.67 | 3.94 (3.70, 4.18) |
| United States of America | 2484.81 (2234.58, 2716.01) | 0.78 (0.71, 0.86) |  | 10408.76 (8343.50, 12654.46) | 1.88 (1.51, 2.28) |  | 318.89 | 3.11 (2.83, 3.39) |
| United States Virgin Islands | 0.73 (0.49, 1.03) | 0.94 (0.64, 1.32) |  | 0.98 (0.64, 1.37) | 0.52 (0.35, 0.72) |  | 33.79 | -2.19 (-2.77, -1.60) |
| Uruguay | 18.96 (13.52, 24.61) | 0.48 (0.34, 0.62) |  | 43.77 (29.96, 60.16) | 0.78 (0.52, 1.09) |  | 130.89 | 2.11 (1.96, 2.26) |
| Uzbekistan | 35.53 (26.94, 44.30) | 0.34 (0.26, 0.42) |  | 410.69 (274.59, 564.09) | 2.26 (1.61, 3.00) |  | 1056.04 | 8.88 (7.69, 10.08) |
| Vanuatu | 1.16 (0.65, 1.96) | 2.00 (1.15, 3.32) |  | 3.02 (1.73, 4.62) | 1.92 (1.16, 2.88) |  | 161.39 | -0.11 (-0.19, -0.03) |
| Venezuela | 215.23 (160.12, 270.40) | 2.36 (1.77, 2.97) |  | 227.22 (151.97, 318.66) | 0.81 (0.55, 1.13) |  | 5.57 | -2.95 (-4.15, -1.74) |
| Viet Nam | 346.85 (192.22, 542.29) | 0.91 (0.52, 1.41) |  | 535.80 (308.48, 841.97) | 0.65 (0.38, 1.01) |  | 54.48 | -1.35 (-1.50, -1.19) |
| Yemen | 64.39 (37.33, 102.64) | 1.50 (0.87, 2.35) |  | 174.68 (114.85, 251.52) | 1.51 (1.02, 2.15) |  | 171.30 | 0.07 (-0.04, 0.18) |
| Zambia | 13.46 (8.54, 20.45) | 0.56 (0.36, 0.84) |  | 35.92 (22.94, 50.60) | 0.65 (0.43, 0.91) |  | 166.96 | -0.08 (-0.38, 0.22) |
| Zimbabwe | 139.49 (95.32, 202.13) | 3.69 (2.59, 5.26) |  | 325.32 (215.45, 461.60) | 5.02 (3.37, 7.07) |  | 133.22 | 0.34 (-0.11, 0.79) |

ASIR: age-standardized incidence rate; CI: confidence interval; EAPC: estimated annual percentage change; UI: uncertainty interval.


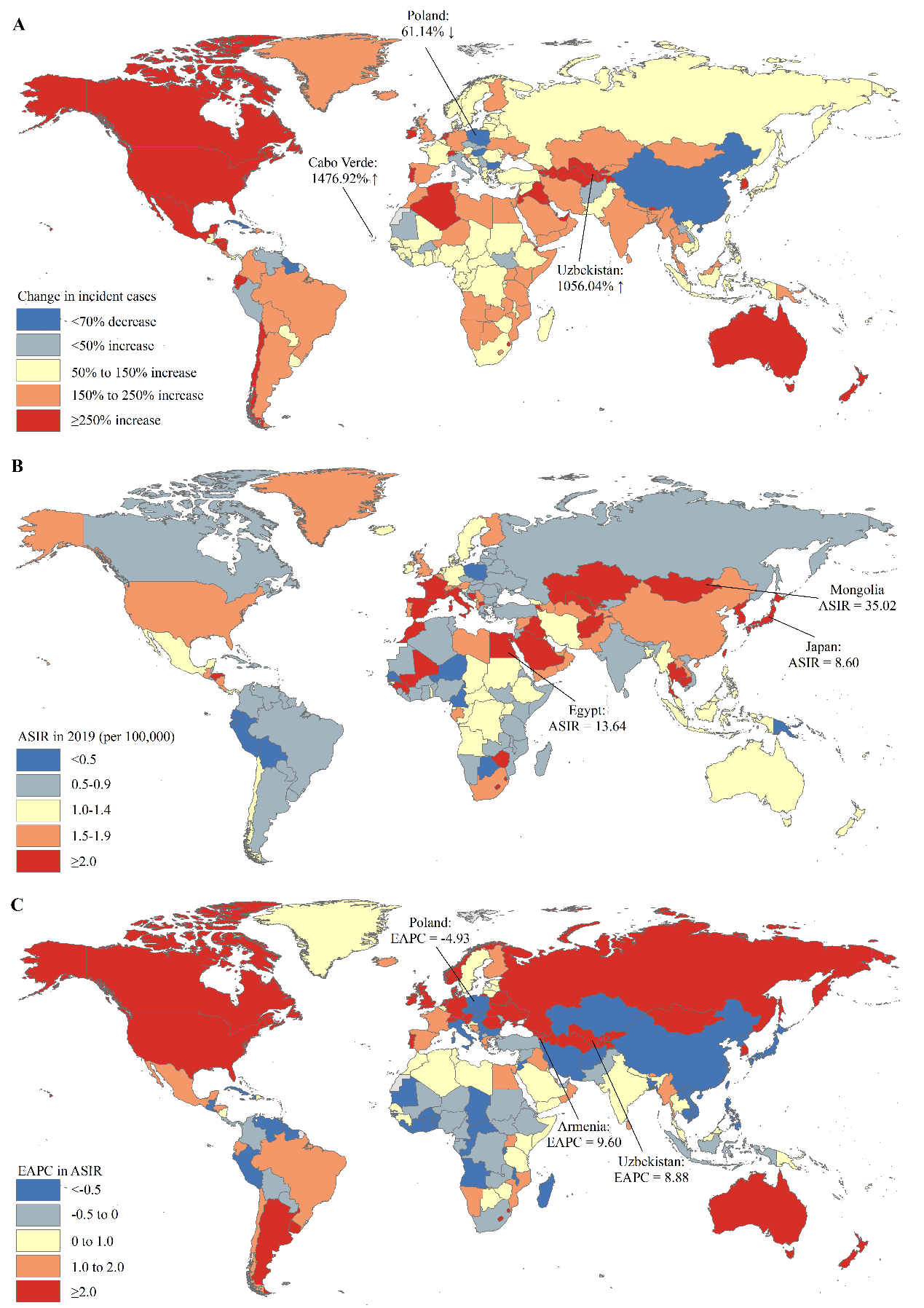


**Figure S2.** The global trends in the incidence of primary liver cancer due to hepatitis C in 204 countries and territories. (A) The percentage change in incident cases of primary liver cancer due to hepatitis C between 1990 and 2019; (B) The ASIR of primary liver cancer due to hepatitis C in 2019; (C) The EAPCs in ASIR of primary liver cancer due to hepatitis C from 1990 to 2019.

Note: ASIR: age-standardized incidence rate; EAPC: estimated annual percentage change.

**Table S15**. **The incident cases and ASIR of primary liver cancer due to alcohol use in 1990 and 2019 and their change trends from 1990 to 2019 at national level.**

| Nation | 1990 | |  | 2019 | |  | 1990-2019 | |
| --- | --- | --- | --- | --- | --- | --- | --- | --- |
|  | Incident cases  No. (95% UI) | ASIR per 100,000  No. (95% UI) |  | Incident cases  No. x 10^3^ (95% UI) | ASIR per 100,000  No. (95% UI) |  | Percentage change in incident cases  No. (%) | EAPC in ASIR  No. (95% CI) |
| Afghanistan | 68.58 (39.89, 107.33) | 0.95 (0.56, 1.47) |  | 101.78 (62.39, 158.24) | 0.83 (0.50, 1.26) |  | 48.41 | -0.55 (-0.59, -0.52) |
| Albania | 65.62 (47.56, 85.28) | 3.21 (2.31, 4.17) |  | 106.81 (66.76, 156.86) | 2.44 (1.53, 3.58) |  | 62.78 | -1.65 (-2.07, -1.23) |
| Algeria | 18.63 (11.12, 28.76) | 0.16 (0.09, 0.24) |  | 72.11 (42.55, 110.77) | 0.22 (0.13, 0.34) |  | 286.97 | 1.32 (1.20, 1.45) |
| American Samoa | 0.14 (0.08, 0.21) | 0.59 (0.35, 0.91) |  | 0.37 (0.23, 0.56) | 0.76 (0.48, 1.13) |  | 167.81 | 1.24 (1.08, 1.40) |
| Andorra | 2.27 (1.53, 3.37) | 3.99 (2.72, 5.85) |  | 6.01 (3.92, 8.42) | 4.37 (2.86, 6.13) |  | 164.74 | 0.29 (0.23, 0.35) |
| Angola | 13.33 (8.04, 20.99) | 0.35 (0.21, 0.55) |  | 45.20 (28.68, 67.32) | 0.41 (0.26, 0.61) |  | 239.17 | 0.65 (0.58, 0.73) |
| Antigua and Barbuda | 1.30 (0.94, 1.71) | 2.46 (1.76, 3.20) |  | 0.95 (0.67, 1.29) | 0.95 (0.67, 1.28) |  | -26.74 | -3.60 (-4.65, -2.55) |
| Argentina | 156.45 (108.88, 206.45) | 0.48 (0.33, 0.63) |  | 366.65 (241.89, 510.09) | 0.68 (0.45, 0.94) |  | 134.36 | 1.94 (1.63, 2.25) |
| Armenia | 6.63 (4.65, 8.97) | 0.25 (0.18, 0.34) |  | 80.89 (56.13, 110.37) | 1.94 (1.35, 2.66) |  | 1120.25 | 10.54 (8.91, 12.20) |
| Australia | 164.51 (126.14, 200.46) | 0.83 (0.64, 1.01) |  | 706.96 (485.52, 988.86) | 1.78 (1.21, 2.50) |  | 329.73 | 3.09 (2.77, 3.40) |
| Austria | 210.24 (173.97, 244.89) | 1.84 (1.53, 2.14) |  | 499.30 (365.98, 662.58) | 2.94 (2.14, 3.90) |  | 137.49 | 1.84 (1.51, 2.16) |
| Azerbaijan | 10.67 (7.35, 14.56) | 0.22 (0.15, 0.30) |  | 98.02 (60.93, 149.72) | 1.08 (0.66, 1.65) |  | 818.96 | 6.65 (4.66, 8.69) |
| Bahamas | 4.70 (3.44, 6.10) | 3.03 (2.22, 3.93) |  | 4.10 (2.83, 5.72) | 1.04 (0.72, 1.44) |  | -12.64 | -3.94 (-4.92, -2.95) |
| Bahrain | 1.01 (0.62, 1.53) | 0.65 (0.40, 0.97) |  | 4.46 (2.65, 7.04) | 0.53 (0.32, 0.83) |  | 341.30 | -1.51 (-1.85, -1.17) |
| Bangladesh | 324.43 (216.27, 466.46) | 0.67 (0.44, 0.96) |  | 707.14 (440.78, 1041.51) | 0.54 (0.33, 0.79) |  | 117.97 | -1.23 (-1.40, -1.07) |
| Barbados | 2.65 (1.68, 4.00) | 0.90 (0.57, 1.37) |  | 4.72 (3.30, 6.28) | 0.95 (0.67, 1.26) |  | 78.20 | -0.02 (-0.37, 0.32) |
| Belarus | 58.42 (43.41, 72.52) | 0.45 (0.34, 0.55) |  | 164.80 (105.98, 241.40) | 1.04 (0.67, 1.53) |  | 182.08 | 3.82 (3.01, 4.63) |
| Belgium | 161.49 (121.66, 201.86) | 1.04 (0.79, 1.29) |  | 358.25 (242.45, 494.94) | 1.67 (1.13, 2.33) |  | 121.85 | 1.56 (1.29, 1.84) |
| Belize | 1.68 (1.22, 2.20) | 1.83 (1.33, 2.39) |  | 2.83 (2.04, 3.72) | 1.06 (0.77, 1.38) |  | 68.10 | -2.06 (-2.66, -1.45) |
| Benin | 22.99 (14.92, 35.42) | 1.16 (0.76, 1.79) |  | 44.18 (27.38, 69.19) | 0.93 (0.58, 1.45) |  | 92.18 | -1.14 (-1.32, -0.96) |
| Bermuda | 1.72 (1.28, 2.17) | 2.74 (2.04, 3.47) |  | 1.04 (0.74, 1.40) | 0.80 (0.57, 1.07) |  | -39.26 | -4.15 (-5.25, -3.05) |
| Bhutan | 1.62 (0.92, 2.71) | 0.63 (0.37, 1.04) |  | 4.86 (2.80, 7.74) | 0.87 (0.51, 1.39) |  | 200.49 | 1.08 (0.99, 1.16) |
| Bolivia | 41.81 (26.16, 62.48) | 1.35 (0.85, 2.01) |  | 121.30 (78.21, 176.08) | 1.42 (0.92, 2.05) |  | 190.10 | 0.17 (0.01, 0.33) |
| Bosnia and Herzegovina | 75.30 (54.11, 98.31) | 1.81 (1.32, 2.35) |  | 159.04 (108.27, 221.62) | 2.59 (1.78, 3.59) |  | 111.21 | 1.68 (1.42, 1.94) |
| Botswana | 1.03 (0.38, 2.98) | 0.18 (0.07, 0.50) |  | 4.59 (2.74, 7.28) | 0.31 (0.19, 0.48) |  | 344.07 | 0.99 (0.03, 1.96) |
| Brazil | 484.23 (418.48, 550.98) | 0.54 (0.47, 0.61) |  | 1711.75 (1482.70, 1942.46) | 0.72 (0.62, 0.81) |  | 253.50 | 1.47 (1.31, 1.63) |
| Brunei | 0.88 (0.54, 1.33) | 0.92 (0.56, 1.39) |  | 3.43 (2.08, 5.36) | 1.08 (0.67, 1.60) |  | 289.89 | 1.42 (0.94, 1.90) |
| Bulgaria | 323.72 (245.27, 401.10) | 2.49 (1.92, 3.08) |  | 282.07 (204.18, 379.86) | 2.02 (1.46, 2.75) |  | -12.87 | -0.03 (-0.57, 0.50) |
| Burkina Faso | 18.77 (11.73, 27.54) | 0.44 (0.28, 0.64) |  | 32.28 (20.35, 47.11) | 0.37 (0.23, 0.53) |  | 72.02 | -0.89 (-1.09, -0.69) |
| Burundi | 28.10 (18.09, 41.15) | 1.20 (0.77, 1.76) |  | 37.32 (20.69, 66.99) | 0.83 (0.48, 1.47) |  | 32.81 | -1.91 (-2.14, -1.67) |
| Cambodia | 53.59 (34.46, 80.40) | 1.18 (0.75, 1.79) |  | 182.00 (115.17, 264.15) | 1.52 (0.97, 2.19) |  | 239.60 | 0.58 (0.41, 0.75) |
| Cameroon | 5.27 (2.98, 9.12) | 0.12 (0.07, 0.21) |  | 16.30 (9.95, 25.27) | 0.14 (0.09, 0.21) |  | 209.51 | 0.33 (0.12, 0.53) |
| Canada | 369.99 (317.53, 420.47) | 1.14 (0.98, 1.30) |  | 1741.78 (1223.58, 2341.35) | 2.64 (1.85, 3.53) |  | 370.77 | 3.23 (3.05, 3.42) |
| Cape Verde | 0.42 (0.27, 0.60) | 0.18 (0.12, 0.26) |  | 9.33 (6.39, 12.76) | 2.23 (1.50, 3.07) |  | 2124.69 | 6.69 (4.50, 8.92) |
| Central African Republic | 6.80 (3.80, 10.84) | 0.57 (0.33, 0.89) |  | 10.91 (5.87, 19.09) | 0.50 (0.28, 0.87) |  | 60.30 | -1.00 (-1.26, -0.74) |
| Chad | 25.89 (15.05, 39.91) | 0.91 (0.54, 1.43) |  | 46.27 (28.24, 70.62) | 0.85 (0.52, 1.30) |  | 78.74 | -0.41 (-0.48, -0.34) |
| Chile | 67.24 (48.92, 87.64) | 0.67 (0.48, 0.87) |  | 225.99 (147.58, 319.00) | 0.93 (0.61, 1.32) |  | 236.10 | 1.80 (1.40, 2.21) |
| China | 17553.60 (12726.04, 23390.03) | 1.95 (1.44, 2.59) |  | 19187.81 (14314.77, 25624.93) | 0.92 (0.69, 1.22) |  | 9.31 | -4.10 (-4.98, -3.22) |
| Colombia | 143.26 (103.68, 183.80) | 0.82 (0.60, 1.06) |  | 417.44 (277.40, 603.41) | 0.80 (0.53, 1.15) |  | 191.38 | 0.13 (-0.40, 0.67) |
| Comoros | 1.41 (0.66, 2.94) | 0.63 (0.30, 1.31) |  | 2.71 (1.48, 5.25) | 0.56 (0.31, 1.09) |  | 92.18 | -0.54 (-0.71, -0.38) |
| Congo | 6.60 (3.83, 10.62) | 0.61 (0.35, 0.95) |  | 12.54 (7.39, 19.83) | 0.48 (0.30, 0.74) |  | 90.18 | -1.16 (-1.33, -0.98) |
| Cook Islands | 0.20 (0.12, 0.29) | 1.56 (0.96, 2.25) |  | 0.46 (0.29, 0.67) | 1.84 (1.18, 2.68) |  | 128.74 | 0.87 (0.75, 1.00) |
| Costa Rica | 31.74 (23.52, 40.27) | 1.83 (1.34, 2.34) |  | 90.69 (61.22, 129.33) | 1.78 (1.20, 2.53) |  | 185.77 | -0.32 (-0.93, 0.28) |
| Cote d'Ivoire | 57.22 (33.84, 86.58) | 1.44 (0.88, 2.21) |  | 102.70 (62.31, 159.74) | 1.00 (0.62, 1.55) |  | 79.49 | -2.24 (-2.59, -1.89) |
| Croatia | 87.21 (67.01, 106.57) | 1.34 (1.05, 1.63) |  | 152.02 (107.45, 203.50) | 1.75 (1.22, 2.35) |  | 74.31 | 1.63 (1.17, 2.09) |
| Cuba | 213.70 (159.06, 271.05) | 2.06 (1.53, 2.61) |  | 165.27 (113.20, 226.01) | 0.87 (0.59, 1.19) |  | -22.66 | -3.17 (-4.39, -1.93) |
| Cyprus | 9.34 (6.43, 12.54) | 1.11 (0.78, 1.47) |  | 26.81 (18.89, 35.33) | 1.36 (0.97, 1.81) |  | 187.14 | 0.97 (0.79, 1.15) |
| Czech Republic | 275.39 (219.55, 328.06) | 1.97 (1.57, 2.34) |  | 302.41 (223.40, 394.53) | 1.43 (1.05, 1.87) |  | 9.81 | -1.31 (-1.47, -1.14) |
| Democratic Republic of the Congo | 51.58 (32.71, 75.71) | 0.33 (0.21, 0.47) |  | 102.81 (60.72, 157.34) | 0.28 (0.17, 0.42) |  | 99.30 | -0.67 (-0.77, -0.58) |
| Denmark | 77.28 (58.39, 96.43) | 0.97 (0.74, 1.20) |  | 174.92 (118.33, 243.02) | 1.58 (1.06, 2.23) |  | 126.35 | 2.03 (1.80, 2.26) |
| Djibouti | 1.04 (0.49, 2.29) | 0.74 (0.37, 1.58) |  | 4.55 (2.34, 8.80) | 0.78 (0.42, 1.49) |  | 338.90 | 0.09 (-0.06, 0.24) |
| Dominica | 2.04 (1.46, 2.68) | 2.82 (2.02, 3.71) |  | 1.04 (0.71, 1.44) | 1.15 (0.79, 1.59) |  | -49.21 | -3.37 (-4.19, -2.54) |
| Dominican Republic | 47.18 (34.38, 61.79) | 1.26 (0.91, 1.65) |  | 150.80 (89.94, 254.96) | 1.63 (0.97, 2.76) |  | 219.66 | 1.06 (0.59, 1.53) |
| Ecuador | 36.91 (26.16, 49.81) | 0.72 (0.50, 0.98) |  | 149.11 (99.16, 215.94) | 1.01 (0.68, 1.45) |  | 303.98 | 1.81 (1.45, 2.16) |
| Egypt | 381.73 (225.14, 598.15) | 1.24 (0.73, 1.93) |  | 1218.74 (662.06, 2118.05) | 1.79 (0.99, 3.07) |  | 219.27 | 2.17 (1.74, 2.59) |
| El Salvador | 25.49 (18.20, 33.34) | 0.86 (0.62, 1.13) |  | 36.68 (23.67, 52.75) | 0.63 (0.40, 0.91) |  | 43.89 | -1.36 (-1.93, -0.78) |
| Equatorial Guinea | 0.83 (0.49, 1.30) | 0.41 (0.24, 0.63) |  | 2.67 (1.35, 4.47) | 0.57 (0.29, 0.95) |  | 222.14 | 1.24 (1.04, 1.44) |
| Eritrea | 7.23 (3.87, 13.02) | 0.72 (0.39, 1.28) |  | 18.55 (10.32, 31.91) | 0.70 (0.39, 1.17) |  | 156.69 | -0.55 (-0.74, -0.35) |
| Estonia | 15.03 (11.14, 18.88) | 0.73 (0.54, 0.91) |  | 42.32 (29.92, 55.96) | 1.67 (1.18, 2.23) |  | 181.64 | 1.98 (1.48, 2.49) |
| Eswatini | 3.16 (1.59, 7.48) | 1.05 (0.53, 2.50) |  | 26.98 (6.49, 55.83) | 4.38 (1.09, 8.93) |  | 753.40 | 5.97 (4.51, 7.45) |
| Ethiopia | 129.19 (87.43, 187.68) | 0.65 (0.44, 0.91) |  | 247.47 (183.15, 330.95) | 0.63 (0.47, 0.85) |  | 91.55 | -0.28 (-0.38, -0.17) |
| Federated States of Micronesia | 0.64 (0.37, 1.02) | 1.34 (0.78, 2.14) |  | 0.96 (0.50, 1.58) | 1.29 (0.72, 2.03) |  | 48.99 | -0.19 (-0.34, -0.05) |
| Fiji | 2.53 (1.55, 3.90) | 0.70 (0.42, 1.07) |  | 6.27 (3.69, 9.94) | 0.81 (0.49, 1.26) |  | 147.60 | 1.11 (0.75, 1.47) |
| Finland | 85.85 (63.99, 107.97) | 1.20 (0.90, 1.50) |  | 256.84 (176.06, 358.78) | 2.11 (1.44, 2.92) |  | 199.16 | 2.47 (2.23, 2.71) |
| France | 1506.66 (1097.93, 1915.76) | 1.89 (1.38, 2.39) |  | 2839.43 (1841.22, 4082.94) | 2.27 (1.48, 3.32) |  | 88.46 | 0.34 (0.13, 0.56) |
| Gabon | 3.23 (1.85, 4.97) | 0.56 (0.33, 0.86) |  | 6.27 (3.29, 11.15) | 0.60 (0.32, 1.04) |  | 94.40 | -0.00 (-0.13, 0.12) |
| Gambia | 19.32 (12.13, 29.51) | 5.18 (3.29, 7.79) |  | 73.13 (42.25, 111.04) | 7.37 (4.25, 11.17) |  | 278.45 | 0.94 (0.71, 1.18) |
| Georgia | 17.80 (12.09, 23.85) | 0.29 (0.20, 0.38) |  | 67.57 (47.33, 90.65) | 1.18 (0.83, 1.59) |  | 279.56 | 4.16 (2.84, 5.49) |
| Germany | 1393.18 (1123.39, 1671.72) | 1.11 (0.90, 1.33) |  | 4399.78 (3178.43, 5988.22) | 2.42 (1.74, 3.30) |  | 215.81 | 3.09 (2.67, 3.51) |
| Ghana | 70.64 (44.14, 110.90) | 1.14 (0.71, 1.75) |  | 203.98 (130.14, 299.46) | 1.27 (0.82, 1.85) |  | 188.74 | 0.14 (-0.07, 0.35) |
| Greece | 116.96 (86.74, 150.28) | 0.75 (0.55, 0.96) |  | 273.71 (188.28, 383.66) | 1.18 (0.82, 1.67) |  | 134.01 | 1.82 (1.67, 1.97) |
| Greenland | 0.71 (0.52, 0.93) | 1.84 (1.37, 2.37) |  | 1.94 (1.32, 2.69) | 2.64 (1.83, 3.62) |  | 172.87 | 1.54 (1.44, 1.64) |
| Grenada | 2.12 (1.55, 2.74) | 2.99 (2.17, 3.89) |  | 1.27 (0.93, 1.65) | 1.13 (0.83, 1.45) |  | -39.93 | -3.78 (-4.69, -2.86) |
| Guam | 0.35 (0.22, 0.51) | 0.44 (0.28, 0.65) |  | 1.42 (0.86, 2.17) | 0.73 (0.45, 1.09) |  | 310.67 | 2.31 (2.13, 2.48) |
| Guatemala | 82.27 (57.33, 111.79) | 2.27 (1.60, 3.06) |  | 123.89 (80.66, 180.15) | 1.13 (0.73, 1.64) |  | 50.59 | -3.12 (-4.44, -1.78) |
| Guinea | 159.77 (102.70, 237.43) | 4.81 (3.11, 7.15) |  | 313.00 (180.87, 476.31) | 5.74 (3.32, 8.62) |  | 95.91 | 0.78 (0.74, 0.82) |
| Guinea-Bissau | 5.63 (3.33, 9.17) | 1.38 (0.83, 2.21) |  | 7.80 (4.69, 12.05) | 1.09 (0.67, 1.66) |  | 38.44 | -1.03 (-1.11, -0.95) |
| Guyana | 8.74 (6.23, 11.47) | 2.29 (1.65, 3.03) |  | 6.51 (4.40, 9.14) | 1.05 (0.71, 1.46) |  | -25.52 | -3.01 (-3.90, -2.11) |
| Haiti | 67.78 (39.07, 107.27) | 2.11 (1.21, 3.37) |  | 92.15 (49.29, 157.98) | 1.37 (0.74, 2.31) |  | 35.96 | -1.66 (-1.87, -1.45) |
| Honduras | 59.90 (19.16, 99.58) | 2.96 (0.91, 4.92) |  | 252.25 (104.36, 413.56) | 4.22 (1.75, 6.85) |  | 321.12 | 1.62 (1.44, 1.79) |
| Hungary | 425.65 (340.00, 504.71) | 2.84 (2.28, 3.37) |  | 209.13 (149.01, 276.68) | 1.09 (0.78, 1.45) |  | -50.87 | -2.52 (-3.25, -1.79) |
| Iceland | 1.81 (1.29, 2.38) | 0.64 (0.46, 0.85) |  | 6.90 (4.98, 8.97) | 1.29 (0.94, 1.68) |  | 281.60 | 2.43 (2.33, 2.53) |
| India | 2841.37 (2183.58, 3558.86) | 0.65 (0.49, 0.80) |  | 8285.27 (6535.37, 10413.18) | 0.72 (0.57, 0.90) |  | 191.59 | 0.40 (0.34, 0.46) |
| Indonesia | 406.91 (319.18, 504.16) | 0.42 (0.33, 0.51) |  | 844.04 (649.01, 1072.87) | 0.40 (0.31, 0.50) |  | 107.43 | -0.22 (-0.29, -0.14) |
| Iran | 114.97 (82.62, 154.15) | 0.47 (0.34, 0.60) |  | 279.03 (211.20, 363.97) | 0.41 (0.30, 0.53) |  | 142.70 | -0.89 (-1.52, -0.26) |
| Iraq | 31.26 (18.79, 47.96) | 0.41 (0.25, 0.64) |  | 129.51 (78.48, 198.24) | 0.59 (0.35, 0.89) |  | 314.26 | 1.37 (0.92, 1.82) |
| Ireland | 21.29 (15.72, 27.26) | 0.51 (0.38, 0.65) |  | 110.43 (74.51, 154.50) | 1.48 (1.00, 2.08) |  | 418.71 | 4.25 (3.87, 4.63) |
| Israel | 28.35 (19.30, 38.82) | 0.57 (0.39, 0.78) |  | 88.96 (57.21, 130.48) | 0.78 (0.50, 1.16) |  | 213.73 | 1.08 (1.01, 1.16) |
| Italy | 1673.05 (1447.73, 1896.78) | 1.88 (1.63, 2.13) |  | 2058.61 (1556.00, 2672.59) | 1.60 (1.20, 2.10) |  | 23.05 | -0.91 (-1.14, -0.67) |
| Jamaica | 22.28 (16.66, 29.01) | 1.25 (0.93, 1.61) |  | 25.62 (17.49, 35.46) | 0.87 (0.60, 1.21) |  | 14.99 | -0.32 (-1.28, 0.66) |
| Japan | 3198.84 (2708.39, 3703.75) | 1.84 (1.56, 2.13) |  | 5040.29 (3874.14, 6362.20) | 1.48 (1.14, 1.88) |  | 57.57 | -1.55 (-2.00, -1.10) |
| Jordan | 3.67 (2.22, 5.62) | 0.30 (0.18, 0.45) |  | 16.21 (9.61, 25.43) | 0.27 (0.16, 0.42) |  | 341.39 | -0.41 (-0.52, -0.29) |
| Kazakhstan | 137.29 (99.43, 175.19) | 1.07 (0.78, 1.36) |  | 354.68 (249.02, 465.11) | 2.01 (1.43, 2.63) |  | 158.35 | -1.53 (-2.74, -0.31) |
| Kenya | 54.08 (33.24, 100.38) | 0.66 (0.41, 1.22) |  | 166.85 (102.31, 262.50) | 0.76 (0.47, 1.21) |  | 208.53 | -0.29 (-0.76, 0.18) |
| Kiribati | 0.55 (0.32, 0.85) | 1.37 (0.82, 2.10) |  | 0.89 (0.52, 1.35) | 1.17 (0.70, 1.74) |  | 62.58 | -0.56 (-0.61, -0.51) |
| Kuwait | 1.65 (1.06, 2.42) | 0.28 (0.18, 0.42) |  | 6.19 (3.76, 9.56) | 0.28 (0.17, 0.43) |  | 274.45 | 0.58 (0.35, 0.81) |
| Kyrgyzstan | 9.66 (6.77, 12.87) | 0.32 (0.23, 0.43) |  | 34.21 (24.06, 45.41) | 0.77 (0.53, 1.02) |  | 254.23 | 3.86 (3.39, 4.33) |
| Lao | 43.23 (25.28, 68.85) | 2.01 (1.20, 3.14) |  | 70.34 (43.44, 106.48) | 1.60 (1.02, 2.38) |  | 62.73 | -1.08 (-1.26, -0.90) |
| Latvia | 20.63 (15.48, 25.96) | 0.57 (0.43, 0.72) |  | 43.82 (31.67, 57.75) | 1.15 (0.84, 1.52) |  | 112.38 | 1.55 (0.79, 2.32) |
| Lebanon | 9.86 (6.07, 14.81) | 0.43 (0.27, 0.65) |  | 22.98 (13.41, 37.86) | 0.44 (0.26, 0.73) |  | 132.97 | 0.17 (0.01, 0.34) |
| Lesotho | 9.58 (4.49, 23.58) | 0.95 (0.45, 2.32) |  | 44.47 (14.60, 74.96) | 3.37 (1.15, 5.71) |  | 364.36 | 4.73 (3.91, 5.55) |
| Liberia | 14.09 (8.71, 22.15) | 1.26 (0.78, 1.95) |  | 18.38 (10.97, 29.67) | 0.93 (0.56, 1.51) |  | 30.49 | -1.26 (-1.42, -1.11) |
| Libya | 8.72 (5.25, 13.59) | 0.48 (0.29, 0.75) |  | 24.65 (14.56, 40.13) | 0.49 (0.28, 0.80) |  | 182.76 | 0.25 (0.10, 0.39) |
| Lithuania | 26.28 (19.37, 32.56) | 0.58 (0.43, 0.72) |  | 78.79 (55.34, 102.41) | 1.46 (1.03, 1.90) |  | 199.85 | 2.51 (2.20, 2.83) |
| Luxembourg | 6.57 (4.95, 8.14) | 1.20 (0.90, 1.49) |  | 15.39 (10.46, 21.44) | 1.59 (1.08, 2.22) |  | 134.05 | 1.02 (0.75, 1.28) |
| Macedonia | 59.23 (43.37, 76.14) | 3.13 (2.31, 4.00) |  | 101.81 (68.19, 142.53) | 3.06 (2.07, 4.31) |  | 71.89 | -0.24 (-0.37, -0.12) |
| Madagascar | 35.53 (18.26, 73.85) | 0.70 (0.35, 1.48) |  | 62.70 (33.56, 115.77) | 0.59 (0.32, 1.07) |  | 76.49 | -0.87 (-1.02, -0.73) |
| Malawi | 28.38 (16.11, 49.41) | 0.73 (0.42, 1.26) |  | 49.36 (32.19, 70.55) | 0.70 (0.46, 1.00) |  | 73.93 | -1.22 (-1.64, -0.80) |
| Malaysia | 64.61 (42.03, 95.26) | 0.72 (0.46, 1.08) |  | 221.05 (136.15, 343.78) | 0.83 (0.50, 1.28) |  | 242.12 | 0.49 (0.24, 0.75) |
| Maldives | 1.20 (0.65, 2.14) | 1.42 (0.81, 2.45) |  | 3.57 (2.32, 5.17) | 1.23 (0.80, 1.79) |  | 197.29 | -0.39 (-0.52, -0.26) |
| Mali | 104.53 (63.96, 156.37) | 2.37 (1.48, 3.51) |  | 224.54 (136.71, 347.97) | 2.49 (1.51, 3.77) |  | 114.80 | 0.11 (-0.01, 0.23) |
| Malta | 2.24 (1.62, 2.92) | 0.51 (0.37, 0.67) |  | 8.05 (5.59, 10.94) | 0.88 (0.62, 1.18) |  | 259.05 | 1.89 (1.68, 2.10) |
| Marshall Islands | 0.23 (0.13, 0.36) | 1.37 (0.79, 2.20) |  | 0.47 (0.26, 0.77) | 1.30 (0.77, 2.07) |  | 105.10 | -0.02 (-0.11, 0.08) |
| Mauritania | 9.93 (6.15, 15.44) | 0.98 (0.61, 1.50) |  | 12.41 (7.30, 19.23) | 0.62 (0.37, 0.95) |  | 25.04 | -1.57 (-1.73, -1.40) |
| Mauritius | 2.82 (1.92, 3.87) | 0.38 (0.26, 0.52) |  | 7.95 (4.90, 11.96) | 0.45 (0.29, 0.67) |  | 181.95 | 1.87 (1.42, 2.31) |
| Mexico | 249.86 (217.31, 284.03) | 0.60 (0.52, 0.68) |  | 1222.84 (993.91, 1482.92) | 1.06 (0.86, 1.28) |  | 389.40 | 2.01 (1.88, 2.13) |
| Moldova | 36.59 (30.21, 42.54) | 0.81 (0.68, 0.95) |  | 64.77 (50.94, 80.80) | 1.12 (0.88, 1.39) |  | 77.01 | -1.41 (-3.01, 0.21) |
| Monaco | 0.79 (0.52, 1.10) | 1.18 (0.79, 1.64) |  | 2.83 (1.93, 3.91) | 3.20 (2.19, 4.44) |  | 258.51 | 4.14 (3.26, 5.02) |
| Mongolia | 179.05 (112.36, 255.61) | 16.95 (10.71, 23.76) |  | 714.29 (468.21, 1045.62) | 31.82 (21.31, 44.72) |  | 298.93 | 2.82 (2.41, 3.23) |
| Montenegro | 16.00 (11.69, 20.63) | 2.55 (1.88, 3.30) |  | 24.86 (17.59, 33.82) | 2.48 (1.76, 3.34) |  | 55.42 | -0.17 (-0.31, -0.03) |
| Morocco | 26.99 (15.92, 42.23) | 0.21 (0.12, 0.32) |  | 72.62 (43.77, 111.96) | 0.25 (0.15, 0.38) |  | 169.08 | 0.43 (0.11, 0.75) |
| Mozambique | 24.75 (13.56, 48.12) | 0.44 (0.25, 0.86) |  | 94.66 (54.45, 138.85) | 0.91 (0.52, 1.33) |  | 282.44 | 2.51 (2.25, 2.77) |
| Myanmar | 110.59 (66.91, 188.08) | 0.49 (0.30, 0.81) |  | 375.83 (240.54, 548.71) | 0.83 (0.54, 1.19) |  | 239.83 | 1.76 (1.61, 1.92) |
| Namibia | 2.63 (1.22, 6.01) | 0.36 (0.16, 0.80) |  | 11.50 (7.29, 16.76) | 0.80 (0.51, 1.15) |  | 337.11 | 2.93 (2.44, 3.43) |
| Nauru | 0.05 (0.03, 0.08) | 1.26 (0.75, 1.93) |  | 0.05 (0.03, 0.09) | 1.20 (0.68, 1.85) |  | 8.01 | -0.32 (-0.62, -0.02) |
| Nepal | 41.12 (27.73, 59.05) | 0.46 (0.31, 0.67) |  | 125.13 (73.79, 208.14) | 0.58 (0.34, 0.95) |  | 204.31 | 0.93 (0.78, 1.08) |
| Netherlands | 115.06 (91.21, 139.92) | 0.58 (0.46, 0.70) |  | 439.65 (312.12, 596.55) | 1.33 (0.94, 1.82) |  | 282.10 | 3.25 (3.08, 3.43) |
| New Zealand | 37.57 (32.41, 42.26) | 0.98 (0.84, 1.10) |  | 137.67 (106.97, 176.03) | 1.88 (1.46, 2.39) |  | 266.42 | 2.44 (2.25, 2.64) |
| Nicaragua | 13.14 (9.05, 17.64) | 0.86 (0.58, 1.15) |  | 51.04 (34.46, 71.38) | 1.17 (0.79, 1.64) |  | 288.32 | 1.23 (0.77, 1.70) |
| Niger | 2.86 (1.73, 4.32) | 0.10 (0.06, 0.16) |  | 7.43 (4.36, 11.46) | 0.10 (0.06, 0.15) |  | 159.64 | -0.38 (-0.48, -0.29) |
| Nigeria | 279.10 (196.20, 384.87) | 0.65 (0.46, 0.89) |  | 574.62 (429.10, 759.55) | 0.73 (0.55, 0.95) |  | 105.88 | 0.47 (0.39, 0.54) |
| Niue | 0.02 (0.01, 0.03) | 0.99 (0.61, 1.48) |  | 0.02 (0.01, 0.03) | 1.02 (0.64, 1.54) |  | 6.10 | -0.05 (-0.14, 0.04) |
| North Korea | 182.65 (108.08, 297.85) | 1.05 (0.65, 1.66) |  | 270.79 (154.80, 434.61) | 0.82 (0.48, 1.30) |  | 48.26 | -1.08 (-1.26, -0.90) |
| Northern Mariana Islands | 0.15 (0.09, 0.23) | 0.75 (0.46, 1.12) |  | 0.56 (0.34, 0.87) | 0.98 (0.63, 1.43) |  | 286.94 | 1.30 (1.09, 1.51) |
| Norway | 33.29 (29.22, 37.88) | 0.51 (0.45, 0.58) |  | 89.88 (69.67, 112.63) | 0.99 (0.76, 1.25) |  | 170.00 | 2.80 (2.57, 3.02) |
| Oman | 2.78 (1.55, 4.44) | 0.41 (0.24, 0.65) |  | 9.33 (5.78, 14.75) | 0.54 (0.34, 0.84) |  | 235.06 | 1.76 (1.27, 2.25) |
| Pakistan | 302.56 (189.50, 423.92) | 0.52 (0.32, 0.72) |  | 615.68 (456.76, 833.27) | 0.52 (0.39, 0.70) |  | 103.49 | 0.06 (-0.13, 0.25) |
| Palau | 0.13 (0.07, 0.21) | 1.25 (0.70, 2.06) |  | 0.32 (0.19, 0.50) | 1.36 (0.84, 2.06) |  | 149.90 | 0.32 (0.23, 0.41) |
| Palestine | 6.05 (3.47, 9.37) | 0.73 (0.42, 1.12) |  | 13.24 (8.18, 19.76) | 0.60 (0.37, 0.90) |  | 118.87 | -0.64 (-0.72, -0.56) |
| Panama | 15.23 (11.17, 19.62) | 1.02 (0.75, 1.31) |  | 41.89 (27.81, 60.56) | 1.02 (0.68, 1.47) |  | 175.08 | 0.37 (-0.16, 0.90) |
| Papua New Guinea | 2.98 (1.74, 4.71) | 0.18 (0.11, 0.28) |  | 8.58 (5.07, 14.01) | 0.21 (0.13, 0.34) |  | 187.77 | 0.78 (0.72, 0.84) |
| Paraguay | 17.51 (12.22, 22.69) | 0.80 (0.55, 1.04) |  | 38.02 (24.27, 54.82) | 0.70 (0.44, 1.01) |  | 117.14 | -0.55 (-1.15, 0.05) |
| Peru | 215.84 (151.09, 290.62) | 1.87 (1.31, 2.52) |  | 278.84 (180.02, 394.84) | 0.88 (0.57, 1.26) |  | 29.19 | -3.36 (-4.02, -2.70) |
| Philippines | 651.03 (444.91, 870.76) | 2.09 (1.43, 2.77) |  | 1235.31 (928.34, 1629.70) | 1.52 (1.15, 1.99) |  | 89.75 | -1.49 (-1.78, -1.19) |
| Poland | 1093.51 (966.47, 1222.09) | 2.49 (2.20, 2.78) |  | 608.19 (490.00, 738.96) | 0.86 (0.70, 1.05) |  | -44.38 | -3.30 (-4.63, -1.94) |
| Portugal | 97.82 (74.41, 121.55) | 0.69 (0.53, 0.86) |  | 421.09 (286.05, 590.96) | 1.96 (1.34, 2.78) |  | 330.48 | 3.28 (2.95, 3.62) |
| Puerto Rico | 84.47 (62.42, 107.28) | 2.29 (1.69, 2.92) |  | 65.74 (42.95, 94.15) | 0.95 (0.62, 1.37) |  | -22.17 | -1.95 (-2.60, -1.29) |
| Qatar | 1.51 (0.86, 2.38) | 1.75 (1.01, 2.80) |  | 11.69 (6.37, 19.06) | 1.89 (1.08, 3.06) |  | 674.41 | 0.84 (0.66, 1.03) |
| Romania | 196.83 (146.81, 247.19) | 0.69 (0.52, 0.86) |  | 479.77 (342.69, 628.42) | 1.34 (0.95, 1.76) |  | 143.75 | 3.20 (2.69, 3.71) |
| Russia | 1014.63 (879.70, 1155.44) | 0.55 (0.48, 0.63) |  | 2472.46 (1982.27, 3077.23) | 1.06 (0.85, 1.32) |  | 143.68 | 2.61 (2.35, 2.88) |
| Rwanda | 46.76 (29.37, 71.55) | 1.60 (1.03, 2.38) |  | 83.19 (55.48, 122.77) | 1.37 (0.94, 1.96) |  | 77.89 | -1.38 (-1.66, -1.10) |
| Saint Kitts and Nevis | 1.52 (1.09, 1.99) | 3.92 (2.84, 5.13) |  | 0.85 (0.59, 1.19) | 1.28 (0.90, 1.76) |  | -44.11 | -4.51 (-5.61, -3.40) |
| Saint Lucia | 1.95 (1.48, 2.49) | 2.20 (1.67, 2.79) |  | 1.80 (1.31, 2.37) | 0.84 (0.61, 1.10) |  | -7.44 | -3.59 (-4.62, -2.56) |
| Saint Vincent and the Grenadines | 1.67 (1.21, 2.18) | 2.29 (1.65, 2.96) |  | 1.53 (1.13, 1.98) | 1.13 (0.83, 1.45) |  | -8.07 | -2.61 (-3.60, -1.61) |
| Samoa | 0.71 (0.41, 1.11) | 0.79 (0.47, 1.21) |  | 0.97 (0.58, 1.48) | 0.65 (0.39, 0.99) |  | 37.49 | -0.66 (-0.72, -0.59) |
| San Marino | 0.27 (0.19, 0.36) | 0.80 (0.58, 1.06) |  | 0.74 (0.49, 1.06) | 1.22 (0.80, 1.76) |  | 171.93 | 2.07 (1.86, 2.27) |
| Sao Tome and Principe | 0.27 (0.17, 0.39) | 0.42 (0.27, 0.59) |  | 0.59 (0.34, 0.93) | 0.56 (0.33, 0.87) |  | 120.86 | 0.98 (0.85, 1.11) |
| Saudi Arabia | 22.26 (13.03, 35.68) | 0.41 (0.24, 0.65) |  | 63.50 (36.62, 101.95) | 0.43 (0.25, 0.68) |  | 185.24 | 0.04 (-0.23, 0.30) |
| Senegal | 9.54 (6.07, 14.14) | 0.30 (0.19, 0.44) |  | 20.25 (12.32, 30.22) | 0.28 (0.17, 0.41) |  | 112.28 | 0.04 (-0.16, 0.24) |
| Serbia | 244.66 (173.59, 317.93) | 2.10 (1.52, 2.72) |  | 327.93 (222.02, 455.11) | 2.02 (1.36, 2.79) |  | 34.03 | -0.43 (-0.67, -0.19) |
| Seychelles | 0.90 (0.59, 1.30) | 1.59 (1.05, 2.30) |  | 1.60 (1.03, 2.25) | 1.42 (0.94, 2.00) |  | 77.81 | -0.22 (-0.44, 0.01) |
| Sierra Leone | 20.66 (13.13, 31.97) | 1.08 (0.69, 1.66) |  | 30.21 (18.27, 45.85) | 0.86 (0.53, 1.31) |  | 46.22 | -0.94 (-1.09, -0.80) |
| Singapore | 13.63 (8.85, 19.86) | 0.62 (0.40, 0.91) |  | 70.30 (40.97, 113.30) | 0.91 (0.53, 1.46) |  | 415.97 | 1.18 (0.87, 1.49) |
| Slovakia | 120.63 (93.56, 145.40) | 2.00 (1.57, 2.40) |  | 142.63 (98.76, 193.86) | 1.53 (1.06, 2.09) |  | 18.24 | -1.44 (-1.67, -1.21) |
| Slovenia | 27.81 (19.10, 38.62) | 1.13 (0.77, 1.56) |  | 88.74 (60.13, 125.49) | 2.12 (1.42, 3.01) |  | 219.12 | 2.51 (2.23, 2.78) |
| Solomon Islands | 1.02 (0.59, 1.65) | 0.72 (0.43, 1.17) |  | 1.92 (1.15, 2.92) | 0.62 (0.38, 0.94) |  | 88.34 | -0.45 (-0.55, -0.36) |
| Somalia | 18.89 (9.67, 40.94) | 0.76 (0.41, 1.63) |  | 44.47 (23.84, 85.82) | 0.68 (0.37, 1.31) |  | 135.38 | -0.33 (-0.42, -0.23) |
| South Africa | 256.92 (150.40, 486.87) | 1.19 (0.69, 2.25) |  | 541.47 (442.18, 659.61) | 1.18 (0.96, 1.42) |  | 110.75 | -0.55 (-1.22, 0.13) |
| South Korea | 447.21 (285.03, 662.43) | 1.44 (0.93, 2.12) |  | 3603.17 (2376.50, 5240.04) | 3.98 (2.60, 5.79) |  | 705.70 | 4.23 (2.96, 5.52) |
| South Sudan | 16.76 (8.19, 37.55) | 0.70 (0.34, 1.57) |  | 22.75 (10.66, 47.02) | 0.62 (0.31, 1.29) |  | 35.73 | -0.41 (-0.46, -0.36) |
| Spain | 667.19 (470.15, 865.28) | 1.21 (0.86, 1.56) |  | 1509.02 (969.06, 2160.75) | 1.73 (1.12, 2.49) |  | 126.17 | 1.00 (0.60, 1.41) |
| Sri Lanka | 43.50 (28.67, 61.52) | 0.42 (0.28, 0.59) |  | 180.48 (110.21, 275.89) | 0.69 (0.43, 1.05) |  | 314.92 | 2.76 (2.40, 3.11) |
| Sudan | 42.02 (21.16, 80.95) | 0.46 (0.23, 0.87) |  | 90.77 (47.43, 171.02) | 0.53 (0.28, 1.00) |  | 115.98 | 0.50 (0.35, 0.66) |
| Suriname | 5.49 (3.94, 7.18) | 2.14 (1.54, 2.80) |  | 5.43 (3.84, 7.61) | 0.91 (0.64, 1.27) |  | -1.11 | -2.80 (-3.55, -2.05) |
| Sweden | 139.64 (121.15, 158.47) | 0.95 (0.83, 1.08) |  | 262.97 (208.83, 327.41) | 1.35 (1.07, 1.69) |  | 88.33 | 1.74 (1.13, 2.36) |
| Switzerland | 117.46 (89.56, 142.30) | 1.16 (0.89, 1.40) |  | 396.55 (266.04, 560.40) | 2.40 (1.61, 3.41) |  | 237.60 | 1.76 (1.18, 2.33) |
| Syrian Arab Republic | 22.66 (13.56, 34.58) | 0.45 (0.27, 0.69) |  | 48.82 (27.91, 78.39) | 0.41 (0.23, 0.64) |  | 115.47 | -0.45 (-0.56, -0.33) |
| Taiwan (Province of China) | 207.19 (135.38, 301.84) | 1.21 (0.80, 1.76) |  | 307.74 (183.29, 481.96) | 0.77 (0.46, 1.21) |  | 48.53 | -2.76 (-3.97, -1.53) |
| Tajikistan | 5.69 (3.82, 7.78) | 0.21 (0.14, 0.29) |  | 37.90 (24.63, 55.01) | 0.82 (0.53, 1.20) |  | 565.67 | 5.60 (5.38, 5.83) |
| Tanzania | 56.96 (37.41, 83.08) | 0.53 (0.35, 0.77) |  | 147.02 (95.77, 214.05) | 0.62 (0.41, 0.91) |  | 158.12 | 0.50 (0.35, 0.64) |
| Thailand | 1892.32 (1294.88, 2620.13) | 5.20 (3.54, 7.14) |  | 7426.36 (4787.57, 11075.26) | 7.16 (4.64, 10.56) |  | 292.45 | 1.32 (1.17, 1.47) |
| Timor-Leste | 3.78 (2.07, 6.20) | 1.33 (0.75, 2.12) |  | 10.26 (5.35, 17.00) | 1.24 (0.67, 2.03) |  | 171.61 | -0.58 (-0.78, -0.39) |
| Togo | 13.49 (8.29, 20.94) | 1.10 (0.68, 1.66) |  | 32.05 (19.67, 48.79) | 0.88 (0.56, 1.32) |  | 137.68 | -1.35 (-1.54, -1.16) |
| Tokelau | 0.01 (0.01, 0.02) | 0.91 (0.53, 1.42) |  | 0.01 (0.01, 0.02) | 0.86 (0.49, 1.31) |  | -7.30 | -0.28 (-0.33, -0.24) |
| Tonga | 1.62 (0.89, 2.56) | 2.82 (1.58, 4.40) |  | 2.56 (1.50, 3.97) | 3.23 (1.89, 5.01) |  | 58.17 | 0.46 (0.25, 0.68) |
| Trinidad and Tobago | 18.12 (13.33, 23.26) | 2.14 (1.57, 2.74) |  | 16.17 (10.49, 23.30) | 0.86 (0.56, 1.23) |  | -10.75 | -3.25 (-4.31, -2.18) |
| Tunisia | 10.00 (5.42, 16.82) | 0.20 (0.11, 0.33) |  | 27.34 (14.11, 49.86) | 0.22 (0.11, 0.39) |  | 173.34 | 0.38 (0.27, 0.50) |
| Turkey | 156.65 (100.67, 233.82) | 0.46 (0.29, 0.68) |  | 351.16 (219.19, 532.02) | 0.40 (0.25, 0.61) |  | 124.17 | -0.33 (-0.58, -0.09) |
| Turkmenistan | 4.61 (3.19, 6.21) | 0.25 (0.17, 0.34) |  | 69.45 (45.44, 98.80) | 1.68 (1.11, 2.38) |  | 1406.36 | 7.68 (6.50, 8.88) |
| Tuvalu | 0.08 (0.05, 0.13) | 1.15 (0.67, 1.79) |  | 0.11 (0.06, 0.17) | 1.02 (0.60, 1.61) |  | 28.66 | -0.49 (-0.64, -0.34) |
| Uganda | 98.91 (65.04, 136.34) | 1.49 (1.00, 2.03) |  | 286.84 (194.71, 409.92) | 1.97 (1.34, 2.74) |  | 190.01 | 1.10 (0.88, 1.31) |
| Ukraine | 202.46 (174.13, 229.88) | 0.28 (0.24, 0.31) |  | 687.68 (552.56, 840.39) | 0.91 (0.73, 1.12) |  | 239.66 | 5.18 (4.49, 5.88) |
| United Arab Emirates | 2.19 (0.90, 4.67) | 0.54 (0.21, 1.22) |  | 22.97 (7.35, 61.46) | 0.58 (0.19, 1.57) |  | 950.95 | 0.14 (-0.04, 0.33) |
| United Kingdom | 653.87 (580.45, 727.05) | 0.73 (0.65, 0.81) |  | 2295.95 (1801.53, 2880.57) | 1.88 (1.47, 2.37) |  | 251.13 | 4.06 (3.81, 4.31) |
| United States of America | 2022.09 (1789.12, 2247.23) | 0.65 (0.57, 0.72) |  | 8927.66 (6832.86, 11476.75) | 1.66 (1.27, 2.15) |  | 341.51 | 3.12 (2.97, 3.26) |
| United States Virgin Islands | 1.08 (0.76, 1.46) | 1.26 (0.89, 1.73) |  | 1.67 (1.17, 2.31) | 0.86 (0.61, 1.18) |  | 54.72 | -1.29 (-1.95, -0.62) |
| Uruguay | 15.50 (10.73, 20.99) | 0.39 (0.27, 0.53) |  | 36.42 (23.72, 52.29) | 0.70 (0.45, 1.00) |  | 135.01 | 2.41 (2.23, 2.58) |
| Uzbekistan | 22.53 (15.71, 29.75) | 0.21 (0.14, 0.27) |  | 377.03 (252.08, 523.59) | 1.78 (1.21, 2.42) |  | 1573.35 | 9.85 (8.55, 11.16) |
| Vanuatu | 0.73 (0.37, 1.29) | 1.11 (0.56, 1.94) |  | 2.05 (1.08, 3.37) | 1.16 (0.63, 1.93) |  | 179.13 | 0.31 (0.21, 0.42) |
| Venezuela | 205.79 (151.66, 258.33) | 2.16 (1.57, 2.72) |  | 233.12 (153.77, 333.74) | 0.81 (0.54, 1.15) |  | 13.28 | -2.66 (-3.78, -1.52) |
| Viet Nam | 270.82 (164.59, 419.23) | 0.67 (0.41, 1.04) |  | 558.21 (338.26, 825.34) | 0.63 (0.38, 0.92) |  | 106.12 | -0.45 (-0.64, -0.26) |
| Yemen | 9.40 (4.91, 17.14) | 0.20 (0.10, 0.35) |  | 27.72 (15.80, 46.83) | 0.22 (0.12, 0.37) |  | 194.75 | 0.49 (0.39, 0.59) |
| Zambia | 16.75 (9.98, 29.45) | 0.62 (0.37, 1.09) |  | 47.96 (31.03, 67.20) | 0.76 (0.51, 1.07) |  | 186.23 | -0.08 (-0.50, 0.34) |
| Zimbabwe | 84.24 (47.41, 162.38) | 1.98 (1.14, 3.80) |  | 152.68 (90.79, 252.20) | 2.07 (1.25, 3.38) |  | 81.24 | -1.31 (-1.91, -0.70) |

ASIR: age-standardized incidence rate; CI: confidence interval; EAPC: estimated annual percentage change; UI: uncertainty interval.


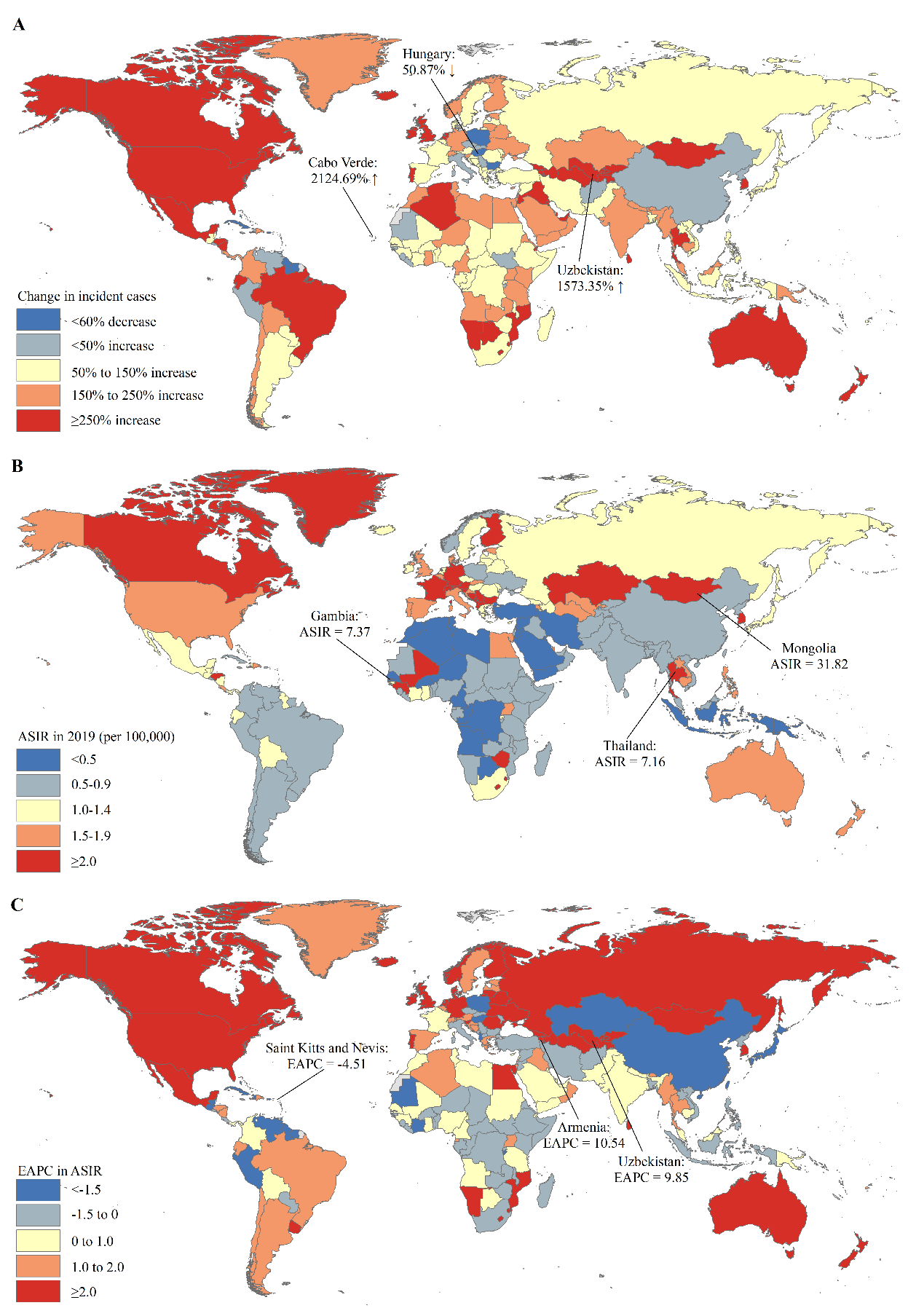


**Figure S3.** The global trends in the incidence of primary liver cancer due to alcohol use in 204 countries and territories. (A) The percentage change in incident cases of primary liver cancer due to alcohol use between 1990 and 2019; (B) The ASIR of primary liver cancer due to alcohol use in 2019; (C) The EAPCs in ASIR of primary liver cancer due to alcohol use from 1990 to 2019.

Note: ASIR: age-standardized incidence rate; EAPC: estimated annual percentage change.

**Table S16**. **The incident cases and ASIR of primary liver cancer due to NASH in 1990 and 2019 and their change trends from 1990 to 2019 at national level.**

| Nation | 1990 | |  | 2019 | |  | 1990-2019 | |
| --- | --- | --- | --- | --- | --- | --- | --- | --- |
|  | Incident cases  No. (95% UI) | ASIR per 100,000  No. (95% UI) |  | Incident cases  No. x 10^3^ (95% UI) | ASIR per 100,000  No. (95% UI) |  | Percentage change in incident cases  No. (%) | EAPC in ASIR  No. (95% CI) |
| Afghanistan | 61.87 (40.31, 93.34) | 0.86 (0.57, 1.28) |  | 115.91 (74.29, 168.02) | 0.86 (0.54, 1.26) |  | 87.35 | 0.03 (0.01, 0.06) |
| Albania | 15.63 (11.03, 21.94) | 0.79 (0.55, 1.12) |  | 22.52 (13.90, 34.86) | 0.52 (0.33, 0.80) |  | 44.07 | -2.55 (-3.11, -1.98) |
| Algeria | 16.78 (10.94, 25.08) | 0.15 (0.10, 0.22) |  | 79.27 (51.09, 118.54) | 0.25 (0.16, 0.37) |  | 372.32 | 1.96 (1.89, 2.03) |
| American Samoa | 0.13 (0.08, 0.18) | 0.57 (0.37, 0.82) |  | 0.38 (0.26, 0.54) | 0.80 (0.55, 1.13) |  | 198.38 | 1.32 (0.94, 1.70) |
| Andorra | 0.28 (0.17, 0.45) | 0.53 (0.32, 0.83) |  | 1.00 (0.61, 1.57) | 0.72 (0.45, 1.14) |  | 256.22 | 1.15 (1.10, 1.20) |
| Angola | 5.70 (3.61, 8.68) | 0.15 (0.09, 0.22) |  | 18.75 (12.43, 27.98) | 0.17 (0.11, 0.25) |  | 229.18 | 0.25 (0.17, 0.34) |
| Antigua and Barbuda | 0.32 (0.22, 0.45) | 0.58 (0.41, 0.82) |  | 0.25 (0.17, 0.36) | 0.26 (0.18, 0.37) |  | -22.40 | -3.21 (-4.21, -2.19) |
| Argentina | 32.54 (21.81, 48.83) | 0.10 (0.07, 0.15) |  | 100.27 (64.41, 148.49) | 0.18 (0.12, 0.27) |  | 208.19 | 2.78 (2.55, 3.02) |
| Armenia | 1.50 (1.03, 2.15) | 0.06 (0.04, 0.09) |  | 21.06 (14.18, 30.25) | 0.51 (0.35, 0.74) |  | 1301.87 | 10.92 (9.28, 12.58) |
| Australia | 28.39 (19.87, 40.24) | 0.14 (0.10, 0.20) |  | 191.41 (122.41, 288.67) | 0.46 (0.30, 0.70) |  | 574.30 | 4.39 (4.02, 4.76) |
| Austria | 16.98 (11.59, 24.91) | 0.14 (0.10, 0.20) |  | 46.68 (29.04, 72.16) | 0.26 (0.17, 0.40) |  | 174.86 | 2.50 (2.10, 2.89) |
| Azerbaijan | 2.72 (1.86, 3.91) | 0.06 (0.04, 0.08) |  | 26.75 (17.15, 41.07) | 0.33 (0.21, 0.53) |  | 884.02 | 7.24 (5.28, 9.25) |
| Bahamas | 0.95 (0.68, 1.32) | 0.61 (0.43, 0.86) |  | 1.14 (0.77, 1.65) | 0.30 (0.21, 0.44) |  | 20.19 | -2.75 (-3.58, -1.92) |
| Bahrain | 0.88 (0.60, 1.27) | 0.61 (0.41, 0.91) |  | 5.73 (3.70, 8.66) | 0.83 (0.55, 1.24) |  | 550.78 | 0.37 (0.02, 0.72) |
| Bangladesh | 117.51 (81.47, 164.73) | 0.21 (0.14, 0.29) |  | 266.67 (178.78, 390.09) | 0.20 (0.13, 0.30) |  | 126.93 | -0.49 (-0.62, -0.36) |
| Barbados | 0.74 (0.47, 1.13) | 0.24 (0.16, 0.36) |  | 1.33 (0.90, 1.93) | 0.27 (0.19, 0.39) |  | 78.73 | 0.27 (0.09, 0.45) |
| Belarus | 10.72 (7.48, 15.16) | 0.08 (0.06, 0.12) |  | 27.62 (17.26, 42.36) | 0.17 (0.11, 0.27) |  | 157.77 | 3.19 (2.71, 3.68) |
| Belgium | 26.20 (17.43, 37.74) | 0.17 (0.11, 0.24) |  | 59.72 (37.27, 91.66) | 0.26 (0.16, 0.40) |  | 127.97 | 1.35 (1.01, 1.70) |
| Belize | 0.47 (0.33, 0.66) | 0.51 (0.35, 0.71) |  | 0.80 (0.56, 1.10) | 0.30 (0.21, 0.42) |  | 68.67 | -2.06 (-2.54, -1.58) |
| Benin | 9.66 (6.43, 14.08) | 0.49 (0.32, 0.71) |  | 22.92 (15.29, 35.15) | 0.48 (0.32, 0.73) |  | 137.29 | -0.26 (-0.36, -0.17) |
| Bermuda | 0.40 (0.27, 0.56) | 0.65 (0.45, 0.91) |  | 0.29 (0.19, 0.42) | 0.22 (0.14, 0.32) |  | -27.89 | -3.81 (-4.84, -2.78) |
| Bhutan | 0.41 (0.24, 0.67) | 0.16 (0.10, 0.26) |  | 1.59 (0.96, 2.49) | 0.29 (0.17, 0.46) |  | 286.61 | 2.23 (2.10, 2.36) |
| Bolivia | 12.14 (7.33, 20.68) | 0.41 (0.25, 0.68) |  | 37.98 (23.49, 60.27) | 0.46 (0.29, 0.73) |  | 212.96 | 0.38 (0.28, 0.47) |
| Bosnia and Herzegovina | 14.73 (10.01, 21.15) | 0.38 (0.26, 0.55) |  | 42.97 (28.27, 63.95) | 0.72 (0.48, 1.06) |  | 191.74 | 2.96 (2.49, 3.43) |
| Botswana | 0.47 (0.20, 1.03) | 0.08 (0.04, 0.18) |  | 2.05 (1.25, 3.12) | 0.15 (0.09, 0.23) |  | 336.09 | 1.27 (0.64, 1.90) |
| Brazil | 97.76 (83.28, 113.84) | 0.11 (0.09, 0.13) |  | 351.69 (299.14, 412.42) | 0.15 (0.13, 0.18) |  | 259.77 | 1.58 (1.36, 1.79) |
| Brunei | 0.43 (0.28, 0.65) | 0.52 (0.33, 0.78) |  | 1.90 (1.23, 2.87) | 0.69 (0.46, 1.01) |  | 342.43 | 1.66 (1.16, 2.17) |
| Bulgaria | 54.07 (36.89, 76.61) | 0.43 (0.30, 0.60) |  | 46.48 (30.58, 68.35) | 0.33 (0.22, 0.48) |  | -14.03 | -0.47 (-0.95, 0.01) |
| Burkina Faso | 8.38 (5.60, 12.30) | 0.20 (0.14, 0.29) |  | 17.50 (11.24, 25.32) | 0.19 (0.13, 0.29) |  | 108.79 | -0.29 (-0.48, -0.11) |
| Burundi | 9.05 (5.65, 13.82) | 0.39 (0.24, 0.59) |  | 14.85 (9.20, 24.45) | 0.34 (0.21, 0.55) |  | 64.06 | -0.96 (-1.14, -0.79) |
| Cambodia | 31.01 (21.21, 45.05) | 0.62 (0.41, 0.88) |  | 74.48 (48.75, 109.05) | 0.62 (0.41, 0.91) |  | 140.19 | -0.22 (-0.33, -0.11) |
| Cameroon | 2.43 (1.46, 3.88) | 0.06 (0.03, 0.09) |  | 7.62 (4.76, 11.83) | 0.07 (0.04, 0.10) |  | 213.22 | 0.25 (0.07, 0.44) |
| Canada | 67.13 (48.47, 93.44) | 0.21 (0.15, 0.28) |  | 393.70 (258.31, 575.57) | 0.58 (0.39, 0.83) |  | 486.43 | 3.98 (3.76, 4.20) |
| Cape Verde | 0.23 (0.15, 0.33) | 0.10 (0.06, 0.14) |  | 5.23 (3.67, 7.52) | 1.27 (0.87, 1.87) |  | 2190.85 | 7.36 (5.19, 9.57) |
| Central African Republic | 2.59 (1.56, 4.11) | 0.22 (0.13, 0.34) |  | 4.72 (2.68, 7.93) | 0.22 (0.13, 0.36) |  | 82.35 | -0.41 (-0.63, -0.19) |
| Chad | 12.66 (8.18, 18.67) | 0.45 (0.29, 0.66) |  | 23.92 (15.18, 36.12) | 0.44 (0.28, 0.66) |  | 89.04 | -0.24 (-0.30, -0.18) |
| Chile | 16.54 (11.51, 23.25) | 0.17 (0.12, 0.24) |  | 73.77 (47.59, 110.92) | 0.31 (0.20, 0.46) |  | 346.00 | 2.71 (2.44, 2.98) |
| China | 8809.01 (6898.09, 10829.66) | 1.01 (0.80, 1.24) |  | 10034.48 (7812.80, 12509.44) | 0.51 (0.40, 0.63) |  | 13.91 | -3.90 (-4.76, -3.04) |
| Colombia | 30.07 (21.32, 42.52) | 0.17 (0.12, 0.25) |  | 101.98 (65.22, 155.30) | 0.19 (0.12, 0.30) |  | 239.13 | 0.60 (0.12, 1.10) |
| Comoros | 0.87 (0.45, 1.50) | 0.40 (0.22, 0.68) |  | 1.98 (1.21, 3.12) | 0.42 (0.25, 0.66) |  | 126.86 | -0.10 (-0.26, 0.05) |
| Congo | 2.82 (1.73, 4.31) | 0.26 (0.17, 0.40) |  | 5.92 (3.54, 9.25) | 0.23 (0.14, 0.36) |  | 110.30 | -0.83 (-0.97, -0.70) |
| Cook Islands | 0.15 (0.10, 0.21) | 1.20 (0.83, 1.70) |  | 0.32 (0.22, 0.46) | 1.32 (0.87, 1.85) |  | 115.52 | 0.23 (0.12, 0.34) |
| Costa Rica | 6.68 (4.66, 9.26) | 0.38 (0.26, 0.53) |  | 23.27 (15.13, 35.08) | 0.46 (0.30, 0.69) |  | 248.33 | 0.42 (-0.14, 0.98) |
| Cote d'Ivoire | 20.19 (13.19, 29.58) | 0.53 (0.34, 0.79) |  | 43.84 (28.15, 66.65) | 0.44 (0.29, 0.69) |  | 117.10 | -1.31 (-1.55, -1.08) |
| Croatia | 16.71 (11.52, 23.26) | 0.27 (0.18, 0.37) |  | 30.99 (19.92, 46.05) | 0.34 (0.22, 0.51) |  | 85.49 | 1.50 (1.09, 1.91) |
| Cuba | 56.87 (40.18, 78.46) | 0.54 (0.39, 0.75) |  | 45.11 (29.34, 67.87) | 0.23 (0.15, 0.36) |  | -20.67 | -3.29 (-4.51, -2.05) |
| Cyprus | 1.35 (0.86, 1.99) | 0.17 (0.12, 0.25) |  | 4.42 (2.87, 6.65) | 0.23 (0.15, 0.34) |  | 226.50 | 1.36 (1.10, 1.63) |
| Czech Republic | 37.55 (25.98, 54.25) | 0.27 (0.19, 0.38) |  | 45.52 (29.41, 67.97) | 0.21 (0.14, 0.32) |  | 21.24 | -0.99 (-1.19, -0.80) |
| Democratic Republic of the Congo | 24.25 (15.87, 35.51) | 0.15 (0.10, 0.22) |  | 56.19 (36.61, 86.07) | 0.15 (0.10, 0.23) |  | 131.72 | -0.22 (-0.29, -0.16) |
| Denmark | 10.74 (7.17, 15.79) | 0.13 (0.09, 0.19) |  | 26.79 (16.73, 41.07) | 0.24 (0.15, 0.36) |  | 149.43 | 2.47 (2.23, 2.71) |
| Djibouti | 0.50 (0.27, 0.85) | 0.37 (0.20, 0.63) |  | 2.37 (1.30, 4.17) | 0.43 (0.24, 0.74) |  | 376.38 | 0.44 (0.32, 0.56) |
| Dominica | 0.53 (0.37, 0.75) | 0.71 (0.49, 0.99) |  | 0.28 (0.19, 0.40) | 0.31 (0.21, 0.44) |  | -48.09 | -3.05 (-3.81, -2.29) |
| Dominican Republic | 10.65 (7.54, 15.10) | 0.28 (0.20, 0.40) |  | 37.69 (22.88, 58.64) | 0.41 (0.25, 0.64) |  | 253.90 | 1.69 (1.17, 2.21) |
| Ecuador | 14.75 (10.34, 20.61) | 0.29 (0.20, 0.41) |  | 60.70 (39.77, 88.65) | 0.43 (0.28, 0.61) |  | 311.50 | 1.73 (1.41, 2.05) |
| Egypt | 323.90 (212.25, 484.56) | 1.08 (0.70, 1.65) |  | 1270.84 (763.93, 2053.17) | 1.94 (1.18, 3.07) |  | 292.35 | 2.80 (2.44, 3.17) |
| El Salvador | 7.46 (5.22, 10.53) | 0.25 (0.17, 0.36) |  | 10.72 (6.67, 16.60) | 0.18 (0.11, 0.28) |  | 43.76 | -1.18 (-1.88, -0.47) |
| Equatorial Guinea | 0.37 (0.22, 0.59) | 0.19 (0.11, 0.29) |  | 1.38 (0.77, 2.23) | 0.28 (0.15, 0.47) |  | 272.00 | 1.57 (1.45, 1.69) |
| Eritrea | 3.47 (2.02, 5.77) | 0.37 (0.21, 0.63) |  | 10.07 (6.23, 16.30) | 0.41 (0.25, 0.65) |  | 189.94 | 0.03 (-0.15, 0.22) |
| Estonia | 2.99 (2.05, 4.31) | 0.15 (0.10, 0.21) |  | 7.92 (5.01, 12.06) | 0.29 (0.19, 0.44) |  | 165.09 | 1.45 (0.97, 1.93) |
| Eswatini | 1.60 (0.92, 3.03) | 0.54 (0.31, 1.01) |  | 10.51 (3.24, 19.79) | 1.75 (0.57, 3.25) |  | 558.80 | 4.78 (3.50, 6.08) |
| Ethiopia | 54.02 (35.82, 79.77) | 0.27 (0.19, 0.39) |  | 111.87 (86.70, 146.75) | 0.28 (0.21, 0.37) |  | 107.08 | -0.15 (-0.24, -0.05) |
| Federated States of Micronesia | 0.41 (0.26, 0.64) | 0.89 (0.56, 1.39) |  | 0.68 (0.38, 1.11) | 1.01 (0.60, 1.56) |  | 67.75 | 0.22 (0.12, 0.32) |
| Fiji | 1.71 (1.12, 2.52) | 0.48 (0.31, 0.71) |  | 4.54 (2.88, 6.80) | 0.63 (0.41, 0.91) |  | 165.57 | 1.29 (1.05, 1.52) |
| Finland | 14.44 (9.68, 21.06) | 0.20 (0.13, 0.29) |  | 46.08 (28.81, 71.37) | 0.37 (0.23, 0.56) |  | 219.05 | 2.39 (2.19, 2.60) |
| France | 195.01 (133.32, 291.76) | 0.24 (0.16, 0.35) |  | 531.23 (324.98, 837.12) | 0.40 (0.25, 0.61) |  | 172.41 | 1.58 (1.30, 1.85) |
| Gabon | 1.15 (0.73, 1.75) | 0.21 (0.13, 0.31) |  | 2.74 (1.64, 4.20) | 0.27 (0.16, 0.42) |  | 137.05 | 0.62 (0.44, 0.80) |
| Gambia | 7.33 (4.67, 10.94) | 2.05 (1.33, 3.01) |  | 31.50 (19.88, 47.48) | 3.20 (2.05, 4.80) |  | 329.82 | 1.21 (0.99, 1.44) |
| Georgia | 4.65 (3.14, 6.67) | 0.08 (0.05, 0.11) |  | 14.16 (9.75, 20.37) | 0.24 (0.17, 0.34) |  | 204.65 | 3.04 (1.82, 4.27) |
| Germany | 227.08 (158.74, 316.11) | 0.18 (0.12, 0.24) |  | 772.72 (481.88, 1170.01) | 0.40 (0.26, 0.60) |  | 240.29 | 3.13 (2.60, 3.67) |
| Ghana | 23.65 (15.24, 35.74) | 0.39 (0.25, 0.60) |  | 73.64 (49.25, 109.56) | 0.47 (0.31, 0.70) |  | 211.36 | 0.33 (0.08, 0.58) |
| Greece | 23.99 (15.97, 34.93) | 0.15 (0.10, 0.22) |  | 63.38 (40.08, 96.14) | 0.24 (0.16, 0.37) |  | 164.20 | 1.79 (1.67, 1.91) |
| Greenland | 0.14 (0.09, 0.20) | 0.40 (0.28, 0.58) |  | 0.42 (0.28, 0.63) | 0.63 (0.41, 0.91) |  | 207.95 | 1.31 (1.15, 1.47) |
| Grenada | 0.49 (0.34, 0.69) | 0.65 (0.45, 0.92) |  | 0.28 (0.19, 0.40) | 0.26 (0.18, 0.38) |  | -42.60 | -3.39 (-4.36, -2.41) |
| Guam | 0.25 (0.17, 0.36) | 0.35 (0.24, 0.50) |  | 1.07 (0.71, 1.56) | 0.56 (0.38, 0.83) |  | 333.11 | 2.10 (1.96, 2.23) |
| Guatemala | 23.17 (16.31, 32.47) | 0.65 (0.45, 0.91) |  | 43.65 (28.43, 64.10) | 0.39 (0.25, 0.59) |  | 88.40 | -2.43 (-3.89, -0.95) |
| Guinea | 67.32 (44.48, 97.44) | 2.08 (1.38, 3.01) |  | 136.81 (86.91, 207.97) | 2.52 (1.59, 3.89) |  | 103.22 | 0.69 (0.65, 0.74) |
| Guinea-Bissau | 2.34 (1.45, 3.77) | 0.58 (0.36, 0.91) |  | 3.99 (2.43, 6.22) | 0.57 (0.35, 0.87) |  | 70.03 | -0.27 (-0.33, -0.22) |
| Guyana | 1.82 (1.26, 2.59) | 0.48 (0.33, 0.69) |  | 1.67 (1.09, 2.51) | 0.28 (0.18, 0.41) |  | -8.25 | -2.14 (-2.96, -1.32) |
| Haiti | 13.63 (7.45, 22.11) | 0.45 (0.25, 0.74) |  | 19.08 (9.68, 31.54) | 0.29 (0.15, 0.48) |  | 39.98 | -1.47 (-1.72, -1.23) |
| Honduras | 14.99 (5.04, 26.09) | 0.73 (0.22, 1.30) |  | 72.70 (28.28, 126.54) | 1.24 (0.47, 2.19) |  | 385.04 | 2.14 (2.02, 2.26) |
| Hungary | 66.11 (46.17, 93.13) | 0.45 (0.32, 0.63) |  | 40.64 (27.53, 58.26) | 0.21 (0.14, 0.30) |  | -38.52 | -2.04 (-2.74, -1.33) |
| Iceland | 0.38 (0.26, 0.54) | 0.13 (0.09, 0.19) |  | 1.32 (0.89, 1.93) | 0.24 (0.16, 0.35) |  | 250.26 | 1.99 (1.83, 2.16) |
| India | 1111.33 (879.20, 1366.68) | 0.27 (0.21, 0.33) |  | 3338.70 (2655.95, 4188.43) | 0.30 (0.24, 0.38) |  | 200.42 | 0.39 (0.31, 0.48) |
| Indonesia | 213.81 (171.72, 260.79) | 0.21 (0.17, 0.26) |  | 444.47 (349.82, 539.32) | 0.22 (0.18, 0.27) |  | 107.87 | 0.03 (-0.02, 0.09) |
| Iran | 116.10 (87.95, 150.04) | 0.52 (0.39, 0.69) |  | 388.76 (325.86, 460.99) | 0.57 (0.48, 0.68) |  | 234.86 | -0.17 (-0.89, 0.55) |
| Iraq | 31.94 (21.05, 46.92) | 0.42 (0.27, 0.61) |  | 152.05 (96.76, 227.40) | 0.69 (0.45, 1.00) |  | 376.08 | 2.03 (1.52, 2.54) |
| Ireland | 3.79 (2.55, 5.55) | 0.09 (0.06, 0.13) |  | 21.39 (13.37, 32.79) | 0.28 (0.18, 0.43) |  | 464.34 | 4.82 (4.45, 5.19) |
| Israel | 9.15 (6.02, 13.51) | 0.18 (0.12, 0.27) |  | 27.26 (17.08, 41.50) | 0.24 (0.15, 0.36) |  | 197.96 | 0.70 (0.57, 0.83) |
| Italy | 269.62 (226.44, 319.02) | 0.30 (0.25, 0.35) |  | 373.16 (286.85, 475.34) | 0.27 (0.21, 0.34) |  | 38.40 | -0.84 (-1.09, -0.60) |
| Jamaica | 5.51 (3.85, 7.85) | 0.30 (0.21, 0.43) |  | 7.55 (4.99, 11.18) | 0.25 (0.17, 0.38) |  | 36.98 | 0.44 (-0.50, 1.40) |
| Japan | 973.37 (823.91, 1147.82) | 0.57 (0.48, 0.67) |  | 2195.29 (1661.27, 2802.15) | 0.56 (0.44, 0.71) |  | 125.53 | -0.78 (-1.34, -0.22) |
| Jordan | 3.77 (2.47, 5.63) | 0.31 (0.20, 0.48) |  | 19.34 (12.76, 28.39) | 0.33 (0.22, 0.49) |  | 412.48 | 0.10 (-0.05, 0.25) |
| Kazakhstan | 25.27 (17.52, 36.29) | 0.20 (0.14, 0.29) |  | 82.04 (55.16, 119.49) | 0.49 (0.33, 0.72) |  | 224.65 | 0.02 (-1.01, 1.05) |
| Kenya | 28.46 (19.58, 48.46) | 0.35 (0.24, 0.59) |  | 95.86 (62.72, 142.01) | 0.45 (0.30, 0.65) |  | 236.78 | 0.31 (-0.01, 0.63) |
| Kiribati | 0.41 (0.27, 0.62) | 1.07 (0.69, 1.61) |  | 0.78 (0.52, 1.16) | 1.11 (0.73, 1.65) |  | 89.23 | 0.02 (-0.14, 0.19) |
| Kuwait | 1.76 (1.23, 2.44) | 0.31 (0.21, 0.44) |  | 9.16 (6.13, 13.59) | 0.42 (0.27, 0.63) |  | 420.02 | 1.96 (1.64, 2.27) |
| Kyrgyzstan | 2.09 (1.46, 3.04) | 0.07 (0.05, 0.10) |  | 6.96 (4.78, 10.07) | 0.16 (0.11, 0.24) |  | 233.39 | 3.60 (3.37, 3.84) |
| Lao | 14.28 (8.27, 23.31) | 0.69 (0.40, 1.09) |  | 22.62 (14.17, 33.55) | 0.53 (0.34, 0.79) |  | 58.42 | -1.13 (-1.27, -0.99) |
| Latvia | 4.20 (2.89, 5.95) | 0.12 (0.08, 0.16) |  | 8.45 (5.50, 12.56) | 0.21 (0.14, 0.31) |  | 101.50 | 1.20 (0.50, 1.91) |
| Lebanon | 5.56 (3.63, 8.17) | 0.26 (0.17, 0.38) |  | 19.81 (12.54, 31.62) | 0.38 (0.24, 0.60) |  | 255.92 | 1.42 (1.20, 1.64) |
| Lesotho | 4.08 (2.18, 8.08) | 0.42 (0.23, 0.82) |  | 16.39 (6.77, 27.88) | 1.30 (0.56, 2.20) |  | 301.72 | 4.38 (3.79, 4.98) |
| Liberia | 5.61 (3.69, 8.31) | 0.52 (0.34, 0.77) |  | 9.71 (6.19, 14.95) | 0.50 (0.31, 0.77) |  | 72.97 | -0.21 (-0.37, -0.05) |
| Libya | 10.29 (6.54, 15.49) | 0.57 (0.36, 0.87) |  | 34.50 (22.64, 54.43) | 0.70 (0.45, 1.09) |  | 235.23 | 0.97 (0.76, 1.17) |
| Lithuania | 5.13 (3.58, 7.29) | 0.11 (0.08, 0.16) |  | 12.69 (8.23, 18.56) | 0.22 (0.14, 0.32) |  | 147.28 | 1.45 (1.14, 1.76) |
| Luxembourg | 0.91 (0.61, 1.33) | 0.16 (0.11, 0.24) |  | 2.53 (1.59, 3.84) | 0.25 (0.16, 0.38) |  | 178.06 | 1.55 (1.22, 1.87) |
| Macedonia | 11.15 (7.69, 15.69) | 0.62 (0.43, 0.87) |  | 22.96 (15.02, 34.26) | 0.73 (0.48, 1.07) |  | 105.94 | 0.58 (0.47, 0.69) |
| Madagascar | 16.69 (10.40, 26.96) | 0.32 (0.20, 0.54) |  | 32.85 (19.74, 52.10) | 0.31 (0.19, 0.49) |  | 96.82 | -0.36 (-0.45, -0.26) |
| Malawi | 14.01 (9.07, 21.44) | 0.37 (0.24, 0.56) |  | 24.86 (16.33, 36.09) | 0.36 (0.24, 0.52) |  | 77.46 | -0.84 (-1.14, -0.53) |
| Malaysia | 41.48 (28.63, 59.20) | 0.47 (0.32, 0.68) |  | 175.47 (113.41, 262.31) | 0.69 (0.45, 1.01) |  | 323.04 | 1.36 (1.05, 1.67) |
| Maldives | 0.38 (0.21, 0.68) | 0.48 (0.28, 0.83) |  | 1.48 (0.98, 2.21) | 0.53 (0.35, 0.79) |  | 288.36 | 0.54 (0.35, 0.74) |
| Mali | 37.10 (25.04, 54.06) | 0.86 (0.58, 1.26) |  | 89.90 (57.15, 138.41) | 1.00 (0.64, 1.51) |  | 142.34 | 0.36 (0.28, 0.44) |
| Malta | 0.42 (0.28, 0.62) | 0.10 (0.07, 0.14) |  | 1.60 (1.03, 2.38) | 0.17 (0.12, 0.25) |  | 279.31 | 1.88 (1.53, 2.24) |
| Marshall Islands | 0.13 (0.09, 0.20) | 0.82 (0.52, 1.28) |  | 0.29 (0.18, 0.45) | 0.86 (0.55, 1.31) |  | 115.54 | 0.10 (-0.02, 0.21) |
| Mauritania | 6.16 (4.14, 8.99) | 0.62 (0.42, 0.91) |  | 9.92 (6.32, 14.58) | 0.50 (0.32, 0.73) |  | 61.01 | -0.82 (-0.90, -0.74) |
| Mauritius | 1.17 (0.82, 1.66) | 0.16 (0.11, 0.23) |  | 3.78 (2.45, 5.73) | 0.23 (0.15, 0.34) |  | 223.32 | 2.37 (1.90, 2.84) |
| Mexico | 78.26 (67.73, 91.18) | 0.19 (0.16, 0.22) |  | 404.04 (324.31, 493.46) | 0.35 (0.28, 0.43) |  | 416.30 | 2.38 (2.23, 2.53) |
| Moldova | 3.42 (2.41, 4.85) | 0.08 (0.06, 0.11) |  | 8.61 (5.91, 12.46) | 0.15 (0.10, 0.22) |  | 151.83 | 0.55 (-0.90, 2.02) |
| Monaco | 0.17 (0.11, 0.27) | 0.24 (0.15, 0.37) |  | 0.67 (0.42, 1.05) | 0.72 (0.47, 1.10) |  | 288.07 | 4.45 (3.54, 5.37) |
| Mongolia | 33.40 (21.43, 50.38) | 3.27 (2.12, 4.90) |  | 146.16 (92.50, 219.51) | 7.62 (4.88, 11.42) |  | 337.58 | 3.90 (3.28, 4.52) |
| Montenegro | 2.57 (1.73, 3.70) | 0.42 (0.28, 0.60) |  | 4.82 (3.22, 7.06) | 0.49 (0.34, 0.72) |  | 87.34 | 0.46 (0.27, 0.65) |
| Morocco | 21.85 (13.57, 32.98) | 0.18 (0.11, 0.27) |  | 72.11 (46.60, 108.37) | 0.26 (0.17, 0.38) |  | 230.08 | 1.19 (0.92, 1.45) |
| Mozambique | 12.75 (7.98, 20.06) | 0.24 (0.15, 0.37) |  | 42.37 (27.46, 63.13) | 0.42 (0.27, 0.63) |  | 232.27 | 1.90 (1.69, 2.10) |
| Myanmar | 53.02 (31.67, 85.04) | 0.25 (0.15, 0.40) |  | 193.55 (130.31, 282.60) | 0.44 (0.30, 0.64) |  | 265.08 | 2.03 (1.85, 2.20) |
| Namibia | 1.16 (0.64, 2.08) | 0.16 (0.09, 0.29) |  | 4.12 (2.67, 6.10) | 0.30 (0.19, 0.44) |  | 256.87 | 1.99 (1.60, 2.38) |
| Nauru | 0.03 (0.02, 0.05) | 0.85 (0.55, 1.25) |  | 0.04 (0.02, 0.06) | 0.89 (0.57, 1.38) |  | 18.30 | -0.22 (-0.44, -0.00) |
| Nepal | 13.07 (8.68, 19.98) | 0.15 (0.10, 0.22) |  | 44.53 (28.35, 68.35) | 0.21 (0.13, 0.33) |  | 240.80 | 1.36 (1.17, 1.55) |
| Netherlands | 20.00 (13.93, 28.72) | 0.10 (0.07, 0.14) |  | 87.96 (56.48, 131.75) | 0.26 (0.17, 0.39) |  | 339.72 | 3.82 (3.64, 4.00) |
| New Zealand | 7.33 (6.12, 8.68) | 0.19 (0.16, 0.22) |  | 39.24 (30.85, 48.70) | 0.52 (0.41, 0.65) |  | 435.22 | 3.65 (3.39, 3.91) |
| Nicaragua | 3.22 (2.28, 4.45) | 0.20 (0.14, 0.29) |  | 13.69 (9.07, 19.58) | 0.32 (0.21, 0.46) |  | 325.93 | 1.65 (1.24, 2.07) |
| Niger | 1.19 (0.78, 1.79) | 0.04 (0.03, 0.07) |  | 3.54 (2.21, 5.40) | 0.05 (0.03, 0.07) |  | 197.60 | 0.04 (-0.04, 0.13) |
| Nigeria | 125.15 (90.22, 167.54) | 0.30 (0.22, 0.40) |  | 268.85 (200.85, 345.57) | 0.34 (0.26, 0.43) |  | 114.82 | 0.52 (0.46, 0.58) |
| Niue | 0.02 (0.01, 0.02) | 0.68 (0.46, 1.02) |  | 0.02 (0.01, 0.02) | 0.79 (0.51, 1.15) |  | 12.35 | 0.42 (0.30, 0.54) |
| North Korea | 110.09 (70.59, 170.12) | 0.68 (0.43, 1.03) |  | 158.72 (97.83, 244.89) | 0.49 (0.31, 0.76) |  | 44.18 | -1.34 (-1.47, -1.22) |
| Northern Mariana Islands | 0.12 (0.08, 0.17) | 0.67 (0.46, 0.96) |  | 0.40 (0.26, 0.61) | 0.78 (0.53, 1.13) |  | 234.35 | 0.48 (0.39, 0.57) |
| Norway | 6.62 (5.50, 7.84) | 0.10 (0.08, 0.11) |  | 17.63 (13.82, 22.35) | 0.19 (0.15, 0.24) |  | 166.51 | 2.76 (2.59, 2.94) |
| Oman | 2.04 (1.18, 3.26) | 0.32 (0.18, 0.52) |  | 10.52 (7.27, 15.18) | 0.66 (0.45, 0.97) |  | 417.13 | 3.51 (3.09, 3.93) |
| Pakistan | 140.56 (92.94, 199.24) | 0.23 (0.15, 0.34) |  | 318.61 (240.22, 408.81) | 0.27 (0.20, 0.35) |  | 126.67 | 0.44 (0.32, 0.56) |
| Palau | 0.06 (0.04, 0.10) | 0.64 (0.38, 1.02) |  | 0.18 (0.11, 0.29) | 0.86 (0.54, 1.28) |  | 190.62 | 0.90 (0.69, 1.11) |
| Palestine | 6.48 (4.00, 10.11) | 0.78 (0.47, 1.22) |  | 14.76 (10.10, 21.14) | 0.69 (0.47, 1.00) |  | 127.87 | -0.49 (-0.55, -0.42) |
| Panama | 3.21 (2.27, 4.51) | 0.21 (0.15, 0.30) |  | 9.86 (6.27, 14.75) | 0.24 (0.15, 0.36) |  | 206.71 | 0.71 (0.28, 1.15) |
| Papua New Guinea | 1.40 (0.86, 2.20) | 0.09 (0.06, 0.14) |  | 4.27 (2.72, 6.52) | 0.11 (0.07, 0.18) |  | 205.98 | 0.79 (0.73, 0.85) |
| Paraguay | 2.52 (1.71, 3.65) | 0.11 (0.08, 0.17) |  | 6.09 (3.86, 9.15) | 0.11 (0.07, 0.17) |  | 141.91 | 0.08 (-0.49, 0.65) |
| Peru | 50.43 (34.67, 72.77) | 0.44 (0.29, 0.63) |  | 78.68 (48.84, 114.60) | 0.25 (0.15, 0.36) |  | 56.02 | -2.94 (-3.50, -2.37) |
| Philippines | 195.75 (146.85, 251.83) | 0.65 (0.49, 0.84) |  | 398.42 (311.09, 505.89) | 0.52 (0.41, 0.65) |  | 103.54 | -1.13 (-1.43, -0.83) |
| Poland | 238.01 (201.52, 279.50) | 0.56 (0.47, 0.65) |  | 111.14 (87.86, 139.28) | 0.16 (0.12, 0.19) |  | -53.30 | -4.28 (-5.66, -2.88) |
| Portugal | 13.84 (9.74, 20.02) | 0.10 (0.07, 0.14) |  | 65.79 (41.28, 99.80) | 0.29 (0.18, 0.44) |  | 375.28 | 3.27 (2.91, 3.64) |
| Puerto Rico | 24.28 (17.01, 34.08) | 0.66 (0.46, 0.92) |  | 25.23 (16.36, 37.85) | 0.35 (0.23, 0.52) |  | 3.94 | -0.99 (-1.70, -0.27) |
| Qatar | 1.55 (0.98, 2.31) | 2.04 (1.26, 3.16) |  | 14.58 (8.68, 23.18) | 2.84 (1.77, 4.32) |  | 843.54 | 1.90 (1.70, 2.10) |
| Romania | 29.85 (21.14, 43.10) | 0.11 (0.08, 0.16) |  | 81.63 (53.99, 119.64) | 0.22 (0.15, 0.32) |  | 173.49 | 3.23 (2.77, 3.69) |
| Russia | 206.94 (176.48, 244.76) | 0.12 (0.10, 0.14) |  | 548.17 (437.32, 670.90) | 0.24 (0.19, 0.29) |  | 164.89 | 2.96 (2.63, 3.30) |
| Rwanda | 15.23 (9.12, 23.98) | 0.53 (0.32, 0.83) |  | 30.63 (19.75, 44.63) | 0.53 (0.34, 0.76) |  | 101.11 | -0.61 (-0.82, -0.41) |
| Saint Kitts and Nevis | 0.42 (0.29, 0.60) | 1.09 (0.77, 1.53) |  | 0.23 (0.15, 0.33) | 0.37 (0.26, 0.53) |  | -45.80 | -4.03 (-5.09, -2.96) |
| Saint Lucia | 0.39 (0.27, 0.55) | 0.45 (0.31, 0.62) |  | 0.41 (0.28, 0.57) | 0.19 (0.13, 0.27) |  | 3.84 | -3.37 (-4.44, -2.29) |
| Saint Vincent and the Grenadines | 0.40 (0.28, 0.56) | 0.54 (0.38, 0.77) |  | 0.35 (0.24, 0.49) | 0.26 (0.18, 0.37) |  | -11.82 | -2.45 (-3.39, -1.50) |
| Samoa | 0.41 (0.27, 0.62) | 0.47 (0.31, 0.71) |  | 0.64 (0.41, 0.94) | 0.44 (0.29, 0.65) |  | 56.00 | -0.46 (-0.55, -0.36) |
| San Marino | 0.04 (0.03, 0.07) | 0.13 (0.09, 0.20) |  | 0.13 (0.08, 0.21) | 0.21 (0.14, 0.34) |  | 202.86 | 2.30 (2.07, 2.53) |
| Sao Tome and Principe | 0.13 (0.09, 0.19) | 0.20 (0.14, 0.30) |  | 0.27 (0.17, 0.40) | 0.26 (0.16, 0.39) |  | 109.77 | 0.84 (0.72, 0.95) |
| Saudi Arabia | 27.68 (17.15, 41.52) | 0.52 (0.32, 0.78) |  | 122.86 (81.16, 186.73) | 0.83 (0.55, 1.24) |  | 343.93 | 1.50 (1.20, 1.81) |
| Senegal | 7.36 (4.87, 10.54) | 0.24 (0.16, 0.34) |  | 18.82 (12.02, 27.58) | 0.26 (0.17, 0.39) |  | 155.59 | 0.46 (0.34, 0.59) |
| Serbia | 38.49 (24.79, 57.06) | 0.36 (0.23, 0.52) |  | 61.42 (39.25, 91.04) | 0.38 (0.25, 0.56) |  | 59.56 | -0.04 (-0.25, 0.18) |
| Seychelles | 0.39 (0.28, 0.55) | 0.69 (0.49, 0.97) |  | 0.63 (0.43, 0.90) | 0.59 (0.41, 0.83) |  | 59.88 | -0.30 (-0.58, -0.02) |
| Sierra Leone | 8.03 (5.12, 12.01) | 0.43 (0.27, 0.63) |  | 15.01 (9.66, 22.58) | 0.43 (0.28, 0.65) |  | 86.93 | -0.01 (-0.08, 0.06) |
| Singapore | 6.58 (4.36, 9.79) | 0.32 (0.21, 0.48) |  | 41.66 (25.44, 63.18) | 0.56 (0.34, 0.87) |  | 533.01 | 2.06 (1.79, 2.33) |
| Slovakia | 16.93 (11.71, 23.35) | 0.28 (0.20, 0.39) |  | 23.52 (14.97, 35.49) | 0.26 (0.17, 0.38) |  | 38.95 | -0.97 (-1.30, -0.63) |
| Slovenia | 5.08 (3.26, 7.62) | 0.21 (0.13, 0.31) |  | 20.35 (12.43, 31.58) | 0.46 (0.29, 0.70) |  | 300.93 | 3.22 (2.90, 3.53) |
| Solomon Islands | 0.76 (0.48, 1.17) | 0.49 (0.31, 0.78) |  | 1.61 (1.08, 2.33) | 0.48 (0.32, 0.69) |  | 112.69 | -0.12 (-0.32, 0.07) |
| Somalia | 9.66 (5.21, 16.80) | 0.40 (0.22, 0.68) |  | 25.13 (14.70, 44.25) | 0.41 (0.24, 0.71) |  | 160.09 | 0.15 (0.06, 0.24) |
| South Africa | 128.50 (83.15, 211.41) | 0.59 (0.37, 0.98) |  | 299.08 (251.64, 357.47) | 0.67 (0.57, 0.81) |  | 132.74 | 0.13 (-0.46, 0.72) |
| South Korea | 148.20 (98.08, 213.13) | 0.53 (0.36, 0.76) |  | 1118.59 (730.77, 1649.53) | 1.25 (0.83, 1.83) |  | 654.79 | 3.62 (2.43, 4.82) |
| South Sudan | 8.37 (4.73, 14.54) | 0.36 (0.20, 0.63) |  | 12.99 (6.98, 22.85) | 0.36 (0.20, 0.61) |  | 55.13 | 0.04 (0.01, 0.08) |
| Spain | 101.23 (68.41, 147.37) | 0.18 (0.13, 0.27) |  | 299.70 (185.69, 456.08) | 0.32 (0.20, 0.49) |  | 196.07 | 1.73 (1.26, 2.21) |
| Sri Lanka | 18.40 (12.87, 25.96) | 0.18 (0.12, 0.26) |  | 68.34 (42.43, 107.58) | 0.28 (0.17, 0.43) |  | 271.45 | 2.31 (2.02, 2.61) |
| Sudan | 31.71 (16.48, 51.93) | 0.36 (0.18, 0.59) |  | 85.97 (49.22, 137.97) | 0.50 (0.29, 0.80) |  | 171.15 | 1.25 (1.02, 1.49) |
| Suriname | 1.28 (0.90, 1.83) | 0.50 (0.34, 0.72) |  | 1.44 (0.97, 2.11) | 0.25 (0.17, 0.36) |  | 13.08 | -2.15 (-2.93, -1.36) |
| Sweden | 26.22 (21.61, 31.83) | 0.17 (0.14, 0.21) |  | 45.87 (35.71, 56.53) | 0.22 (0.18, 0.28) |  | 74.93 | 1.38 (0.76, 2.00) |
| Switzerland | 13.49 (9.15, 19.70) | 0.13 (0.09, 0.18) |  | 55.45 (34.25, 87.37) | 0.32 (0.20, 0.50) |  | 311.03 | 2.58 (2.04, 3.13) |
| Syrian Arab Republic | 24.25 (15.57, 36.22) | 0.49 (0.31, 0.76) |  | 63.74 (40.13, 97.86) | 0.56 (0.36, 0.85) |  | 162.86 | 0.40 (0.25, 0.55) |
| Taiwan (Province of China) | 102.65 (71.56, 148.11) | 0.62 (0.43, 0.89) |  | 266.06 (169.97, 403.52) | 0.68 (0.43, 1.03) |  | 159.19 | -0.63 (-1.80, 0.56) |
| Tajikistan | 1.27 (0.89, 1.79) | 0.05 (0.03, 0.07) |  | 9.46 (6.26, 13.87) | 0.22 (0.14, 0.32) |  | 647.57 | 6.24 (5.92, 6.57) |
| Tanzania | 25.16 (17.52, 35.85) | 0.24 (0.17, 0.34) |  | 71.67 (48.95, 102.49) | 0.30 (0.20, 0.43) |  | 184.90 | 0.79 (0.68, 0.91) |
| Thailand | 519.95 (355.95, 740.99) | 1.50 (1.03, 2.14) |  | 2374.86 (1491.47, 3588.37) | 2.34 (1.48, 3.51) |  | 356.75 | 1.76 (1.62, 1.90) |
| Timor-Leste | 1.56 (0.93, 2.47) | 0.56 (0.34, 0.89) |  | 4.08 (2.43, 6.29) | 0.52 (0.31, 0.79) |  | 162.53 | -0.49 (-0.69, -0.28) |
| Togo | 5.96 (4.03, 8.78) | 0.49 (0.33, 0.72) |  | 16.63 (10.83, 25.65) | 0.48 (0.32, 0.75) |  | 178.85 | -0.51 (-0.64, -0.38) |
| Tokelau | 0.01 (0.01, 0.01) | 0.68 (0.43, 1.03) |  | 0.01 (0.01, 0.01) | 0.72 (0.46, 1.07) |  | 1.72 | 0.02 (-0.06, 0.09) |
| Tonga | 1.02 (0.61, 1.59) | 1.82 (1.10, 2.78) |  | 1.86 (1.19, 2.75) | 2.34 (1.47, 3.47) |  | 83.54 | 0.58 (0.20, 0.97) |
| Trinidad and Tobago | 4.93 (3.52, 6.99) | 0.59 (0.42, 0.84) |  | 4.95 (3.20, 7.53) | 0.27 (0.18, 0.41) |  | 0.44 | -2.78 (-3.86, -1.69) |
| Tunisia | 5.51 (3.53, 8.72) | 0.11 (0.07, 0.18) |  | 18.75 (11.08, 30.23) | 0.15 (0.09, 0.25) |  | 240.06 | 1.33 (1.19, 1.48) |
| Turkey | 104.99 (68.73, 156.31) | 0.31 (0.20, 0.46) |  | 315.10 (210.97, 455.57) | 0.37 (0.25, 0.54) |  | 200.11 | 0.72 (0.32, 1.13) |
| Turkmenistan | 1.17 (0.81, 1.65) | 0.07 (0.04, 0.09) |  | 16.14 (10.46, 24.03) | 0.40 (0.26, 0.60) |  | 1281.24 | 7.29 (6.26, 8.33) |
| Tuvalu | 0.05 (0.03, 0.08) | 0.77 (0.49, 1.19) |  | 0.07 (0.05, 0.12) | 0.75 (0.47, 1.13) |  | 46.02 | -0.34 (-0.44, -0.23) |
| Uganda | 27.46 (18.51, 39.75) | 0.43 (0.29, 0.62) |  | 99.42 (67.65, 146.85) | 0.70 (0.46, 1.05) |  | 262.04 | 1.82 (1.59, 2.05) |
| Ukraine | 51.80 (43.26, 61.52) | 0.07 (0.06, 0.09) |  | 154.99 (123.97, 187.68) | 0.21 (0.17, 0.25) |  | 199.21 | 4.41 (3.96, 4.86) |
| United Arab Emirates | 1.63 (0.83, 3.00) | 0.43 (0.20, 0.87) |  | 21.87 (8.69, 53.96) | 0.61 (0.24, 1.52) |  | 1241.87 | 1.22 (0.84, 1.60) |
| United Kingdom | 112.74 (95.03, 133.35) | 0.12 (0.11, 0.14) |  | 452.88 (355.45, 568.41) | 0.36 (0.28, 0.45) |  | 301.72 | 4.44 (4.21, 4.67) |
| United States of America | 661.10 (568.58, 766.98) | 0.20 (0.18, 0.24) |  | 2790.69 (2183.64, 3465.14) | 0.51 (0.40, 0.63) |  | 322.13 | 3.28 (3.03, 3.54) |
| United States Virgin Islands | 0.33 (0.22, 0.48) | 0.41 (0.27, 0.58) |  | 0.56 (0.38, 0.83) | 0.30 (0.21, 0.43) |  | 70.83 | -1.16 (-1.69, -0.64) |
| Uruguay | 4.31 (2.90, 6.22) | 0.11 (0.07, 0.16) |  | 12.08 (7.87, 18.40) | 0.22 (0.14, 0.33) |  | 180.51 | 2.82 (2.65, 2.98) |
| Uzbekistan | 5.73 (3.98, 8.19) | 0.05 (0.04, 0.08) |  | 97.28 (64.70, 141.51) | 0.51 (0.35, 0.73) |  | 1598.14 | 10.26 (9.12, 11.42) |
| Vanuatu | 0.40 (0.23, 0.67) | 0.63 (0.35, 1.04) |  | 1.26 (0.75, 1.98) | 0.74 (0.45, 1.14) |  | 212.09 | 0.55 (0.48, 0.62) |
| Venezuela | 42.36 (29.66, 59.42) | 0.44 (0.31, 0.62) |  | 54.44 (35.62, 84.96) | 0.19 (0.13, 0.30) |  | 28.51 | -2.12 (-3.36, -0.87) |
| Viet Nam | 117.96 (73.02, 177.25) | 0.30 (0.19, 0.46) |  | 205.54 (129.28, 313.78) | 0.24 (0.16, 0.37) |  | 74.25 | -0.96 (-1.16, -0.77) |
| Yemen | 10.48 (5.52, 17.32) | 0.23 (0.12, 0.39) |  | 33.57 (20.81, 53.39) | 0.27 (0.17, 0.43) |  | 220.33 | 0.73 (0.61, 0.85) |
| Zambia | 8.00 (5.38, 12.13) | 0.29 (0.19, 0.44) |  | 23.55 (15.37, 34.63) | 0.37 (0.24, 0.53) |  | 194.54 | 0.21 (-0.10, 0.51) |
| Zimbabwe | 37.42 (23.82, 60.58) | 0.91 (0.57, 1.50) |  | 88.82 (56.81, 133.14) | 1.25 (0.80, 1.90) |  | 137.39 | -0.03 (-0.62, 0.57) |

ASIR: age-standardized incidence rate; CI: confidence interval; EAPC: estimated annual percentage change; NASH: nonalcoholic steatohepatitis; UI: uncertainty interval.


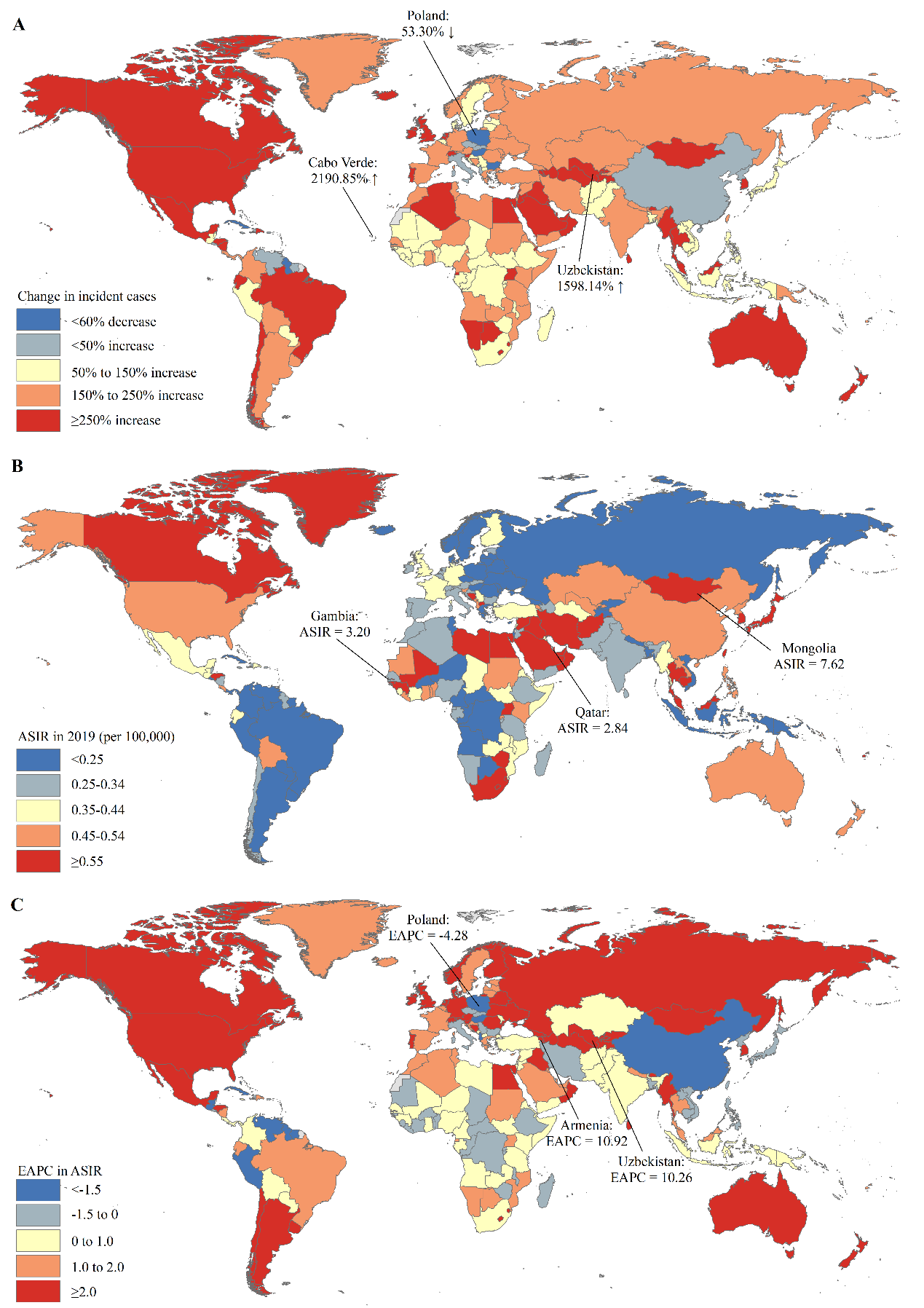


**Figure S4.** The global trends in the incidence of primary liver cancer due to NASH in 204 countries and territories. (A) The percentage change in incident cases of primary liver cancer due to NASH between 1990 and 2019; (B) The ASIR of primary liver cancer due to NASH in 2019; (C) The EAPCs in ASIR of primary liver cancer due to NASH from 1990 to 2019.

Note: ASIR: age-standardized incidence rate; EAPC: estimated annual percentage change; NASH: nonalcoholic steatohepatitis.

**Table S17**. **The incident cases and ASIR of primary liver cancer due to other causes in 1990 and 2019 and their change trends from 1990 to 2019 at national level.**

| Nation | 1990 | |  | 2019 | |  | 1990-2019 | |
| --- | --- | --- | --- | --- | --- | --- | --- | --- |
|  | Incident cases  No. (95% UI) | ASIR per 100,000  No. (95% UI) |  | Incident cases  No. x 10^3^ (95% UI) | ASIR per 100,000  No. (95% UI) |  | Percentage change in incident cases  No. (%) | EAPC in ASIR  No. (95% CI) |
| Afghanistan | 69.17 (45.77, 100.01) | 0.84 (0.55, 1.22) |  | 145.49 (101.00, 208.50) | 0.75 (0.49, 1.09) |  | 110.34 | -0.38 (-0.51, -0.25) |
| Albania | 11.03 (8.21, 14.49) | 0.47 (0.33, 0.63) |  | 9.78 (6.05, 14.83) | 0.27 (0.18, 0.39) |  | -11.30 | -2.80 (-3.28, -2.32) |
| Algeria | 23.45 (17.15, 31.08) | 0.13 (0.09, 0.17) |  | 57.00 (40.07, 79.27) | 0.16 (0.11, 0.22) |  | 143.10 | 0.77 (0.67, 0.86) |
| American Samoa | 0.07 (0.05, 0.10) | 0.24 (0.16, 0.36) |  | 0.15 (0.10, 0.22) | 0.28 (0.19, 0.41) |  | 107.79 | 0.90 (0.69, 1.10) |
| Andorra | 0.26 (0.16, 0.38) | 0.49 (0.32, 0.72) |  | 0.69 (0.43, 1.05) | 0.56 (0.37, 0.84) |  | 167.95 | 0.61 (0.57, 0.65) |
| Angola | 18.09 (9.92, 32.63) | 0.16 (0.10, 0.24) |  | 40.28 (22.66, 67.24) | 0.15 (0.09, 0.22) |  | 122.61 | -0.40 (-0.49, -0.30) |
| Antigua and Barbuda | 0.26 (0.19, 0.36) | 0.49 (0.35, 0.66) |  | 0.17 (0.11, 0.23) | 0.17 (0.12, 0.24) |  | -37.54 | -3.85 (-4.77, -2.93) |
| Argentina | 34.27 (25.72, 44.84) | 0.10 (0.08, 0.14) |  | 66.50 (44.20, 94.97) | 0.13 (0.09, 0.19) |  | 94.02 | 1.39 (1.21, 1.58) |
| Armenia | 4.39 (2.98, 6.63) | 0.13 (0.09, 0.19) |  | 9.71 (6.35, 13.95) | 0.27 (0.18, 0.40) |  | 121.43 | 4.35 (3.51, 5.21) |
| Australia | 20.89 (16.18, 27.04) | 0.12 (0.10, 0.15) |  | 82.35 (53.46, 122.54) | 0.24 (0.16, 0.35) |  | 294.14 | 2.77 (2.54, 3.01) |
| Austria | 19.98 (14.77, 26.86) | 0.21 (0.16, 0.27) |  | 41.74 (26.90, 61.28) | 0.30 (0.20, 0.42) |  | 108.95 | 1.45 (1.16, 1.75) |
| Azerbaijan | 8.42 (6.44, 12.52) | 0.11 (0.09, 0.16) |  | 18.27 (12.15, 28.73) | 0.20 (0.14, 0.32) |  | 116.91 | 2.62 (1.53, 3.72) |
| Bahamas | 0.87 (0.64, 1.16) | 0.49 (0.35, 0.67) |  | 0.79 (0.55, 1.10) | 0.20 (0.14, 0.28) |  | -8.83 | -3.28 (-4.10, -2.46) |
| Bahrain | 0.64 (0.46, 0.87) | 0.31 (0.22, 0.46) |  | 2.92 (1.94, 4.35) | 0.32 (0.22, 0.48) |  | 358.01 | -0.58 (-1.00, -0.16) |
| Bangladesh | 201.11 (133.44, 292.76) | 0.18 (0.13, 0.25) |  | 237.80 (169.36, 328.39) | 0.16 (0.11, 0.22) |  | 18.24 | -0.98 (-1.20, -0.77) |
| Barbados | 0.64 (0.42, 0.94) | 0.24 (0.16, 0.36) |  | 0.81 (0.54, 1.14) | 0.23 (0.15, 0.32) |  | 25.61 | -0.27 (-0.39, -0.15) |
| Belarus | 13.54 (9.81, 18.34) | 0.14 (0.10, 0.19) |  | 17.62 (12.36, 24.73) | 0.18 (0.13, 0.25) |  | 30.08 | 1.57 (1.28, 1.86) |
| Belgium | 22.31 (16.14, 31.06) | 0.17 (0.13, 0.23) |  | 41.82 (27.04, 61.45) | 0.23 (0.16, 0.33) |  | 87.41 | 0.95 (0.68, 1.23) |
| Belize | 0.53 (0.41, 0.67) | 0.45 (0.33, 0.60) |  | 0.62 (0.45, 0.84) | 0.20 (0.14, 0.27) |  | 18.14 | -3.03 (-3.64, -2.42) |
| Benin | 10.46 (7.33, 14.70) | 0.32 (0.22, 0.45) |  | 19.39 (12.59, 28.36) | 0.26 (0.17, 0.39) |  | 85.44 | -1.01 (-1.17, -0.85) |
| Bermuda | 0.24 (0.17, 0.33) | 0.40 (0.29, 0.54) |  | 0.13 (0.08, 0.19) | 0.13 (0.09, 0.18) |  | -46.34 | -3.66 (-4.59, -2.73) |
| Bhutan | 0.45 (0.20, 0.73) | 0.09 (0.05, 0.14) |  | 0.85 (0.56, 1.27) | 0.14 (0.09, 0.21) |  | 87.63 | 1.39 (1.27, 1.52) |
| Bolivia | 16.65 (10.84, 25.42) | 0.40 (0.25, 0.64) |  | 32.38 (20.67, 47.91) | 0.35 (0.22, 0.52) |  | 94.48 | -0.51 (-0.61, -0.41) |
| Bosnia and Herzegovina | 9.04 (6.31, 12.78) | 0.22 (0.15, 0.30) |  | 15.97 (9.70, 24.67) | 0.29 (0.19, 0.43) |  | 76.65 | 1.63 (1.26, 2.01) |
| Botswana | 0.40 (0.22, 0.75) | 0.05 (0.03, 0.11) |  | 1.29 (0.81, 1.90) | 0.07 (0.05, 0.11) |  | 220.92 | 0.64 (0.10, 1.18) |
| Brazil | 159.03 (141.35, 179.66) | 0.13 (0.12, 0.15) |  | 305.61 (264.87, 349.69) | 0.14 (0.12, 0.16) |  | 92.16 | 0.51 (0.39, 0.63) |
| Brunei | 0.43 (0.29, 0.61) | 0.38 (0.25, 0.57) |  | 1.64 (1.06, 2.35) | 0.49 (0.33, 0.71) |  | 282.27 | 1.50 (1.05, 1.96) |
| Bulgaria | 26.46 (18.16, 36.80) | 0.23 (0.17, 0.31) |  | 18.49 (11.64, 27.12) | 0.16 (0.11, 0.23) |  | -30.14 | -0.62 (-1.06, -0.17) |
| Burkina Faso | 23.20 (12.83, 38.27) | 0.21 (0.14, 0.30) |  | 43.92 (25.45, 72.49) | 0.18 (0.12, 0.27) |  | 89.32 | -0.59 (-0.83, -0.34) |
| Burundi | 12.09 (7.42, 19.50) | 0.29 (0.19, 0.44) |  | 17.72 (11.58, 26.79) | 0.23 (0.15, 0.35) |  | 46.50 | -1.20 (-1.32, -1.08) |
| Cambodia | 115.24 (48.50, 246.95) | 0.79 (0.43, 1.48) |  | 85.72 (47.17, 152.89) | 0.54 (0.31, 0.92) |  | -25.62 | -1.61 (-1.78, -1.45) |
| Cameroon | 2.00 (1.36, 2.87) | 0.03 (0.02, 0.05) |  | 6.52 (4.26, 9.54) | 0.03 (0.02, 0.05) |  | 225.69 | 0.22 (0.08, 0.36) |
| Canada | 48.70 (37.54, 62.81) | 0.17 (0.13, 0.22) |  | 180.51 (118.05, 265.79) | 0.33 (0.23, 0.47) |  | 270.63 | 2.73 (2.56, 2.89) |
| Cape Verde | 0.19 (0.14, 0.25) | 0.06 (0.05, 0.09) |  | 2.57 (1.80, 3.62) | 0.57 (0.39, 0.82) |  | 1248.56 | 6.21 (4.17, 8.29) |
| Central African Republic | 5.82 (3.16, 9.56) | 0.22 (0.13, 0.33) |  | 7.91 (4.55, 12.99) | 0.18 (0.11, 0.30) |  | 36.02 | -0.89 (-1.02, -0.76) |
| Chad | 13.20 (8.84, 18.36) | 0.31 (0.21, 0.44) |  | 25.48 (17.02, 35.45) | 0.28 (0.18, 0.41) |  | 93.09 | -0.53 (-0.60, -0.47) |
| Chile | 16.72 (12.66, 22.11) | 0.15 (0.11, 0.20) |  | 43.19 (28.02, 63.71) | 0.20 (0.14, 0.29) |  | 158.30 | 1.53 (1.26, 1.79) |
| China | 16605.36 (13559.50, 20527.19) | 1.72 (1.41, 2.14) |  | 12175.98 (9859.27, 15010.90) | 0.64 (0.53, 0.78) |  | -26.67 | -4.92 (-5.74, -4.09) |
| Colombia | 44.12 (34.47, 55.03) | 0.18 (0.14, 0.23) |  | 77.00 (51.86, 108.51) | 0.16 (0.11, 0.22) |  | 74.53 | -0.15 (-0.56, 0.25) |
| Comoros | 0.88 (0.43, 1.38) | 0.27 (0.13, 0.43) |  | 1.31 (0.81, 2.00) | 0.24 (0.15, 0.36) |  | 49.30 | -0.64 (-0.84, -0.44) |
| Congo | 4.73 (2.93, 7.22) | 0.22 (0.15, 0.31) |  | 5.82 (3.81, 8.63) | 0.15 (0.09, 0.22) |  | 23.05 | -1.70 (-1.81, -1.59) |
| Cook Islands | 0.08 (0.06, 0.12) | 0.58 (0.39, 0.83) |  | 0.10 (0.07, 0.15) | 0.43 (0.28, 0.63) |  | 22.22 | -0.97 (-1.05, -0.89) |
| Costa Rica | 6.46 (4.96, 8.30) | 0.30 (0.22, 0.41) |  | 13.86 (9.17, 19.99) | 0.28 (0.19, 0.40) |  | 114.55 | -0.40 (-0.92, 0.11) |
| Cote d'Ivoire | 22.81 (15.08, 32.55) | 0.33 (0.21, 0.47) |  | 34.72 (22.48, 50.45) | 0.23 (0.14, 0.35) |  | 52.22 | -1.84 (-2.09, -1.58) |
| Croatia | 8.63 (6.07, 11.92) | 0.15 (0.11, 0.20) |  | 11.31 (7.19, 16.73) | 0.16 (0.11, 0.22) |  | 31.11 | 0.68 (0.35, 1.02) |
| Cuba | 43.88 (31.94, 58.51) | 0.42 (0.31, 0.57) |  | 23.12 (14.96, 33.54) | 0.14 (0.10, 0.20) |  | -47.31 | -4.15 (-5.22, -3.08) |
| Cyprus | 1.45 (1.03, 2.01) | 0.19 (0.13, 0.25) |  | 3.55 (2.46, 4.97) | 0.22 (0.16, 0.30) |  | 144.69 | 1.01 (0.82, 1.20) |
| Czech Republic | 19.36 (13.70, 26.81) | 0.16 (0.12, 0.21) |  | 17.67 (11.57, 25.68) | 0.11 (0.08, 0.15) |  | -8.74 | -1.32 (-1.46, -1.18) |
| Democratic Republic of the Congo | 86.49 (50.01, 148.79) | 0.18 (0.12, 0.26) |  | 120.06 (64.32, 216.58) | 0.15 (0.09, 0.23) |  | 38.80 | -0.68 (-0.73, -0.64) |
| Denmark | 10.00 (7.01, 13.85) | 0.16 (0.12, 0.20) |  | 20.86 (13.25, 31.07) | 0.24 (0.16, 0.33) |  | 108.63 | 1.72 (1.54, 1.89) |
| Djibouti | 0.78 (0.47, 1.30) | 0.26 (0.16, 0.42) |  | 2.04 (1.17, 3.33) | 0.25 (0.15, 0.41) |  | 161.51 | -0.31 (-0.41, -0.20) |
| Dominica | 0.41 (0.29, 0.56) | 0.58 (0.41, 0.79) |  | 0.17 (0.11, 0.24) | 0.20 (0.14, 0.28) |  | -59.61 | -3.68 (-4.43, -2.93) |
| Dominican Republic | 16.72 (13.01, 20.76) | 0.31 (0.24, 0.40) |  | 31.34 (20.58, 46.49) | 0.32 (0.21, 0.48) |  | 87.47 | 0.37 (-0.12, 0.85) |
| Ecuador | 15.31 (11.51, 19.65) | 0.23 (0.17, 0.31) |  | 38.72 (26.21, 54.62) | 0.25 (0.17, 0.36) |  | 152.86 | 0.84 (0.48, 1.20) |
| Egypt | 353.58 (254.99, 493.58) | 0.85 (0.59, 1.19) |  | 794.07 (500.43, 1237.82) | 1.06 (0.66, 1.67) |  | 124.58 | 1.67 (1.27, 2.08) |
| El Salvador | 9.69 (7.65, 12.22) | 0.25 (0.18, 0.33) |  | 7.11 (4.77, 10.23) | 0.12 (0.08, 0.17) |  | -26.69 | -2.49 (-3.25, -1.73) |
| Equatorial Guinea | 0.81 (0.49, 1.26) | 0.18 (0.12, 0.26) |  | 1.34 (0.76, 2.29) | 0.15 (0.08, 0.24) |  | 66.14 | -0.79 (-0.88, -0.69) |
| Eritrea | 4.98 (3.00, 7.85) | 0.28 (0.17, 0.44) |  | 10.61 (6.90, 16.13) | 0.27 (0.17, 0.40) |  | 113.15 | -0.29 (-0.41, -0.17) |
| Estonia | 2.21 (1.47, 3.12) | 0.13 (0.08, 0.19) |  | 2.94 (1.87, 4.40) | 0.14 (0.10, 0.21) |  | 33.08 | -0.31 (-0.59, -0.03) |
| Eswatini | 1.16 (0.71, 2.00) | 0.29 (0.17, 0.51) |  | 5.37 (1.80, 10.65) | 0.74 (0.25, 1.44) |  | 364.87 | 4.24 (3.19, 5.29) |
| Ethiopia | 110.61 (66.89, 192.86) | 0.24 (0.16, 0.38) |  | 135.57 (103.42, 178.42) | 0.19 (0.15, 0.24) |  | 22.58 | -1.10 (-1.17, -1.02) |
| Federated States of Micronesia | 0.28 (0.18, 0.41) | 0.49 (0.30, 0.73) |  | 0.36 (0.19, 0.60) | 0.44 (0.25, 0.73) |  | 26.91 | -0.38 (-0.48, -0.29) |
| Fiji | 1.30 (0.89, 1.85) | 0.27 (0.18, 0.40) |  | 2.33 (1.51, 3.44) | 0.28 (0.18, 0.42) |  | 78.90 | 0.58 (0.24, 0.93) |
| Finland | 12.76 (9.19, 17.29) | 0.20 (0.15, 0.26) |  | 29.97 (18.70, 45.66) | 0.31 (0.21, 0.44) |  | 134.86 | 1.68 (1.53, 1.84) |
| France | 174.12 (124.30, 245.09) | 0.24 (0.18, 0.33) |  | 375.29 (238.82, 554.15) | 0.35 (0.23, 0.51) |  | 115.53 | 1.14 (0.91, 1.37) |
| Gabon | 1.81 (1.04, 2.89) | 0.18 (0.11, 0.27) |  | 1.99 (1.25, 2.90) | 0.14 (0.09, 0.21) |  | 10.20 | -1.03 (-1.09, -0.96) |
| Gambia | 9.69 (6.21, 13.90) | 1.46 (0.93, 2.10) |  | 26.22 (17.58, 36.05) | 1.91 (1.25, 2.79) |  | 170.73 | 0.65 (0.46, 0.84) |
| Georgia | 5.43 (3.35, 8.20) | 0.10 (0.06, 0.16) |  | 6.46 (4.32, 9.26) | 0.14 (0.09, 0.20) |  | 19.14 | 0.39 (-0.24, 1.03) |
| Germany | 207.19 (151.89, 281.04) | 0.19 (0.15, 0.25) |  | 513.70 (333.85, 757.86) | 0.33 (0.23, 0.48) |  | 147.93 | 2.02 (1.63, 2.41) |
| Ghana | 26.93 (18.85, 37.07) | 0.26 (0.18, 0.37) |  | 59.45 (40.60, 82.99) | 0.27 (0.18, 0.38) |  | 120.74 | -0.22 (-0.41, -0.03) |
| Greece | 19.26 (13.81, 26.41) | 0.15 (0.11, 0.19) |  | 38.18 (23.70, 57.07) | 0.20 (0.13, 0.28) |  | 98.26 | 1.27 (1.16, 1.38) |
| Greenland | 0.17 (0.13, 0.24) | 0.42 (0.30, 0.56) |  | 0.35 (0.22, 0.55) | 0.51 (0.33, 0.75) |  | 102.36 | 0.44 (0.27, 0.60) |
| Grenada | 0.45 (0.33, 0.61) | 0.62 (0.44, 0.83) |  | 0.20 (0.14, 0.28) | 0.19 (0.13, 0.25) |  | -55.79 | -4.25 (-5.22, -3.27) |
| Guam | 0.15 (0.10, 0.20) | 0.16 (0.11, 0.22) |  | 0.38 (0.25, 0.54) | 0.20 (0.14, 0.29) |  | 157.35 | 1.09 (0.97, 1.21) |
| Guatemala | 32.43 (24.66, 41.51) | 0.64 (0.47, 0.86) |  | 41.13 (28.84, 56.18) | 0.31 (0.21, 0.43) |  | 26.82 | -3.02 (-4.41, -1.60) |
| Guinea | 72.08 (50.42, 102.28) | 1.42 (1.01, 1.99) |  | 121.95 (78.69, 178.10) | 1.52 (0.99, 2.27) |  | 69.19 | 0.30 (0.26, 0.34) |
| Guinea-Bissau | 2.42 (1.52, 3.71) | 0.39 (0.24, 0.59) |  | 3.28 (2.14, 4.97) | 0.33 (0.20, 0.52) |  | 35.70 | -0.71 (-0.77, -0.65) |
| Guyana | 2.15 (1.59, 2.83) | 0.45 (0.32, 0.61) |  | 1.41 (0.93, 2.01) | 0.21 (0.14, 0.30) |  | -34.66 | -2.77 (-3.53, -1.99) |
| Haiti | 21.14 (11.84, 34.97) | 0.50 (0.28, 0.80) |  | 25.86 (15.07, 40.24) | 0.30 (0.17, 0.47) |  | 22.29 | -1.72 (-1.97, -1.48) |
| Honduras | 19.06 (9.22, 29.12) | 0.69 (0.28, 1.15) |  | 53.71 (22.81, 90.86) | 0.82 (0.33, 1.39) |  | 181.73 | 0.68 (0.57, 0.78) |
| Hungary | 30.58 (21.14, 41.64) | 0.23 (0.17, 0.31) |  | 14.90 (9.84, 21.56) | 0.10 (0.07, 0.14) |  | -51.27 | -2.19 (-2.75, -1.64) |
| Iceland | 0.40 (0.30, 0.53) | 0.15 (0.12, 0.20) |  | 0.96 (0.67, 1.34) | 0.21 (0.15, 0.28) |  | 141.72 | 1.04 (0.88, 1.19) |
| India | 857.34 (703.81, 1086.98) | 0.13 (0.10, 0.15) |  | 1591.97 (1284.32, 1997.45) | 0.13 (0.10, 0.16) |  | 85.69 | 0.04 (-0.04, 0.11) |
| Indonesia | 232.81 (179.16, 310.88) | 0.14 (0.11, 0.18) |  | 220.21 (179.30, 267.46) | 0.10 (0.08, 0.12) |  | -5.41 | -1.34 (-1.44, -1.25) |
| Iran | 112.09 (92.42, 135.17) | 0.35 (0.28, 0.44) |  | 217.55 (186.12, 253.10) | 0.30 (0.25, 0.35) |  | 94.09 | -0.91 (-1.58, -0.24) |
| Iraq | 33.97 (24.19, 45.17) | 0.31 (0.21, 0.43) |  | 110.55 (75.05, 160.90) | 0.41 (0.27, 0.60) |  | 225.46 | 1.23 (0.87, 1.59) |
| Ireland | 4.25 (3.23, 5.53) | 0.11 (0.08, 0.14) |  | 15.44 (10.23, 22.31) | 0.24 (0.16, 0.34) |  | 263.36 | 3.37 (3.09, 3.64) |
| Israel | 8.86 (6.35, 12.18) | 0.18 (0.13, 0.24) |  | 22.12 (14.79, 31.91) | 0.21 (0.14, 0.30) |  | 149.67 | 0.46 (0.35, 0.56) |
| Italy | 169.94 (144.96, 198.34) | 0.23 (0.20, 0.27) |  | 209.68 (164.29, 262.94) | 0.22 (0.17, 0.27) |  | 23.38 | -0.51 (-0.66, -0.35) |
| Jamaica | 5.24 (3.91, 6.93) | 0.27 (0.20, 0.37) |  | 5.25 (3.57, 7.41) | 0.18 (0.12, 0.26) |  | 0.35 | -0.40 (-1.31, 0.51) |
| Japan | 761.13 (651.84, 879.09) | 0.47 (0.41, 0.54) |  | 1236.37 (937.41, 1548.68) | 0.42 (0.34, 0.50) |  | 62.44 | -1.25 (-1.69, -0.79) |
| Jordan | 4.04 (3.00, 5.35) | 0.21 (0.14, 0.30) |  | 13.36 (9.61, 18.22) | 0.17 (0.12, 0.24) |  | 230.93 | -1.00 (-1.19, -0.81) |
| Kazakhstan | 28.09 (18.34, 40.75) | 0.18 (0.12, 0.26) |  | 43.82 (29.81, 60.03) | 0.25 (0.17, 0.34) |  | 56.02 | -1.50 (-2.34, -0.64) |
| Kenya | 28.37 (21.42, 43.87) | 0.22 (0.16, 0.36) |  | 67.27 (45.59, 97.61) | 0.24 (0.16, 0.35) |  | 137.14 | -0.25 (-0.58, 0.08) |
| Kiribati | 0.32 (0.22, 0.44) | 0.66 (0.45, 0.93) |  | 0.53 (0.36, 0.78) | 0.60 (0.40, 0.89) |  | 66.80 | -0.30 (-0.35, -0.25) |
| Kuwait | 1.80 (1.39, 2.27) | 0.18 (0.14, 0.25) |  | 5.00 (3.60, 6.73) | 0.18 (0.13, 0.25) |  | 177.58 | 0.53 (0.31, 0.76) |
| Kyrgyzstan | 5.08 (2.66, 8.66) | 0.11 (0.06, 0.17) |  | 6.49 (4.58, 9.09) | 0.12 (0.08, 0.16) |  | 27.84 | 0.76 (0.46, 1.06) |
| Lao | 8.64 (4.92, 14.45) | 0.31 (0.18, 0.50) |  | 10.78 (6.98, 15.61) | 0.21 (0.13, 0.30) |  | 24.75 | -1.69 (-1.79, -1.58) |
| Latvia | 3.33 (2.13, 4.76) | 0.12 (0.08, 0.19) |  | 3.38 (2.27, 4.86) | 0.12 (0.08, 0.16) |  | 1.64 | -0.43 (-0.76, -0.10) |
| Lebanon | 4.64 (3.21, 6.50) | 0.18 (0.12, 0.25) |  | 10.95 (7.30, 16.66) | 0.21 (0.14, 0.32) |  | 136.24 | 0.73 (0.56, 0.90) |
| Lesotho | 2.90 (1.69, 5.27) | 0.25 (0.14, 0.47) |  | 8.81 (3.72, 15.19) | 0.60 (0.26, 1.04) |  | 203.75 | 3.55 (3.05, 4.05) |
| Liberia | 4.82 (3.21, 7.10) | 0.32 (0.22, 0.45) |  | 6.60 (4.30, 9.90) | 0.24 (0.15, 0.37) |  | 37.06 | -1.13 (-1.27, -0.98) |
| Libya | 9.26 (6.30, 13.25) | 0.37 (0.25, 0.55) |  | 19.74 (12.87, 29.75) | 0.36 (0.23, 0.55) |  | 113.26 | 0.05 (-0.12, 0.22) |
| Lithuania | 4.25 (2.70, 6.02) | 0.12 (0.07, 0.17) |  | 5.22 (3.46, 7.60) | 0.12 (0.09, 0.17) |  | 22.74 | 0.01 (-0.18, 0.19) |
| Luxembourg | 0.81 (0.58, 1.11) | 0.18 (0.14, 0.23) |  | 1.94 (1.28, 2.82) | 0.24 (0.16, 0.33) |  | 139.31 | 1.10 (0.82, 1.39) |
| Macedonia | 6.75 (4.92, 9.21) | 0.35 (0.26, 0.48) |  | 9.52 (6.07, 14.71) | 0.32 (0.21, 0.47) |  | 40.99 | -0.50 (-0.63, -0.36) |
| Madagascar | 23.25 (14.48, 36.61) | 0.26 (0.17, 0.39) |  | 34.33 (22.28, 51.14) | 0.21 (0.13, 0.31) |  | 47.66 | -0.84 (-0.92, -0.75) |
| Malawi | 46.62 (26.65, 75.64) | 0.43 (0.29, 0.62) |  | 59.77 (40.41, 87.16) | 0.34 (0.24, 0.47) |  | 28.20 | -1.23 (-1.44, -1.01) |
| Malaysia | 16.29 (11.65, 21.95) | 0.15 (0.10, 0.21) |  | 41.56 (26.95, 60.86) | 0.15 (0.10, 0.22) |  | 155.03 | 0.18 (-0.04, 0.39) |
| Maldives | 0.23 (0.14, 0.39) | 0.19 (0.11, 0.33) |  | 0.51 (0.35, 0.73) | 0.15 (0.10, 0.22) |  | 124.62 | -0.67 (-0.83, -0.51) |
| Mali | 57.96 (38.35, 85.24) | 0.73 (0.51, 1.06) |  | 86.47 (57.56, 124.96) | 0.65 (0.42, 0.95) |  | 49.19 | -0.72 (-0.96, -0.49) |
| Malta | 0.47 (0.35, 0.63) | 0.12 (0.09, 0.16) |  | 1.13 (0.77, 1.65) | 0.18 (0.13, 0.24) |  | 138.64 | 1.28 (1.10, 1.45) |
| Marshall Islands | 0.11 (0.07, 0.15) | 0.51 (0.32, 0.75) |  | 0.19 (0.11, 0.28) | 0.44 (0.27, 0.67) |  | 71.23 | -0.54 (-0.61, -0.48) |
| Mauritania | 4.38 (3.06, 5.92) | 0.34 (0.22, 0.49) |  | 5.51 (3.47, 8.08) | 0.22 (0.14, 0.33) |  | 25.61 | -1.47 (-1.62, -1.32) |
| Mauritius | 0.62 (0.46, 0.81) | 0.07 (0.05, 0.09) |  | 1.01 (0.65, 1.45) | 0.07 (0.05, 0.09) |  | 62.87 | 0.75 (0.43, 1.08) |
| Mexico | 116.74 (104.23, 133.79) | 0.18 (0.16, 0.20) |  | 271.43 (224.84, 322.08) | 0.23 (0.19, 0.27) |  | 132.50 | 1.07 (0.92, 1.23) |
| Moldova | 4.21 (2.46, 6.59) | 0.10 (0.06, 0.15) |  | 4.01 (2.81, 5.52) | 0.09 (0.07, 0.12) |  | -4.66 | -1.36 (-2.51, -0.20) |
| Monaco | 0.11 (0.07, 0.16) | 0.23 (0.16, 0.31) |  | 0.37 (0.23, 0.55) | 0.50 (0.34, 0.71) |  | 232.39 | 3.31 (2.58, 4.05) |
| Mongolia | 22.74 (14.96, 34.15) | 2.03 (1.31, 3.09) |  | 78.92 (50.62, 120.85) | 3.47 (2.19, 5.27) |  | 247.09 | 2.60 (2.04, 3.17) |
| Montenegro | 1.49 (1.07, 2.08) | 0.24 (0.17, 0.33) |  | 1.93 (1.29, 2.79) | 0.22 (0.15, 0.32) |  | 29.56 | -0.37 (-0.61, -0.12) |
| Morocco | 18.69 (12.93, 25.76) | 0.12 (0.08, 0.17) |  | 40.32 (26.22, 59.48) | 0.13 (0.09, 0.19) |  | 115.78 | 0.21 (0.04, 0.38) |
| Mozambique | 19.53 (11.60, 31.65) | 0.18 (0.11, 0.27) |  | 61.86 (37.88, 96.72) | 0.28 (0.19, 0.41) |  | 216.70 | 1.72 (1.59, 1.85) |
| Myanmar | 48.78 (29.48, 84.31) | 0.13 (0.09, 0.22) |  | 102.56 (68.98, 150.80) | 0.21 (0.14, 0.30) |  | 110.24 | 1.31 (1.08, 1.53) |
| Namibia | 0.95 (0.58, 1.58) | 0.10 (0.06, 0.18) |  | 2.84 (1.88, 4.24) | 0.16 (0.11, 0.24) |  | 197.50 | 1.68 (1.44, 1.92) |
| Nauru | 0.03 (0.02, 0.04) | 0.47 (0.30, 0.71) |  | 0.03 (0.02, 0.04) | 0.43 (0.26, 0.69) |  | 2.85 | -0.44 (-0.61, -0.27) |
| Nepal | 25.60 (15.59, 43.97) | 0.12 (0.08, 0.18) |  | 31.64 (21.58, 46.91) | 0.12 (0.08, 0.17) |  | 23.60 | 0.18 (0.07, 0.30) |
| Netherlands | 19.58 (15.00, 25.40) | 0.13 (0.10, 0.16) |  | 58.88 (38.31, 84.42) | 0.24 (0.17, 0.32) |  | 200.63 | 2.50 (2.35, 2.66) |
| New Zealand | 5.74 (5.04, 6.49) | 0.16 (0.14, 0.18) |  | 18.13 (14.50, 22.24) | 0.29 (0.24, 0.35) |  | 215.79 | 2.20 (2.05, 2.36) |
| Nicaragua | 5.67 (4.55, 6.94) | 0.21 (0.16, 0.28) |  | 11.52 (8.37, 15.62) | 0.23 (0.16, 0.31) |  | 103.20 | 0.40 (-0.09, 0.89) |
| Niger | 1.47 (1.07, 1.95) | 0.03 (0.02, 0.04) |  | 4.46 (3.01, 6.45) | 0.03 (0.02, 0.05) |  | 203.63 | -0.08 (-0.18, 0.02) |
| Nigeria | 197.14 (151.06, 248.87) | 0.23 (0.18, 0.29) |  | 265.18 (173.79, 363.37) | 0.19 (0.14, 0.25) |  | 34.52 | -0.39 (-0.73, -0.05) |
| Niue | 0.01 (0.01, 0.01) | 0.37 (0.24, 0.53) |  | 0.01 (0.00, 0.01) | 0.32 (0.20, 0.48) |  | -18.54 | -0.51 (-0.56, -0.46) |
| North Korea | 172.91 (110.46, 251.52) | 0.92 (0.60, 1.33) |  | 192.80 (118.76, 289.86) | 0.60 (0.38, 0.88) |  | 11.51 | -1.70 (-1.84, -1.56) |
| Northern Mariana Islands | 0.08 (0.05, 0.11) | 0.30 (0.20, 0.43) |  | 0.15 (0.09, 0.23) | 0.28 (0.19, 0.40) |  | 97.39 | -0.16 (-0.27, -0.06) |
| Norway | 6.77 (5.95, 7.69) | 0.13 (0.12, 0.15) |  | 14.70 (11.90, 18.08) | 0.20 (0.17, 0.25) |  | 117.33 | 1.81 (1.67, 1.94) |
| Oman | 3.06 (2.14, 4.20) | 0.27 (0.18, 0.39) |  | 8.97 (6.72, 11.97) | 0.37 (0.27, 0.51) |  | 192.70 | 1.79 (1.42, 2.16) |
| Pakistan | 164.83 (125.01, 215.62) | 0.16 (0.12, 0.21) |  | 355.02 (272.06, 467.54) | 0.18 (0.14, 0.23) |  | 115.39 | 0.37 (0.28, 0.45) |
| Palau | 0.04 (0.02, 0.06) | 0.33 (0.20, 0.51) |  | 0.07 (0.04, 0.12) | 0.32 (0.21, 0.49) |  | 100.68 | 0.12 (0.02, 0.22) |
| Palestine | 5.98 (3.88, 8.81) | 0.56 (0.35, 0.86) |  | 11.48 (8.28, 15.59) | 0.41 (0.29, 0.58) |  | 91.86 | -1.03 (-1.09, -0.97) |
| Panama | 3.99 (3.08, 5.09) | 0.21 (0.16, 0.29) |  | 7.49 (5.11, 10.36) | 0.18 (0.12, 0.25) |  | 87.76 | -0.28 (-0.69, 0.13) |
| Papua New Guinea | 1.91 (1.35, 2.63) | 0.07 (0.05, 0.09) |  | 4.82 (3.43, 6.70) | 0.07 (0.05, 0.10) |  | 151.82 | 0.39 (0.35, 0.43) |
| Paraguay | 4.43 (3.37, 5.93) | 0.14 (0.10, 0.19) |  | 6.69 (4.34, 9.97) | 0.11 (0.07, 0.16) |  | 50.79 | -0.72 (-1.17, -0.26) |
| Peru | 62.90 (46.78, 81.08) | 0.42 (0.30, 0.58) |  | 63.90 (42.44, 91.00) | 0.20 (0.13, 0.28) |  | 1.60 | -3.40 (-3.88, -2.91) |
| Philippines | 119.98 (97.50, 146.52) | 0.29 (0.23, 0.37) |  | 186.56 (146.86, 235.28) | 0.20 (0.16, 0.25) |  | 55.49 | -1.57 (-1.82, -1.32) |
| Poland | 120.01 (102.89, 137.52) | 0.29 (0.25, 0.33) |  | 44.08 (35.70, 53.53) | 0.08 (0.07, 0.10) |  | -63.27 | -3.95 (-5.08, -2.80) |
| Portugal | 15.04 (11.30, 19.69) | 0.13 (0.10, 0.17) |  | 46.18 (29.16, 69.67) | 0.26 (0.18, 0.37) |  | 206.96 | 2.22 (1.98, 2.45) |
| Puerto Rico | 13.90 (9.83, 18.95) | 0.38 (0.27, 0.52) |  | 9.63 (6.10, 14.13) | 0.16 (0.11, 0.23) |  | -30.70 | -1.92 (-2.54, -1.30) |
| Qatar | 0.94 (0.63, 1.36) | 0.84 (0.52, 1.27) |  | 6.61 (4.16, 10.21) | 0.85 (0.50, 1.35) |  | 600.01 | 0.71 (0.53, 0.90) |
| Romania | 20.32 (15.09, 26.58) | 0.08 (0.06, 0.10) |  | 32.88 (21.77, 47.11) | 0.12 (0.09, 0.16) |  | 61.84 | 1.91 (1.60, 2.21) |
| Russia | 218.79 (181.30, 277.15) | 0.15 (0.12, 0.20) |  | 292.61 (220.24, 381.98) | 0.17 (0.12, 0.25) |  | 33.74 | 0.68 (0.36, 1.00) |
| Rwanda | 19.10 (11.52, 31.37) | 0.39 (0.24, 0.60) |  | 28.59 (18.81, 42.66) | 0.34 (0.23, 0.48) |  | 49.70 | -0.97 (-1.14, -0.81) |
| Saint Kitts and Nevis | 0.32 (0.23, 0.44) | 0.86 (0.62, 1.18) |  | 0.14 (0.09, 0.21) | 0.22 (0.15, 0.32) |  | -55.78 | -4.59 (-5.65, -3.53) |
| Saint Lucia | 0.39 (0.29, 0.51) | 0.40 (0.29, 0.54) |  | 0.26 (0.18, 0.36) | 0.13 (0.09, 0.18) |  | -33.47 | -4.06 (-5.06, -3.06) |
| Saint Vincent and the Grenadines | 0.39 (0.30, 0.50) | 0.48 (0.36, 0.63) |  | 0.23 (0.16, 0.31) | 0.18 (0.12, 0.24) |  | -42.00 | -3.53 (-4.36, -2.70) |
| Samoa | 0.27 (0.18, 0.37) | 0.24 (0.16, 0.35) |  | 0.34 (0.22, 0.49) | 0.20 (0.13, 0.30) |  | 28.33 | -0.62 (-0.71, -0.54) |
| San Marino | 0.05 (0.03, 0.06) | 0.19 (0.14, 0.24) |  | 0.10 (0.07, 0.15) | 0.25 (0.17, 0.34) |  | 123.91 | 1.40 (1.28, 1.53) |
| Sao Tome and Principe | 0.11 (0.08, 0.15) | 0.12 (0.09, 0.17) |  | 0.18 (0.13, 0.26) | 0.13 (0.09, 0.19) |  | 66.04 | 0.24 (0.09, 0.39) |
| Saudi Arabia | 18.24 (12.53, 25.63) | 0.24 (0.15, 0.35) |  | 50.66 (32.22, 77.05) | 0.26 (0.16, 0.39) |  | 177.67 | 0.12 (-0.05, 0.29) |
| Senegal | 7.55 (5.27, 10.80) | 0.15 (0.10, 0.20) |  | 14.57 (9.59, 20.79) | 0.15 (0.10, 0.22) |  | 92.93 | 0.26 (0.07, 0.46) |
| Serbia | 21.74 (15.08, 31.49) | 0.20 (0.15, 0.28) |  | 23.59 (14.67, 35.60) | 0.17 (0.11, 0.24) |  | 8.50 | -0.97 (-1.19, -0.76) |
| Seychelles | 0.13 (0.09, 0.18) | 0.22 (0.15, 0.32) |  | 0.17 (0.11, 0.25) | 0.16 (0.11, 0.22) |  | 30.32 | -0.93 (-1.23, -0.62) |
| Sierra Leone | 8.11 (5.37, 12.21) | 0.28 (0.19, 0.41) |  | 13.75 (8.85, 20.15) | 0.26 (0.17, 0.39) |  | 69.58 | -0.35 (-0.46, -0.24) |
| Singapore | 5.72 (4.03, 7.79) | 0.25 (0.18, 0.35) |  | 24.14 (14.53, 36.82) | 0.34 (0.22, 0.51) |  | 321.99 | 1.08 (0.85, 1.32) |
| Slovakia | 9.69 (7.04, 13.01) | 0.17 (0.13, 0.22) |  | 9.95 (6.33, 14.55) | 0.13 (0.09, 0.19) |  | 2.67 | -1.31 (-1.57, -1.05) |
| Slovenia | 2.65 (1.70, 3.89) | 0.12 (0.08, 0.17) |  | 6.80 (4.19, 10.20) | 0.18 (0.12, 0.27) |  | 156.31 | 1.89 (1.64, 2.14) |
| Solomon Islands | 1.03 (0.70, 1.49) | 0.41 (0.27, 0.58) |  | 1.88 (1.28, 2.56) | 0.36 (0.25, 0.49) |  | 82.69 | -0.43 (-0.55, -0.31) |
| Somalia | 12.97 (6.95, 21.66) | 0.29 (0.16, 0.48) |  | 32.78 (19.18, 55.23) | 0.27 (0.16, 0.46) |  | 152.70 | -0.03 (-0.09, 0.03) |
| South Africa | 86.81 (61.80, 134.33) | 0.33 (0.22, 0.51) |  | 134.03 (111.85, 159.43) | 0.27 (0.23, 0.32) |  | 54.40 | -0.89 (-1.46, -0.30) |
| South Korea | 122.49 (81.92, 174.00) | 0.38 (0.25, 0.54) |  | 647.35 (422.54, 944.99) | 0.76 (0.52, 1.09) |  | 428.47 | 3.07 (1.87, 4.28) |
| South Sudan | 10.43 (6.13, 16.40) | 0.25 (0.15, 0.41) |  | 13.13 (8.10, 20.22) | 0.21 (0.13, 0.34) |  | 25.90 | -0.51 (-0.60, -0.42) |
| Spain | 81.50 (58.63, 111.67) | 0.17 (0.13, 0.23) |  | 183.57 (115.57, 270.27) | 0.25 (0.17, 0.37) |  | 125.26 | 1.29 (0.97, 1.61) |
| Sri Lanka | 11.23 (7.94, 18.35) | 0.08 (0.06, 0.12) |  | 25.10 (15.37, 38.11) | 0.11 (0.07, 0.16) |  | 123.47 | 1.49 (1.27, 1.71) |
| Sudan | 34.54 (19.82, 50.99) | 0.29 (0.16, 0.43) |  | 68.17 (41.79, 104.49) | 0.30 (0.17, 0.48) |  | 97.36 | 0.23 (0.11, 0.35) |
| Suriname | 1.31 (0.96, 1.72) | 0.44 (0.32, 0.59) |  | 1.01 (0.69, 1.44) | 0.17 (0.12, 0.24) |  | -22.37 | -3.06 (-3.81, -2.29) |
| Sweden | 23.38 (19.83, 27.29) | 0.20 (0.18, 0.23) |  | 34.86 (27.42, 43.05) | 0.22 (0.18, 0.27) |  | 49.09 | 0.78 (0.31, 1.26) |
| Switzerland | 12.37 (9.03, 16.94) | 0.15 (0.11, 0.20) |  | 41.10 (25.15, 62.19) | 0.30 (0.19, 0.42) |  | 232.19 | 1.78 (1.24, 2.33) |
| Syrian Arab Republic | 23.77 (17.02, 32.97) | 0.34 (0.23, 0.50) |  | 42.07 (27.55, 62.22) | 0.33 (0.22, 0.48) |  | 76.97 | -0.08 (-0.23, 0.06) |
| Taiwan (Province of China) | 107.84 (78.53, 146.34) | 0.60 (0.44, 0.83) |  | 159.17 (101.52, 240.91) | 0.46 (0.30, 0.66) |  | 47.59 | -1.93 (-3.06, -0.78) |
| Tajikistan | 8.06 (6.12, 11.65) | 0.12 (0.09, 0.17) |  | 12.67 (8.93, 20.27) | 0.17 (0.12, 0.26) |  | 57.22 | 0.79 (0.43, 1.15) |
| Tanzania | 55.53 (33.79, 88.87) | 0.21 (0.15, 0.29) |  | 103.66 (63.30, 171.36) | 0.21 (0.15, 0.30) |  | 86.66 | 0.13 (-0.00, 0.26) |
| Thailand | 248.59 (171.19, 339.24) | 0.62 (0.41, 0.86) |  | 650.95 (395.33, 1005.58) | 0.65 (0.40, 0.98) |  | 161.86 | 0.27 (0.16, 0.39) |
| Timor-Leste | 1.07 (0.61, 1.72) | 0.24 (0.15, 0.38) |  | 1.98 (1.19, 2.90) | 0.21 (0.13, 0.31) |  | 85.48 | -0.85 (-1.08, -0.62) |
| Togo | 6.56 (4.72, 9.06) | 0.32 (0.22, 0.44) |  | 12.56 (8.45, 17.77) | 0.27 (0.18, 0.38) |  | 91.30 | -1.02 (-1.15, -0.90) |
| Tokelau | 0.01 (0.00, 0.01) | 0.41 (0.26, 0.63) |  | 0.00 (0.00, 0.01) | 0.32 (0.20, 0.48) |  | -23.58 | -0.97 (-1.04, -0.90) |
| Tonga | 0.57 (0.36, 0.85) | 0.91 (0.56, 1.37) |  | 0.78 (0.48, 1.19) | 0.94 (0.58, 1.45) |  | 37.68 | 0.14 (-0.00, 0.28) |
| Trinidad and Tobago | 4.22 (3.15, 5.53) | 0.46 (0.34, 0.61) |  | 2.84 (1.81, 4.23) | 0.17 (0.11, 0.24) |  | -32.63 | -3.62 (-4.62, -2.60) |
| Tunisia | 6.44 (4.53, 8.86) | 0.10 (0.07, 0.14) |  | 11.78 (7.48, 18.09) | 0.10 (0.06, 0.15) |  | 83.08 | 0.13 (0.05, 0.22) |
| Turkey | 100.67 (71.18, 139.33) | 0.24 (0.16, 0.34) |  | 173.46 (118.35, 243.69) | 0.21 (0.15, 0.29) |  | 72.30 | -0.35 (-0.69, 0.00) |
| Turkmenistan | 5.95 (4.54, 8.78) | 0.14 (0.11, 0.20) |  | 14.91 (10.94, 20.00) | 0.31 (0.23, 0.42) |  | 150.76 | 3.63 (3.19, 4.07) |
| Tuvalu | 0.04 (0.02, 0.05) | 0.48 (0.31, 0.72) |  | 0.04 (0.02, 0.06) | 0.37 (0.23, 0.56) |  | 8.56 | -1.01 (-1.13, -0.89) |
| Uganda | 36.26 (24.19, 51.41) | 0.30 (0.21, 0.43) |  | 113.25 (75.56, 164.93) | 0.45 (0.31, 0.65) |  | 212.28 | 1.55 (1.35, 1.75) |
| Ukraine | 62.16 (45.24, 84.34) | 0.13 (0.09, 0.18) |  | 87.40 (65.00, 115.15) | 0.19 (0.13, 0.28) |  | 40.61 | 1.76 (1.53, 1.98) |
| United Arab Emirates | 1.70 (1.07, 2.66) | 0.24 (0.13, 0.48) |  | 13.10 (5.66, 30.41) | 0.24 (0.10, 0.55) |  | 669.40 | -0.18 (-0.50, 0.15) |
| United Kingdom | 105.24 (91.78, 119.20) | 0.15 (0.13, 0.17) |  | 307.96 (248.01, 379.43) | 0.31 (0.25, 0.37) |  | 192.63 | 3.17 (2.96, 3.37) |
| United States of America | 741.23 (656.83, 824.81) | 0.26 (0.23, 0.29) |  | 2325.10 (1857.27, 2895.98) | 0.49 (0.39, 0.60) |  | 213.68 | 2.10 (1.89, 2.30) |
| United States Virgin Islands | 0.25 (0.18, 0.34) | 0.28 (0.19, 0.38) |  | 0.24 (0.16, 0.34) | 0.14 (0.10, 0.20) |  | -6.06 | -2.50 (-2.94, -2.05) |
| Uruguay | 3.66 (2.64, 5.00) | 0.10 (0.08, 0.14) |  | 6.76 (4.41, 9.98) | 0.14 (0.10, 0.21) |  | 84.57 | 1.55 (1.40, 1.69) |
| Uzbekistan | 23.68 (13.63, 39.18) | 0.10 (0.06, 0.16) |  | 73.53 (49.56, 103.07) | 0.29 (0.20, 0.39) |  | 210.52 | 5.28 (4.60, 5.97) |
| Vanuatu | 0.30 (0.18, 0.47) | 0.37 (0.21, 0.60) |  | 0.76 (0.46, 1.16) | 0.38 (0.22, 0.58) |  | 152.57 | 0.03 (-0.04, 0.10) |
| Venezuela | 43.24 (33.25, 55.74) | 0.36 (0.26, 0.49) |  | 36.78 (24.42, 53.28) | 0.13 (0.09, 0.19) |  | -14.94 | -2.80 (-3.88, -1.72) |
| Viet Nam | 59.48 (41.40, 84.95) | 0.13 (0.08, 0.19) |  | 83.00 (55.46, 122.50) | 0.10 (0.07, 0.14) |  | 39.54 | -1.18 (-1.38, -0.98) |
| Yemen | 15.84 (9.12, 25.88) | 0.23 (0.13, 0.36) |  | 40.85 (26.81, 58.64) | 0.24 (0.15, 0.35) |  | 157.90 | 0.32 (0.23, 0.41) |
| Zambia | 14.66 (8.41, 24.52) | 0.22 (0.14, 0.32) |  | 26.33 (17.65, 37.42) | 0.22 (0.15, 0.31) |  | 79.64 | -0.45 (-0.70, -0.20) |
| Zimbabwe | 28.10 (18.76, 42.35) | 0.53 (0.35, 0.83) |  | 65.32 (42.82, 95.69) | 0.72 (0.46, 1.10) |  | 132.47 | 0.33 (-0.11, 0.76) |

ASIR: age-standardized incidence rate; CI: confidence interval; EAPC: estimated annual percentage change; UI: uncertainty interval.


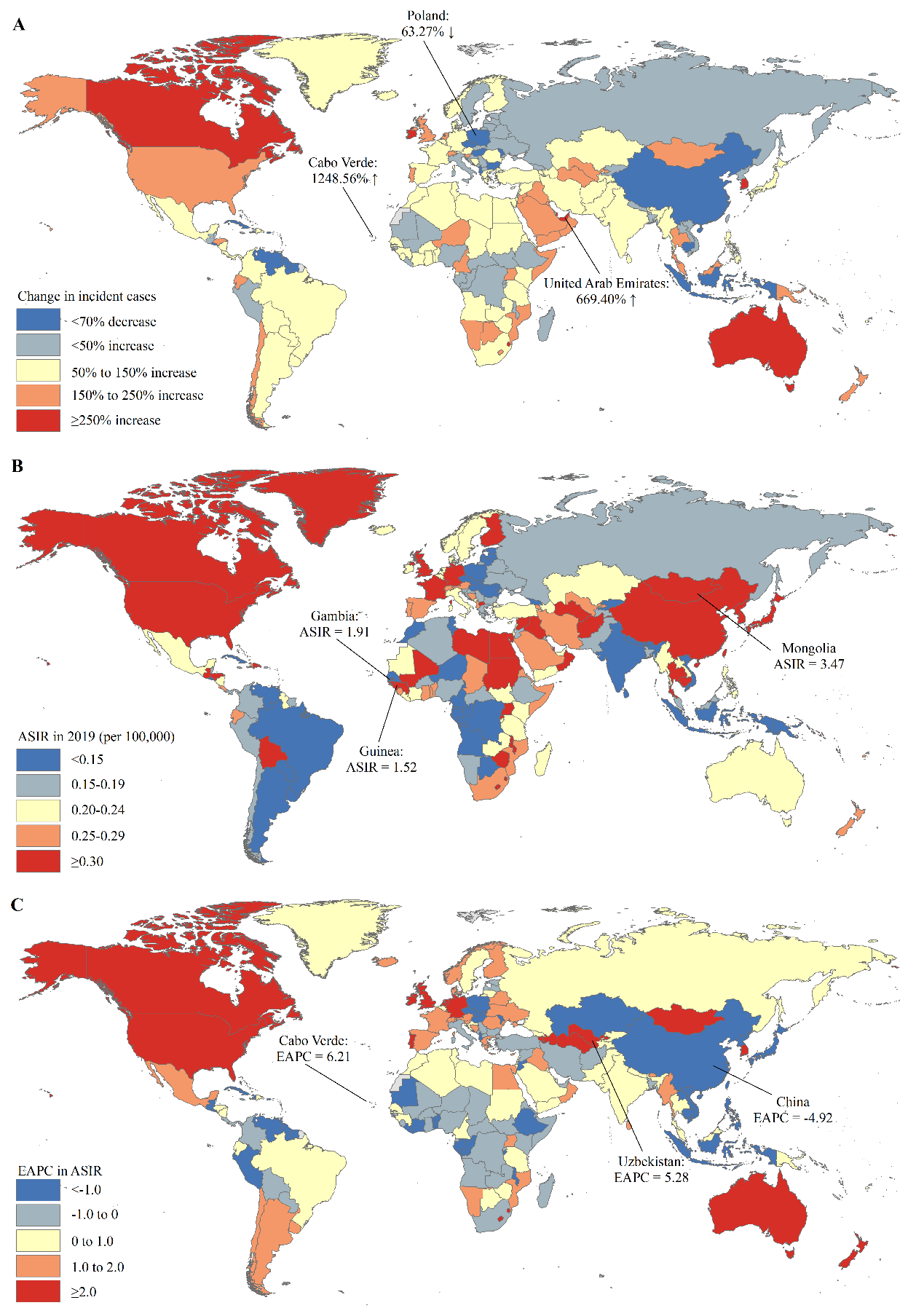


**Figure S5.** The global trends in the incidence of primary liver cancer due to other causes in 204 countries and territories. (A) The percentage change in incident cases of primary liver cancer due to other causes between 1990 and 2019; (B) The ASIR of primary liver cancer due to other causes in 2019; (C) The EAPCs in ASIR of primary liver cancer due to other causes from 1990 to 2019.

Note: ASIR: age-standardized incidence rate; EAPC: estimated annual percentage change.

**Table S18. The deaths and ASMR of primary liver cancer in 1990 and 2019 and their change trends from 1990 to 2019 at national level.**

| Nation | 1990 | |  | 2019 | |  | 1990-2019 | |
| --- | --- | --- | --- | --- | --- | --- | --- | --- |
|  | Deaths  No. (95% UI) | ASMR per 100,000  No. (95% UI) |  | Deaths  No. x 10^3^ (95% UI) | ASMR per 100,000  No. (95% UI) |  | Percentage change in deaths  No. (%) | EAPC in ASMR  No. (95% CI) |
| Afghanistan | 851.07 (656.87, 1089.35) | 11.97 (9.38, 15.15) |  | 1376.25 (1041.25, 1789.35) | 10.27 (7.98, 12.93) |  | 61.71 | -0.58 (-0.68, -0.49) |
| Albania | 232.98 (216.67, 248.76) | 11.62 (10.81, 12.44) |  | 294.16 (215.14, 392.47) | 6.84 (5.04, 9.09) |  | 26.26 | -2.85 (-3.42, -2.28) |
| Algeria | 209.01 (169.71, 258.16) | 1.81 (1.48, 2.20) |  | 687.88 (533.44, 873.82) | 2.20 (1.72, 2.78) |  | 229.12 | 0.69 (0.53, 0.86) |
| American Samoa | 1.33 (1.01, 1.63) | 5.69 (4.37, 6.94) |  | 3.39 (2.80, 4.13) | 7.02 (5.85, 8.45) |  | 155.97 | 1.11 (0.88, 1.33) |
| Andorra | 5.01 (3.73, 6.84) | 9.38 (7.05, 12.68) |  | 13.26 (10.06, 17.08) | 9.44 (7.14, 12.24) |  | 164.74 | 0.06 (-0.03, 0.15) |
| Angola | 116.85 (90.60, 150.48) | 2.75 (2.15, 3.43) |  | 301.62 (232.19, 385.33) | 2.59 (2.09, 3.23) |  | 158.12 | -0.35 (-0.43, -0.26) |
| Antigua and Barbuda | 4.37 (3.97, 4.81) | 8.11 (7.35, 8.96) |  | 2.78 (2.36, 3.24) | 2.85 (2.44, 3.31) |  | -36.24 | -3.83 (-4.85, -2.81) |
| Argentina | 480.95 (419.24, 545.17) | 1.51 (1.32, 1.70) |  | 1172.17 (1088.88, 1267.64) | 2.15 (2.00, 2.33) |  | 143.72 | 1.98 (1.74, 2.22) |
| Armenia | 29.32 (27.53, 31.40) | 1.13 (1.06, 1.21) |  | 289.45 (239.93, 342.82) | 7.07 (5.88, 8.38) |  | 887.16 | 9.56 (7.95, 11.20) |
| Australia | 382.15 (365.41, 398.61) | 1.96 (1.87, 2.04) |  | 1725.71 (1566.23, 1885.59) | 4.20 (3.83, 4.59) |  | 351.58 | 3.01 (2.78, 3.24) |
| Austria | 387.22 (368.02, 404.72) | 3.30 (3.14, 3.46) |  | 799.40 (714.12, 886.03) | 4.50 (4.04, 4.99) |  | 106.45 | 1.32 (1.05, 1.60) |
| Azerbaijan | 50.81 (45.90, 56.41) | 1.02 (0.92, 1.14) |  | 364.79 (280.25, 483.81) | 4.37 (3.31, 5.94) |  | 617.92 | 6.16 (4.06, 8.29) |
| Bahamas | 13.23 (11.68, 14.74) | 8.47 (7.48, 9.48) |  | 12.35 (10.18, 15.18) | 3.21 (2.66, 3.96) |  | -6.63 | -3.58 (-4.47, -2.68) |
| Bahrain | 9.00 (7.50, 10.63) | 6.11 (5.14, 7.17) |  | 38.73 (29.78, 50.01) | 5.23 (4.12, 6.53) |  | 330.28 | -1.34 (-1.77, -0.90) |
| Bangladesh | 1740.40 (1404.11, 2117.24) | 3.24 (2.68, 3.91) |  | 3521.46 (2772.27, 4396.14) | 2.75 (2.17, 3.42) |  | 102.34 | -0.94 (-1.10, -0.78) |
| Barbados | 8.60 (6.41, 11.06) | 2.91 (2.16, 3.72) |  | 13.31 (11.11, 15.86) | 2.74 (2.29, 3.27) |  | 54.89 | -0.40 (-0.64, -0.15) |
| Belarus | 166.90 (157.74, 175.94) | 1.31 (1.24, 1.38) |  | 376.87 (275.10, 504.46) | 2.41 (1.76, 3.24) |  | 125.80 | 2.96 (2.27, 3.67) |
| Belgium | 456.97 (431.79, 479.20) | 2.93 (2.78, 3.07) |  | 865.45 (786.42, 943.42) | 3.78 (3.45, 4.12) |  | 89.39 | 0.77 (0.39, 1.14) |
| Belize | 5.97 (5.44, 6.51) | 6.33 (5.76, 6.94) |  | 8.64 (7.40, 9.96) | 3.21 (2.75, 3.70) |  | 44.81 | -2.51 (-3.12, -1.90) |
| Benin | 144.17 (115.74, 182.18) | 6.96 (5.58, 8.87) |  | 255.35 (189.53, 343.46) | 5.12 (3.89, 6.79) |  | 77.12 | -1.37 (-1.53, -1.21) |
| Bermuda | 4.60 (4.13, 5.09) | 7.49 (6.74, 8.28) |  | 2.69 (2.24, 3.30) | 2.05 (1.70, 2.51) |  | -41.47 | -4.40 (-5.49, -3.29) |
| Bhutan | 5.95 (3.87, 8.87) | 2.29 (1.55, 3.33) |  | 17.83 (12.34, 25.15) | 3.27 (2.29, 4.61) |  | 199.74 | 1.34 (1.24, 1.44) |
| Bolivia | 169.13 (127.54, 221.77) | 5.29 (4.04, 6.95) |  | 427.28 (318.67, 549.84) | 5.03 (3.77, 6.43) |  | 152.64 | -0.27 (-0.36, -0.17) |
| Bosnia and Herzegovina | 233.28 (217.17, 250.89) | 5.78 (5.38, 6.20) |  | 481.25 (384.96, 600.59) | 8.01 (6.40, 10.00) |  | 106.30 | 1.58 (1.22, 1.95) |
| Botswana | 5.86 (2.85, 13.38) | 1.03 (0.50, 2.27) |  | 21.92 (15.41, 29.89) | 1.53 (1.13, 2.02) |  | 273.72 | 0.56 (-0.26, 1.38) |
| Brazil | 1830.00 (1751.11, 1894.07) | 2.09 (1.98, 2.17) |  | 5822.11 (5434.32, 6127.20) | 2.50 (2.33, 2.64) |  | 218.15 | 1.10 (0.94, 1.25) |
| Brunei | 9.39 (7.70, 11.28) | 10.13 (8.33, 12.08) |  | 34.65 (29.15, 41.13) | 11.53 (9.82, 13.38) |  | 268.89 | 1.15 (0.63, 1.67) |
| Bulgaria | 806.62 (756.83, 861.22) | 6.43 (6.04, 6.86) |  | 645.95 (516.06, 793.95) | 4.62 (3.68, 5.71) |  | -19.92 | -0.55 (-1.11, 0.00) |
| Burkina Faso | 153.38 (121.18, 188.77) | 3.25 (2.63, 3.93) |  | 248.23 (189.48, 317.03) | 2.37 (1.80, 2.96) |  | 61.84 | -1.27 (-1.47, -1.06) |
| Burundi | 97.42 (71.02, 129.07) | 3.94 (2.93, 5.15) |  | 144.30 (98.98, 224.74) | 3.11 (2.17, 4.80) |  | 48.13 | -1.35 (-1.54, -1.17) |
| Cambodia | 676.07 (523.06, 898.62) | 11.62 (9.34, 13.98) |  | 1183.92 (924.99, 1462.43) | 9.86 (7.85, 12.03) |  | 75.12 | -0.81 (-0.92, -0.70) |
| Cameroon | 30.58 (21.33, 46.20) | 0.69 (0.48, 1.04) |  | 84.73 (60.59, 115.29) | 0.69 (0.51, 0.92) |  | 177.10 | -0.19 (-0.44, 0.05) |
| Canada | 618.41 (591.19, 641.85) | 1.92 (1.83, 1.99) |  | 2667.69 (2373.58, 2951.98) | 3.91 (3.48, 4.32) |  | 331.38 | 2.84 (2.62, 3.05) |
| Cape Verde | 2.80 (2.40, 3.22) | 1.17 (1.01, 1.35) |  | 52.78 (43.70, 63.64) | 12.34 (10.20, 14.87) |  | 1786.75 | 6.41 (4.07, 8.80) |
| Central African Republic | 48.12 (33.45, 67.39) | 3.82 (2.71, 5.18) |  | 75.39 (47.93, 112.92) | 3.42 (2.27, 5.01) |  | 56.67 | -0.90 (-1.14, -0.66) |
| Chad | 190.09 (147.76, 243.07) | 6.54 (5.05, 8.35) |  | 325.01 (246.25, 421.13) | 5.68 (4.37, 7.29) |  | 70.97 | -0.64 (-0.75, -0.54) |
| Chile | 218.45 (203.69, 235.02) | 2.21 (2.06, 2.37) |  | 726.57 (664.15, 791.16) | 3.02 (2.76, 3.29) |  | 232.60 | 1.72 (1.44, 1.99) |
| China | 232449.17 (197395.48, 275388.25) | 25.99 (22.29, 30.55) |  | 187699.58 (158261.72, 222767.27) | 9.41 (7.95, 11.13) |  | -19.25 | -5.06 (-5.88, -4.24) |
| Colombia | 522.62 (494.73, 548.57) | 3.02 (2.83, 3.18) |  | 1396.49 (1071.36, 1788.80) | 2.65 (2.03, 3.40) |  | 167.21 | -0.27 (-0.74, 0.20) |
| Comoros | 7.54 (4.04, 12.58) | 3.31 (1.87, 5.51) |  | 14.45 (10.02, 22.51) | 3.01 (2.12, 4.65) |  | 91.68 | -0.50 (-0.64, -0.36) |
| Congo | 47.31 (35.05, 63.90) | 4.28 (3.25, 5.70) |  | 78.64 (56.21, 107.73) | 3.14 (2.33, 4.24) |  | 66.20 | -1.41 (-1.56, -1.26) |
| Cook Islands | 1.74 (1.44, 2.05) | 13.60 (11.34, 15.98) |  | 2.73 (2.21, 3.31) | 11.13 (9.03, 13.51) |  | 56.99 | -0.62 (-0.75, -0.50) |
| Costa Rica | 99.58 (92.00, 106.47) | 5.74 (5.29, 6.13) |  | 268.86 (208.00, 341.00) | 5.30 (4.11, 6.74) |  | 170.00 | -0.50 (-1.04, 0.05) |
| Cote d'Ivoire | 331.23 (248.63, 425.83) | 7.93 (6.08, 9.91) |  | 537.01 (388.29, 741.83) | 5.06 (3.77, 6.83) |  | 62.12 | -2.35 (-2.67, -2.02) |
| Croatia | 204.44 (188.57, 220.92) | 3.22 (2.98, 3.47) |  | 310.47 (244.57, 390.71) | 3.53 (2.77, 4.48) |  | 51.87 | 0.60 (0.03, 1.17) |
| Cuba | 695.19 (655.07, 728.15) | 6.71 (6.31, 7.03) |  | 464.16 (374.51, 573.00) | 2.43 (1.95, 3.00) |  | -33.23 | -3.79 (-5.09, -2.47) |
| Cyprus | 26.21 (21.54, 30.85) | 3.40 (2.87, 3.90) |  | 64.67 (55.61, 74.79) | 3.42 (2.95, 3.94) |  | 146.71 | 0.22 (-0.02, 0.47) |
| Czech Republic | 626.51 (594.30, 655.45) | 4.52 (4.29, 4.73) |  | 632.41 (522.22, 776.56) | 2.99 (2.45, 3.68) |  | 0.94 | -1.64 (-1.87, -1.42) |
| Democratic Republic of the Congo | 476.90 (384.55, 585.54) | 2.67 (2.20, 3.18) |  | 886.70 (675.62, 1161.28) | 2.28 (1.77, 2.94) |  | 85.93 | -0.62 (-0.69, -0.56) |
| Denmark | 155.14 (147.01, 162.71) | 1.91 (1.82, 2.01) |  | 370.21 (332.64, 407.17) | 3.25 (2.93, 3.57) |  | 138.63 | 2.19 (1.94, 2.44) |
| Djibouti | 5.14 (3.30, 9.08) | 3.37 (2.23, 5.79) |  | 20.38 (12.55, 34.07) | 3.49 (2.29, 5.59) |  | 296.77 | 0.05 (-0.08, 0.18) |
| Dominica | 6.63 (5.82, 7.45) | 9.13 (8.01, 10.31) |  | 3.05 (2.48, 3.75) | 3.39 (2.76, 4.16) |  | -54.03 | -3.56 (-4.39, -2.72) |
| Dominican Republic | 159.33 (139.45, 180.83) | 4.16 (3.61, 4.73) |  | 453.11 (309.61, 678.46) | 4.92 (3.40, 7.23) |  | 184.38 | 0.80 (0.27, 1.33) |
| Ecuador | 161.75 (144.54, 179.45) | 3.03 (2.69, 3.38) |  | 539.18 (430.22, 691.85) | 3.71 (2.97, 4.73) |  | 233.34 | 1.11 (0.77, 1.45) |
| Egypt | 5197.60 (4127.98, 6224.10) | 17.43 (13.67, 20.93) |  | 13590.24 (9715.31, 18631.61) | 21.25 (15.44, 28.92) |  | 161.47 | 1.52 (1.17, 1.88) |
| El Salvador | 106.19 (98.09, 114.40) | 3.55 (3.26, 3.84) |  | 126.80 (96.11, 164.16) | 2.12 (1.61, 2.76) |  | 19.40 | -1.98 (-2.68, -1.28) |
| Equatorial Guinea | 6.86 (5.06, 8.94) | 3.24 (2.44, 4.21) |  | 16.58 (9.57, 24.58) | 3.48 (2.04, 5.04) |  | 141.53 | 0.20 (-0.02, 0.42) |
| Eritrea | 35.70 (23.99, 55.16) | 3.40 (2.31, 5.24) |  | 87.94 (60.16, 128.54) | 3.32 (2.36, 4.73) |  | 146.33 | -0.38 (-0.56, -0.20) |
| Estonia | 43.07 (40.21, 46.09) | 2.12 (1.98, 2.27) |  | 94.76 (72.65, 118.45) | 3.61 (2.76, 4.54) |  | 120.00 | 0.98 (0.38, 1.58) |
| Eswatini | 17.86 (11.44, 34.84) | 5.95 (3.91, 11.21) |  | 117.82 (34.41, 214.87) | 19.09 (5.98, 33.88) |  | 559.55 | 5.04 (3.69, 6.41) |
| Ethiopia | 667.87 (460.89, 961.08) | 3.13 (2.24, 4.22) |  | 1226.11 (972.57, 1546.96) | 3.02 (2.40, 3.84) |  | 83.58 | -0.31 (-0.41, -0.20) |
| Federated States of Micronesia | 5.48 (4.05, 7.11) | 11.58 (8.62, 14.99) |  | 7.73 (4.98, 11.15) | 10.70 (7.24, 14.74) |  | 40.96 | -0.35 (-0.49, -0.21) |
| Fiji | 22.01 (17.75, 27.37) | 5.95 (4.79, 7.33) |  | 46.90 (35.73, 60.10) | 6.23 (4.86, 7.83) |  | 113.05 | 0.67 (0.28, 1.07) |
| Finland | 205.38 (192.50, 217.92) | 2.85 (2.67, 3.03) |  | 509.50 (462.48, 559.91) | 4.03 (3.68, 4.42) |  | 148.07 | 1.56 (1.42, 1.70) |
| France | 4009.35 (3813.28, 4199.18) | 4.94 (4.70, 5.16) |  | 7792.06 (6825.76, 8774.98) | 5.80 (5.10, 6.56) |  | 94.35 | 0.26 (-0.01, 0.53) |
| Gabon | 20.57 (15.09, 26.65) | 3.59 (2.67, 4.57) |  | 35.54 (23.61, 50.20) | 3.54 (2.33, 5.01) |  | 72.75 | -0.25 (-0.40, -0.11) |
| Gambia | 121.81 (89.60, 161.32) | 30.76 (23.19, 40.07) |  | 410.37 (295.82, 537.83) | 39.51 (29.01, 50.99) |  | 236.90 | 0.55 (0.36, 0.74) |
| Georgia | 70.58 (63.69, 78.05) | 1.20 (1.08, 1.32) |  | 210.09 (173.67, 255.19) | 3.63 (2.97, 4.42) |  | 197.69 | 3.07 (1.86, 4.30) |
| Germany | 3081.64 (2841.84, 3313.89) | 2.43 (2.24, 2.60) |  | 7743.48 (7108.84, 8362.48) | 4.02 (3.70, 4.33) |  | 151.28 | 2.03 (1.64, 2.42) |
| Ghana | 399.47 (307.10, 535.21) | 6.21 (4.81, 8.22) |  | 990.31 (733.89, 1265.52) | 6.00 (4.59, 7.59) |  | 147.90 | -0.37 (-0.56, -0.19) |
| Greece | 351.30 (329.48, 374.85) | 2.30 (2.16, 2.44) |  | 782.36 (714.07, 850.18) | 3.21 (2.95, 3.47) |  | 122.70 | 1.38 (1.23, 1.54) |
| Greenland | 1.68 (1.44, 1.97) | 4.60 (4.06, 5.31) |  | 4.43 (3.45, 5.64) | 6.35 (5.03, 7.96) |  | 164.06 | 1.15 (1.04, 1.27) |
| Grenada | 6.85 (6.15, 7.58) | 9.40 (8.39, 10.41) |  | 3.42 (3.00, 3.88) | 3.13 (2.76, 3.54) |  | -50.14 | -4.11 (-5.07, -3.13) |
| Guam | 2.97 (2.51, 3.51) | 3.91 (3.35, 4.54) |  | 11.09 (8.99, 13.63) | 5.79 (4.71, 7.05) |  | 273.38 | 1.88 (1.66, 2.10) |
| Guatemala | 352.75 (312.16, 393.84) | 10.05 (8.95, 11.17) |  | 521.27 (413.10, 653.09) | 4.81 (3.84, 6.01) |  | 47.77 | -3.46 (-4.95, -1.95) |
| Guinea | 1075.83 (894.98, 1294.66) | 31.80 (26.71, 38.10) |  | 1935.13 (1347.51, 2506.00) | 34.05 (23.98, 44.01) |  | 79.87 | 0.37 (0.31, 0.44) |
| Guinea-Bissau | 35.24 (25.21, 50.16) | 8.34 (6.09, 11.61) |  | 47.06 (33.62, 64.57) | 6.36 (4.67, 8.51) |  | 33.54 | -1.06 (-1.14, -0.98) |
| Guyana | 26.28 (22.62, 30.05) | 6.86 (5.94, 7.84) |  | 19.45 (15.07, 24.79) | 3.20 (2.53, 4.03) |  | -26.01 | -2.74 (-3.57, -1.90) |
| Haiti | 225.33 (139.45, 309.59) | 7.11 (4.41, 9.95) |  | 297.17 (180.96, 445.34) | 4.42 (2.67, 6.66) |  | 31.89 | -1.76 (-1.99, -1.53) |
| Honduras | 241.51 (93.37, 348.79) | 11.92 (4.40, 17.38) |  | 934.21 (430.78, 1372.24) | 16.14 (7.41, 23.50) |  | 286.82 | 1.35 (1.16, 1.55) |
| Hungary | 1004.72 (959.04, 1047.48) | 6.82 (6.51, 7.11) |  | 511.96 (423.39, 622.13) | 2.65 (2.18, 3.23) |  | -49.04 | -2.56 (-3.39, -1.71) |
| Iceland | 5.36 (4.90, 5.84) | 1.88 (1.72, 2.04) |  | 15.70 (13.88, 17.72) | 2.84 (2.51, 3.20) |  | 192.86 | 1.43 (1.28, 1.58) |
| India | 11859.03 (9875.95, 13742.18) | 2.70 (2.21, 3.15) |  | 30709.38 (25883.53, 36350.77) | 2.75 (2.32, 3.27) |  | 158.95 | 0.06 (-0.05, 0.17) |
| Indonesia | 2554.49 (2192.00, 2891.02) | 2.62 (2.25, 3.03) |  | 4720.82 (3974.44, 5402.93) | 2.45 (2.08, 2.76) |  | 84.81 | -0.36 (-0.45, -0.27) |
| Iran | 1075.84 (889.42, 1258.15) | 4.57 (3.70, 5.55) |  | 2501.26 (2271.82, 2749.90) | 3.64 (3.29, 4.02) |  | 132.49 | -1.21 (-1.87, -0.54) |
| Iraq | 357.99 (274.80, 444.50) | 4.58 (3.53, 5.66) |  | 1336.43 (1018.38, 1686.56) | 6.07 (4.72, 7.47) |  | 273.32 | 1.22 (0.77, 1.67) |
| Ireland | 60.67 (56.60, 65.01) | 1.47 (1.37, 1.57) |  | 257.08 (229.68, 282.57) | 3.40 (3.04, 3.75) |  | 323.75 | 3.62 (3.26, 3.97) |
| Israel | 129.60 (116.74, 143.16) | 2.65 (2.39, 2.92) |  | 324.28 (294.66, 354.23) | 2.79 (2.55, 3.04) |  | 150.21 | 0.10 (-0.00, 0.21) |
| Italy | 5829.35 (5641.88, 5959.70) | 6.50 (6.29, 6.64) |  | 6918.81 (6225.11, 7495.48) | 4.80 (4.36, 5.17) |  | 18.69 | -1.55 (-1.82, -1.27) |
| Jamaica | 74.03 (69.23, 79.33) | 4.10 (3.84, 4.39) |  | 81.55 (65.02, 101.13) | 2.75 (2.19, 3.41) |  | 10.17 | -0.73 (-1.67, 0.22) |
| Japan | 19986.95 (19306.23, 20459.88) | 11.67 (11.25, 11.96) |  | 34514.62 (29605.21, 37429.86) | 8.78 (7.80, 9.42) |  | 72.69 | -1.84 (-2.41, -1.27) |
| Jordan | 40.17 (31.94, 49.54) | 3.21 (2.52, 4.02) |  | 141.26 (113.49, 176.86) | 2.38 (1.91, 2.97) |  | 251.67 | -1.27 (-1.40, -1.13) |
| Kazakhstan | 453.43 (424.52, 482.71) | 3.57 (3.34, 3.82) |  | 1115.73 (956.32, 1290.61) | 6.54 (5.63, 7.50) |  | 146.06 | -1.17 (-2.32, -0.01) |
| Kenya | 223.58 (156.33, 392.40) | 2.65 (1.82, 4.67) |  | 724.22 (490.59, 1056.50) | 3.34 (2.28, 4.81) |  | 223.93 | 0.27 (-0.12, 0.67) |
| Kiribati | 5.73 (4.43, 7.17) | 14.29 (11.14, 17.77) |  | 8.94 (6.92, 11.40) | 11.95 (9.29, 15.08) |  | 56.16 | -0.67 (-0.72, -0.62) |
| Kuwait | 17.02 (15.18, 18.99) | 2.76 (2.45, 3.09) |  | 53.64 (41.88, 67.62) | 2.36 (1.85, 2.96) |  | 215.19 | 0.23 (-0.11, 0.57) |
| Kyrgyzstan | 40.62 (37.23, 44.14) | 1.32 (1.21, 1.42) |  | 117.42 (99.92, 135.75) | 2.70 (2.31, 3.09) |  | 189.04 | 3.34 (2.94, 3.75) |
| Lao | 222.60 (152.96, 303.47) | 10.47 (7.33, 14.06) |  | 308.46 (228.14, 398.50) | 7.11 (5.40, 9.01) |  | 38.57 | -1.61 (-1.73, -1.48) |
| Latvia | 60.19 (56.39, 64.27) | 1.70 (1.59, 1.81) |  | 106.17 (88.55, 127.06) | 2.70 (2.25, 3.29) |  | 76.39 | 0.76 (-0.04, 1.57) |
| Lebanon | 79.45 (65.05, 97.53) | 3.56 (2.95, 4.32) |  | 154.72 (114.48, 215.55) | 2.97 (2.19, 4.14) |  | 94.74 | -0.68 (-0.78, -0.59) |
| Lesotho | 53.06 (32.56, 111.50) | 5.42 (3.38, 11.16) |  | 196.83 (79.85, 311.76) | 15.17 (6.45, 23.71) |  | 270.95 | 3.99 (3.25, 4.74) |
| Liberia | 80.96 (64.02, 104.47) | 7.22 (5.74, 9.41) |  | 108.48 (77.32, 157.43) | 5.29 (3.89, 7.78) |  | 33.98 | -1.27 (-1.43, -1.11) |
| Libya | 100.71 (76.38, 130.24) | 5.37 (4.06, 6.94) |  | 255.70 (189.74, 351.18) | 5.05 (3.79, 6.86) |  | 153.89 | -0.19 (-0.32, -0.07) |
| Lithuania | 74.44 (69.75, 78.99) | 1.66 (1.56, 1.76) |  | 169.43 (134.26, 206.04) | 3.03 (2.38, 3.70) |  | 127.60 | 1.42 (1.04, 1.79) |
| Luxembourg | 16.24 (15.05, 17.58) | 2.96 (2.75, 3.20) |  | 36.32 (29.53, 45.26) | 3.62 (2.94, 4.50) |  | 123.71 | 0.76 (0.52, 1.00) |
| Macedonia | 168.37 (150.55, 184.87) | 9.13 (8.14, 10.05) |  | 280.08 (221.98, 355.01) | 8.82 (7.01, 11.10) |  | 66.34 | -0.23 (-0.36, -0.10) |
| Madagascar | 168.36 (120.60, 287.06) | 3.07 (2.12, 5.36) |  | 288.52 (195.56, 434.50) | 2.62 (1.80, 4.02) |  | 71.37 | -0.74 (-0.86, -0.62) |
| Malawi | 165.59 (122.65, 227.77) | 3.42 (2.62, 4.81) |  | 242.93 (191.47, 301.06) | 3.04 (2.43, 3.74) |  | 46.70 | -1.31 (-1.72, -0.91) |
| Malaysia | 531.73 (456.26, 622.05) | 5.78 (4.93, 6.75) |  | 1628.22 (1239.71, 2103.27) | 6.22 (4.78, 7.94) |  | 206.21 | 0.40 (0.09, 0.71) |
| Maldives | 6.07 (4.12, 9.26) | 7.33 (5.14, 10.73) |  | 16.50 (12.99, 20.25) | 5.63 (4.46, 6.89) |  | 171.65 | -0.86 (-0.99, -0.72) |
| Mali | 710.84 (585.09, 851.78) | 15.70 (12.98, 18.83) |  | 1372.14 (1010.91, 1818.36) | 15.03 (11.25, 19.52) |  | 93.03 | -0.32 (-0.42, -0.22) |
| Malta | 7.00 (6.40, 7.61) | 1.64 (1.50, 1.78) |  | 20.49 (17.79, 23.13) | 2.20 (1.93, 2.49) |  | 192.60 | 1.05 (0.80, 1.30) |
| Marshall Islands | 2.03 (1.56, 2.64) | 12.14 (9.19, 15.86) |  | 3.77 (2.67, 5.16) | 10.57 (7.64, 14.31) |  | 85.61 | -0.47 (-0.55, -0.40) |
| Mauritania | 74.52 (60.29, 90.68) | 7.31 (5.92, 8.82) |  | 90.17 (66.31, 117.12) | 4.43 (3.30, 5.68) |  | 21.01 | -1.78 (-1.92, -1.63) |
| Mauritius | 13.76 (12.74, 14.75) | 1.90 (1.75, 2.04) |  | 33.88 (26.04, 43.73) | 2.00 (1.55, 2.57) |  | 146.25 | 1.45 (1.07, 1.82) |
| Mexico | 1009.77 (971.66, 1039.02) | 2.43 (2.31, 2.50) |  | 4182.93 (3606.06, 4792.25) | 3.69 (3.18, 4.22) |  | 314.24 | 1.49 (1.32, 1.65) |
| Moldova | 74.36 (69.78, 79.23) | 1.70 (1.60, 1.81) |  | 137.92 (116.91, 161.70) | 2.40 (2.03, 2.81) |  | 85.47 | -1.07 (-2.66, 0.55) |
| Monaco | 2.27 (1.76, 2.80) | 3.24 (2.53, 4.00) |  | 7.25 (5.82, 8.79) | 7.63 (6.05, 9.39) |  | 219.82 | 3.65 (2.79, 4.51) |
| Mongolia | 692.85 (555.56, 838.04) | 66.77 (54.18, 79.90) |  | 2365.47 (1818.56, 3038.55) | 115.23 (91.48, 142.48) |  | 241.41 | 2.65 (2.16, 3.15) |
| Montenegro | 38.74 (31.93, 44.72) | 6.26 (5.17, 7.22) |  | 59.86 (47.98, 73.76) | 6.09 (4.93, 7.48) |  | 54.52 | -0.15 (-0.38, 0.08) |
| Morocco | 258.50 (188.98, 318.42) | 2.07 (1.48, 2.57) |  | 652.00 (496.86, 799.60) | 2.31 (1.77, 2.79) |  | 152.23 | 0.20 (-0.07, 0.46) |
| Mozambique | 139.42 (100.55, 224.78) | 2.30 (1.66, 3.71) |  | 458.94 (318.70, 609.32) | 3.99 (2.79, 5.24) |  | 229.17 | 1.86 (1.65, 2.07) |
| Myanmar | 678.82 (492.01, 997.59) | 3.03 (2.23, 4.41) |  | 1955.18 (1629.39, 2324.71) | 4.44 (3.76, 5.26) |  | 188.03 | 1.28 (1.08, 1.47) |
| Namibia | 14.27 (8.92, 25.44) | 1.98 (1.25, 3.47) |  | 49.26 (36.93, 63.75) | 3.46 (2.66, 4.39) |  | 245.35 | 2.02 (1.60, 2.45) |
| Nauru | 0.44 (0.31, 0.59) | 10.89 (7.88, 13.83) |  | 0.43 (0.29, 0.63) | 9.39 (6.70, 12.73) |  | -2.44 | -0.68 (-0.99, -0.38) |
| Nepal | 188.07 (142.23, 242.38) | 1.97 (1.53, 2.47) |  | 490.07 (353.57, 700.83) | 2.36 (1.71, 3.39) |  | 160.58 | 0.70 (0.58, 0.82) |
| Netherlands | 275.03 (258.62, 288.99) | 1.39 (1.31, 1.46) |  | 938.77 (855.14, 1016.30) | 2.75 (2.53, 2.97) |  | 241.33 | 2.67 (2.53, 2.82) |
| New Zealand | 81.54 (76.13, 87.64) | 2.12 (1.98, 2.28) |  | 280.32 (258.58, 301.99) | 3.72 (3.44, 4.00) |  | 243.77 | 2.16 (1.98, 2.34) |
| Nicaragua | 50.93 (44.67, 57.67) | 3.24 (2.81, 3.70) |  | 171.79 (140.18, 209.53) | 4.10 (3.37, 4.96) |  | 237.33 | 0.97 (0.62, 1.33) |
| Niger | 20.21 (15.74, 25.12) | 0.71 (0.55, 0.87) |  | 51.34 (37.75, 68.33) | 0.65 (0.49, 0.84) |  | 154.04 | -0.41 (-0.49, -0.33) |
| Nigeria | 1645.80 (1262.99, 2062.84) | 3.57 (2.71, 4.54) |  | 2920.17 (2284.04, 3704.33) | 3.57 (2.87, 4.44) |  | 77.43 | 0.08 (0.02, 0.14) |
| Niue | 0.18 (0.15, 0.22) | 8.32 (6.66, 10.36) |  | 0.16 (0.12, 0.20) | 7.34 (5.67, 9.36) |  | -12.50 | -0.54 (-0.58, -0.50) |
| North Korea | 2531.27 (1944.43, 3304.26) | 14.77 (11.51, 19.02) |  | 3312.66 (2499.02, 4331.68) | 10.20 (7.75, 13.30) |  | 30.87 | -1.52 (-1.63, -1.41) |
| Northern Mariana Islands | 1.39 (1.04, 1.80) | 6.93 (5.45, 8.53) |  | 4.29 (3.46, 5.36) | 7.84 (6.50, 9.45) |  | 207.66 | 0.73 (0.54, 0.91) |
| Norway | 103.04 (97.79, 107.19) | 1.54 (1.47, 1.60) |  | 231.74 (207.09, 260.52) | 2.45 (2.20, 2.76) |  | 124.90 | 2.07 (1.89, 2.25) |
| Oman | 29.84 (20.96, 40.07) | 4.36 (3.03, 5.79) |  | 82.97 (66.53, 103.08) | 4.75 (3.98, 5.69) |  | 178.02 | 1.06 (0.66, 1.47) |
| Pakistan | 2060.47 (1427.56, 2752.28) | 3.47 (2.31, 4.72) |  | 3911.55 (3164.50, 4832.96) | 3.46 (2.75, 4.30) |  | 89.84 | -0.04 (-0.19, 0.11) |
| Palau | 1.00 (0.69, 1.39) | 9.78 (6.71, 13.43) |  | 2.22 (1.69, 2.93) | 9.79 (7.59, 12.53) |  | 122.45 | 0.00 (-0.07, 0.07) |
| Palestine | 74.36 (55.24, 98.71) | 9.00 (6.80, 11.92) |  | 142.23 (118.35, 171.84) | 6.61 (5.56, 7.88) |  | 91.27 | -1.08 (-1.16, -1.00) |
| Panama | 51.77 (47.72, 55.63) | 3.45 (3.18, 3.72) |  | 126.08 (95.29, 162.12) | 3.06 (2.32, 3.93) |  | 143.54 | -0.09 (-0.51, 0.34) |
| Papua New Guinea | 24.93 (19.27, 31.95) | 1.52 (1.19, 1.93) |  | 66.66 (50.99, 86.93) | 1.67 (1.31, 2.12) |  | 167.38 | 0.39 (0.36, 0.42) |
| Paraguay | 54.56 (47.28, 62.37) | 2.47 (2.13, 2.84) |  | 117.38 (88.13, 152.29) | 2.19 (1.64, 2.82) |  | 115.13 | -0.29 (-0.86, 0.28) |
| Peru | 744.03 (640.88, 854.19) | 6.21 (5.34, 7.13) |  | 873.59 (651.39, 1150.45) | 2.74 (2.04, 3.61) |  | 17.41 | -3.57 (-4.18, -2.96) |
| Philippines | 3150.52 (2361.72, 3839.36) | 10.04 (7.53, 12.27) |  | 5280.37 (4268.78, 6454.15) | 6.58 (5.35, 7.98) |  | 67.60 | -1.84 (-2.14, -1.54) |
| Poland | 3241.88 (3101.79, 3340.81) | 7.57 (7.21, 7.81) |  | 1454.80 (1223.73, 1719.26) | 2.06 (1.73, 2.44) |  | -55.12 | -4.30 (-5.66, -2.93) |
| Portugal | 264.09 (250.16, 278.60) | 1.92 (1.82, 2.03) |  | 1049.79 (962.41, 1136.23) | 4.59 (4.19, 4.97) |  | 297.52 | 2.76 (2.40, 3.12) |
| Puerto Rico | 254.92 (238.65, 268.77) | 6.97 (6.50, 7.36) |  | 194.71 (145.62, 249.96) | 2.74 (2.04, 3.55) |  | -23.62 | -2.18 (-2.97, -1.39) |
| Qatar | 14.47 (10.97, 18.94) | 17.37 (12.99, 22.54) |  | 90.68 (62.74, 123.92) | 15.88 (11.76, 20.79) |  | 526.52 | -0.13 (-0.35, 0.09) |
| Romania | 504.12 (451.51, 565.15) | 1.85 (1.66, 2.06) |  | 1112.39 (906.97, 1343.69) | 3.08 (2.50, 3.75) |  | 120.66 | 2.50 (2.05, 2.95) |
| Russia | 3100.86 (2962.77, 3267.62) | 1.76 (1.68, 1.85) |  | 6825.77 (5811.82, 8144.75) | 2.97 (2.53, 3.55) |  | 120.13 | 2.25 (1.95, 2.54) |
| Rwanda | 162.27 (117.45, 224.74) | 5.36 (3.93, 7.14) |  | 285.60 (218.82, 373.28) | 4.72 (3.78, 5.98) |  | 76.00 | -1.12 (-1.40, -0.84) |
| Saint Kitts and Nevis | 5.06 (4.59, 5.59) | 13.47 (12.28, 14.87) |  | 2.46 (2.04, 2.94) | 3.88 (3.28, 4.57) |  | -51.44 | -4.55 (-5.61, -3.49) |
| Saint Lucia | 5.63 (5.16, 6.14) | 6.45 (5.92, 7.05) |  | 4.84 (4.03, 5.78) | 2.29 (1.91, 2.73) |  | -14.09 | -3.84 (-4.90, -2.77) |
| Saint Vincent and the Grenadines | 5.52 (5.07, 6.00) | 7.61 (7.00, 8.26) |  | 4.19 (3.63, 4.85) | 3.13 (2.72, 3.61) |  | -24.14 | -2.99 (-3.90, -2.07) |
| Samoa | 5.56 (4.39, 7.03) | 6.19 (4.93, 7.80) |  | 7.60 (5.79, 9.64) | 5.06 (3.91, 6.33) |  | 36.53 | -0.72 (-0.80, -0.63) |
| San Marino | 0.69 (0.57, 0.82) | 2.07 (1.71, 2.47) |  | 1.76 (1.17, 2.50) | 2.75 (1.80, 3.98) |  | 156.00 | 1.47 (1.25, 1.70) |
| Sao Tome and Principe | 1.67 (1.29, 2.03) | 2.60 (2.05, 3.13) |  | 3.03 (2.03, 4.02) | 2.80 (1.89, 3.69) |  | 80.66 | 0.19 (0.08, 0.31) |
| Saudi Arabia | 330.43 (235.00, 433.76) | 5.91 (4.21, 7.67) |  | 732.65 (551.53, 958.23) | 4.90 (3.86, 6.25) |  | 121.73 | -0.96 (-1.22, -0.70) |
| Senegal | 79.95 (63.49, 94.78) | 2.39 (1.92, 2.83) |  | 167.28 (124.33, 211.95) | 2.21 (1.65, 2.78) |  | 109.23 | -0.19 (-0.32, -0.05) |
| Serbia | 699.88 (582.33, 807.60) | 6.27 (5.22, 7.21) |  | 884.68 (700.67, 1107.46) | 5.49 (4.36, 6.87) |  | 26.41 | -0.68 (-0.97, -0.39) |
| Seychelles | 4.51 (3.92, 5.21) | 8.01 (6.98, 9.25) |  | 6.23 (5.20, 7.41) | 5.70 (4.81, 6.72) |  | 38.25 | -1.08 (-1.35, -0.81) |
| Sierra Leone | 122.16 (92.29, 156.70) | 6.24 (4.76, 8.03) |  | 180.79 (134.76, 239.08) | 4.94 (3.79, 6.39) |  | 47.99 | -0.88 (-0.98, -0.79) |
| Singapore | 180.14 (169.36, 191.07) | 8.27 (7.75, 8.76) |  | 657.97 (585.65, 731.75) | 8.68 (7.67, 9.66) |  | 265.26 | 0.17 (-0.14, 0.48) |
| Slovakia | 278.71 (261.37, 298.50) | 4.66 (4.37, 4.98) |  | 312.11 (243.20, 395.02) | 3.39 (2.64, 4.28) |  | 11.99 | -1.65 (-1.98, -1.32) |
| Slovenia | 73.56 (56.54, 93.19) | 3.00 (2.31, 3.82) |  | 221.52 (170.59, 282.69) | 5.14 (3.93, 6.61) |  | 201.15 | 2.11 (1.76, 2.47) |
| Solomon Islands | 10.55 (7.88, 13.67) | 6.81 (5.07, 8.82) |  | 18.66 (14.72, 23.06) | 5.52 (4.46, 6.68) |  | 76.94 | -0.71 (-0.79, -0.63) |
| Somalia | 100.04 (60.84, 172.57) | 3.76 (2.35, 6.53) |  | 248.39 (159.96, 433.68) | 3.61 (2.35, 6.40) |  | 148.29 | -0.06 (-0.14, 0.01) |
| South Africa | 1312.43 (867.31, 2258.04) | 6.04 (3.93, 10.36) |  | 2638.04 (2327.93, 2995.33) | 5.87 (5.18, 6.68) |  | 101.00 | -0.58 (-1.21, 0.06) |
| South Korea | 3412.87 (2831.28, 4174.37) | 11.04 (9.25, 13.40) |  | 14477.37 (12937.32, 16038.64) | 16.20 (14.47, 17.94) |  | 324.20 | 1.97 (0.67, 3.28) |
| South Sudan | 81.75 (52.05, 147.08) | 3.26 (2.05, 5.88) |  | 114.69 (67.08, 198.48) | 2.97 (1.79, 5.14) |  | 40.29 | -0.28 (-0.33, -0.23) |
| Spain | 2188.92 (2069.23, 2288.21) | 3.98 (3.76, 4.16) |  | 4973.21 (4472.46, 5437.03) | 5.18 (4.67, 5.67) |  | 127.20 | 0.70 (0.40, 1.00) |
| Sri Lanka | 228.73 (200.07, 260.73) | 2.22 (1.95, 2.52) |  | 705.81 (512.12, 941.04) | 2.84 (2.11, 3.77) |  | 208.57 | 1.84 (1.47, 2.20) |
| Sudan | 344.33 (202.16, 520.73) | 3.80 (2.21, 5.72) |  | 684.11 (414.15, 1074.25) | 3.92 (2.41, 6.04) |  | 98.68 | 0.09 (-0.04, 0.22) |
| Suriname | 17.63 (15.72, 19.61) | 6.83 (6.10, 7.57) |  | 16.16 (12.98, 20.08) | 2.74 (2.21, 3.38) |  | -8.33 | -3.12 (-3.91, -2.31) |
| Sweden | 380.33 (358.21, 399.95) | 2.52 (2.39, 2.65) |  | 624.15 (572.93, 669.65) | 3.02 (2.80, 3.24) |  | 64.11 | 1.19 (0.59, 1.81) |
| Switzerland | 244.46 (230.56, 256.78) | 2.36 (2.23, 2.47) |  | 765.95 (684.55, 849.62) | 4.43 (3.97, 4.91) |  | 213.32 | 1.72 (1.18, 2.26) |
| Syrian Arab Republic | 263.76 (198.70, 333.06) | 5.21 (3.84, 6.69) |  | 501.62 (373.92, 667.14) | 4.41 (3.35, 5.77) |  | 90.18 | -0.75 (-0.93, -0.56) |
| Taiwan (Province of China) | 2024.62 (1941.42, 2109.13) | 11.98 (11.46, 12.47) |  | 2851.69 (2228.42, 3659.70) | 7.27 (5.69, 9.32) |  | 40.85 | -2.69 (-3.54, -1.83) |
| Tajikistan | 32.75 (29.64, 36.25) | 1.07 (0.96, 1.18) |  | 174.25 (139.68, 218.34) | 4.02 (3.23, 5.06) |  | 432.00 | 5.31 (5.03, 5.59) |
| Tanzania | 270.66 (216.63, 331.65) | 2.21 (1.83, 2.69) |  | 639.16 (500.47, 812.63) | 2.46 (1.98, 3.02) |  | 136.15 | 0.31 (0.16, 0.46) |
| Thailand | 7883.04 (6843.63, 9011.14) | 21.60 (18.83, 24.52) |  | 24526.93 (18170.80, 32510.83) | 24.01 (17.88, 31.65) |  | 211.14 | 0.48 (0.35, 0.61) |
| Timor-Leste | 23.08 (15.13, 32.64) | 7.84 (5.35, 10.86) |  | 52.60 (35.38, 73.85) | 6.60 (4.53, 9.09) |  | 127.94 | -0.79 (-0.97, -0.61) |
| Togo | 87.57 (69.41, 111.23) | 6.76 (5.41, 8.54) |  | 192.37 (145.58, 251.34) | 5.24 (4.09, 6.74) |  | 119.67 | -1.31 (-1.50, -1.12) |
| Tokelau | 0.12 (0.09, 0.15) | 8.64 (6.43, 11.29) |  | 0.10 (0.07, 0.13) | 7.48 (5.46, 10.06) |  | -14.90 | -0.58 (-0.62, -0.54) |
| Tonga | 13.65 (9.46, 17.74) | 23.93 (16.74, 30.82) |  | 19.83 (14.46, 25.82) | 24.74 (18.09, 32.04) |  | 45.24 | 0.06 (-0.09, 0.21) |
| Trinidad and Tobago | 59.09 (55.33, 62.97) | 7.10 (6.63, 7.57) |  | 48.60 (36.90, 63.37) | 2.66 (2.03, 3.46) |  | -17.75 | -3.59 (-4.63, -2.53) |
| Tunisia | 96.60 (75.00, 122.65) | 1.98 (1.54, 2.51) |  | 220.33 (155.16, 311.42) | 1.80 (1.27, 2.53) |  | 128.08 | -0.23 (-0.31, -0.15) |
| Turkey | 1376.97 (1104.03, 1665.60) | 3.92 (3.13, 4.69) |  | 2536.79 (2009.00, 3120.69) | 2.96 (2.36, 3.63) |  | 84.23 | -1.00 (-1.19, -0.81) |
| Turkmenistan | 24.47 (22.93, 26.21) | 1.21 (1.13, 1.30) |  | 229.43 (180.93, 291.03) | 5.59 (4.41, 7.07) |  | 837.39 | 6.16 (5.11, 7.21) |
| Tuvalu | 0.74 (0.56, 0.95) | 10.76 (8.16, 13.96) |  | 0.89 (0.65, 1.19) | 8.72 (6.51, 11.52) |  | 19.18 | -0.84 (-0.97, -0.70) |
| Uganda | 329.49 (265.31, 399.29) | 4.75 (3.85, 5.74) |  | 974.26 (762.91, 1223.85) | 6.39 (5.12, 7.88) |  | 195.69 | 1.17 (0.97, 1.37) |
| Ukraine | 704.50 (671.99, 736.75) | 1.02 (0.97, 1.07) |  | 1964.85 (1665.01, 2294.69) | 2.66 (2.24, 3.11) |  | 178.90 | 4.11 (3.52, 4.71) |
| United Arab Emirates | 19.66 (10.50, 35.36) | 4.57 (2.27, 8.67) |  | 202.72 (83.58, 464.33) | 4.90 (2.06, 11.30) |  | 931.31 | 0.17 (-0.10, 0.44) |
| United Kingdom | 1680.82 (1609.11, 1724.52) | 1.87 (1.80, 1.92) |  | 5155.24 (4748.87, 5460.32) | 4.03 (3.75, 4.26) |  | 206.71 | 3.37 (3.16, 3.58) |
| United States of America | 6453.80 (6182.63, 6615.83) | 2.04 (1.96, 2.09) |  | 23806.82 (21184.78, 26095.79) | 4.33 (3.86, 4.75) |  | 268.88 | 2.64 (2.48, 2.81) |
| United States Virgin Islands | 3.57 (2.94, 4.20) | 4.29 (3.54, 5.05) |  | 4.83 (4.00, 5.68) | 2.60 (2.17, 3.07) |  | 35.16 | -1.74 (-2.34, -1.13) |
| Uruguay | 56.05 (49.18, 63.06) | 1.43 (1.25, 1.61) |  | 128.38 (115.91, 141.78) | 2.36 (2.12, 2.61) |  | 129.05 | 2.14 (1.97, 2.31) |
| Uzbekistan | 112.64 (99.93, 123.20) | 0.95 (0.86, 1.02) |  | 1324.64 (1083.90, 1586.83) | 6.68 (5.58, 7.83) |  | 1075.97 | 9.10 (7.95, 10.27) |
| Vanuatu | 6.40 (4.13, 9.61) | 9.62 (6.08, 14.29) |  | 16.44 (11.20, 23.32) | 9.36 (6.41, 13.20) |  | 156.99 | -0.07 (-0.19, 0.06) |
| Venezuela | 639.11 (608.02, 669.57) | 6.75 (6.38, 7.09) |  | 687.52 (531.10, 891.08) | 2.44 (1.88, 3.15) |  | 7.58 | -2.84 (-4.10, -1.57) |
| Viet Nam | 1577.15 (1181.74, 1982.71) | 3.97 (2.95, 4.96) |  | 2387.05 (1839.27, 2982.15) | 2.75 (2.15, 3.40) |  | 51.35 | -1.49 (-1.71, -1.28) |
| Yemen | 159.28 (100.29, 233.74) | 3.42 (2.16, 5.05) |  | 423.33 (308.79, 575.07) | 3.42 (2.49, 4.57) |  | 165.77 | 0.04 (-0.05, 0.13) |
| Zambia | 80.20 (59.74, 112.42) | 2.60 (1.95, 3.76) |  | 202.09 (156.09, 252.40) | 2.99 (2.37, 3.67) |  | 151.99 | -0.17 (-0.56, 0.22) |
| Zimbabwe | 509.88 (395.52, 794.04) | 12.16 (9.51, 18.38) |  | 1016.03 (752.28, 1381.14) | 14.03 (10.58, 18.67) |  | 99.27 | -0.48 (-1.08, 0.13) |

ASMR: age-standardized mortality rate; CI: confidence interval; EAPC: estimated annual percentage change; UI: uncertainty interval.

**Table S19. The deaths and ASMR of primary liver cancer due to hepatitis B in 1990 and 2019 and their change trends from 1990 to 2019 at national level.**

| Nation | 1990 | |  | 2019 | |  | 1990-2019 | |
| --- | --- | --- | --- | --- | --- | --- | --- | --- |
|  | Deaths  No. (95% UI) | ASMR per 100,000  No. (95% UI) |  | Deaths  No. x 10^3^ (95% UI) | ASMR per 100,000  No. (95% UI) |  | Percentage change in deaths  No. (%) | EAPC in ASMR  No. (95% CI) |
| Afghanistan | 332.58 (232.40, 462.25) | 4.42 (3.11, 6.12) |  | 572.06 (397.96, 798.14) | 3.60 (2.51, 4.96) |  | 72.01 | -0.74 (-0.85, -0.64) |
| Albania | 70.04 (51.37, 92.21) | 3.22 (2.34, 4.25) |  | 68.08 (42.30, 102.53) | 1.64 (1.04, 2.44) |  | -2.80 | -3.48 (-4.11, -2.84) |
| Algeria | 77.17 (54.49, 104.86) | 0.61 (0.43, 0.82) |  | 235.03 (161.63, 329.54) | 0.68 (0.46, 0.96) |  | 204.56 | 0.40 (0.19, 0.62) |
| American Samoa | 0.73 (0.50, 0.97) | 2.84 (1.97, 3.75) |  | 1.82 (1.38, 2.39) | 3.58 (2.73, 4.65) |  | 149.05 | 1.22 (1.02, 1.41) |
| Andorra | 0.67 (0.40, 1.04) | 1.18 (0.72, 1.83) |  | 1.54 (0.95, 2.39) | 1.12 (0.70, 1.75) |  | 128.22 | -0.18 (-0.29, -0.08) |
| Angola | 30.50 (20.99, 42.98) | 0.65 (0.44, 0.93) |  | 75.58 (50.34, 108.82) | 0.55 (0.37, 0.79) |  | 147.78 | -0.73 (-0.84, -0.62) |
| Antigua and Barbuda | 1.26 (0.91, 1.71) | 2.44 (1.76, 3.28) |  | 0.71 (0.50, 1.00) | 0.69 (0.48, 0.97) |  | -43.91 | -4.64 (-5.65, -3.62) |
| Argentina | 81.95 (55.31, 116.60) | 0.25 (0.17, 0.36) |  | 187.97 (128.81, 269.56) | 0.35 (0.24, 0.50) |  | 129.37 | 1.80 (1.60, 2.00) |
| Armenia | 6.73 (4.85, 8.86) | 0.24 (0.17, 0.33) |  | 57.12 (37.84, 82.88) | 1.40 (0.94, 2.03) |  | 748.77 | 9.21 (7.58, 10.86) |
| Australia | 56.72 (39.17, 80.60) | 0.30 (0.21, 0.41) |  | 210.35 (143.14, 301.95) | 0.55 (0.38, 0.79) |  | 270.85 | 2.36 (2.13, 2.60) |
| Austria | 28.41 (19.06, 40.98) | 0.26 (0.18, 0.37) |  | 49.57 (31.51, 74.99) | 0.31 (0.20, 0.46) |  | 74.50 | 0.61 (0.30, 0.93) |
| Azerbaijan | 11.83 (8.47, 16.02) | 0.23 (0.16, 0.31) |  | 91.65 (60.55, 136.82) | 0.94 (0.62, 1.39) |  | 674.63 | 6.07 (3.82, 8.38) |
| Bahamas | 4.05 (3.04, 5.36) | 2.40 (1.76, 3.26) |  | 3.60 (2.56, 5.01) | 0.87 (0.62, 1.21) |  | -11.09 | -3.73 (-4.58, -2.88) |
| Bahrain | 3.45 (2.54, 4.64) | 1.88 (1.33, 2.60) |  | 14.52 (9.86, 20.78) | 1.40 (0.95, 2.02) |  | 320.43 | -2.08 (-2.57, -1.59) |
| Bangladesh | 518.46 (373.14, 700.98) | 0.84 (0.60, 1.15) |  | 778.92 (538.48, 1120.48) | 0.56 (0.39, 0.82) |  | 50.24 | -1.97 (-2.18, -1.75) |
| Barbados | 2.12 (1.31, 3.25) | 0.77 (0.48, 1.17) |  | 2.87 (1.97, 4.14) | 0.60 (0.43, 0.86) |  | 35.48 | -1.16 (-1.40, -0.93) |
| Belarus | 36.55 (25.96, 49.72) | 0.28 (0.20, 0.38) |  | 72.00 (43.56, 112.94) | 0.47 (0.29, 0.74) |  | 96.96 | 2.66 (1.85, 3.47) |
| Belgium | 55.42 (37.50, 79.18) | 0.38 (0.26, 0.53) |  | 96.29 (64.47, 139.21) | 0.47 (0.32, 0.67) |  | 73.74 | 0.59 (0.30, 0.87) |
| Belize | 1.63 (1.21, 2.15) | 1.68 (1.24, 2.26) |  | 2.44 (1.78, 3.28) | 0.83 (0.60, 1.14) |  | 49.53 | -2.79 (-3.36, -2.21) |
| Benin | 71.83 (52.99, 95.91) | 3.42 (2.52, 4.60) |  | 119.55 (82.31, 166.53) | 2.22 (1.53, 3.08) |  | 66.44 | -1.84 (-2.01, -1.67) |
| Bermuda | 1.13 (0.81, 1.57) | 1.78 (1.28, 2.50) |  | 0.61 (0.40, 0.87) | 0.49 (0.33, 0.69) |  | -46.22 | -4.29 (-5.42, -3.15) |
| Bhutan | 1.84 (1.05, 3.01) | 0.63 (0.37, 1.03) |  | 4.42 (2.55, 6.90) | 0.75 (0.44, 1.17) |  | 139.79 | 0.58 (0.45, 0.71) |
| Bolivia | 80.88 (57.56, 109.45) | 2.45 (1.74, 3.32) |  | 182.67 (123.96, 254.07) | 2.08 (1.43, 2.87) |  | 125.83 | -0.75 (-0.84, -0.65) |
| Bosnia and Herzegovina | 69.50 (50.12, 93.26) | 1.59 (1.15, 2.13) |  | 99.36 (64.38, 150.74) | 1.69 (1.12, 2.54) |  | 42.95 | 0.41 (0.09, 0.74) |
| Botswana | 2.26 (0.92, 6.07) | 0.35 (0.14, 0.92) |  | 8.68 (5.64, 13.05) | 0.51 (0.33, 0.75) |  | 284.77 | 0.30 (-0.65, 1.27) |
| Brazil | 399.13 (344.72, 455.84) | 0.41 (0.35, 0.47) |  | 997.75 (839.82, 1170.22) | 0.42 (0.35, 0.49) |  | 149.98 | 0.39 (0.26, 0.51) |
| Brunei | 4.90 (3.67, 6.36) | 4.36 (3.21, 5.78) |  | 17.81 (13.37, 22.97) | 4.91 (3.67, 6.40) |  | 263.72 | 1.28 (0.76, 1.81) |
| Bulgaria | 197.82 (139.65, 270.13) | 1.60 (1.16, 2.15) |  | 126.05 (83.30, 189.12) | 1.00 (0.67, 1.47) |  | -36.28 | -1.18 (-1.66, -0.69) |
| Burkina Faso | 59.24 (42.07, 78.80) | 1.31 (0.93, 1.74) |  | 89.08 (62.43, 121.81) | 0.87 (0.60, 1.19) |  | 50.38 | -1.57 (-1.74, -1.40) |
| Burundi | 26.39 (17.44, 37.41) | 1.02 (0.67, 1.45) |  | 42.42 (25.17, 70.82) | 0.79 (0.46, 1.33) |  | 60.75 | -1.41 (-1.61, -1.21) |
| Cambodia | 225.30 (168.14, 296.52) | 3.95 (2.89, 5.31) |  | 389.26 (274.05, 544.99) | 2.95 (2.05, 4.14) |  | 72.77 | -1.24 (-1.37, -1.11) |
| Cameroon | 15.62 (9.96, 24.65) | 0.33 (0.21, 0.52) |  | 41.75 (27.74, 59.71) | 0.31 (0.21, 0.44) |  | 167.32 | -0.47 (-0.78, -0.15) |
| Canada | 48.75 (33.71, 70.64) | 0.15 (0.11, 0.22) |  | 171.21 (111.97, 257.47) | 0.27 (0.18, 0.40) |  | 251.19 | 2.32 (2.11, 2.54) |
| Cape Verde | 1.24 (0.94, 1.60) | 0.54 (0.41, 0.69) |  | 24.20 (18.51, 31.12) | 5.36 (4.04, 6.98) |  | 1846.13 | 6.16 (3.71, 8.68) |
| Central African Republic | 14.15 (8.69, 21.07) | 1.01 (0.63, 1.51) |  | 21.63 (12.22, 35.71) | 0.82 (0.47, 1.35) |  | 52.87 | -1.20 (-1.47, -0.93) |
| Chad | 97.34 (68.44, 131.67) | 3.31 (2.32, 4.49) |  | 166.82 (117.23, 226.12) | 2.76 (1.93, 3.79) |  | 71.37 | -0.77 (-0.89, -0.66) |
| Chile | 43.52 (30.68, 61.10) | 0.42 (0.29, 0.59) |  | 114.23 (77.48, 166.25) | 0.48 (0.33, 0.69) |  | 162.51 | 1.06 (0.82, 1.30) |
| China | 152613.68 (126398.25, 183861.42) | 16.22 (13.47, 19.53) |  | 116997.79 (94646.16, 142974.25) | 5.76 (4.68, 7.02) |  | -23.34 | -5.17 (-6.00, -4.33) |
| Colombia | 111.01 (82.68, 148.09) | 0.57 (0.42, 0.79) |  | 231.37 (146.55, 346.74) | 0.44 (0.28, 0.66) |  | 108.43 | -0.84 (-1.29, -0.38) |
| Comoros | 2.40 (1.03, 4.45) | 1.02 (0.48, 1.87) |  | 4.35 (2.48, 7.59) | 0.84 (0.49, 1.44) |  | 81.09 | -0.92 (-1.10, -0.74) |
| Congo | 12.58 (8.03, 19.24) | 1.01 (0.64, 1.55) |  | 20.09 (12.90, 30.84) | 0.66 (0.42, 1.00) |  | 59.74 | -1.83 (-2.01, -1.65) |
| Cook Islands | 0.93 (0.70, 1.21) | 6.88 (5.16, 8.98) |  | 1.25 (0.93, 1.67) | 5.18 (3.82, 6.86) |  | 35.15 | -0.96 (-1.17, -0.76) |
| Costa Rica | 16.65 (12.12, 22.66) | 0.89 (0.63, 1.24) |  | 38.46 (24.53, 58.73) | 0.74 (0.47, 1.14) |  | 131.05 | -0.95 (-1.45, -0.45) |
| Cote d'Ivoire | 183.61 (129.00, 245.47) | 4.05 (2.90, 5.43) |  | 270.99 (181.91, 384.54) | 2.28 (1.55, 3.27) |  | 47.59 | -2.89 (-3.25, -2.53) |
| Croatia | 46.90 (33.13, 63.82) | 0.73 (0.52, 0.98) |  | 60.55 (38.70, 90.41) | 0.74 (0.49, 1.11) |  | 29.09 | 0.16 (-0.36, 0.69) |
| Cuba | 187.47 (138.33, 249.64) | 1.81 (1.33, 2.40) |  | 107.39 (72.05, 155.46) | 0.58 (0.39, 0.83) |  | -42.71 | -4.28 (-5.67, -2.87) |
| Cyprus | 3.73 (2.42, 5.49) | 0.46 (0.31, 0.67) |  | 7.33 (4.78, 10.87) | 0.39 (0.26, 0.57) |  | 96.68 | -0.54 (-0.78, -0.29) |
| Czech Republic | 132.49 (93.11, 184.01) | 0.98 (0.70, 1.34) |  | 106.91 (69.52, 160.57) | 0.54 (0.36, 0.80) |  | -19.31 | -2.21 (-2.55, -1.87) |
| Democratic Republic of the Congo | 116.01 (79.39, 161.98) | 0.62 (0.42, 0.87) |  | 224.79 (147.32, 336.66) | 0.49 (0.32, 0.76) |  | 93.76 | -0.82 (-0.94, -0.70) |
| Denmark | 19.24 (13.01, 28.03) | 0.26 (0.18, 0.36) |  | 46.64 (30.60, 69.17) | 0.45 (0.30, 0.65) |  | 142.38 | 2.36 (2.10, 2.61) |
| Djibouti | 1.78 (0.97, 3.50) | 1.01 (0.55, 1.97) |  | 6.86 (3.68, 12.23) | 0.98 (0.55, 1.74) |  | 285.09 | -0.15 (-0.32, 0.02) |
| Dominica | 1.68 (1.19, 2.38) | 2.47 (1.78, 3.36) |  | 0.74 (0.49, 1.05) | 0.84 (0.56, 1.19) |  | -56.08 | -3.78 (-4.59, -2.96) |
| Dominican Republic | 49.38 (37.18, 64.12) | 1.18 (0.87, 1.57) |  | 125.41 (74.30, 209.45) | 1.30 (0.77, 2.15) |  | 153.97 | 0.47 (-0.11, 1.05) |
| Ecuador | 77.42 (62.31, 94.11) | 1.41 (1.10, 1.73) |  | 222.12 (155.69, 302.85) | 1.49 (1.04, 2.03) |  | 186.91 | 0.50 (0.20, 0.80) |
| Egypt | 761.57 (511.41, 1119.83) | 2.26 (1.50, 3.33) |  | 1773.98 (1060.09, 2858.61) | 2.41 (1.46, 3.89) |  | 132.94 | 0.72 (0.46, 0.98) |
| El Salvador | 20.06 (14.69, 27.05) | 0.63 (0.45, 0.87) |  | 18.16 (11.23, 27.67) | 0.31 (0.19, 0.47) |  | -9.46 | -2.79 (-3.48, -2.10) |
| Equatorial Guinea | 1.82 (1.19, 2.71) | 0.79 (0.52, 1.17) |  | 4.55 (2.41, 7.35) | 0.77 (0.41, 1.25) |  | 150.21 | -0.18 (-0.41, 0.06) |
| Eritrea | 12.39 (7.63, 19.48) | 1.02 (0.63, 1.63) |  | 28.02 (16.67, 43.92) | 0.87 (0.52, 1.37) |  | 126.10 | -0.93 (-1.14, -0.73) |
| Estonia | 10.21 (7.26, 14.11) | 0.51 (0.36, 0.69) |  | 15.86 (10.00, 23.45) | 0.67 (0.43, 0.99) |  | 55.33 | -0.25 (-0.94, 0.44) |
| Eswatini | 7.18 (3.98, 15.60) | 2.09 (1.15, 4.48) |  | 51.89 (12.21, 99.88) | 7.43 (1.82, 14.12) |  | 622.43 | 5.37 (3.86, 6.92) |
| Ethiopia | 153.33 (102.38, 226.87) | 0.67 (0.45, 0.96) |  | 274.32 (204.06, 367.28) | 0.60 (0.44, 0.81) |  | 78.91 | -0.53 (-0.66, -0.40) |
| Federated States of Micronesia | 3.04 (2.08, 4.16) | 5.95 (4.04, 8.28) |  | 4.28 (2.60, 6.42) | 5.31 (3.29, 7.72) |  | 41.02 | -0.46 (-0.63, -0.28) |
| Fiji | 12.14 (9.06, 15.66) | 2.94 (2.17, 3.86) |  | 24.12 (17.13, 33.50) | 2.94 (2.10, 4.07) |  | 98.70 | 0.58 (0.11, 1.05) |
| Finland | 27.01 (18.42, 38.93) | 0.39 (0.27, 0.56) |  | 56.76 (36.66, 86.11) | 0.51 (0.35, 0.73) |  | 110.18 | 1.22 (1.08, 1.35) |
| France | 529.68 (356.16, 756.84) | 0.69 (0.47, 0.98) |  | 897.35 (585.65, 1331.06) | 0.76 (0.50, 1.11) |  | 69.41 | 0.01 (-0.25, 0.27) |
| Gabon | 4.43 (2.83, 6.39) | 0.72 (0.46, 1.04) |  | 7.98 (4.64, 12.75) | 0.68 (0.40, 1.10) |  | 80.07 | -0.32 (-0.54, -0.11) |
| Gambia | 69.95 (49.60, 96.20) | 17.14 (12.17, 23.10) |  | 222.49 (155.40, 312.11) | 20.34 (14.26, 28.33) |  | 218.08 | 0.23 (0.04, 0.42) |
| Georgia | 16.01 (11.32, 22.17) | 0.26 (0.18, 0.36) |  | 46.50 (31.55, 66.95) | 0.87 (0.60, 1.25) |  | 190.53 | 3.32 (1.95, 4.70) |
| Germany | 353.03 (249.53, 492.76) | 0.29 (0.21, 0.40) |  | 693.17 (458.19, 1016.61) | 0.40 (0.27, 0.59) |  | 96.35 | 1.04 (0.65, 1.44) |
| Ghana | 211.45 (152.00, 294.10) | 3.11 (2.23, 4.28) |  | 502.89 (352.88, 685.43) | 2.83 (1.99, 3.86) |  | 137.83 | -0.62 (-0.84, -0.41) |
| Greece | 118.57 (87.35, 152.05) | 0.78 (0.58, 0.99) |  | 249.03 (182.39, 333.31) | 1.13 (0.85, 1.48) |  | 110.03 | 1.48 (1.31, 1.66) |
| Greenland | 0.26 (0.17, 0.39) | 0.61 (0.41, 0.93) |  | 0.55 (0.34, 0.86) | 0.76 (0.47, 1.15) |  | 111.39 | 0.92 (0.83, 1.00) |
| Grenada | 1.76 (1.29, 2.40) | 2.58 (1.91, 3.46) |  | 0.85 (0.60, 1.19) | 0.74 (0.52, 1.02) |  | -51.70 | -4.64 (-5.63, -3.65) |
| Guam | 1.70 (1.33, 2.16) | 1.99 (1.56, 2.54) |  | 6.43 (4.87, 8.26) | 3.33 (2.54, 4.24) |  | 278.07 | 2.43 (2.19, 2.67) |
| Guatemala | 71.74 (51.64, 97.41) | 1.75 (1.24, 2.43) |  | 87.51 (57.51, 128.33) | 0.74 (0.48, 1.10) |  | 21.99 | -3.90 (-5.33, -2.43) |
| Guinea | 573.76 (429.27, 738.02) | 16.82 (12.73, 21.71) |  | 1033.44 (692.61, 1417.07) | 17.49 (11.60, 23.83) |  | 80.12 | 0.31 (0.22, 0.41) |
| Guinea-Bissau | 18.78 (12.65, 27.32) | 4.26 (2.89, 6.06) |  | 23.91 (16.31, 34.33) | 2.91 (1.99, 4.10) |  | 27.29 | -1.37 (-1.47, -1.28) |
| Guyana | 7.84 (5.81, 10.55) | 1.85 (1.32, 2.54) |  | 5.48 (3.64, 7.96) | 0.82 (0.55, 1.18) |  | -30.13 | -2.87 (-3.64, -2.10) |
| Haiti | 67.80 (39.75, 107.56) | 1.95 (1.14, 3.09) |  | 91.02 (49.76, 152.39) | 1.22 (0.68, 2.08) |  | 34.25 | -1.74 (-1.98, -1.50) |
| Honduras | 50.42 (17.83, 84.79) | 2.26 (0.74, 3.86) |  | 158.81 (63.06, 275.03) | 2.53 (0.98, 4.37) |  | 214.95 | 0.47 (0.34, 0.61) |
| Hungary | 212.27 (149.16, 292.14) | 1.47 (1.06, 1.99) |  | 103.24 (68.63, 150.76) | 0.58 (0.39, 0.83) |  | -51.36 | -2.54 (-3.40, -1.66) |
| Iceland | 0.82 (0.57, 1.19) | 0.30 (0.21, 0.43) |  | 2.17 (1.46, 3.20) | 0.42 (0.29, 0.61) |  | 165.29 | 1.15 (1.01, 1.29) |
| India | 4898.15 (3918.52, 5898.32) | 1.01 (0.80, 1.22) |  | 10783.87 (8690.49, 13219.93) | 0.91 (0.74, 1.12) |  | 120.16 | -0.26 (-0.40, -0.12) |
| Indonesia | 573.60 (474.16, 689.36) | 0.50 (0.41, 0.61) |  | 973.68 (749.68, 1221.59) | 0.43 (0.34, 0.53) |  | 69.75 | -0.69 (-0.84, -0.54) |
| Iran | 457.52 (377.33, 539.54) | 1.72 (1.41, 2.03) |  | 973.82 (834.24, 1127.64) | 1.32 (1.13, 1.55) |  | 112.85 | -1.38 (-2.03, -0.72) |
| Iraq | 132.71 (93.37, 183.35) | 1.60 (1.11, 2.21) |  | 487.51 (331.31, 690.87) | 1.97 (1.34, 2.76) |  | 267.34 | 0.89 (0.48, 1.31) |
| Ireland | 7.72 (5.20, 11.27) | 0.19 (0.13, 0.27) |  | 29.64 (19.75, 43.82) | 0.41 (0.28, 0.60) |  | 283.86 | 3.52 (3.13, 3.90) |
| Israel | 19.87 (13.51, 29.69) | 0.41 (0.29, 0.61) |  | 44.78 (30.08, 65.64) | 0.41 (0.28, 0.59) |  | 125.41 | -0.21 (-0.32, -0.09) |
| Italy | 687.13 (573.05, 824.11) | 0.80 (0.68, 0.96) |  | 654.36 (535.99, 798.62) | 0.52 (0.43, 0.64) |  | -4.77 | -2.19 (-2.56, -1.81) |
| Jamaica | 20.81 (15.27, 27.70) | 1.19 (0.87, 1.57) |  | 21.10 (14.35, 29.51) | 0.71 (0.48, 1.00) |  | 1.38 | -1.08 (-2.02, -0.13) |
| Japan | 2934.09 (2545.63, 3384.57) | 1.71 (1.49, 1.96) |  | 3368.15 (2759.74, 4091.38) | 1.05 (0.89, 1.24) |  | 14.79 | -2.63 (-3.14, -2.12) |
| Jordan | 16.00 (11.71, 21.80) | 1.12 (0.80, 1.56) |  | 51.88 (36.28, 71.87) | 0.77 (0.53, 1.08) |  | 224.32 | -1.65 (-1.80, -1.49) |
| Kazakhstan | 115.54 (83.22, 155.60) | 0.86 (0.61, 1.17) |  | 257.27 (177.44, 364.08) | 1.40 (0.96, 1.98) |  | 122.67 | -2.01 (-3.29, -0.71) |
| Kenya | 61.93 (38.80, 109.52) | 0.67 (0.42, 1.18) |  | 208.25 (132.45, 312.46) | 0.81 (0.52, 1.20) |  | 236.29 | 0.06 (-0.41, 0.54) |
| Kiribati | 3.37 (2.47, 4.48) | 7.71 (5.69, 10.33) |  | 5.07 (3.63, 6.81) | 5.97 (4.30, 8.02) |  | 50.33 | -0.97 (-1.05, -0.88) |
| Kuwait | 7.42 (5.82, 9.33) | 1.02 (0.75, 1.34) |  | 19.83 (13.48, 28.25) | 0.73 (0.48, 1.07) |  | 167.35 | -0.41 (-0.77, -0.05) |
| Kyrgyzstan | 9.09 (6.49, 12.32) | 0.29 (0.21, 0.40) |  | 26.52 (18.25, 37.04) | 0.54 (0.38, 0.77) |  | 191.94 | 2.89 (2.42, 3.36) |
| Lao | 98.94 (61.60, 146.61) | 4.31 (2.71, 6.29) |  | 128.51 (86.33, 181.35) | 2.64 (1.79, 3.70) |  | 29.89 | -1.92 (-2.01, -1.83) |
| Latvia | 14.09 (10.00, 19.43) | 0.40 (0.29, 0.55) |  | 18.50 (12.34, 27.16) | 0.53 (0.36, 0.77) |  | 31.35 | -0.19 (-1.13, 0.76) |
| Lebanon | 42.06 (31.33, 54.34) | 1.80 (1.36, 2.31) |  | 75.55 (52.28, 107.47) | 1.45 (1.00, 2.06) |  | 79.62 | -0.81 (-0.91, -0.70) |
| Lesotho | 20.67 (10.88, 48.72) | 1.94 (1.03, 4.51) |  | 81.12 (27.44, 140.68) | 5.62 (1.95, 9.50) |  | 292.41 | 4.02 (3.13, 4.92) |
| Liberia | 40.64 (29.70, 54.75) | 3.57 (2.60, 4.78) |  | 54.17 (35.24, 84.93) | 2.38 (1.57, 3.65) |  | 33.30 | -1.54 (-1.71, -1.37) |
| Libya | 39.13 (26.79, 54.16) | 1.93 (1.33, 2.70) |  | 97.08 (64.30, 143.93) | 1.71 (1.14, 2.50) |  | 148.11 | -0.55 (-0.64, -0.45) |
| Lithuania | 17.53 (12.59, 23.77) | 0.39 (0.29, 0.53) |  | 30.41 (20.01, 44.94) | 0.61 (0.40, 0.90) |  | 73.45 | 0.48 (0.01, 0.95) |
| Luxembourg | 1.94 (1.29, 2.85) | 0.37 (0.25, 0.53) |  | 3.94 (2.46, 6.01) | 0.41 (0.26, 0.63) |  | 103.27 | 0.33 (0.02, 0.65) |
| Macedonia | 44.24 (31.33, 60.42) | 2.28 (1.62, 3.11) |  | 63.23 (41.09, 94.27) | 1.97 (1.30, 2.90) |  | 42.94 | -0.65 (-0.88, -0.42) |
| Madagascar | 56.23 (35.38, 99.89) | 0.97 (0.59, 1.75) |  | 95.88 (57.26, 159.29) | 0.74 (0.43, 1.25) |  | 70.49 | -1.19 (-1.33, -1.06) |
| Malawi | 43.62 (28.78, 69.13) | 0.99 (0.63, 1.61) |  | 61.21 (40.78, 87.08) | 0.75 (0.50, 1.10) |  | 40.32 | -2.14 (-2.65, -1.64) |
| Malaysia | 318.50 (252.29, 386.41) | 3.28 (2.57, 4.07) |  | 922.02 (655.07, 1217.64) | 3.36 (2.39, 4.44) |  | 189.49 | 0.24 (-0.03, 0.50) |
| Maldives | 2.86 (1.75, 4.73) | 3.06 (1.90, 4.88) |  | 6.99 (5.03, 9.29) | 2.08 (1.45, 2.84) |  | 143.81 | -1.36 (-1.52, -1.20) |
| Mali | 280.18 (198.48, 370.11) | 6.03 (4.31, 8.08) |  | 534.61 (345.54, 775.12) | 5.43 (3.47, 7.84) |  | 90.81 | -0.55 (-0.65, -0.45) |
| Malta | 1.00 (0.66, 1.48) | 0.23 (0.16, 0.34) |  | 2.40 (1.59, 3.56) | 0.29 (0.19, 0.42) |  | 140.90 | 0.61 (0.33, 0.89) |
| Marshall Islands | 1.16 (0.83, 1.62) | 6.42 (4.50, 9.11) |  | 2.16 (1.45, 3.14) | 5.38 (3.58, 7.88) |  | 86.68 | -0.56 (-0.63, -0.48) |
| Mauritania | 39.11 (29.43, 50.23) | 3.71 (2.79, 4.77) |  | 43.43 (29.34, 61.06) | 2.02 (1.39, 2.83) |  | 11.04 | -2.16 (-2.31, -2.00) |
| Mauritius | 5.46 (4.15, 6.89) | 0.70 (0.53, 0.90) |  | 11.26 (7.43, 16.10) | 0.64 (0.43, 0.93) |  | 106.28 | 0.98 (0.61, 1.35) |
| Mexico | 139.32 (118.59, 164.72) | 0.30 (0.26, 0.36) |  | 476.15 (374.29, 596.58) | 0.40 (0.32, 0.50) |  | 241.76 | 0.85 (0.65, 1.05) |
| Moldova | 14.45 (10.31, 19.97) | 0.32 (0.23, 0.44) |  | 23.91 (16.22, 34.87) | 0.42 (0.29, 0.61) |  | 65.50 | -1.53 (-3.34, 0.32) |
| Monaco | 0.29 (0.18, 0.46) | 0.48 (0.31, 0.73) |  | 0.83 (0.53, 1.23) | 1.02 (0.66, 1.50) |  | 184.08 | 3.04 (2.23, 3.85) |
| Mongolia | 236.69 (164.55, 326.64) | 21.66 (15.15, 30.17) |  | 704.78 (467.69, 1024.09) | 28.23 (18.92, 40.83) |  | 197.77 | 1.32 (0.96, 1.68) |
| Montenegro | 9.09 (6.31, 12.94) | 1.42 (0.98, 2.01) |  | 12.17 (8.01, 17.83) | 1.28 (0.85, 1.84) |  | 33.96 | -0.36 (-0.74, 0.01) |
| Morocco | 99.92 (69.98, 138.47) | 0.74 (0.51, 1.02) |  | 229.96 (152.92, 326.02) | 0.74 (0.49, 1.06) |  | 130.14 | -0.21 (-0.45, 0.03) |
| Mozambique | 55.52 (34.33, 98.31) | 0.88 (0.54, 1.55) |  | 182.58 (115.51, 261.31) | 1.49 (0.95, 2.15) |  | 228.87 | 1.75 (1.49, 2.02) |
| Myanmar | 249.45 (160.68, 381.83) | 1.04 (0.68, 1.58) |  | 644.98 (456.74, 891.10) | 1.35 (0.95, 1.88) |  | 158.56 | 0.89 (0.69, 1.10) |
| Namibia | 4.94 (2.57, 10.14) | 0.65 (0.34, 1.32) |  | 16.85 (11.09, 24.19) | 1.08 (0.72, 1.55) |  | 241.03 | 1.97 (1.42, 2.51) |
| Nauru | 0.27 (0.17, 0.37) | 5.66 (3.84, 7.66) |  | 0.25 (0.16, 0.39) | 4.60 (3.09, 6.58) |  | -3.89 | -0.89 (-1.23, -0.56) |
| Nepal | 48.11 (31.97, 68.80) | 0.48 (0.31, 0.68) |  | 110.04 (67.39, 173.58) | 0.49 (0.30, 0.78) |  | 128.72 | 0.09 (0.01, 0.18) |
| Netherlands | 33.92 (23.33, 48.41) | 0.18 (0.12, 0.25) |  | 104.52 (69.95, 151.38) | 0.34 (0.24, 0.48) |  | 208.09 | 2.54 (2.39, 2.70) |
| New Zealand | 14.06 (11.70, 16.92) | 0.38 (0.31, 0.45) |  | 41.88 (34.77, 50.66) | 0.61 (0.51, 0.73) |  | 197.95 | 1.96 (1.79, 2.14) |
| Nicaragua | 9.86 (7.09, 13.58) | 0.56 (0.38, 0.81) |  | 25.51 (16.56, 37.05) | 0.55 (0.36, 0.81) |  | 158.70 | 0.02 (-0.43, 0.46) |
| Niger | 11.16 (8.09, 14.88) | 0.37 (0.27, 0.48) |  | 26.44 (17.72, 36.68) | 0.31 (0.21, 0.43) |  | 136.84 | -0.58 (-0.67, -0.50) |
| Nigeria | 645.79 (474.63, 841.63) | 1.39 (1.01, 1.81) |  | 1129.38 (840.72, 1479.76) | 1.30 (0.99, 1.70) |  | 74.88 | -0.13 (-0.22, -0.05) |
| Niue | 0.09 (0.06, 0.12) | 4.21 (3.07, 5.51) |  | 0.08 (0.05, 0.10) | 3.54 (2.52, 4.84) |  | -13.96 | -0.74 (-0.82, -0.66) |
| North Korea | 1576.35 (1139.75, 2155.51) | 8.58 (6.22, 11.52) |  | 1936.37 (1346.98, 2687.15) | 5.84 (4.08, 8.10) |  | 22.84 | -1.58 (-1.70, -1.46) |
| Northern Mariana Islands | 0.85 (0.59, 1.15) | 3.53 (2.52, 4.67) |  | 2.53 (1.90, 3.36) | 4.26 (3.21, 5.54) |  | 196.28 | 1.06 (0.84, 1.27) |
| Norway | 14.58 (12.26, 17.17) | 0.24 (0.21, 0.29) |  | 28.56 (22.91, 35.66) | 0.33 (0.27, 0.41) |  | 95.93 | 1.54 (1.32, 1.75) |
| Oman | 12.72 (7.98, 18.56) | 1.61 (1.01, 2.38) |  | 35.99 (24.78, 50.38) | 1.60 (1.13, 2.24) |  | 182.83 | 0.63 (0.20, 1.06) |
| Pakistan | 321.41 (216.72, 446.58) | 0.50 (0.33, 0.71) |  | 684.32 (527.51, 883.09) | 0.51 (0.39, 0.66) |  | 112.91 | -0.04 (-0.23, 0.15) |
| Palau | 0.62 (0.40, 0.89) | 5.74 (3.72, 8.18) |  | 1.35 (0.96, 1.86) | 5.55 (4.02, 7.45) |  | 117.50 | -0.17 (-0.25, -0.10) |
| Palestine | 24.86 (16.75, 36.13) | 2.81 (1.88, 4.08) |  | 50.37 (37.01, 67.17) | 2.02 (1.46, 2.75) |  | 102.61 | -1.28 (-1.41, -1.16) |
| Panama | 9.54 (6.87, 13.15) | 0.59 (0.42, 0.83) |  | 18.24 (11.64, 27.42) | 0.44 (0.28, 0.66) |  | 91.31 | -0.88 (-1.27, -0.49) |
| Papua New Guinea | 12.39 (8.68, 17.24) | 0.69 (0.48, 0.94) |  | 32.14 (22.23, 44.84) | 0.72 (0.50, 1.00) |  | 159.34 | 0.23 (0.20, 0.27) |
| Paraguay | 9.37 (6.53, 13.14) | 0.40 (0.27, 0.57) |  | 20.56 (12.73, 31.59) | 0.36 (0.23, 0.56) |  | 119.37 | -0.11 (-0.64, 0.43) |
| Peru | 345.79 (268.41, 439.11) | 2.79 (2.12, 3.58) |  | 353.05 (238.98, 505.55) | 1.10 (0.74, 1.58) |  | 2.10 | -3.98 (-4.56, -3.40) |
| Philippines | 1587.93 (1160.41, 2009.54) | 4.52 (3.25, 5.75) |  | 2363.56 (1849.36, 2974.75) | 2.69 (2.10, 3.37) |  | 48.85 | -2.26 (-2.55, -1.97) |
| Poland | 718.97 (613.23, 840.88) | 1.66 (1.43, 1.93) |  | 260.18 (204.46, 335.61) | 0.39 (0.31, 0.50) |  | -63.81 | -5.02 (-6.29, -3.74) |
| Portugal | 35.20 (24.32, 49.66) | 0.27 (0.19, 0.37) |  | 124.99 (84.17, 183.11) | 0.63 (0.43, 0.91) |  | 255.08 | 2.69 (2.28, 3.09) |
| Puerto Rico | 64.66 (46.37, 87.41) | 1.78 (1.29, 2.39) |  | 43.14 (26.94, 64.48) | 0.67 (0.43, 0.99) |  | -33.29 | -2.27 (-3.02, -1.51) |
| Qatar | 6.10 (4.13, 8.89) | 5.44 (3.54, 8.22) |  | 37.39 (23.52, 56.85) | 4.36 (2.71, 6.73) |  | 512.64 | -0.81 (-1.02, -0.60) |
| Romania | 118.52 (83.78, 160.32) | 0.43 (0.31, 0.57) |  | 209.26 (140.88, 304.68) | 0.63 (0.43, 0.90) |  | 76.57 | 1.90 (1.44, 2.37) |
| Russia | 815.56 (694.40, 964.90) | 0.45 (0.39, 0.53) |  | 1503.27 (1149.52, 1934.72) | 0.68 (0.53, 0.88) |  | 84.33 | 1.76 (1.42, 2.09) |
| Rwanda | 45.77 (30.09, 68.71) | 1.40 (0.92, 2.07) |  | 79.11 (50.93, 114.97) | 1.13 (0.74, 1.62) |  | 72.84 | -1.50 (-1.80, -1.19) |
| Saint Kitts and Nevis | 1.35 (0.97, 1.83) | 3.81 (2.82, 4.97) |  | 0.71 (0.47, 1.01) | 1.01 (0.68, 1.43) |  | -47.82 | -5.11 (-6.16, -4.05) |
| Saint Lucia | 1.48 (1.10, 1.97) | 1.66 (1.23, 2.20) |  | 1.18 (0.83, 1.64) | 0.55 (0.38, 0.76) |  | -20.28 | -3.98 (-5.00, -2.94) |
| Saint Vincent and the Grenadines | 1.59 (1.16, 2.13) | 2.18 (1.60, 2.91) |  | 1.10 (0.78, 1.49) | 0.81 (0.58, 1.09) |  | -30.77 | -3.37 (-4.16, -2.57) |
| Samoa | 3.11 (2.23, 4.09) | 3.33 (2.40, 4.36) |  | 4.19 (3.02, 5.70) | 2.66 (1.93, 3.59) |  | 34.85 | -0.79 (-0.90, -0.68) |
| San Marino | 0.09 (0.06, 0.14) | 0.28 (0.19, 0.43) |  | 0.20 (0.11, 0.34) | 0.35 (0.19, 0.58) |  | 120.08 | 1.08 (0.86, 1.30) |
| Sao Tome and Principe | 0.83 (0.59, 1.07) | 1.26 (0.90, 1.64) |  | 1.48 (0.92, 2.14) | 1.24 (0.78, 1.75) |  | 79.41 | -0.15 (-0.26, -0.05) |
| Saudi Arabia | 140.15 (91.10, 198.15) | 2.22 (1.44, 3.17) |  | 287.48 (192.12, 413.07) | 1.54 (1.01, 2.25) |  | 105.12 | -1.80 (-2.04, -1.55) |
| Senegal | 46.54 (36.08, 57.66) | 1.40 (1.08, 1.74) |  | 95.68 (67.26, 127.79) | 1.23 (0.87, 1.62) |  | 105.60 | -0.44 (-0.57, -0.31) |
| Serbia | 163.12 (111.19, 230.01) | 1.40 (0.96, 1.94) |  | 157.75 (98.38, 241.81) | 1.04 (0.67, 1.56) |  | -3.30 | -1.30 (-1.71, -0.90) |
| Seychelles | 1.83 (1.39, 2.41) | 3.27 (2.47, 4.31) |  | 2.37 (1.71, 3.18) | 2.03 (1.48, 2.71) |  | 29.30 | -1.70 (-2.00, -1.39) |
| Sierra Leone | 60.45 (41.69, 82.19) | 3.06 (2.13, 4.19) |  | 86.37 (59.44, 123.07) | 2.21 (1.52, 3.14) |  | 42.89 | -1.18 (-1.28, -1.08) |
| Singapore | 114.57 (97.13, 132.28) | 5.01 (4.17, 5.88) |  | 361.79 (282.67, 443.35) | 4.65 (3.62, 5.71) |  | 215.79 | -0.28 (-0.60, 0.04) |
| Slovakia | 63.68 (45.58, 86.05) | 1.07 (0.78, 1.45) |  | 59.08 (37.00, 89.30) | 0.66 (0.42, 0.99) |  | -7.21 | -2.31 (-2.72, -1.90) |
| Slovenia | 17.44 (11.36, 25.72) | 0.71 (0.47, 1.05) |  | 45.18 (28.51, 67.66) | 1.13 (0.71, 1.70) |  | 159.14 | 1.62 (1.15, 2.11) |
| Solomon Islands | 6.06 (4.28, 8.11) | 3.70 (2.57, 4.98) |  | 10.11 (7.55, 13.38) | 2.73 (2.05, 3.57) |  | 66.89 | -1.04 (-1.14, -0.93) |
| Somalia | 37.15 (20.54, 71.59) | 1.24 (0.68, 2.38) |  | 89.13 (52.00, 167.71) | 1.13 (0.67, 2.12) |  | 139.89 | -0.29 (-0.36, -0.21) |
| South Africa | 493.85 (317.68, 876.96) | 2.04 (1.28, 3.65) |  | 960.47 (807.78, 1131.97) | 1.94 (1.63, 2.29) |  | 94.49 | -0.69 (-1.35, -0.01) |
| South Korea | 2220.27 (1770.57, 2766.48) | 6.65 (5.29, 8.21) |  | 7876.21 (6388.91, 9395.61) | 8.76 (7.19, 10.42) |  | 254.74 | 1.71 (0.33, 3.11) |
| South Sudan | 26.15 (14.43, 52.67) | 1.01 (0.55, 2.02) |  | 38.16 (19.76, 72.27) | 0.89 (0.46, 1.69) |  | 45.91 | -0.42 (-0.49, -0.35) |
| Spain | 230.42 (158.23, 330.44) | 0.44 (0.31, 0.62) |  | 467.28 (310.04, 685.01) | 0.56 (0.37, 0.81) |  | 102.80 | 0.55 (0.24, 0.85) |
| Sri Lanka | 97.94 (74.95, 126.34) | 0.87 (0.65, 1.14) |  | 252.23 (161.09, 371.66) | 0.98 (0.64, 1.44) |  | 157.54 | 1.35 (0.92, 1.78) |
| Sudan | 128.53 (74.74, 200.24) | 1.35 (0.77, 2.13) |  | 241.15 (132.88, 395.66) | 1.27 (0.71, 2.07) |  | 87.62 | -0.27 (-0.37, -0.16) |
| Suriname | 5.32 (3.97, 7.05) | 1.94 (1.42, 2.59) |  | 4.36 (2.99, 6.20) | 0.71 (0.49, 1.00) |  | -17.93 | -3.47 (-4.22, -2.72) |
| Sweden | 20.95 (16.09, 27.16) | 0.16 (0.12, 0.20) |  | 32.70 (24.64, 42.28) | 0.18 (0.14, 0.24) |  | 56.08 | 1.18 (0.55, 1.81) |
| Switzerland | 30.21 (20.24, 44.02) | 0.31 (0.21, 0.45) |  | 93.05 (61.03, 135.98) | 0.59 (0.39, 0.85) |  | 207.98 | 1.67 (1.09, 2.26) |
| Syrian Arab Republic | 102.56 (73.04, 142.01) | 1.83 (1.25, 2.58) |  | 178.97 (118.07, 260.33) | 1.40 (0.92, 2.04) |  | 74.50 | -1.18 (-1.40, -0.96) |
| Taiwan (Province of China) | 1121.62 (948.35, 1290.42) | 6.34 (5.34, 7.33) |  | 1189.97 (846.92, 1621.26) | 3.08 (2.22, 4.17) |  | 6.09 | -3.58 (-4.33, -2.82) |
| Tajikistan | 5.20 (3.68, 7.10) | 0.18 (0.12, 0.24) |  | 36.04 (24.68, 50.85) | 0.63 (0.42, 0.89) |  | 592.56 | 5.04 (4.78, 5.30) |
| Tanzania | 70.02 (49.29, 99.38) | 0.58 (0.40, 0.82) |  | 160.67 (109.13, 229.52) | 0.58 (0.39, 0.84) |  | 129.48 | -0.08 (-0.24, 0.09) |
| Thailand | 3881.55 (3073.47, 4784.19) | 9.74 (7.65, 12.15) |  | 9765.44 (6627.25, 13873.57) | 9.50 (6.50, 13.47) |  | 151.59 | -0.13 (-0.23, -0.04) |
| Timor-Leste | 11.26 (7.01, 17.55) | 3.28 (2.02, 5.14) |  | 21.46 (11.86, 33.24) | 2.54 (1.42, 3.92) |  | 90.62 | -1.04 (-1.22, -0.86) |
| Togo | 45.70 (33.57, 60.55) | 3.32 (2.42, 4.43) |  | 96.75 (68.12, 134.34) | 2.36 (1.68, 3.25) |  | 111.73 | -1.59 (-1.82, -1.36) |
| Tokelau | 0.06 (0.04, 0.08) | 4.30 (2.87, 6.05) |  | 0.05 (0.03, 0.07) | 3.74 (2.43, 5.33) |  | -11.71 | -0.55 (-0.59, -0.51) |
| Tonga | 7.81 (5.08, 10.84) | 13.10 (8.57, 18.15) |  | 10.51 (7.13, 14.86) | 12.87 (8.72, 18.05) |  | 34.55 | -0.11 (-0.20, -0.01) |
| Trinidad and Tobago | 16.60 (12.32, 21.96) | 1.91 (1.41, 2.55) |  | 11.95 (7.67, 17.80) | 0.65 (0.42, 0.96) |  | -27.98 | -3.96 (-4.96, -2.94) |
| Tunisia | 30.76 (20.65, 44.31) | 0.58 (0.39, 0.84) |  | 61.03 (36.31, 94.55) | 0.48 (0.29, 0.74) |  | 98.38 | -0.69 (-0.79, -0.58) |
| Turkey | 671.54 (505.66, 853.32) | 1.80 (1.34, 2.30) |  | 1063.38 (771.28, 1448.01) | 1.20 (0.86, 1.63) |  | 58.35 | -1.54 (-1.68, -1.41) |
| Turkmenistan | 5.28 (3.84, 7.07) | 0.26 (0.19, 0.36) |  | 63.19 (42.58, 90.32) | 1.40 (0.94, 1.99) |  | 1097.66 | 6.76 (5.45, 8.08) |
| Tuvalu | 0.41 (0.28, 0.56) | 5.58 (3.91, 7.73) |  | 0.47 (0.33, 0.67) | 4.43 (3.08, 6.32) |  | 15.25 | -0.86 (-1.01, -0.71) |
| Uganda | 100.19 (69.25, 142.15) | 1.38 (0.93, 1.98) |  | 283.73 (191.08, 409.01) | 1.63 (1.10, 2.35) |  | 183.19 | 0.58 (0.35, 0.80) |
| Ukraine | 161.33 (135.83, 191.45) | 0.23 (0.19, 0.27) |  | 451.32 (355.43, 565.63) | 0.64 (0.51, 0.81) |  | 179.75 | 4.49 (3.72, 5.26) |
| United Arab Emirates | 9.64 (4.82, 18.49) | 1.57 (0.71, 3.26) |  | 105.94 (43.03, 244.70) | 1.72 (0.68, 4.20) |  | 999.18 | 0.15 (0.01, 0.30) |
| United Kingdom | 215.32 (178.40, 257.44) | 0.26 (0.22, 0.31) |  | 608.12 (499.02, 729.81) | 0.53 (0.44, 0.63) |  | 182.43 | 3.16 (2.95, 3.37) |
| United States of America | 847.51 (734.61, 975.94) | 0.28 (0.24, 0.32) |  | 2742.39 (2215.56, 3353.07) | 0.53 (0.43, 0.65) |  | 223.58 | 2.36 (2.14, 2.57) |
| United States Virgin Islands | 1.08 (0.75, 1.48) | 1.18 (0.82, 1.64) |  | 1.18 (0.80, 1.69) | 0.66 (0.45, 0.93) |  | 8.73 | -2.06 (-2.70, -1.41) |
| Uruguay | 10.70 (7.28, 15.66) | 0.28 (0.19, 0.41) |  | 21.24 (14.61, 30.09) | 0.42 (0.29, 0.59) |  | 98.44 | 1.73 (1.53, 1.93) |
| Uzbekistan | 24.86 (18.18, 33.15) | 0.21 (0.15, 0.29) |  | 367.66 (256.11, 523.24) | 1.50 (1.04, 2.11) |  | 1378.88 | 9.28 (7.95, 10.63) |
| Vanuatu | 3.72 (2.31, 5.71) | 5.17 (3.12, 8.08) |  | 9.15 (5.86, 13.65) | 4.86 (3.15, 7.21) |  | 145.85 | -0.21 (-0.35, -0.07) |
| Venezuela | 108.71 (76.33, 151.26) | 1.05 (0.71, 1.48) |  | 101.44 (63.19, 157.18) | 0.34 (0.22, 0.53) |  | -6.69 | -3.19 (-4.42, -1.94) |
| Viet Nam | 715.45 (487.66, 985.29) | 1.74 (1.19, 2.40) |  | 936.03 (634.24, 1328.78) | 1.00 (0.68, 1.42) |  | 30.83 | -2.22 (-2.50, -1.94) |
| Yemen | 54.96 (32.14, 89.26) | 1.06 (0.62, 1.72) |  | 136.64 (86.19, 207.06) | 0.98 (0.61, 1.51) |  | 148.63 | -0.30 (-0.37, -0.22) |
| Zambia | 22.63 (14.96, 34.60) | 0.71 (0.46, 1.11) |  | 59.81 (40.58, 84.20) | 0.77 (0.52, 1.10) |  | 164.34 | -0.44 (-0.92, 0.03) |
| Zimbabwe | 214.74 (144.49, 365.85) | 4.63 (3.10, 7.81) |  | 377.96 (250.61, 579.81) | 4.49 (2.96, 6.87) |  | 76.00 | -1.35 (-2.07, -0.62) |

ASMR: age-standardized mortality rate; CI: confidence interval; EAPC: estimated annual percentage change; UI: uncertainty interval.


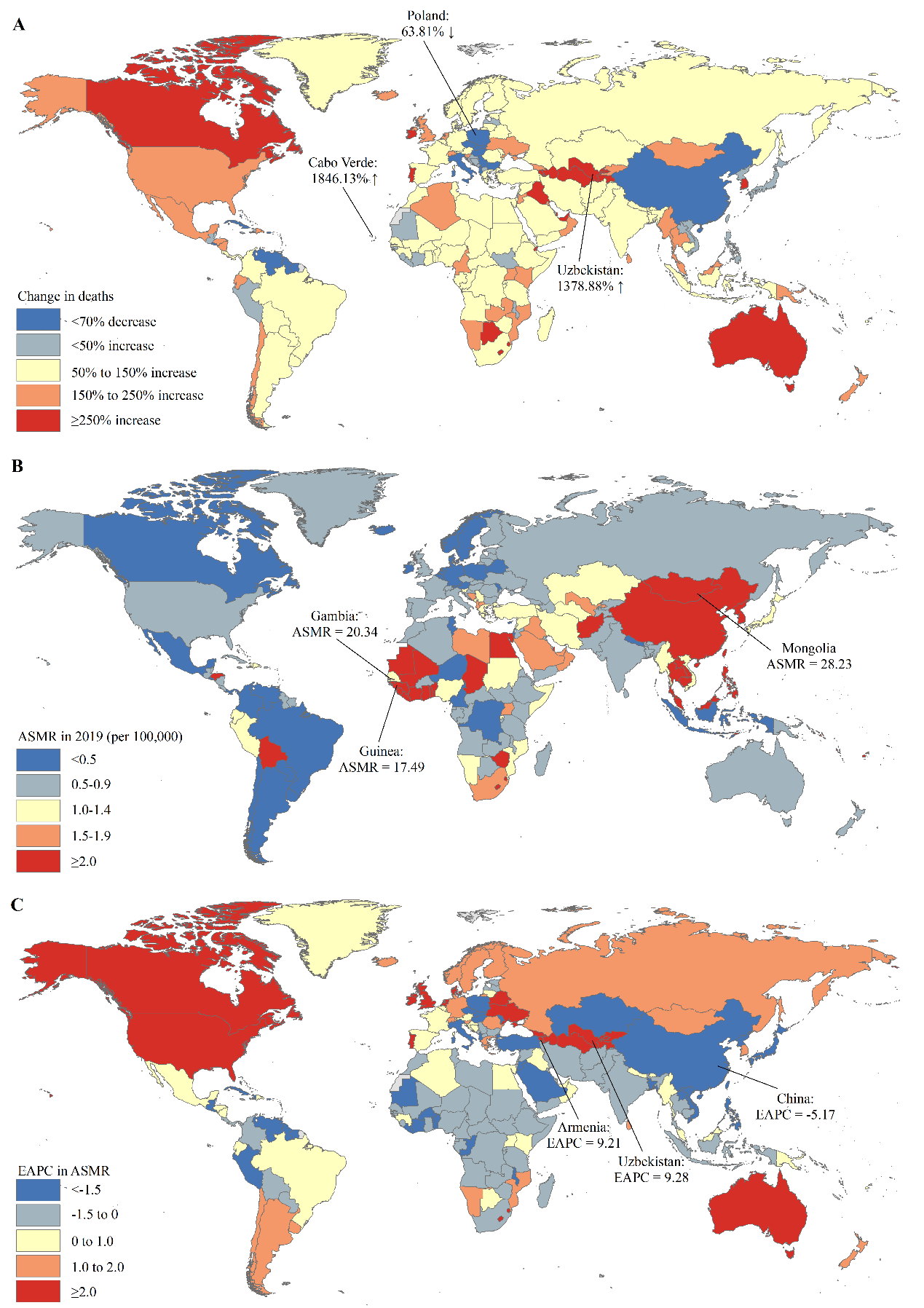


**Figure S6.** The global trends in the mortality of primary liver cancer due to hepatitis B in 204 countries and territories. (A) The percentage change in deaths of primary liver cancer due to hepatitis B between 1990 and 2019; (B) The ASMR of primary liver cancer due to hepatitis B in 2019; (C) The EAPCs in ASMR of primary liver cancer due to hepatitis B from 1990 to 2019.

Note: ASMR: age-standardized mortality rate; EAPC: estimated annual percentage change.

**Table S20. The deaths and ASMR of primary liver cancer due to hepatitis C in 1990 and 2019 and their change trends from 1990 to 2019 at national level.**

| Nation | 1990 | |  | 2019 | |  | 1990-2019 | |
| --- | --- | --- | --- | --- | --- | --- | --- | --- |
|  | Deaths  No. (95% UI) | ASMR per 100,000  No. (95% UI) |  | Deaths  No. x 10^3^ (95% UI) | ASMR per 100,000  No. (95% UI) |  | Percentage change in deaths  No. (%) | EAPC in ASMR  No. (95% CI) |
| Afghanistan | 317.71 (214.35, 442.42) | 4.81 (3.32, 6.52) |  | 451.34 (300.47, 635.30) | 4.13 (2.91, 5.72) |  | 42.06 | -0.61 (-0.73, -0.50) |
| Albania | 66.76 (47.46, 87.67) | 3.62 (2.61, 4.72) |  | 80.62 (51.11, 119.92) | 1.85 (1.19, 2.73) |  | 20.76 | -3.48 (-4.11, -2.85) |
| Algeria | 73.12 (49.65, 99.82) | 0.75 (0.54, 0.97) |  | 256.18 (177.76, 349.93) | 0.89 (0.64, 1.21) |  | 250.36 | 0.64 (0.47, 0.81) |
| American Samoa | 0.26 (0.17, 0.36) | 1.37 (0.90, 1.88) |  | 0.67 (0.44, 0.93) | 1.53 (1.05, 2.08) |  | 159.56 | 0.78 (0.52, 1.03) |
| Andorra | 1.66 (1.04, 2.55) | 3.33 (2.19, 4.98) |  | 5.07 (3.43, 7.07) | 3.51 (2.33, 4.97) |  | 205.53 | 0.29 (0.14, 0.45) |
| Angola | 45.18 (31.43, 61.22) | 1.39 (1.00, 1.84) |  | 114.48 (81.49, 155.20) | 1.26 (0.93, 1.65) |  | 153.39 | -0.55 (-0.64, -0.45) |
| Antigua and Barbuda | 1.09 (0.75, 1.46) | 1.94 (1.34, 2.62) |  | 0.65 (0.45, 0.92) | 0.71 (0.49, 0.98) |  | -39.77 | -3.76 (-4.76, -2.75) |
| Argentina | 163.93 (118.38, 220.63) | 0.53 (0.39, 0.70) |  | 424.62 (312.88, 538.99) | 0.77 (0.57, 0.98) |  | 159.02 | 2.09 (1.85, 2.32) |
| Armenia | 10.64 (7.96, 13.27) | 0.45 (0.34, 0.55) |  | 114.04 (81.42, 150.40) | 2.78 (2.00, 3.62) |  | 971.94 | 9.54 (7.92, 11.20) |
| Australia | 114.56 (83.59, 148.82) | 0.59 (0.43, 0.76) |  | 588.80 (430.55, 763.28) | 1.37 (1.00, 1.78) |  | 413.95 | 3.30 (3.10, 3.49) |
| Austria | 121.05 (88.89, 157.71) | 0.99 (0.73, 1.28) |  | 256.08 (186.57, 342.44) | 1.37 (0.99, 1.85) |  | 111.55 | 1.45 (1.14, 1.76) |
| Azerbaijan | 17.89 (13.44, 22.53) | 0.40 (0.30, 0.50) |  | 128.51 (84.50, 188.07) | 1.72 (1.13, 2.58) |  | 618.27 | 6.14 (3.99, 8.33) |
| Bahamas | 2.51 (1.73, 3.43) | 1.76 (1.23, 2.39) |  | 2.60 (1.72, 3.64) | 0.72 (0.48, 1.00) |  | 3.65 | -3.32 (-4.17, -2.46) |
| Bahrain | 3.00 (2.07, 4.11) | 2.52 (1.82, 3.25) |  | 12.89 (8.75, 17.95) | 2.25 (1.60, 2.96) |  | 328.86 | -1.03 (-1.48, -0.58) |
| Bangladesh | 562.44 (402.88, 760.39) | 1.30 (0.94, 1.75) |  | 1502.11 (1054.62, 1990.51) | 1.24 (0.89, 1.61) |  | 167.07 | -0.36 (-0.51, -0.21) |
| Barbados | 2.16 (1.38, 3.19) | 0.69 (0.45, 1.02) |  | 3.21 (2.16, 4.58) | 0.64 (0.44, 0.91) |  | 48.45 | -0.35 (-0.53, -0.18) |
| Belarus | 47.13 (34.12, 61.27) | 0.37 (0.27, 0.47) |  | 99.42 (64.50, 142.04) | 0.61 (0.40, 0.87) |  | 110.95 | 2.40 (1.88, 2.92) |
| Belgium | 188.25 (143.61, 233.00) | 1.18 (0.90, 1.45) |  | 348.84 (265.05, 437.57) | 1.42 (1.07, 1.81) |  | 85.31 | 0.50 (0.05, 0.95) |
| Belize | 1.50 (1.07, 1.99) | 1.68 (1.19, 2.24) |  | 1.83 (1.23, 2.53) | 0.74 (0.50, 1.02) |  | 22.03 | -2.94 (-3.60, -2.28) |
| Benin | 25.65 (16.41, 36.63) | 1.41 (0.91, 1.98) |  | 44.41 (27.80, 62.92) | 1.08 (0.71, 1.52) |  | 73.15 | -1.11 (-1.24, -0.98) |
| Bermuda | 1.01 (0.69, 1.37) | 1.71 (1.18, 2.30) |  | 0.62 (0.40, 0.88) | 0.45 (0.29, 0.64) |  | -38.68 | -4.66 (-5.72, -3.59) |
| Bhutan | 1.54 (0.90, 2.39) | 0.71 (0.44, 1.10) |  | 5.74 (3.50, 8.81) | 1.12 (0.70, 1.73) |  | 271.65 | 1.81 (1.70, 1.92) |
| Bolivia | 13.10 (7.05, 22.45) | 0.48 (0.27, 0.81) |  | 35.92 (19.91, 59.17) | 0.47 (0.27, 0.75) |  | 174.23 | -0.15 (-0.23, -0.07) |
| Bosnia and Herzegovina | 62.39 (42.51, 83.73) | 1.67 (1.17, 2.20) |  | 150.46 (103.11, 214.02) | 2.50 (1.74, 3.50) |  | 141.16 | 2.01 (1.55, 2.47) |
| Botswana | 1.68 (0.81, 3.31) | 0.35 (0.17, 0.66) |  | 5.36 (3.37, 7.79) | 0.46 (0.30, 0.64) |  | 218.09 | 0.38 (-0.20, 0.98) |
| Brazil | 684.10 (609.43, 762.90) | 0.86 (0.77, 0.96) |  | 2374.49 (2081.78, 2653.04) | 1.04 (0.91, 1.15) |  | 247.10 | 1.17 (0.96, 1.37) |
| Brunei | 2.78 (1.83, 3.91) | 3.86 (2.66, 5.23) |  | 10.45 (7.18, 14.36) | 4.39 (3.24, 5.71) |  | 276.03 | 0.97 (0.42, 1.51) |
| Bulgaria | 190.90 (129.05, 259.53) | 1.55 (1.09, 2.05) |  | 155.31 (102.76, 226.55) | 1.05 (0.69, 1.51) |  | -18.65 | -0.75 (-1.32, -0.17) |
| Burkina Faso | 35.70 (23.90, 49.34) | 0.99 (0.69, 1.33) |  | 51.38 (33.78, 70.88) | 0.68 (0.45, 0.94) |  | 43.92 | -1.47 (-1.69, -1.25) |
| Burundi | 18.80 (11.33, 28.57) | 0.91 (0.57, 1.34) |  | 29.56 (17.80, 45.72) | 0.81 (0.50, 1.22) |  | 57.25 | -0.90 (-1.07, -0.73) |
| Cambodia | 188.25 (130.80, 250.42) | 4.67 (3.32, 6.02) |  | 414.90 (295.64, 549.64) | 3.92 (2.84, 5.08) |  | 120.40 | -0.82 (-0.93, -0.71) |
| Cameroon | 4.92 (2.89, 7.71) | 0.13 (0.08, 0.20) |  | 12.09 (7.46, 17.95) | 0.12 (0.08, 0.18) |  | 145.92 | -0.36 (-0.52, -0.21) |
| Canada | 113.91 (79.64, 155.26) | 0.35 (0.25, 0.48) |  | 521.22 (356.33, 734.10) | 0.73 (0.50, 1.02) |  | 357.57 | 2.92 (2.67, 3.18) |
| Cape Verde | 0.65 (0.44, 0.89) | 0.27 (0.18, 0.37) |  | 10.29 (6.85, 14.46) | 2.56 (1.68, 3.63) |  | 1488.53 | 6.22 (4.03, 8.45) |
| Central African Republic | 17.51 (10.93, 26.17) | 1.72 (1.13, 2.46) |  | 28.98 (17.20, 44.42) | 1.62 (1.01, 2.37) |  | 65.53 | -0.73 (-0.96, -0.50) |
| Chad | 36.99 (23.34, 53.12) | 1.42 (0.91, 2.01) |  | 57.05 (35.58, 83.45) | 1.21 (0.77, 1.72) |  | 54.24 | -0.71 (-0.79, -0.63) |
| Chile | 73.36 (53.27, 93.18) | 0.77 (0.57, 0.98) |  | 266.01 (191.94, 344.22) | 1.10 (0.80, 1.42) |  | 262.59 | 1.84 (1.56, 2.11) |
| China | 37162.99 (30837.15, 45223.29) | 4.99 (4.19, 5.99) |  | 33078.87 (27212.24, 39258.21) | 1.75 (1.45, 2.07) |  | -10.99 | -5.07 (-5.82, -4.31) |
| Colombia | 189.92 (144.13, 234.28) | 1.20 (0.92, 1.47) |  | 541.38 (372.27, 749.57) | 1.02 (0.69, 1.41) |  | 185.06 | -0.42 (-0.86, 0.03) |
| Comoros | 1.76 (0.98, 2.88) | 0.89 (0.52, 1.42) |  | 3.75 (2.33, 5.72) | 0.86 (0.54, 1.31) |  | 112.26 | -0.26 (-0.35, -0.16) |
| Congo | 19.57 (13.17, 27.88) | 2.09 (1.47, 2.88) |  | 33.48 (22.40, 48.61) | 1.55 (1.08, 2.19) |  | 71.11 | -1.37 (-1.52, -1.23) |
| Cook Islands | 0.37 (0.24, 0.51) | 3.24 (2.16, 4.38) |  | 0.59 (0.39, 0.83) | 2.39 (1.61, 3.34) |  | 59.65 | -0.98 (-1.07, -0.90) |
| Costa Rica | 36.64 (27.50, 46.36) | 2.21 (1.66, 2.80) |  | 100.34 (68.32, 137.45) | 1.99 (1.35, 2.72) |  | 173.88 | -0.56 (-1.09, -0.02) |
| Cote d'Ivoire | 43.20 (26.86, 63.69) | 1.39 (0.90, 2.00) |  | 78.72 (47.97, 118.10) | 0.96 (0.62, 1.42) |  | 82.23 | -1.73 (-1.94, -1.51) |
| Croatia | 42.39 (28.80, 58.41) | 0.69 (0.47, 0.93) |  | 61.86 (39.52, 89.08) | 0.67 (0.43, 0.96) |  | 45.93 | 0.17 (-0.42, 0.76) |
| Cuba | 174.90 (124.76, 232.69) | 1.70 (1.20, 2.23) |  | 111.47 (73.92, 157.81) | 0.56 (0.37, 0.80) |  | -36.27 | -4.13 (-5.37, -2.86) |
| Cyprus | 9.85 (6.81, 13.19) | 1.38 (1.00, 1.78) |  | 25.72 (18.61, 33.03) | 1.40 (1.05, 1.79) |  | 161.01 | 0.31 (-0.02, 0.64) |
| Czech Republic | 145.24 (99.92, 194.61) | 1.04 (0.73, 1.38) |  | 150.76 (100.61, 215.62) | 0.69 (0.45, 0.98) |  | 3.80 | -1.61 (-1.82, -1.39) |
| Democratic Republic of the Congo | 177.58 (128.95, 233.34) | 1.33 (1.01, 1.68) |  | 366.01 (256.69, 497.68) | 1.17 (0.85, 1.55) |  | 106.11 | -0.52 (-0.59, -0.44) |
| Denmark | 56.82 (41.79, 73.19) | 0.68 (0.50, 0.86) |  | 136.52 (100.28, 177.48) | 1.15 (0.85, 1.49) |  | 140.26 | 2.21 (1.95, 2.46) |
| Djibouti | 0.95 (0.55, 1.60) | 0.89 (0.55, 1.44) |  | 4.19 (2.43, 7.12) | 0.92 (0.57, 1.47) |  | 340.67 | 0.04 (-0.06, 0.14) |
| Dominica | 1.73 (1.21, 2.35) | 2.28 (1.63, 3.09) |  | 0.72 (0.48, 1.02) | 0.79 (0.52, 1.11) |  | -58.10 | -3.77 (-4.59, -2.94) |
| Dominican Republic | 33.03 (23.26, 44.39) | 1.00 (0.72, 1.33) |  | 97.80 (62.38, 152.25) | 1.12 (0.72, 1.72) |  | 196.13 | 0.70 (0.17, 1.24) |
| Ecuador | 12.72 (8.10, 18.62) | 0.27 (0.17, 0.40) |  | 47.11 (28.64, 70.78) | 0.35 (0.21, 0.52) |  | 270.39 | 1.41 (1.01, 1.82) |
| Egypt | 3360.40 (2517.11, 4136.24) | 11.90 (8.94, 14.63) |  | 8628.54 (5849.16, 12345.07) | 14.05 (9.83, 19.71) |  | 156.77 | 1.48 (1.11, 1.85) |
| El Salvador | 42.56 (32.66, 53.01) | 1.50 (1.15, 1.87) |  | 50.70 (34.56, 70.97) | 0.84 (0.57, 1.18) |  | 19.14 | -2.19 (-2.95, -1.42) |
| Equatorial Guinea | 2.86 (1.92, 4.07) | 1.62 (1.14, 2.20) |  | 6.39 (3.63, 9.90) | 1.61 (0.93, 2.47) |  | 123.06 | -0.11 (-0.34, 0.12) |
| Eritrea | 7.16 (3.80, 12.51) | 0.92 (0.53, 1.53) |  | 19.90 (12.09, 29.84) | 0.98 (0.62, 1.44) |  | 177.81 | 0.01 (-0.15, 0.16) |
| Estonia | 12.16 (8.69, 16.06) | 0.59 (0.42, 0.77) |  | 26.04 (16.91, 36.43) | 0.91 (0.59, 1.29) |  | 114.12 | 0.61 (0.02, 1.20) |
| Eswatini | 4.70 (2.83, 8.20) | 1.88 (1.16, 3.20) |  | 22.75 (7.67, 42.85) | 4.46 (1.64, 8.17) |  | 384.05 | 3.97 (2.94, 5.02) |
| Ethiopia | 200.21 (135.63, 283.79) | 1.21 (0.86, 1.65) |  | 422.19 (327.62, 537.43) | 1.21 (0.95, 1.55) |  | 110.87 | -0.18 (-0.27, -0.08) |
| Federated States of Micronesia | 1.10 (0.66, 1.70) | 2.77 (1.72, 4.09) |  | 1.47 (0.83, 2.38) | 2.53 (1.54, 3.79) |  | 33.28 | -0.37 (-0.51, -0.22) |
| Fiji | 4.38 (2.80, 6.32) | 1.48 (0.98, 2.11) |  | 9.63 (6.13, 14.19) | 1.50 (1.02, 2.14) |  | 119.69 | 0.47 (0.15, 0.78) |
| Finland | 80.56 (60.19, 102.02) | 1.10 (0.83, 1.38) |  | 202.86 (151.93, 257.28) | 1.52 (1.13, 1.94) |  | 151.81 | 1.40 (1.29, 1.50) |
| France | 1604.56 (1201.78, 2056.26) | 1.92 (1.44, 2.50) |  | 3503.58 (2648.58, 4375.37) | 2.45 (1.84, 3.11) |  | 118.35 | 0.60 (0.30, 0.90) |
| Gabon | 9.47 (6.53, 12.74) | 1.84 (1.31, 2.42) |  | 16.12 (10.23, 23.77) | 1.77 (1.15, 2.59) |  | 70.21 | -0.37 (-0.51, -0.24) |
| Gambia | 13.76 (8.45, 20.26) | 4.46 (2.85, 6.40) |  | 52.89 (32.77, 77.60) | 6.04 (3.83, 8.89) |  | 284.27 | 0.80 (0.62, 0.97) |
| Georgia | 26.35 (19.65, 33.65) | 0.46 (0.35, 0.58) |  | 71.25 (51.49, 94.95) | 1.16 (0.83, 1.54) |  | 170.41 | 2.38 (1.17, 3.61) |
| Germany | 978.23 (754.99, 1219.53) | 0.75 (0.58, 0.93) |  | 2413.11 (1783.90, 3079.27) | 1.17 (0.87, 1.49) |  | 146.68 | 1.65 (1.20, 2.11) |
| Ghana | 60.35 (38.12, 87.97) | 1.16 (0.75, 1.66) |  | 137.66 (86.00, 203.60) | 1.02 (0.66, 1.46) |  | 128.09 | -0.62 (-0.75, -0.49) |
| Greece | 69.33 (47.94, 94.20) | 0.46 (0.33, 0.61) |  | 165.61 (115.30, 226.59) | 0.59 (0.41, 0.80) |  | 138.88 | 1.07 (0.96, 1.19) |
| Greenland | 0.41 (0.28, 0.57) | 1.26 (0.89, 1.71) |  | 1.18 (0.76, 1.72) | 1.74 (1.15, 2.52) |  | 185.67 | 0.90 (0.68, 1.12) |
| Grenada | 1.80 (1.28, 2.41) | 2.32 (1.62, 3.12) |  | 0.74 (0.50, 1.04) | 0.73 (0.50, 1.00) |  | -58.62 | -4.19 (-5.19, -3.18) |
| Guam | 0.54 (0.35, 0.77) | 0.92 (0.63, 1.27) |  | 1.81 (1.18, 2.66) | 0.96 (0.63, 1.40) |  | 236.09 | 0.41 (0.21, 0.62) |
| Guatemala | 140.86 (106.38, 177.95) | 4.50 (3.46, 5.61) |  | 218.54 (156.63, 294.47) | 2.13 (1.55, 2.85) |  | 55.15 | -3.49 (-5.02, -1.94) |
| Guinea | 179.42 (118.22, 254.35) | 6.00 (3.94, 8.41) |  | 295.54 (182.09, 436.37) | 6.04 (3.67, 8.90) |  | 64.73 | 0.08 (0.04, 0.12) |
| Guinea-Bissau | 5.71 (3.33, 9.15) | 1.62 (0.99, 2.50) |  | 7.99 (4.74, 12.44) | 1.36 (0.84, 2.09) |  | 39.94 | -0.72 (-0.79, -0.65) |
| Guyana | 5.46 (3.76, 7.69) | 1.62 (1.12, 2.23) |  | 4.14 (2.60, 6.01) | 0.75 (0.49, 1.06) |  | -24.26 | -2.73 (-3.57, -1.89) |
| Haiti | 51.86 (28.63, 84.41) | 1.90 (1.09, 3.14) |  | 64.99 (35.27, 105.83) | 1.10 (0.60, 1.73) |  | 25.30 | -1.97 (-2.20, -1.75) |
| Honduras | 93.68 (31.32, 147.65) | 4.99 (1.63, 7.85) |  | 375.55 (154.23, 604.65) | 6.82 (2.82, 10.85) |  | 300.86 | 1.39 (1.17, 1.61) |
| Hungary | 241.35 (168.49, 321.80) | 1.65 (1.16, 2.16) |  | 130.85 (87.56, 183.81) | 0.64 (0.43, 0.90) |  | -45.78 | -2.54 (-3.36, -1.72) |
| Iceland | 2.15 (1.62, 2.72) | 0.74 (0.56, 0.93) |  | 5.96 (4.40, 7.73) | 1.03 (0.76, 1.35) |  | 177.71 | 1.10 (0.93, 1.28) |
| India | 2030.98 (1592.11, 2530.70) | 0.57 (0.45, 0.70) |  | 6291.88 (4886.73, 7982.91) | 0.61 (0.47, 0.77) |  | 209.80 | 0.16 (0.02, 0.30) |
| Indonesia | 1072.48 (902.26, 1289.15) | 1.29 (1.08, 1.54) |  | 2162.77 (1784.26, 2520.51) | 1.22 (1.02, 1.39) |  | 101.66 | -0.32 (-0.41, -0.22) |
| Iran | 271.70 (209.39, 355.07) | 1.42 (1.08, 1.86) |  | 719.87 (607.18, 839.92) | 1.12 (0.94, 1.31) |  | 164.95 | -1.19 (-1.88, -0.50) |
| Iraq | 127.44 (88.14, 174.52) | 1.80 (1.27, 2.42) |  | 479.53 (325.90, 666.43) | 2.43 (1.70, 3.31) |  | 276.29 | 1.35 (0.88, 1.83) |
| Ireland | 23.83 (17.96, 29.97) | 0.58 (0.44, 0.72) |  | 101.10 (75.76, 129.39) | 1.31 (0.99, 1.68) |  | 324.27 | 3.65 (3.31, 4.00) |
| Israel | 61.09 (46.66, 76.32) | 1.26 (0.97, 1.55) |  | 149.43 (116.32, 185.31) | 1.25 (0.97, 1.56) |  | 144.60 | -0.08 (-0.20, 0.05) |
| Italy | 3121.17 (2853.06, 3391.49) | 3.43 (3.13, 3.73) |  | 4032.14 (3535.40, 4451.72) | 2.66 (2.34, 2.93) |  | 29.19 | -1.38 (-1.64, -1.13) |
| Jamaica | 18.13 (12.65, 23.89) | 0.99 (0.70, 1.30) |  | 19.79 (13.21, 28.25) | 0.65 (0.43, 0.94) |  | 9.18 | -0.86 (-1.77, 0.06) |
| Japan | 13059.85 (12272.80, 13759.61) | 7.63 (7.16, 8.03) |  | 25051.50 (21085.76, 27539.73) | 6.08 (5.30, 6.63) |  | 91.82 | -1.64 (-2.23, -1.04) |
| Jordan | 12.76 (8.34, 17.73) | 1.22 (0.80, 1.68) |  | 45.62 (31.07, 62.43) | 0.88 (0.61, 1.19) |  | 257.61 | -1.34 (-1.48, -1.20) |
| Kazakhstan | 147.44 (107.70, 188.27) | 1.23 (0.90, 1.55) |  | 372.27 (258.94, 501.91) | 2.30 (1.62, 3.05) |  | 152.49 | -0.65 (-1.68, 0.39) |
| Kenya | 46.95 (32.13, 79.87) | 0.66 (0.46, 1.13) |  | 154.57 (102.31, 227.42) | 0.86 (0.58, 1.23) |  | 229.19 | 0.49 (0.17, 0.80) |
| Kiribati | 1.09 (0.71, 1.58) | 3.36 (2.28, 4.69) |  | 1.73 (1.09, 2.52) | 3.00 (1.93, 4.33) |  | 58.89 | -0.36 (-0.43, -0.30) |
| Kuwait | 4.81 (3.42, 6.23) | 0.98 (0.70, 1.25) |  | 17.71 (12.29, 23.96) | 0.89 (0.63, 1.20) |  | 268.48 | 0.41 (0.08, 0.74) |
| Kyrgyzstan | 15.05 (11.24, 18.94) | 0.52 (0.39, 0.65) |  | 42.44 (30.57, 54.53) | 1.06 (0.77, 1.37) |  | 181.98 | 3.33 (2.96, 3.69) |
| Lao | 55.23 (31.75, 86.03) | 2.98 (1.78, 4.46) |  | 73.60 (46.82, 103.53) | 1.98 (1.31, 2.75) |  | 33.27 | -1.71 (-1.85, -1.57) |
| Latvia | 17.14 (12.17, 22.57) | 0.47 (0.34, 0.62) |  | 29.63 (20.34, 40.76) | 0.69 (0.46, 0.95) |  | 72.92 | 0.54 (-0.23, 1.32) |
| Lebanon | 16.98 (10.68, 24.92) | 0.85 (0.56, 1.21) |  | 36.29 (22.56, 57.67) | 0.70 (0.44, 1.11) |  | 113.73 | -0.79 (-0.87, -0.71) |
| Lesotho | 15.36 (8.94, 28.00) | 1.76 (1.05, 3.13) |  | 44.29 (20.25, 74.09) | 3.97 (1.91, 6.49) |  | 188.33 | 3.34 (2.82, 3.87) |
| Liberia | 13.95 (8.73, 20.12) | 1.39 (0.91, 1.94) |  | 18.25 (10.99, 27.62) | 1.09 (0.67, 1.64) |  | 30.87 | -1.14 (-1.28, -1.00) |
| Libya | 32.94 (21.75, 48.11) | 1.96 (1.29, 2.83) |  | 84.25 (55.49, 123.18) | 1.83 (1.21, 2.66) |  | 155.78 | -0.17 (-0.31, -0.02) |
| Lithuania | 21.39 (15.26, 27.68) | 0.47 (0.34, 0.60) |  | 45.00 (29.52, 61.59) | 0.73 (0.48, 1.00) |  | 110.39 | 0.85 (0.47, 1.22) |
| Luxembourg | 5.95 (4.40, 7.76) | 1.07 (0.80, 1.38) |  | 14.38 (10.29, 19.65) | 1.37 (0.98, 1.88) |  | 141.57 | 0.96 (0.71, 1.21) |
| Macedonia | 44.17 (30.53, 59.65) | 2.52 (1.77, 3.34) |  | 77.64 (50.74, 111.82) | 2.55 (1.73, 3.55) |  | 75.78 | -0.00 (-0.12, 0.12) |
| Madagascar | 32.70 (20.21, 52.22) | 0.73 (0.45, 1.17) |  | 59.44 (36.21, 91.16) | 0.69 (0.45, 1.05) |  | 81.77 | -0.38 (-0.49, -0.27) |
| Malawi | 26.77 (16.68, 40.78) | 0.81 (0.52, 1.18) |  | 45.43 (29.63, 63.19) | 0.77 (0.52, 1.07) |  | 69.70 | -0.86 (-1.19, -0.54) |
| Malaysia | 86.11 (55.17, 122.70) | 1.07 (0.69, 1.49) |  | 263.88 (169.00, 395.39) | 1.12 (0.73, 1.65) |  | 206.44 | 0.31 (-0.09, 0.71) |
| Maldives | 1.34 (0.74, 2.38) | 2.00 (1.15, 3.25) |  | 3.99 (2.66, 5.47) | 1.58 (1.04, 2.20) |  | 196.97 | -0.71 (-0.83, -0.58) |
| Mali | 218.23 (147.13, 302.38) | 5.50 (3.82, 7.37) |  | 425.77 (276.70, 606.06) | 5.28 (3.56, 7.22) |  | 95.10 | -0.34 (-0.45, -0.23) |
| Malta | 2.86 (2.14, 3.64) | 0.68 (0.51, 0.86) |  | 8.29 (6.08, 10.73) | 0.85 (0.63, 1.10) |  | 190.17 | 0.85 (0.61, 1.10) |
| Marshall Islands | 0.40 (0.25, 0.61) | 2.88 (1.84, 4.30) |  | 0.68 (0.40, 1.04) | 2.47 (1.52, 3.65) |  | 70.97 | -0.59 (-0.67, -0.52) |
| Mauritania | 13.70 (8.43, 19.68) | 1.51 (0.96, 2.13) |  | 17.21 (10.79, 24.86) | 0.94 (0.61, 1.36) |  | 25.60 | -1.64 (-1.77, -1.51) |
| Mauritius | 3.58 (2.53, 4.67) | 0.55 (0.39, 0.70) |  | 9.56 (6.32, 13.80) | 0.59 (0.39, 0.83) |  | 167.13 | 1.42 (1.06, 1.78) |
| Mexico | 417.48 (375.72, 458.40) | 1.10 (0.99, 1.20) |  | 1735.73 (1459.89, 2059.87) | 1.56 (1.31, 1.86) |  | 315.76 | 1.26 (1.08, 1.45) |
| Moldova | 15.03 (10.52, 20.50) | 0.36 (0.26, 0.49) |  | 33.22 (22.59, 46.35) | 0.57 (0.39, 0.79) |  | 121.10 | -0.14 (-1.64, 1.38) |
| Monaco | 0.93 (0.63, 1.26) | 1.23 (0.84, 1.71) |  | 2.99 (2.12, 3.96) | 2.93 (2.06, 3.93) |  | 221.66 | 3.73 (2.86, 4.61) |
| Mongolia | 215.42 (145.57, 299.12) | 21.95 (15.12, 29.98) |  | 697.32 (473.07, 971.29) | 40.31 (28.58, 53.28) |  | 223.70 | 3.19 (2.54, 3.85) |
| Montenegro | 9.10 (6.13, 12.57) | 1.51 (1.02, 2.07) |  | 15.31 (10.44, 21.82) | 1.55 (1.07, 2.18) |  | 68.20 | -0.02 (-0.27, 0.23) |
| Morocco | 88.39 (57.27, 122.41) | 0.79 (0.51, 1.07) |  | 231.09 (154.00, 314.38) | 0.88 (0.60, 1.19) |  | 161.45 | 0.26 (-0.02, 0.53) |
| Mozambique | 21.85 (12.97, 34.61) | 0.48 (0.29, 0.76) |  | 60.61 (36.45, 90.27) | 0.72 (0.45, 1.07) |  | 177.32 | 1.34 (1.18, 1.50) |
| Myanmar | 197.37 (121.72, 304.68) | 1.02 (0.66, 1.51) |  | 593.99 (408.55, 794.14) | 1.46 (1.02, 1.93) |  | 200.95 | 1.16 (0.96, 1.37) |
| Namibia | 4.39 (2.69, 7.31) | 0.67 (0.42, 1.09) |  | 13.37 (8.80, 18.51) | 1.05 (0.71, 1.44) |  | 204.33 | 1.52 (1.24, 1.81) |
| Nauru | 0.07 (0.05, 0.11) | 2.53 (1.67, 3.58) |  | 0.07 (0.04, 0.11) | 2.18 (1.38, 3.28) |  | -8.87 | -0.63 (-0.91, -0.35) |
| Nepal | 52.59 (34.02, 75.99) | 0.69 (0.46, 0.97) |  | 164.17 (109.32, 253.13) | 0.87 (0.58, 1.30) |  | 212.14 | 0.79 (0.65, 0.94) |
| Netherlands | 93.63 (69.65, 119.71) | 0.46 (0.35, 0.59) |  | 329.58 (243.83, 417.96) | 0.92 (0.69, 1.17) |  | 252.02 | 2.66 (2.54, 2.79) |
| New Zealand | 22.78 (19.75, 26.43) | 0.59 (0.51, 0.68) |  | 87.97 (75.58, 101.23) | 1.11 (0.95, 1.27) |  | 286.19 | 2.36 (2.20, 2.52) |
| Nicaragua | 18.82 (13.71, 24.04) | 1.34 (0.99, 1.71) |  | 67.80 (49.29, 89.80) | 1.72 (1.27, 2.25) |  | 260.19 | 0.98 (0.68, 1.29) |
| Niger | 3.36 (2.14, 4.94) | 0.15 (0.10, 0.22) |  | 9.13 (5.59, 13.39) | 0.14 (0.09, 0.20) |  | 171.63 | -0.28 (-0.37, -0.20) |
| Nigeria | 334.27 (240.71, 449.13) | 0.88 (0.64, 1.18) |  | 607.66 (459.14, 781.49) | 0.86 (0.66, 1.07) |  | 81.79 | -0.01 (-0.10, 0.09) |
| Niue | 0.05 (0.03, 0.06) | 1.99 (1.33, 2.81) |  | 0.04 (0.02, 0.05) | 1.66 (1.09, 2.40) |  | -21.69 | -0.69 (-0.72, -0.65) |
| North Korea | 495.01 (311.81, 713.23) | 3.49 (2.26, 4.87) |  | 756.33 (491.78, 1071.34) | 2.44 (1.60, 3.41) |  | 52.79 | -1.45 (-1.54, -1.36) |
| Northern Mariana Islands | 0.22 (0.14, 0.31) | 1.64 (1.08, 2.25) |  | 0.70 (0.43, 1.01) | 1.54 (1.00, 2.13) |  | 224.01 | -0.03 (-0.21, 0.16) |
| Norway | 44.08 (39.21, 48.95) | 0.62 (0.56, 0.70) |  | 98.32 (84.16, 112.69) | 0.99 (0.85, 1.13) |  | 123.04 | 2.03 (1.86, 2.19) |
| Oman | 9.62 (6.02, 14.21) | 1.73 (1.10, 2.47) |  | 23.44 (15.82, 32.25) | 1.76 (1.23, 2.30) |  | 143.54 | 0.88 (0.51, 1.25) |
| Pakistan | 1094.49 (704.58, 1511.31) | 2.01 (1.28, 2.79) |  | 1940.80 (1485.60, 2482.79) | 1.94 (1.48, 2.50) |  | 77.32 | -0.16 (-0.31, -0.01) |
| Palau | 0.15 (0.09, 0.23) | 1.74 (1.06, 2.60) |  | 0.32 (0.20, 0.47) | 1.69 (1.11, 2.38) |  | 112.01 | 0.01 (-0.05, 0.07) |
| Palestine | 30.54 (20.74, 42.53) | 3.99 (2.75, 5.47) |  | 54.69 (39.50, 71.18) | 2.89 (2.13, 3.75) |  | 79.08 | -1.05 (-1.13, -0.98) |
| Panama | 19.19 (14.50, 24.22) | 1.34 (1.01, 1.69) |  | 46.66 (31.85, 65.40) | 1.13 (0.77, 1.59) |  | 143.19 | -0.23 (-0.62, 0.16) |
| Papua New Guinea | 6.11 (3.80, 8.84) | 0.47 (0.30, 0.66) |  | 16.30 (10.37, 24.08) | 0.51 (0.34, 0.73) |  | 166.90 | 0.35 (0.31, 0.38) |
| Paraguay | 20.01 (14.58, 25.64) | 0.96 (0.71, 1.24) |  | 44.38 (30.38, 60.93) | 0.85 (0.58, 1.17) |  | 121.81 | -0.22 (-0.78, 0.35) |
| Peru | 49.60 (30.36, 73.52) | 0.46 (0.28, 0.68) |  | 69.28 (42.18, 107.40) | 0.22 (0.13, 0.34) |  | 39.68 | -3.39 (-3.97, -2.81) |
| Philippines | 579.67 (423.69, 734.93) | 2.33 (1.74, 2.92) |  | 1079.49 (857.89, 1340.78) | 1.56 (1.26, 1.92) |  | 86.23 | -1.61 (-1.92, -1.29) |
| Poland | 964.75 (839.37, 1089.46) | 2.31 (2.03, 2.59) |  | 376.19 (296.74, 460.26) | 0.51 (0.41, 0.62) |  | -61.01 | -5.02 (-6.43, -3.60) |
| Portugal | 99.15 (75.43, 126.31) | 0.72 (0.56, 0.91) |  | 404.36 (301.70, 512.15) | 1.63 (1.21, 2.09) |  | 307.81 | 2.64 (2.29, 2.98) |
| Puerto Rico | 59.99 (41.48, 80.33) | 1.66 (1.15, 2.20) |  | 47.79 (30.98, 68.65) | 0.62 (0.40, 0.91) |  | -20.34 | -2.40 (-3.24, -1.55) |
| Qatar | 4.38 (2.83, 6.44) | 6.94 (4.47, 9.83) |  | 25.95 (15.69, 38.16) | 6.48 (4.41, 9.04) |  | 492.81 | 0.04 (-0.21, 0.30) |
| Romania | 127.73 (89.89, 171.86) | 0.48 (0.35, 0.64) |  | 283.76 (192.93, 393.65) | 0.74 (0.50, 1.03) |  | 122.15 | 2.17 (1.74, 2.59) |
| Russia | 823.22 (707.70, 937.01) | 0.47 (0.41, 0.53) |  | 1913.29 (1585.14, 2318.48) | 0.80 (0.66, 0.97) |  | 132.41 | 2.39 (2.08, 2.69) |
| Rwanda | 31.63 (18.80, 50.54) | 1.28 (0.79, 1.93) |  | 59.05 (39.86, 83.91) | 1.19 (0.82, 1.67) |  | 86.69 | -0.77 (-1.00, -0.54) |
| Saint Kitts and Nevis | 1.29 (0.88, 1.75) | 3.43 (2.46, 4.50) |  | 0.53 (0.35, 0.76) | 0.93 (0.62, 1.28) |  | -59.06 | -4.40 (-5.42, -3.37) |
| Saint Lucia | 1.28 (0.90, 1.72) | 1.55 (1.11, 2.05) |  | 1.05 (0.71, 1.48) | 0.51 (0.35, 0.72) |  | -17.64 | -4.22 (-5.34, -3.10) |
| Saint Vincent and the Grenadines | 1.33 (0.94, 1.78) | 1.90 (1.36, 2.50) |  | 0.88 (0.60, 1.21) | 0.67 (0.46, 0.93) |  | -34.25 | -3.38 (-4.34, -2.41) |
| Samoa | 1.05 (0.67, 1.55) | 1.29 (0.84, 1.86) |  | 1.45 (0.92, 2.12) | 1.07 (0.69, 1.55) |  | 38.55 | -0.62 (-0.74, -0.50) |
| San Marino | 0.26 (0.18, 0.35) | 0.77 (0.54, 1.02) |  | 0.69 (0.43, 1.04) | 1.00 (0.61, 1.53) |  | 168.40 | 1.48 (1.23, 1.73) |
| Sao Tome and Principe | 0.31 (0.20, 0.43) | 0.53 (0.36, 0.73) |  | 0.47 (0.28, 0.73) | 0.53 (0.32, 0.82) |  | 53.20 | 0.00 (-0.13, 0.13) |
| Saudi Arabia | 112.59 (72.40, 160.11) | 2.31 (1.48, 3.26) |  | 245.99 (162.78, 345.17) | 1.97 (1.36, 2.71) |  | 118.48 | -0.72 (-1.02, -0.42) |
| Senegal | 6.64 (4.01, 9.88) | 0.24 (0.15, 0.35) |  | 14.70 (8.69, 22.69) | 0.23 (0.14, 0.35) |  | 121.49 | 0.06 (-0.08, 0.20) |
| Serbia | 224.17 (155.09, 309.39) | 2.09 (1.48, 2.84) |  | 304.99 (209.45, 426.82) | 1.87 (1.30, 2.56) |  | 36.05 | -0.57 (-0.84, -0.31) |
| Seychelles | 1.18 (0.83, 1.59) | 2.10 (1.48, 2.83) |  | 1.45 (0.99, 1.98) | 1.44 (1.00, 1.93) |  | 22.19 | -1.10 (-1.37, -0.83) |
| Sierra Leone | 21.55 (13.59, 31.48) | 1.23 (0.79, 1.80) |  | 32.04 (19.82, 46.58) | 1.05 (0.66, 1.51) |  | 48.71 | -0.59 (-0.64, -0.54) |
| Singapore | 40.62 (27.44, 55.30) | 2.08 (1.43, 2.80) |  | 193.49 (129.11, 259.96) | 2.65 (1.77, 3.55) |  | 376.34 | 0.87 (0.56, 1.19) |
| Slovakia | 64.28 (44.91, 86.33) | 1.07 (0.76, 1.44) |  | 76.85 (49.73, 110.60) | 0.82 (0.54, 1.18) |  | 19.55 | -1.43 (-1.78, -1.07) |
| Slovenia | 19.44 (12.73, 28.67) | 0.79 (0.52, 1.16) |  | 61.35 (40.02, 86.55) | 1.33 (0.86, 1.90) |  | 215.54 | 2.17 (1.85, 2.50) |
| Solomon Islands | 1.70 (0.99, 2.60) | 1.44 (0.87, 2.08) |  | 3.17 (2.02, 4.51) | 1.27 (0.86, 1.75) |  | 86.49 | -0.41 (-0.49, -0.33) |
| Somalia | 19.48 (10.37, 33.55) | 0.96 (0.55, 1.58) |  | 50.92 (28.62, 88.66) | 0.99 (0.59, 1.76) |  | 161.40 | 0.24 (0.16, 0.31) |
| South Africa | 343.27 (221.84, 533.05) | 1.80 (1.15, 2.79) |  | 683.72 (579.61, 823.63) | 1.70 (1.45, 2.04) |  | 99.18 | -0.60 (-1.16, -0.03) |
| South Korea | 487.47 (325.83, 692.81) | 1.98 (1.35, 2.75) |  | 2707.17 (1879.05, 3573.81) | 3.08 (2.18, 4.04) |  | 455.35 | 2.07 (0.93, 3.22) |
| South Sudan | 17.27 (9.82, 29.45) | 0.83 (0.49, 1.41) |  | 24.43 (13.28, 42.00) | 0.78 (0.44, 1.30) |  | 41.45 | -0.18 (-0.23, -0.13) |
| Spain | 1102.49 (879.51, 1340.67) | 1.99 (1.60, 2.42) |  | 2735.49 (2182.35, 3275.18) | 2.70 (2.10, 3.30) |  | 148.12 | 0.91 (0.62, 1.20) |
| Sri Lanka | 54.30 (36.62, 73.43) | 0.61 (0.43, 0.82) |  | 180.62 (116.13, 266.48) | 0.77 (0.50, 1.12) |  | 232.61 | 1.82 (1.49, 2.15) |
| Sudan | 102.26 (55.56, 158.60) | 1.26 (0.69, 1.94) |  | 193.76 (110.80, 312.73) | 1.24 (0.72, 1.96) |  | 89.48 | -0.09 (-0.21, 0.03) |
| Suriname | 3.91 (2.74, 5.24) | 1.61 (1.14, 2.17) |  | 3.58 (2.39, 5.06) | 0.63 (0.43, 0.90) |  | -8.50 | -3.13 (-3.93, -2.31) |
| Sweden | 157.35 (134.29, 182.44) | 1.00 (0.86, 1.16) |  | 236.72 (198.07, 276.79) | 1.08 (0.91, 1.27) |  | 50.44 | 0.82 (0.20, 1.45) |
| Switzerland | 84.41 (62.38, 109.23) | 0.78 (0.57, 1.02) |  | 288.77 (211.17, 377.33) | 1.58 (1.15, 2.08) |  | 242.13 | 2.03 (1.48, 2.58) |
| Syrian Arab Republic | 89.42 (58.81, 126.03) | 2.02 (1.31, 2.82) |  | 179.09 (121.16, 252.18) | 1.75 (1.22, 2.40) |  | 100.28 | -0.61 (-0.77, -0.45) |
| Taiwan (Province of China) | 502.02 (362.70, 641.90) | 3.26 (2.40, 4.08) |  | 1042.64 (721.11, 1425.43) | 2.60 (1.81, 3.56) |  | 107.69 | -1.63 (-2.66, -0.59) |
| Tajikistan | 13.70 (10.87, 16.80) | 0.52 (0.41, 0.63) |  | 79.12 (57.81, 105.13) | 2.10 (1.57, 2.75) |  | 477.45 | 5.64 (5.33, 5.94) |
| Tanzania | 49.09 (32.83, 67.65) | 0.55 (0.38, 0.74) |  | 125.52 (84.25, 172.22) | 0.62 (0.42, 0.84) |  | 155.67 | 0.33 (0.21, 0.46) |
| Thailand | 1296.26 (873.88, 1802.02) | 4.20 (2.91, 5.70) |  | 4318.85 (2728.57, 6415.68) | 4.30 (2.73, 6.36) |  | 233.18 | 0.38 (0.21, 0.56) |
| Timor-Leste | 5.28 (3.18, 8.17) | 2.30 (1.44, 3.46) |  | 14.07 (8.92, 20.99) | 1.94 (1.27, 2.81) |  | 166.52 | -0.74 (-0.91, -0.57) |
| Togo | 14.61 (9.44, 20.65) | 1.40 (0.94, 1.95) |  | 32.96 (20.58, 47.27) | 1.13 (0.74, 1.60) |  | 125.60 | -1.04 (-1.16, -0.93) |
| Tokelau | 0.03 (0.02, 0.04) | 2.24 (1.44, 3.15) |  | 0.02 (0.01, 0.03) | 1.78 (1.18, 2.53) |  | -25.77 | -0.89 (-0.93, -0.84) |
| Tonga | 2.60 (1.62, 3.91) | 5.07 (3.22, 7.50) |  | 3.99 (2.56, 5.81) | 5.15 (3.28, 7.50) |  | 53.76 | 0.06 (-0.08, 0.19) |
| Trinidad and Tobago | 13.86 (9.63, 18.43) | 1.79 (1.27, 2.34) |  | 11.39 (7.29, 16.67) | 0.63 (0.40, 0.92) |  | -17.84 | -3.77 (-4.83, -2.69) |
| Tunisia | 43.36 (29.67, 58.71) | 0.97 (0.67, 1.28) |  | 106.35 (70.76, 155.50) | 0.89 (0.60, 1.30) |  | 145.30 | -0.12 (-0.20, -0.05) |
| Turkey | 332.32 (216.46, 465.67) | 1.05 (0.69, 1.44) |  | 707.21 (477.48, 947.33) | 0.86 (0.58, 1.14) |  | 112.81 | -0.63 (-0.93, -0.34) |
| Turkmenistan | 8.28 (6.20, 10.32) | 0.49 (0.38, 0.60) |  | 68.07 (44.77, 95.00) | 1.79 (1.19, 2.44) |  | 721.92 | 5.14 (4.18, 6.11) |
| Tuvalu | 0.16 (0.10, 0.25) | 2.68 (1.72, 3.98) |  | 0.19 (0.12, 0.28) | 2.07 (1.30, 3.00) |  | 17.99 | -1.06 (-1.20, -0.91) |
| Uganda | 57.09 (36.98, 81.18) | 0.99 (0.66, 1.38) |  | 176.23 (115.77, 249.59) | 1.46 (0.98, 2.04) |  | 208.66 | 1.50 (1.29, 1.71) |
| Ukraine | 225.07 (196.04, 255.91) | 0.32 (0.28, 0.36) |  | 572.81 (466.88, 690.17) | 0.73 (0.60, 0.89) |  | 154.51 | 3.40 (2.96, 3.84) |
| United Arab Emirates | 4.68 (2.31, 9.39) | 1.70 (0.79, 3.45) |  | 43.25 (15.95, 106.87) | 1.70 (0.67, 4.26) |  | 823.27 | -0.06 (-0.45, 0.32) |
| United Kingdom | 664.35 (589.92, 738.66) | 0.72 (0.64, 0.79) |  | 2086.86 (1830.65, 2334.86) | 1.55 (1.36, 1.73) |  | 214.12 | 3.40 (3.18, 3.63) |
| United States of America | 2436.39 (2191.65, 2665.78) | 0.76 (0.68, 0.83) |  | 9231.35 (7939.38, 10567.02) | 1.63 (1.40, 1.87) |  | 278.89 | 2.77 (2.56, 2.99) |
| United States Virgin Islands | 0.79 (0.53, 1.11) | 1.05 (0.72, 1.46) |  | 1.07 (0.70, 1.48) | 0.58 (0.39, 0.79) |  | 35.37 | -2.15 (-2.72, -1.58) |
| Uruguay | 20.77 (15.05, 26.70) | 0.52 (0.38, 0.67) |  | 48.75 (35.50, 62.46) | 0.85 (0.61, 1.10) |  | 134.69 | 2.09 (1.93, 2.24) |
| Uzbekistan | 38.88 (29.74, 48.22) | 0.38 (0.29, 0.47) |  | 417.45 (280.45, 572.53) | 2.48 (1.80, 3.26) |  | 973.59 | 8.80 (7.70, 9.91) |
| Vanuatu | 1.22 (0.69, 2.04) | 2.22 (1.28, 3.66) |  | 3.19 (1.82, 4.84) | 2.12 (1.27, 3.15) |  | 160.99 | -0.13 (-0.24, -0.02) |
| Venezuela | 230.61 (173.12, 287.97) | 2.58 (1.95, 3.23) |  | 248.57 (169.15, 345.66) | 0.90 (0.62, 1.25) |  | 7.79 | -2.97 (-4.25, -1.67) |
| Viet Nam | 383.04 (213.29, 594.55) | 1.03 (0.60, 1.60) |  | 579.20 (333.18, 896.58) | 0.72 (0.43, 1.11) |  | 51.21 | -1.35 (-1.52, -1.18) |
| Yemen | 67.96 (39.83, 107.78) | 1.66 (0.98, 2.60) |  | 184.21 (123.30, 264.17) | 1.66 (1.13, 2.33) |  | 171.05 | 0.05 (-0.05, 0.15) |
| Zambia | 14.51 (9.18, 22.05) | 0.64 (0.41, 0.97) |  | 38.90 (25.09, 54.94) | 0.75 (0.51, 1.04) |  | 168.07 | -0.04 (-0.36, 0.27) |
| Zimbabwe | 144.59 (99.09, 209.05) | 3.98 (2.82, 5.67) |  | 333.06 (220.44, 472.79) | 5.36 (3.65, 7.57) |  | 130.34 | 0.52 (0.10, 0.95) |

ASMR: age-standardized mortality rate; CI: confidence interval; EAPC: estimated annual percentage change; UI: uncertainty interval.

**
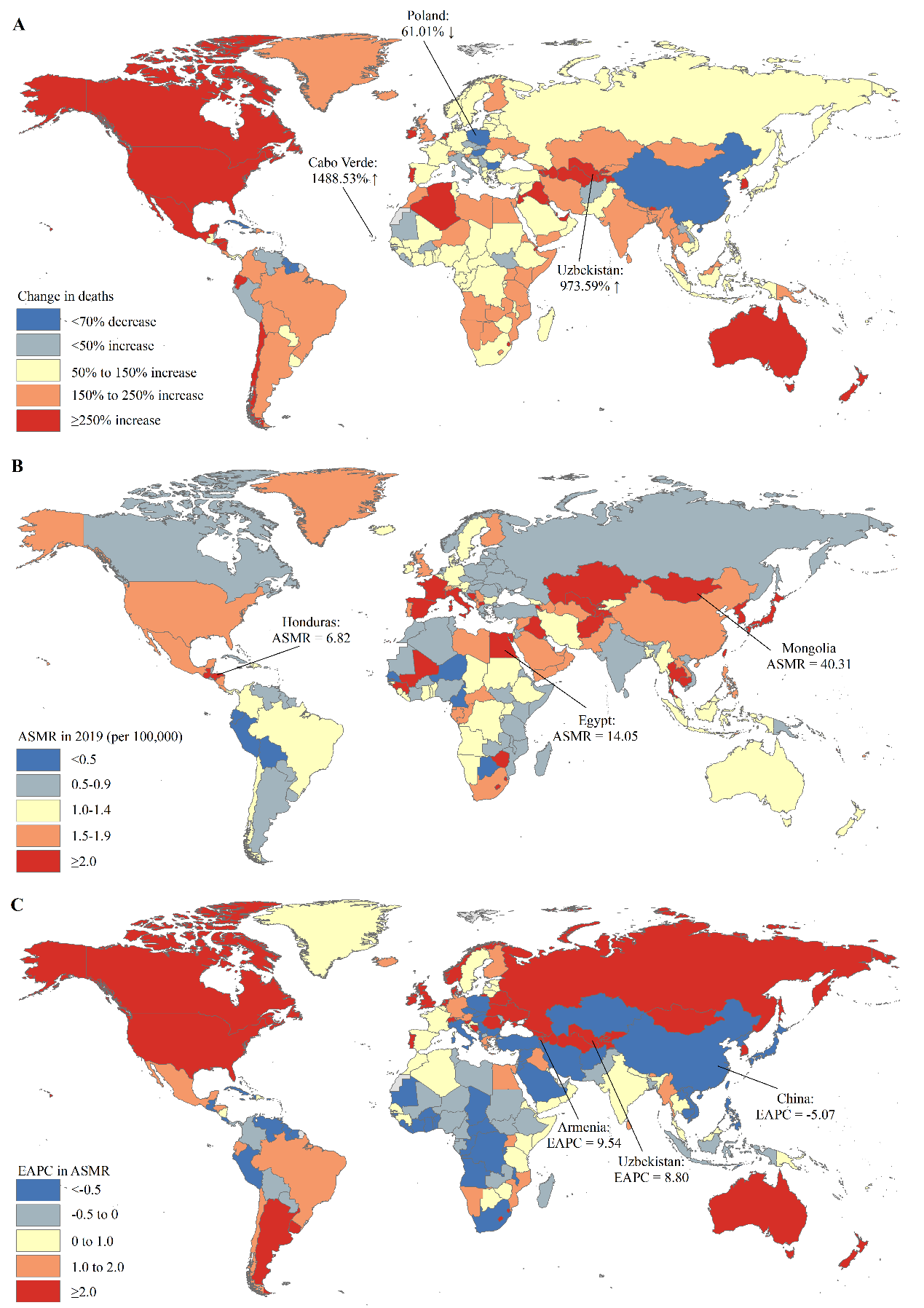
**

**Figure S7.** The global trends in the mortality of primary liver cancer due to hepatitis C in 204 countries and territories. (A) The percentage change in deaths of primary liver cancer due to hepatitis C between 1990 and 2019; (B) The ASMR of primary liver cancer due to hepatitis C in 2019; (C) The EAPCs in ASMR of primary liver cancer due to hepatitis C from 1990 to 2019.

Note: ASMR: age-standardized mortality rate; EAPC: estimated annual percentage change.

**Table S21. The deaths and ASMR of primary liver cancer due to alcohol use in 1990 and 2019 and their change trends from 1990 to 2019 at national level.**

| Nation | 1990 | |  | 2019 | |  | 1990-2019 | |
| --- | --- | --- | --- | --- | --- | --- | --- | --- |
|  | Deaths  No. (95% UI) | ASMR per 100,000  No. (95% UI) |  | Deaths  No. x 10^3^ (95% UI) | ASMR per 100,000  No. (95% UI) |  | Percentage change in deaths  No. (%) | EAPC in ASMR  No. (95% CI) |
| Afghanistan | 71.08 (41.74, 111.77) | 1.01 (0.60, 1.56) |  | 103.13 (62.53, 159.39) | 0.88 (0.53, 1.33) |  | 45.09 | -0.55 (-0.59, -0.51) |
| Albania | 68.45 (49.73, 88.66) | 3.42 (2.48, 4.47) |  | 111.49 (70.08, 162.84) | 2.53 (1.60, 3.68) |  | 62.87 | -1.82 (-2.31, -1.34) |
| Algeria | 19.44 (11.55, 29.87) | 0.17 (0.10, 0.25) |  | 72.18 (42.77, 110.90) | 0.23 (0.13, 0.35) |  | 271.32 | 1.13 (1.00, 1.26) |
| American Samoa | 0.14 (0.08, 0.22) | 0.62 (0.38, 0.95) |  | 0.38 (0.23, 0.56) | 0.78 (0.49, 1.17) |  | 167.97 | 1.21 (1.01, 1.40) |
| Andorra | 2.18 (1.47, 3.22) | 3.90 (2.66, 5.72) |  | 5.17 (3.40, 7.15) | 3.73 (2.45, 5.15) |  | 137.31 | -0.18 (-0.21, -0.14) |
| Angola | 13.73 (8.18, 21.47) | 0.38 (0.23, 0.57) |  | 46.10 (29.33, 68.51) | 0.44 (0.28, 0.65) |  | 235.75 | 0.66 (0.58, 0.74) |
| Antigua and Barbuda | 1.39 (1.01, 1.83) | 2.60 (1.88, 3.39) |  | 0.99 (0.71, 1.34) | 1.00 (0.72, 1.35) |  | -28.80 | -3.43 (-4.47, -2.38) |
| Argentina | 166.24 (115.63, 219.99) | 0.51 (0.36, 0.68) |  | 385.08 (274.49, 497.36) | 0.70 (0.50, 0.91) |  | 131.64 | 1.88 (1.58, 2.19) |
| Armenia | 6.91 (4.83, 9.31) | 0.27 (0.19, 0.36) |  | 85.46 (59.47, 116.86) | 2.06 (1.44, 2.80) |  | 1137.65 | 10.45 (8.78, 12.15) |
| Australia | 163.43 (123.76, 199.74) | 0.82 (0.63, 1.01) |  | 658.93 (497.03, 823.04) | 1.62 (1.22, 2.01) |  | 303.20 | 2.78 (2.52, 3.03) |
| Austria | 202.92 (166.91, 236.28) | 1.74 (1.44, 2.02) |  | 419.56 (336.03, 506.13) | 2.40 (1.94, 2.90) |  | 106.76 | 1.33 (1.07, 1.58) |
| Azerbaijan | 11.11 (7.66, 15.04) | 0.24 (0.16, 0.32) |  | 100.34 (61.94, 151.86) | 1.16 (0.72, 1.76) |  | 802.78 | 6.70 (4.63, 8.81) |
| Bahamas | 4.83 (3.56, 6.26) | 3.16 (2.33, 4.07) |  | 4.19 (2.90, 5.82) | 1.09 (0.75, 1.49) |  | -13.16 | -3.88 (-4.86, -2.90) |
| Bahrain | 1.04 (0.63, 1.57) | 0.71 (0.44, 1.06) |  | 4.14 (2.45, 6.53) | 0.52 (0.31, 0.81) |  | 297.80 | -1.88 (-2.27, -1.49) |
| Bangladesh | 330.25 (220.94, 471.20) | 0.70 (0.46, 1.00) |  | 742.07 (457.23, 1088.67) | 0.58 (0.35, 0.84) |  | 124.70 | -1.09 (-1.27, -0.91) |
| Barbados | 2.87 (1.80, 4.36) | 0.96 (0.60, 1.45) |  | 5.00 (3.51, 6.67) | 1.00 (0.70, 1.33) |  | 74.19 | -0.05 (-0.40, 0.30) |
| Belarus | 60.71 (45.14, 75.45) | 0.46 (0.35, 0.58) |  | 161.87 (104.96, 235.06) | 1.01 (0.66, 1.48) |  | 166.62 | 3.72 (2.88, 4.57) |
| Belgium | 164.45 (123.42, 206.64) | 1.05 (0.79, 1.31) |  | 326.37 (243.46, 416.13) | 1.47 (1.10, 1.87) |  | 98.47 | 1.12 (0.79, 1.46) |
| Belize | 1.79 (1.29, 2.33) | 1.96 (1.41, 2.55) |  | 2.94 (2.13, 3.83) | 1.13 (0.82, 1.48) |  | 64.55 | -2.01 (-2.63, -1.38) |
| Benin | 24.29 (15.89, 37.35) | 1.25 (0.82, 1.93) |  | 46.07 (28.72, 72.03) | 1.00 (0.62, 1.55) |  | 89.66 | -1.12 (-1.31, -0.93) |
| Bermuda | 1.80 (1.34, 2.29) | 2.89 (2.16, 3.67) |  | 1.05 (0.76, 1.40) | 0.79 (0.57, 1.06) |  | -41.54 | -4.38 (-5.50, -3.24) |
| Bhutan | 1.64 (0.95, 2.77) | 0.66 (0.40, 1.09) |  | 5.12 (2.98, 8.13) | 0.94 (0.54, 1.48) |  | 212.36 | 1.18 (1.09, 1.28) |
| Bolivia | 44.52 (28.13, 66.25) | 1.47 (0.94, 2.18) |  | 131.76 (85.75, 190.59) | 1.57 (1.02, 2.27) |  | 195.96 | 0.23 (0.08, 0.37) |
| Bosnia and Herzegovina | 76.95 (55.44, 100.34) | 1.89 (1.39, 2.47) |  | 167.73 (113.76, 232.30) | 2.73 (1.86, 3.77) |  | 117.96 | 1.71 (1.42, 2.00) |
| Botswana | 1.06 (0.39, 3.03) | 0.19 (0.07, 0.54) |  | 4.59 (2.76, 7.27) | 0.32 (0.20, 0.50) |  | 333.18 | 0.98 (-0.05, 2.02) |
| Brazil | 499.09 (432.66, 569.08) | 0.57 (0.49, 0.65) |  | 1781.73 (1533.47, 2023.26) | 0.76 (0.65, 0.86) |  | 256.99 | 1.46 (1.31, 1.62) |
| Brunei | 0.88 (0.54, 1.33) | 0.95 (0.58, 1.44) |  | 3.16 (1.93, 4.88) | 1.06 (0.65, 1.57) |  | 260.97 | 1.17 (0.64, 1.70) |
| Bulgaria | 334.32 (253.63, 413.42) | 2.59 (2.00, 3.19) |  | 295.26 (212.65, 397.51) | 2.08 (1.49, 2.81) |  | -11.68 | -0.09 (-0.68, 0.51) |
| Burkina Faso | 19.78 (12.31, 29.10) | 0.48 (0.30, 0.69) |  | 33.65 (21.15, 48.76) | 0.40 (0.25, 0.58) |  | 70.16 | -0.91 (-1.13, -0.69) |
| Burundi | 29.53 (18.84, 43.66) | 1.29 (0.83, 1.91) |  | 38.54 (21.43, 69.38) | 0.89 (0.51, 1.58) |  | 30.52 | -1.88 (-2.12, -1.64) |
| Cambodia | 54.87 (35.14, 82.58) | 1.25 (0.80, 1.91) |  | 187.19 (119.26, 271.89) | 1.61 (1.03, 2.32) |  | 241.15 | 0.61 (0.44, 0.79) |
| Cameroon | 5.49 (3.12, 9.51) | 0.13 (0.07, 0.23) |  | 16.76 (10.28, 25.93) | 0.15 (0.09, 0.23) |  | 205.34 | 0.30 (0.09, 0.52) |
| Canada | 349.55 (299.45, 397.73) | 1.07 (0.92, 1.22) |  | 1477.21 (1184.12, 1747.91) | 2.17 (1.75, 2.56) |  | 322.60 | 2.78 (2.55, 3.01) |
| Cape Verde | 0.46 (0.30, 0.65) | 0.20 (0.13, 0.28) |  | 9.90 (6.76, 13.63) | 2.40 (1.62, 3.32) |  | 2060.08 | 6.71 (4.40, 9.07) |
| Central African Republic | 7.09 (3.97, 11.29) | 0.61 (0.36, 0.94) |  | 11.22 (6.13, 19.52) | 0.54 (0.30, 0.92) |  | 58.37 | -1.06 (-1.32, -0.81) |
| Chad | 27.39 (15.92, 42.90) | 0.98 (0.58, 1.52) |  | 48.46 (29.43, 74.52) | 0.92 (0.56, 1.41) |  | 76.93 | -0.38 (-0.49, -0.27) |
| Chile | 68.51 (49.83, 88.79) | 0.69 (0.50, 0.90) |  | 228.21 (164.02, 296.11) | 0.94 (0.68, 1.22) |  | 233.08 | 1.75 (1.41, 2.09) |
| China | 17525.56 (13038.58, 23668.59) | 1.99 (1.49, 2.67) |  | 17435.70 (12814.45, 22545.49) | 0.85 (0.63, 1.09) |  | -0.51 | -4.46 (-5.32, -3.60) |
| Colombia | 148.31 (108.30, 190.08) | 0.87 (0.64, 1.12) |  | 440.15 (292.34, 632.26) | 0.84 (0.56, 1.21) |  | 196.78 | 0.08 (-0.44, 0.60) |
| Comoros | 1.49 (0.70, 3.09) | 0.68 (0.34, 1.42) |  | 2.86 (1.59, 5.53) | 0.61 (0.34, 1.16) |  | 92.19 | -0.49 (-0.65, -0.34) |
| Congo | 6.83 (3.98, 10.87) | 0.65 (0.38, 1.01) |  | 12.85 (7.62, 20.31) | 0.52 (0.32, 0.79) |  | 88.07 | -1.12 (-1.31, -0.93) |
| Cook Islands | 0.20 (0.13, 0.29) | 1.61 (1.00, 2.33) |  | 0.45 (0.29, 0.66) | 1.82 (1.17, 2.63) |  | 123.32 | 0.73 (0.60, 0.87) |
| Costa Rica | 33.07 (24.22, 42.05) | 1.93 (1.41, 2.47) |  | 92.59 (62.46, 131.00) | 1.82 (1.24, 2.58) |  | 179.99 | -0.40 (-0.99, 0.19) |
| Cote d'Ivoire | 58.33 (35.01, 88.95) | 1.55 (0.94, 2.36) |  | 105.45 (64.06, 164.79) | 1.08 (0.67, 1.67) |  | 80.78 | -2.16 (-2.54, -1.79) |
| Croatia | 89.10 (69.03, 108.44) | 1.38 (1.08, 1.69) |  | 146.50 (103.27, 197.15) | 1.66 (1.17, 2.22) |  | 64.41 | 1.00 (0.40, 1.60) |
| Cuba | 226.86 (168.79, 289.17) | 2.18 (1.63, 2.77) |  | 173.39 (118.63, 236.40) | 0.90 (0.62, 1.23) |  | -23.57 | -3.21 (-4.51, -1.90) |
| Cyprus | 9.80 (6.71, 13.34) | 1.18 (0.84, 1.59) |  | 24.43 (17.60, 31.88) | 1.24 (0.90, 1.61) |  | 149.36 | 0.31 (0.13, 0.48) |
| Czech Republic | 288.73 (231.23, 343.75) | 2.06 (1.65, 2.45) |  | 309.39 (229.26, 402.63) | 1.44 (1.07, 1.88) |  | 7.15 | -1.47 (-1.67, -1.27) |
| Democratic Republic of the Congo | 52.86 (33.61, 78.09) | 0.35 (0.23, 0.51) |  | 103.95 (62.21, 158.13) | 0.29 (0.18, 0.44) |  | 96.65 | -0.76 (-0.87, -0.65) |
| Denmark | 62.72 (47.79, 78.05) | 0.77 (0.59, 0.95) |  | 146.76 (108.74, 186.91) | 1.28 (0.95, 1.63) |  | 134.00 | 2.11 (1.85, 2.36) |
| Djibouti | 1.06 (0.51, 2.34) | 0.79 (0.40, 1.68) |  | 4.70 (2.43, 9.07) | 0.84 (0.46, 1.62) |  | 345.95 | 0.15 (0.01, 0.30) |
| Dominica | 2.20 (1.57, 2.89) | 2.99 (2.15, 3.90) |  | 1.11 (0.77, 1.54) | 1.22 (0.84, 1.70) |  | -49.66 | -3.35 (-4.21, -2.48) |
| Dominican Republic | 49.33 (35.75, 64.49) | 1.35 (0.98, 1.77) |  | 158.23 (94.68, 265.26) | 1.73 (1.04, 2.87) |  | 220.73 | 1.01 (0.51, 1.51) |
| Ecuador | 39.48 (27.92, 53.47) | 0.78 (0.55, 1.07) |  | 161.74 (107.93, 232.37) | 1.11 (0.75, 1.59) |  | 309.71 | 1.78 (1.41, 2.16) |
| Egypt | 383.53 (225.70, 597.00) | 1.27 (0.75, 1.97) |  | 1199.79 (655.54, 2084.32) | 1.80 (1.00, 3.09) |  | 212.83 | 2.08 (1.68, 2.48) |
| El Salvador | 26.50 (19.14, 34.51) | 0.91 (0.65, 1.19) |  | 39.31 (25.47, 56.19) | 0.67 (0.43, 0.96) |  | 48.33 | -1.33 (-1.92, -0.74) |
| Equatorial Guinea | 0.86 (0.50, 1.35) | 0.44 (0.26, 0.67) |  | 2.77 (1.41, 4.64) | 0.62 (0.32, 1.02) |  | 223.19 | 1.36 (1.14, 1.57) |
| Eritrea | 7.34 (3.93, 13.20) | 0.76 (0.42, 1.36) |  | 18.79 (10.50, 32.05) | 0.74 (0.42, 1.24) |  | 155.82 | -0.48 (-0.69, -0.26) |
| Estonia | 15.49 (11.49, 19.42) | 0.75 (0.56, 0.94) |  | 41.63 (29.49, 54.88) | 1.60 (1.13, 2.12) |  | 168.85 | 1.95 (1.34, 2.56) |
| Eswatini | 3.22 (1.64, 7.72) | 1.10 (0.56, 2.59) |  | 27.36 (6.63, 56.70) | 4.58 (1.14, 9.27) |  | 748.70 | 6.06 (4.53, 7.61) |
| Ethiopia | 134.13 (88.78, 192.24) | 0.70 (0.47, 0.96) |  | 263.55 (193.94, 357.38) | 0.69 (0.51, 0.93) |  | 96.48 | -0.18 (-0.32, -0.05) |
| Federated States of Micronesia | 0.66 (0.38, 1.04) | 1.40 (0.81, 2.24) |  | 0.95 (0.51, 1.56) | 1.34 (0.75, 2.10) |  | 45.40 | -0.24 (-0.39, -0.09) |
| Fiji | 2.55 (1.58, 3.94) | 0.73 (0.45, 1.11) |  | 6.31 (3.73, 9.88) | 0.84 (0.51, 1.31) |  | 147.21 | 1.05 (0.67, 1.43) |
| Finland | 74.69 (55.81, 94.03) | 1.03 (0.77, 1.29) |  | 192.71 (140.95, 245.46) | 1.54 (1.14, 1.94) |  | 158.00 | 1.88 (1.67, 2.09) |
| France | 1507.93 (1103.20, 1914.44) | 1.87 (1.38, 2.36) |  | 2557.89 (1802.38, 3413.19) | 1.97 (1.40, 2.61) |  | 69.63 | -0.21 (-0.45, 0.04) |
| Gabon | 3.34 (1.92, 5.12) | 0.60 (0.35, 0.91) |  | 6.45 (3.45, 11.41) | 0.64 (0.34, 1.10) |  | 92.90 | 0.04 (-0.11, 0.19) |
| Gambia | 19.60 (12.40, 29.78) | 5.40 (3.45, 8.02) |  | 74.72 (43.64, 112.13) | 7.70 (4.50, 11.55) |  | 281.28 | 0.98 (0.73, 1.22) |
| Georgia | 18.66 (12.81, 24.94) | 0.30 (0.21, 0.40) |  | 70.50 (49.51, 94.77) | 1.22 (0.85, 1.63) |  | 277.89 | 4.12 (2.90, 5.35) |
| Germany | 1329.52 (1071.46, 1598.29) | 1.05 (0.85, 1.25) |  | 3549.14 (2848.09, 4233.79) | 1.88 (1.53, 2.24) |  | 166.95 | 2.50 (2.14, 2.86) |
| Ghana | 73.46 (45.86, 115.48) | 1.23 (0.77, 1.90) |  | 210.80 (135.67, 309.06) | 1.36 (0.89, 1.98) |  | 186.94 | 0.13 (-0.05, 0.31) |
| Greece | 119.91 (88.69, 154.46) | 0.76 (0.57, 0.98) |  | 266.34 (196.51, 342.25) | 1.10 (0.82, 1.41) |  | 122.11 | 1.50 (1.30, 1.69) |
| Greenland | 0.70 (0.52, 0.91) | 1.88 (1.41, 2.41) |  | 1.92 (1.31, 2.66) | 2.68 (1.86, 3.67) |  | 174.96 | 1.51 (1.40, 1.63) |
| Grenada | 2.28 (1.67, 2.96) | 3.16 (2.28, 4.10) |  | 1.32 (0.97, 1.72) | 1.19 (0.88, 1.54) |  | -41.99 | -3.81 (-4.72, -2.88) |
| Guam | 0.35 (0.22, 0.52) | 0.46 (0.29, 0.68) |  | 1.41 (0.86, 2.12) | 0.73 (0.45, 1.10) |  | 307.29 | 2.11 (1.87, 2.36) |
| Guatemala | 85.13 (58.61, 114.18) | 2.44 (1.68, 3.26) |  | 130.01 (85.44, 188.22) | 1.20 (0.78, 1.73) |  | 52.72 | -3.41 (-4.83, -1.97) |
| Guinea | 168.09 (109.24, 252.07) | 5.16 (3.36, 7.57) |  | 326.89 (189.05, 492.86) | 6.14 (3.54, 9.17) |  | 94.48 | 0.77 (0.72, 0.82) |
| Guinea-Bissau | 5.73 (3.40, 9.23) | 1.44 (0.87, 2.30) |  | 7.86 (4.72, 12.11) | 1.15 (0.71, 1.75) |  | 37.08 | -1.03 (-1.12, -0.94) |
| Guyana | 9.01 (6.49, 11.80) | 2.42 (1.75, 3.18) |  | 6.73 (4.57, 9.36) | 1.11 (0.76, 1.54) |  | -25.31 | -2.83 (-3.72, -1.93) |
| Haiti | 70.29 (40.67, 111.37) | 2.25 (1.28, 3.65) |  | 96.00 (51.40, 162.54) | 1.47 (0.80, 2.50) |  | 36.58 | -1.66 (-1.88, -1.44) |
| Honduras | 63.10 (20.01, 104.67) | 3.18 (0.96, 5.36) |  | 267.10 (110.73, 433.33) | 4.55 (1.87, 7.31) |  | 323.29 | 1.70 (1.46, 1.94) |
| Hungary | 447.99 (357.23, 533.74) | 2.98 (2.39, 3.55) |  | 218.78 (158.08, 288.53) | 1.12 (0.80, 1.48) |  | -51.16 | -2.64 (-3.49, -1.78) |
| Iceland | 1.72 (1.21, 2.24) | 0.60 (0.43, 0.78) |  | 5.69 (4.18, 7.40) | 1.04 (0.76, 1.35) |  | 231.67 | 2.00 (1.85, 2.14) |
| India | 2907.63 (2212.54, 3672.06) | 0.69 (0.52, 0.87) |  | 8576.68 (6764.39, 10583.29) | 0.76 (0.61, 0.94) |  | 194.97 | 0.31 (0.22, 0.41) |
| Indonesia | 417.09 (330.23, 520.80) | 0.44 (0.35, 0.55) |  | 877.80 (678.08, 1097.73) | 0.43 (0.34, 0.53) |  | 110.46 | -0.14 (-0.22, -0.06) |
| Iran | 118.19 (84.58, 159.07) | 0.50 (0.36, 0.65) |  | 271.17 (205.28, 354.82) | 0.40 (0.30, 0.52) |  | 129.44 | -1.14 (-1.76, -0.51) |
| Iraq | 32.36 (19.68, 50.05) | 0.43 (0.26, 0.66) |  | 128.65 (77.66, 194.90) | 0.60 (0.37, 0.91) |  | 297.60 | 1.31 (0.84, 1.78) |
| Ireland | 21.66 (15.98, 27.81) | 0.51 (0.38, 0.66) |  | 95.08 (69.70, 122.59) | 1.26 (0.93, 1.61) |  | 338.97 | 3.62 (3.24, 4.00) |
| Israel | 30.33 (20.56, 41.28) | 0.61 (0.42, 0.83) |  | 84.28 (58.86, 112.19) | 0.73 (0.51, 0.97) |  | 177.86 | 0.63 (0.55, 0.72) |
| Italy | 1596.22 (1377.71, 1808.97) | 1.77 (1.53, 2.00) |  | 1723.91 (1450.42, 2008.47) | 1.25 (1.05, 1.45) |  | 8.00 | -1.61 (-1.90, -1.33) |
| Jamaica | 23.79 (17.76, 30.97) | 1.32 (0.99, 1.72) |  | 27.25 (18.72, 37.54) | 0.93 (0.64, 1.28) |  | 14.57 | -0.56 (-1.51, 0.41) |
| Japan | 2591.68 (2204.31, 2997.98) | 1.49 (1.27, 1.72) |  | 3562.08 (2919.30, 4194.45) | 0.99 (0.83, 1.16) |  | 37.44 | -2.25 (-2.77, -1.73) |
| Jordan | 3.77 (2.30, 5.68) | 0.32 (0.19, 0.48) |  | 15.79 (9.42, 24.78) | 0.27 (0.16, 0.42) |  | 318.65 | -0.62 (-0.74, -0.51) |
| Kazakhstan | 139.69 (101.11, 177.58) | 1.10 (0.80, 1.40) |  | 359.54 (253.58, 469.72) | 2.07 (1.49, 2.72) |  | 157.39 | -1.37 (-2.59, -0.13) |
| Kenya | 56.09 (34.09, 103.42) | 0.71 (0.43, 1.30) |  | 182.54 (112.38, 284.65) | 0.86 (0.54, 1.33) |  | 225.44 | 0.02 (-0.45, 0.49) |
| Kiribati | 0.55 (0.33, 0.86) | 1.42 (0.86, 2.18) |  | 0.88 (0.52, 1.33) | 1.21 (0.72, 1.78) |  | 58.90 | -0.63 (-0.70, -0.56) |
| Kuwait | 1.61 (1.04, 2.37) | 0.29 (0.18, 0.43) |  | 5.51 (3.31, 8.57) | 0.26 (0.15, 0.40) |  | 242.67 | 0.44 (0.10, 0.79) |
| Kyrgyzstan | 10.08 (7.06, 13.43) | 0.34 (0.24, 0.45) |  | 35.26 (24.61, 46.61) | 0.81 (0.57, 1.08) |  | 249.70 | 4.00 (3.48, 4.53) |
| Lao | 44.51 (26.08, 70.61) | 2.12 (1.28, 3.30) |  | 72.15 (45.17, 108.74) | 1.69 (1.10, 2.51) |  | 62.09 | -1.08 (-1.26, -0.90) |
| Latvia | 21.46 (16.18, 27.08) | 0.59 (0.45, 0.75) |  | 45.42 (32.89, 59.39) | 1.16 (0.84, 1.52) |  | 111.69 | 1.52 (0.71, 2.33) |
| Lebanon | 10.11 (6.26, 15.22) | 0.46 (0.29, 0.68) |  | 20.05 (11.45, 33.33) | 0.38 (0.22, 0.63) |  | 98.25 | -0.53 (-0.64, -0.42) |
| Lesotho | 9.90 (4.66, 24.43) | 1.00 (0.49, 2.44) |  | 45.89 (15.37, 78.20) | 3.56 (1.22, 6.01) |  | 363.57 | 4.77 (3.90, 5.65) |
| Liberia | 14.87 (9.21, 23.52) | 1.35 (0.84, 2.11) |  | 19.12 (11.48, 30.90) | 1.01 (0.61, 1.65) |  | 28.55 | -1.25 (-1.42, -1.07) |
| Libya | 8.89 (5.36, 13.90) | 0.50 (0.30, 0.79) |  | 24.13 (14.03, 39.61) | 0.50 (0.28, 0.80) |  | 171.35 | 0.06 (-0.07, 0.19) |
| Lithuania | 26.42 (19.49, 32.87) | 0.58 (0.43, 0.73) |  | 75.73 (53.18, 98.61) | 1.36 (0.96, 1.78) |  | 186.61 | 2.45 (2.08, 2.81) |
| Luxembourg | 6.64 (4.98, 8.22) | 1.20 (0.91, 1.48) |  | 13.91 (9.87, 18.78) | 1.41 (1.00, 1.91) |  | 109.66 | 0.66 (0.43, 0.89) |
| Macedonia | 61.43 (45.29, 78.87) | 3.30 (2.44, 4.21) |  | 105.25 (70.23, 148.00) | 3.20 (2.15, 4.47) |  | 71.33 | -0.27 (-0.39, -0.15) |
| Madagascar | 37.08 (18.95, 78.95) | 0.75 (0.38, 1.59) |  | 64.26 (34.83, 117.16) | 0.63 (0.34, 1.15) |  | 73.29 | -0.78 (-0.95, -0.61) |
| Malawi | 29.22 (16.71, 51.01) | 0.77 (0.45, 1.33) |  | 51.65 (33.89, 73.95) | 0.76 (0.50, 1.08) |  | 76.74 | -1.09 (-1.57, -0.61) |
| Malaysia | 66.83 (43.06, 98.72) | 0.76 (0.49, 1.14) |  | 222.89 (137.08, 348.15) | 0.85 (0.52, 1.31) |  | 233.50 | 0.50 (0.17, 0.82) |
| Maldives | 1.23 (0.68, 2.17) | 1.53 (0.88, 2.62) |  | 3.53 (2.34, 5.08) | 1.26 (0.82, 1.83) |  | 187.68 | -0.61 (-0.73, -0.48) |
| Mali | 106.30 (65.36, 157.95) | 2.46 (1.53, 3.65) |  | 227.80 (139.04, 351.15) | 2.59 (1.59, 3.90) |  | 114.30 | 0.13 (0.01, 0.24) |
| Malta | 2.30 (1.65, 2.96) | 0.53 (0.38, 0.68) |  | 7.33 (5.16, 9.64) | 0.78 (0.56, 1.02) |  | 218.45 | 1.44 (1.20, 1.68) |
| Marshall Islands | 0.23 (0.13, 0.37) | 1.44 (0.83, 2.34) |  | 0.46 (0.27, 0.76) | 1.35 (0.80, 2.18) |  | 99.11 | -0.12 (-0.26, 0.01) |
| Mauritania | 10.40 (6.48, 16.08) | 1.04 (0.66, 1.59) |  | 13.21 (7.83, 20.21) | 0.67 (0.40, 1.03) |  | 27.03 | -1.52 (-1.68, -1.36) |
| Mauritius | 2.91 (2.00, 3.98) | 0.40 (0.28, 0.54) |  | 8.14 (5.13, 12.27) | 0.47 (0.30, 0.70) |  | 179.71 | 1.87 (1.48, 2.27) |
| Mexico | 261.94 (228.17, 297.56) | 0.64 (0.56, 0.73) |  | 1277.17 (1023.56, 1562.40) | 1.11 (0.89, 1.36) |  | 387.57 | 1.93 (1.79, 2.08) |
| Moldova | 37.67 (31.28, 43.78) | 0.85 (0.71, 0.99) |  | 67.48 (53.05, 84.15) | 1.16 (0.92, 1.45) |  | 79.15 | -1.45 (-3.05, 0.18) |
| Monaco | 0.77 (0.51, 1.07) | 1.11 (0.74, 1.53) |  | 2.49 (1.71, 3.41) | 2.68 (1.84, 3.71) |  | 224.28 | 3.77 (2.90, 4.64) |
| Mongolia | 182.91 (115.53, 258.27) | 17.55 (11.24, 24.44) |  | 728.88 (477.30, 1053.99) | 34.20 (23.11, 47.83) |  | 298.49 | 3.00 (2.58, 3.43) |
| Montenegro | 16.40 (12.09, 21.14) | 2.65 (1.96, 3.41) |  | 25.41 (18.00, 34.50) | 2.53 (1.80, 3.42) |  | 54.92 | -0.20 (-0.34, -0.05) |
| Morocco | 28.16 (16.60, 44.10) | 0.22 (0.13, 0.35) |  | 75.78 (45.46, 117.02) | 0.26 (0.16, 0.41) |  | 169.08 | 0.42 (0.07, 0.76) |
| Mozambique | 26.26 (14.50, 51.74) | 0.48 (0.27, 0.95) |  | 99.50 (57.60, 145.87) | 0.99 (0.57, 1.45) |  | 278.85 | 2.49 (2.24, 2.74) |
| Myanmar | 117.22 (71.35, 197.66) | 0.54 (0.33, 0.89) |  | 393.73 (254.31, 571.82) | 0.89 (0.59, 1.27) |  | 235.88 | 1.74 (1.56, 1.92) |
| Namibia | 2.75 (1.29, 6.28) | 0.38 (0.18, 0.84) |  | 11.85 (7.44, 17.01) | 0.84 (0.54, 1.20) |  | 331.17 | 2.92 (2.41, 3.43) |
| Nauru | 0.05 (0.03, 0.08) | 1.31 (0.79, 2.01) |  | 0.05 (0.03, 0.09) | 1.24 (0.71, 1.90) |  | 5.14 | -0.30 (-0.62, 0.01) |
| Nepal | 43.28 (29.09, 61.69) | 0.51 (0.34, 0.73) |  | 134.31 (78.83, 220.04) | 0.64 (0.38, 1.04) |  | 210.34 | 0.97 (0.80, 1.13) |
| Netherlands | 111.40 (87.53, 135.31) | 0.56 (0.44, 0.67) |  | 378.85 (288.95, 469.96) | 1.11 (0.86, 1.36) |  | 240.09 | 2.69 (2.53, 2.85) |
| New Zealand | 33.61 (29.07, 38.02) | 0.87 (0.75, 0.98) |  | 106.16 (92.22, 120.49) | 1.41 (1.23, 1.60) |  | 215.81 | 1.92 (1.73, 2.11) |
| Nicaragua | 13.71 (9.39, 18.31) | 0.92 (0.62, 1.24) |  | 53.33 (36.26, 74.94) | 1.26 (0.85, 1.75) |  | 289.05 | 1.32 (0.90, 1.74) |
| Niger | 2.95 (1.78, 4.45) | 0.11 (0.07, 0.17) |  | 7.69 (4.54, 11.91) | 0.11 (0.06, 0.16) |  | 160.85 | -0.35 (-0.45, -0.25) |
| Nigeria | 294.54 (206.67, 404.73) | 0.70 (0.50, 0.96) |  | 614.11 (450.51, 811.81) | 0.81 (0.61, 1.07) |  | 108.50 | 0.55 (0.46, 0.64) |
| Niue | 0.02 (0.01, 0.03) | 1.02 (0.63, 1.52) |  | 0.02 (0.01, 0.03) | 1.03 (0.65, 1.54) |  | 2.11 | -0.13 (-0.23, -0.04) |
| North Korea | 181.11 (107.35, 295.24) | 1.07 (0.65, 1.70) |  | 270.56 (156.47, 434.58) | 0.83 (0.48, 1.31) |  | 49.39 | -1.12 (-1.30, -0.95) |
| Northern Mariana Islands | 0.14 (0.08, 0.21) | 0.77 (0.48, 1.15) |  | 0.54 (0.32, 0.82) | 0.98 (0.63, 1.43) |  | 283.58 | 1.24 (1.01, 1.47) |
| Norway | 31.88 (27.84, 36.19) | 0.48 (0.42, 0.54) |  | 76.84 (64.23, 90.80) | 0.82 (0.69, 0.97) |  | 141.03 | 2.41 (2.20, 2.62) |
| Oman | 2.78 (1.55, 4.41) | 0.43 (0.24, 0.67) |  | 8.51 (5.24, 13.44) | 0.52 (0.33, 0.81) |  | 206.15 | 1.46 (0.95, 1.97) |
| Pakistan | 309.91 (195.22, 435.29) | 0.54 (0.34, 0.76) |  | 617.75 (461.84, 826.25) | 0.54 (0.41, 0.72) |  | 99.33 | 0.07 (-0.11, 0.25) |
| Palau | 0.13 (0.07, 0.21) | 1.29 (0.73, 2.11) |  | 0.31 (0.18, 0.48) | 1.36 (0.85, 2.04) |  | 139.12 | 0.24 (0.15, 0.33) |
| Palestine | 6.37 (3.67, 9.87) | 0.78 (0.44, 1.20) |  | 13.16 (8.16, 19.72) | 0.62 (0.38, 0.93) |  | 106.68 | -0.82 (-0.92, -0.71) |
| Panama | 15.83 (11.70, 20.32) | 1.07 (0.78, 1.38) |  | 43.66 (29.25, 63.21) | 1.06 (0.71, 1.54) |  | 175.77 | 0.37 (-0.14, 0.87) |
| Papua New Guinea | 3.09 (1.83, 4.89) | 0.19 (0.12, 0.30) |  | 8.96 (5.35, 14.48) | 0.23 (0.15, 0.37) |  | 190.24 | 0.80 (0.74, 0.87) |
| Paraguay | 18.35 (12.83, 23.75) | 0.85 (0.59, 1.11) |  | 39.82 (25.29, 57.16) | 0.74 (0.47, 1.07) |  | 116.97 | -0.48 (-1.10, 0.13) |
| Peru | 229.64 (162.12, 308.19) | 2.03 (1.43, 2.72) |  | 299.58 (194.34, 423.98) | 0.95 (0.61, 1.35) |  | 30.46 | -3.30 (-3.99, -2.62) |
| Philippines | 657.78 (445.16, 872.59) | 2.18 (1.48, 2.87) |  | 1243.67 (928.67, 1628.01) | 1.57 (1.18, 2.03) |  | 89.07 | -1.53 (-1.84, -1.22) |
| Poland | 1166.80 (1032.58, 1305.37) | 2.67 (2.37, 2.99) |  | 650.14 (528.74, 799.90) | 0.91 (0.74, 1.13) |  | -44.28 | -3.38 (-4.73, -2.00) |
| Portugal | 100.97 (76.48, 125.41) | 0.71 (0.55, 0.88) |  | 409.19 (302.85, 513.97) | 1.83 (1.36, 2.30) |  | 305.27 | 2.98 (2.60, 3.36) |
| Puerto Rico | 89.51 (65.97, 113.60) | 2.42 (1.79, 3.07) |  | 67.20 (44.32, 95.90) | 0.95 (0.62, 1.36) |  | -24.92 | -2.24 (-3.01, -1.46) |
| Qatar | 1.52 (0.87, 2.37) | 1.87 (1.08, 2.96) |  | 10.49 (5.69, 17.06) | 1.80 (1.02, 2.90) |  | 592.31 | 0.07 (-0.16, 0.29) |
| Romania | 205.91 (153.10, 258.03) | 0.74 (0.56, 0.91) |  | 498.56 (359.77, 649.82) | 1.37 (0.98, 1.79) |  | 142.13 | 3.01 (2.54, 3.49) |
| Russia | 1040.65 (904.67, 1184.55) | 0.57 (0.50, 0.65) |  | 2549.81 (2034.26, 3199.55) | 1.09 (0.87, 1.37) |  | 145.02 | 2.66 (2.37, 2.95) |
| Rwanda | 48.40 (30.90, 73.29) | 1.70 (1.12, 2.51) |  | 85.56 (57.03, 124.51) | 1.46 (1.00, 2.08) |  | 76.77 | -1.35 (-1.69, -1.01) |
| Saint Kitts and Nevis | 1.63 (1.17, 2.13) | 4.15 (3.01, 5.40) |  | 0.85 (0.59, 1.18) | 1.32 (0.93, 1.80) |  | -47.69 | -4.42 (-5.51, -3.31) |
| Saint Lucia | 2.06 (1.55, 2.63) | 2.34 (1.77, 2.97) |  | 1.91 (1.40, 2.50) | 0.90 (0.66, 1.17) |  | -7.61 | -3.57 (-4.61, -2.51) |
| Saint Vincent and the Grenadines | 1.78 (1.28, 2.31) | 2.43 (1.78, 3.16) |  | 1.61 (1.18, 2.06) | 1.19 (0.88, 1.52) |  | -9.44 | -2.51 (-3.48, -1.52) |
| Samoa | 0.72 (0.43, 1.11) | 0.82 (0.49, 1.24) |  | 0.98 (0.59, 1.46) | 0.67 (0.41, 1.00) |  | 35.36 | -0.71 (-0.78, -0.63) |
| San Marino | 0.26 (0.18, 0.35) | 0.76 (0.54, 1.01) |  | 0.66 (0.38, 1.01) | 1.05 (0.60, 1.63) |  | 154.07 | 1.59 (1.37, 1.82) |
| Sao Tome and Principe | 0.28 (0.18, 0.41) | 0.45 (0.29, 0.64) |  | 0.61 (0.36, 0.96) | 0.61 (0.35, 0.92) |  | 116.99 | 0.96 (0.82, 1.10) |
| Saudi Arabia | 25.44 (14.72, 41.49) | 0.47 (0.27, 0.76) |  | 59.20 (34.48, 95.49) | 0.42 (0.24, 0.67) |  | 132.72 | -0.70 (-0.98, -0.41) |
| Senegal | 10.04 (6.48, 14.71) | 0.33 (0.21, 0.47) |  | 21.29 (12.88, 31.67) | 0.30 (0.18, 0.45) |  | 111.94 | -0.01 (-0.19, 0.18) |
| Serbia | 250.49 (179.82, 326.28) | 2.19 (1.57, 2.85) |  | 333.69 (225.16, 462.35) | 2.03 (1.37, 2.79) |  | 33.21 | -0.49 (-0.76, -0.22) |
| Seychelles | 0.93 (0.62, 1.34) | 1.65 (1.10, 2.38) |  | 1.60 (1.04, 2.23) | 1.45 (0.97, 2.02) |  | 71.26 | -0.33 (-0.57, -0.10) |
| Sierra Leone | 21.97 (13.94, 33.91) | 1.17 (0.75, 1.80) |  | 31.62 (19.31, 48.00) | 0.93 (0.58, 1.40) |  | 43.92 | -0.95 (-1.11, -0.80) |
| Singapore | 13.17 (8.53, 19.25) | 0.61 (0.39, 0.90) |  | 52.78 (32.95, 79.88) | 0.69 (0.43, 1.04) |  | 300.76 | 0.35 (-0.00, 0.70) |
| Slovakia | 123.38 (96.03, 148.62) | 2.04 (1.61, 2.46) |  | 142.31 (98.86, 192.83) | 1.52 (1.05, 2.05) |  | 15.34 | -1.55 (-1.83, -1.27) |
| Slovenia | 28.65 (19.73, 39.53) | 1.16 (0.80, 1.60) |  | 87.94 (59.66, 124.38) | 2.06 (1.39, 2.91) |  | 206.96 | 2.26 (1.95, 2.57) |
| Solomon Islands | 1.02 (0.59, 1.66) | 0.76 (0.45, 1.22) |  | 1.92 (1.15, 2.89) | 0.66 (0.40, 0.98) |  | 88.12 | -0.44 (-0.52, -0.35) |
| Somalia | 19.35 (10.03, 41.88) | 0.82 (0.44, 1.75) |  | 46.27 (24.61, 88.26) | 0.74 (0.40, 1.43) |  | 139.14 | -0.30 (-0.40, -0.20) |
| South Africa | 260.77 (153.23, 500.57) | 1.24 (0.72, 2.35) |  | 552.23 (448.40, 662.25) | 1.23 (1.00, 1.47) |  | 111.77 | -0.62 (-1.33, 0.09) |
| South Korea | 439.23 (281.05, 647.60) | 1.47 (0.95, 2.18) |  | 2598.77 (1790.64, 3640.88) | 2.88 (2.00, 4.03) |  | 491.67 | 2.84 (1.57, 4.11) |
| South Sudan | 17.65 (8.53, 39.51) | 0.76 (0.37, 1.65) |  | 23.85 (11.32, 49.07) | 0.68 (0.33, 1.41) |  | 35.16 | -0.34 (-0.41, -0.27) |
| Spain | 673.25 (474.81, 873.58) | 1.21 (0.86, 1.56) |  | 1323.84 (918.20, 1791.43) | 1.45 (1.00, 1.93) |  | 96.64 | 0.33 (0.02, 0.64) |
| Sri Lanka | 45.54 (30.09, 64.51) | 0.45 (0.30, 0.64) |  | 180.60 (110.25, 275.84) | 0.70 (0.43, 1.05) |  | 296.54 | 2.64 (2.25, 3.03) |
| Sudan | 44.55 (22.16, 85.46) | 0.50 (0.25, 0.95) |  | 95.00 (49.29, 179.34) | 0.57 (0.30, 1.07) |  | 113.26 | 0.46 (0.30, 0.62) |
| Suriname | 5.74 (4.14, 7.48) | 2.27 (1.64, 2.96) |  | 5.67 (4.00, 7.97) | 0.96 (0.68, 1.34) |  | -1.28 | -2.99 (-3.81, -2.17) |
| Sweden | 149.97 (129.54, 170.31) | 1.00 (0.87, 1.13) |  | 271.95 (234.57, 309.15) | 1.35 (1.16, 1.53) |  | 81.33 | 1.61 (1.01, 2.21) |
| Switzerland | 106.59 (81.60, 129.47) | 1.03 (0.78, 1.25) |  | 305.94 (224.33, 388.80) | 1.80 (1.32, 2.29) |  | 187.02 | 1.41 (0.88, 1.94) |
| Syrian Arab Republic | 23.46 (14.07, 35.61) | 0.48 (0.29, 0.75) |  | 47.87 (27.03, 77.20) | 0.41 (0.24, 0.65) |  | 104.03 | -0.70 (-0.86, -0.55) |
| Taiwan (Province of China) | 202.19 (132.64, 293.16) | 1.20 (0.80, 1.74) |  | 262.63 (157.27, 409.20) | 0.66 (0.40, 1.02) |  | 29.89 | -3.24 (-4.19, -2.28) |
| Tajikistan | 5.94 (3.95, 8.15) | 0.22 (0.15, 0.31) |  | 38.65 (25.02, 56.06) | 0.89 (0.57, 1.30) |  | 550.42 | 5.62 (5.36, 5.88) |
| Tanzania | 60.06 (39.77, 87.71) | 0.58 (0.39, 0.83) |  | 155.03 (101.24, 223.45) | 0.68 (0.44, 0.98) |  | 158.13 | 0.48 (0.30, 0.66) |
| Thailand | 1918.13 (1304.67, 2630.63) | 5.41 (3.73, 7.43) |  | 7385.87 (4775.47, 10827.51) | 7.17 (4.61, 10.45) |  | 285.06 | 1.20 (1.02, 1.38) |
| Timor-Leste | 3.80 (2.09, 6.24) | 1.40 (0.80, 2.26) |  | 10.74 (5.62, 17.77) | 1.33 (0.72, 2.16) |  | 182.55 | -0.50 (-0.70, -0.31) |
| Togo | 13.87 (8.51, 21.43) | 1.17 (0.71, 1.79) |  | 32.75 (20.26, 49.17) | 0.94 (0.60, 1.39) |  | 136.10 | -1.34 (-1.56, -1.11) |
| Tokelau | 0.01 (0.01, 0.02) | 0.95 (0.55, 1.48) |  | 0.01 (0.01, 0.02) | 0.88 (0.51, 1.35) |  | -9.41 | -0.32 (-0.36, -0.27) |
| Tonga | 1.64 (0.91, 2.60) | 2.92 (1.66, 4.54) |  | 2.61 (1.54, 4.07) | 3.31 (1.96, 5.14) |  | 58.97 | 0.43 (0.18, 0.67) |
| Trinidad and Tobago | 19.13 (14.09, 24.47) | 2.28 (1.67, 2.90) |  | 17.07 (11.12, 24.47) | 0.92 (0.60, 1.32) |  | -10.81 | -3.34 (-4.40, -2.26) |
| Tunisia | 10.39 (5.67, 17.35) | 0.21 (0.12, 0.35) |  | 26.29 (13.41, 47.55) | 0.21 (0.11, 0.38) |  | 153.11 | 0.02 (-0.10, 0.15) |
| Turkey | 163.21 (105.14, 240.50) | 0.49 (0.31, 0.73) |  | 337.33 (210.06, 515.05) | 0.39 (0.24, 0.60) |  | 106.68 | -0.72 (-0.91, -0.53) |
| Turkmenistan | 4.80 (3.29, 6.45) | 0.27 (0.18, 0.36) |  | 69.03 (45.26, 98.13) | 1.71 (1.14, 2.43) |  | 1338.65 | 7.41 (6.32, 8.52) |
| Tuvalu | 0.08 (0.05, 0.13) | 1.19 (0.70, 1.89) |  | 0.11 (0.06, 0.17) | 1.06 (0.63, 1.66) |  | 28.58 | -0.51 (-0.67, -0.36) |
| Uganda | 103.10 (68.21, 140.95) | 1.59 (1.06, 2.15) |  | 291.76 (198.44, 412.73) | 2.07 (1.42, 2.85) |  | 183.00 | 1.12 (0.92, 1.32) |
| Ukraine | 212.67 (183.09, 241.00) | 0.29 (0.25, 0.33) |  | 699.17 (561.11, 856.30) | 0.92 (0.74, 1.13) |  | 228.75 | 4.95 (4.25, 5.66) |
| United Arab Emirates | 2.15 (0.88, 4.59) | 0.58 (0.23, 1.30) |  | 21.77 (6.92, 58.82) | 0.61 (0.20, 1.63) |  | 911.33 | 0.13 (-0.06, 0.32) |
| United Kingdom | 602.07 (534.65, 670.38) | 0.66 (0.59, 0.74) |  | 1821.73 (1598.31, 2052.55) | 1.45 (1.28, 1.63) |  | 202.58 | 3.40 (3.18, 3.62) |
| United States of America | 1874.50 (1656.19, 2088.64) | 0.59 (0.52, 0.65) |  | 7384.01 (6001.43, 8767.81) | 1.35 (1.10, 1.60) |  | 293.92 | 2.74 (2.65, 2.82) |
| United States Virgin Islands | 1.11 (0.79, 1.52) | 1.33 (0.94, 1.81) |  | 1.74 (1.23, 2.41) | 0.90 (0.64, 1.22) |  | 56.64 | -1.27 (-1.91, -0.63) |
| Uruguay | 16.28 (11.43, 21.93) | 0.41 (0.29, 0.55) |  | 38.24 (26.76, 50.13) | 0.72 (0.51, 0.94) |  | 134.85 | 2.38 (2.19, 2.58) |
| Uzbekistan | 23.69 (16.77, 31.27) | 0.22 (0.15, 0.30) |  | 375.68 (252.63, 522.18) | 1.87 (1.28, 2.55) |  | 1486.05 | 9.80 (8.59, 11.03) |
| Vanuatu | 0.75 (0.38, 1.31) | 1.17 (0.59, 2.05) |  | 2.09 (1.11, 3.43) | 1.22 (0.66, 2.01) |  | 178.40 | 0.28 (0.14, 0.41) |
| Venezuela | 213.21 (156.47, 268.08) | 2.27 (1.66, 2.88) |  | 243.91 (161.72, 347.32) | 0.85 (0.57, 1.21) |  | 14.40 | -2.68 (-3.93, -1.42) |
| Viet Nam | 286.76 (173.86, 444.44) | 0.72 (0.44, 1.12) |  | 572.48 (351.35, 841.89) | 0.66 (0.40, 0.97) |  | 99.63 | -0.48 (-0.68, -0.28) |
| Yemen | 9.72 (5.14, 17.62) | 0.21 (0.11, 0.37) |  | 28.81 (16.36, 48.45) | 0.24 (0.13, 0.40) |  | 196.41 | 0.52 (0.42, 0.62) |
| Zambia | 17.76 (10.56, 31.62) | 0.69 (0.41, 1.23) |  | 50.13 (32.79, 70.36) | 0.83 (0.55, 1.16) |  | 182.33 | -0.10 (-0.55, 0.35) |
| Zimbabwe | 85.45 (48.56, 167.13) | 2.05 (1.18, 3.94) |  | 153.11 (91.54, 254.95) | 2.14 (1.30, 3.49) |  | 79.18 | -1.17 (-1.93, -0.41) |

ASMR: age-standardized mortality rate; CI: confidence interval; EAPC: estimated annual percentage change; UI: uncertainty interval.

**
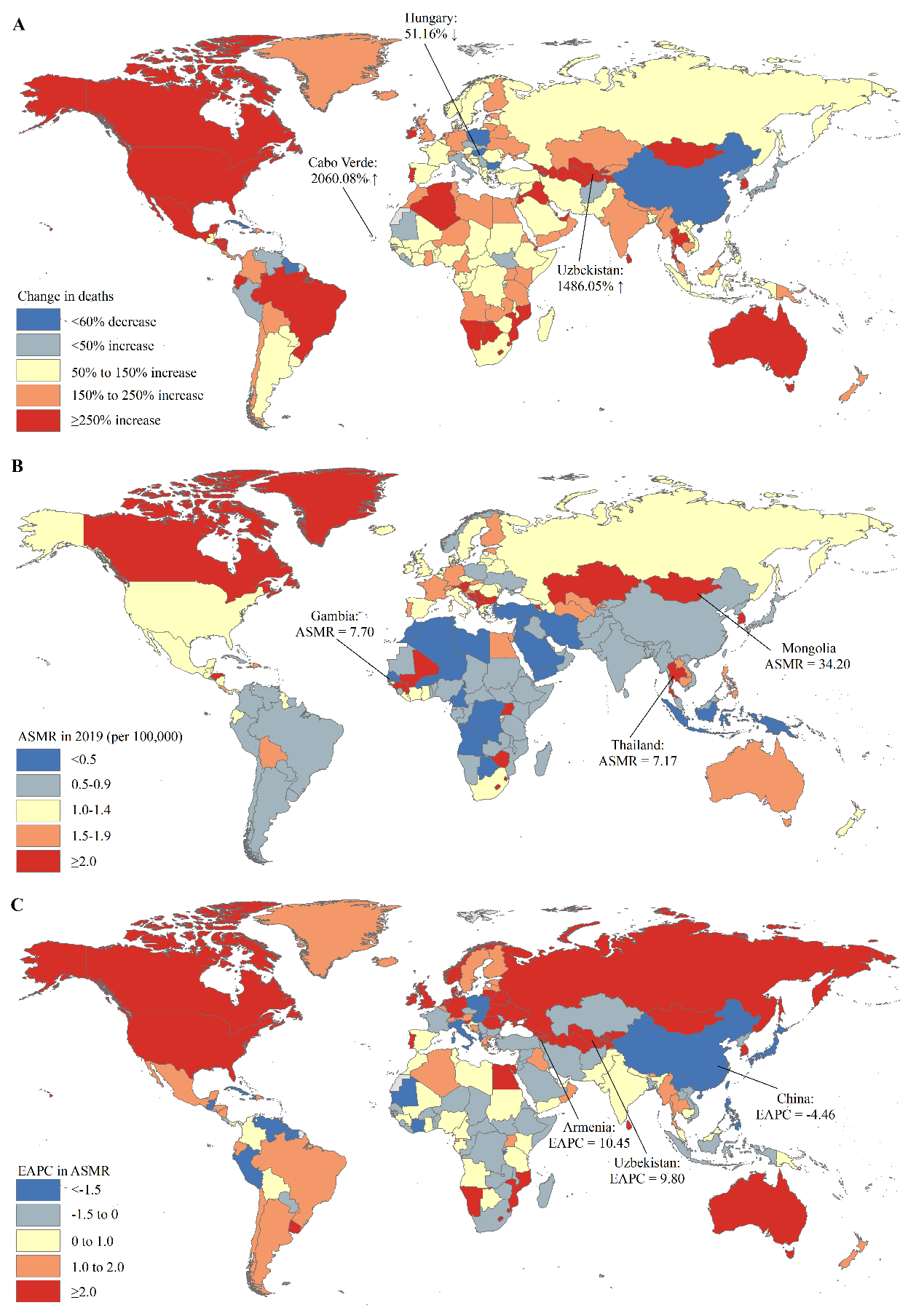
**

**Figure S8.** The global trends in the mortality of primary liver cancer due to alcohol use in 204 countries and territories. (A) The percentage change in deaths of primary liver cancer due to alcohol use between 1990 and 2019; (B) The ASMR of primary liver cancer due to alcohol use in 2019; (C) The EAPCs in ASMR of primary liver cancer due to alcohol use from 1990 to 2019.

Note: ASMR: age-standardized mortality rate; EAPC: estimated annual percentage change.

**Table S22. The deaths and ASMR of primary liver cancer due to NASH in 1990 and 2019 and their change trends from 1990 to 2019 at national level.**

| Nation | 1990 | |  | 2019 | |  | 1990-2019 | |
| --- | --- | --- | --- | --- | --- | --- | --- | --- |
|  | Deaths  No. (95% UI) | ASMR per 100,000  No. (95% UI) |  | Deaths  No. x 10^3^ (95% UI) | ASMR per 100,000  No. (95% UI) |  | Percentage change in deaths  No. (%) | EAPC in ASMR  No. (95% CI) |
| Afghanistan | 62.59 (40.95, 94.15) | 0.90 (0.60, 1.34) |  | 113.35 (72.27, 163.79) | 0.90 (0.57, 1.34) |  | 81.11 | 0.05 (0.01, 0.08) |
| Albania | 16.66 (11.64, 23.34) | 0.88 (0.61, 1.24) |  | 24.22 (14.95, 37.58) | 0.56 (0.35, 0.86) |  | 45.44 | -2.72 (-3.35, -2.09) |
| Algeria | 17.25 (11.15, 25.89) | 0.16 (0.10, 0.24) |  | 75.89 (48.45, 113.87) | 0.25 (0.16, 0.37) |  | 339.92 | 1.66 (1.57, 1.74) |
| American Samoa | 0.13 (0.08, 0.18) | 0.61 (0.40, 0.88) |  | 0.38 (0.27, 0.55) | 0.84 (0.59, 1.20) |  | 201.21 | 1.28 (0.89, 1.67) |
| Andorra | 0.27 (0.16, 0.44) | 0.53 (0.32, 0.84) |  | 0.91 (0.56, 1.42) | 0.65 (0.40, 1.01) |  | 236.18 | 0.73 (0.62, 0.84) |
| Angola | 5.64 (3.61, 8.48) | 0.16 (0.10, 0.24) |  | 18.58 (12.28, 27.89) | 0.18 (0.12, 0.27) |  | 229.26 | 0.29 (0.20, 0.37) |
| Antigua and Barbuda | 0.35 (0.24, 0.51) | 0.63 (0.44, 0.90) |  | 0.27 (0.18, 0.38) | 0.28 (0.19, 0.40) |  | -24.59 | -3.10 (-4.11, -2.08) |
| Argentina | 35.68 (23.57, 53.50) | 0.11 (0.08, 0.17) |  | 109.85 (74.97, 157.87) | 0.20 (0.14, 0.28) |  | 207.87 | 2.74 (2.50, 2.99) |
| Armenia | 1.61 (1.09, 2.29) | 0.07 (0.05, 0.10) |  | 23.18 (15.54, 33.63) | 0.57 (0.39, 0.83) |  | 1343.80 | 10.87 (9.17, 12.59) |
| Australia | 29.45 (20.76, 41.73) | 0.15 (0.11, 0.21) |  | 193.10 (133.81, 264.45) | 0.45 (0.32, 0.62) |  | 555.71 | 4.19 (3.88, 4.50) |
| Austria | 17.06 (11.60, 25.31) | 0.14 (0.10, 0.20) |  | 40.92 (26.69, 61.54) | 0.22 (0.15, 0.33) |  | 139.86 | 1.94 (1.61, 2.27) |
| Azerbaijan | 2.91 (1.98, 4.22) | 0.06 (0.04, 0.09) |  | 28.04 (17.76, 43.85) | 0.37 (0.23, 0.60) |  | 862.15 | 7.29 (5.26, 9.36) |
| Bahamas | 0.99 (0.70, 1.38) | 0.66 (0.46, 0.92) |  | 1.19 (0.80, 1.72) | 0.32 (0.22, 0.47) |  | 20.34 | -2.73 (-3.56, -1.89) |
| Bahrain | 0.90 (0.61, 1.31) | 0.68 (0.45, 1.02) |  | 4.91 (3.20, 7.41) | 0.77 (0.51, 1.16) |  | 445.64 | -0.25 (-0.65, 0.15) |
| Bangladesh | 110.70 (77.10, 154.60) | 0.21 (0.15, 0.30) |  | 273.62 (180.95, 402.32) | 0.22 (0.14, 0.32) |  | 147.16 | -0.28 (-0.42, -0.13) |
| Barbados | 0.83 (0.53, 1.26) | 0.27 (0.17, 0.40) |  | 1.46 (1.00, 2.12) | 0.29 (0.20, 0.43) |  | 75.05 | 0.26 (0.07, 0.45) |
| Belarus | 11.62 (8.06, 16.49) | 0.09 (0.06, 0.13) |  | 28.88 (18.15, 44.21) | 0.18 (0.11, 0.28) |  | 148.51 | 3.11 (2.61, 3.61) |
| Belgium | 27.79 (18.26, 40.27) | 0.17 (0.12, 0.25) |  | 57.80 (38.49, 83.67) | 0.24 (0.16, 0.35) |  | 108.00 | 0.89 (0.48, 1.30) |
| Belize | 0.51 (0.35, 0.72) | 0.56 (0.38, 0.79) |  | 0.84 (0.58, 1.16) | 0.32 (0.22, 0.46) |  | 63.19 | -2.01 (-2.51, -1.52) |
| Benin | 10.21 (6.78, 14.80) | 0.53 (0.35, 0.77) |  | 23.94 (15.97, 36.22) | 0.53 (0.35, 0.80) |  | 134.44 | -0.21 (-0.32, -0.10) |
| Bermuda | 0.43 (0.30, 0.61) | 0.71 (0.49, 1.00) |  | 0.30 (0.20, 0.44) | 0.22 (0.15, 0.32) |  | -30.24 | -4.08 (-5.13, -3.01) |
| Bhutan | 0.41 (0.24, 0.67) | 0.18 (0.11, 0.28) |  | 1.71 (1.02, 2.66) | 0.32 (0.19, 0.51) |  | 313.09 | 2.37 (2.22, 2.51) |
| Bolivia | 13.05 (7.92, 22.29) | 0.46 (0.28, 0.75) |  | 42.29 (26.40, 66.54) | 0.53 (0.34, 0.83) |  | 224.15 | 0.46 (0.38, 0.54) |
| Bosnia and Herzegovina | 15.39 (10.47, 22.42) | 0.41 (0.28, 0.59) |  | 47.03 (30.95, 69.61) | 0.78 (0.52, 1.15) |  | 205.53 | 2.99 (2.48, 3.50) |
| Botswana | 0.48 (0.21, 1.05) | 0.09 (0.04, 0.20) |  | 2.07 (1.27, 3.13) | 0.16 (0.10, 0.24) |  | 328.15 | 1.23 (0.56, 1.92) |
| Brazil | 100.99 (86.17, 117.78) | 0.12 (0.10, 0.14) |  | 373.92 (315.56, 440.38) | 0.16 (0.14, 0.19) |  | 270.27 | 1.57 (1.35, 1.79) |
| Brunei | 0.44 (0.28, 0.65) | 0.57 (0.37, 0.85) |  | 1.78 (1.17, 2.67) | 0.71 (0.47, 1.04) |  | 310.14 | 1.38 (0.86, 1.92) |
| Bulgaria | 57.10 (39.01, 80.85) | 0.46 (0.32, 0.65) |  | 50.49 (33.00, 73.56) | 0.35 (0.23, 0.50) |  | -11.57 | -0.52 (-1.06, 0.03) |
| Burkina Faso | 8.72 (5.79, 12.79) | 0.22 (0.15, 0.33) |  | 17.88 (11.61, 26.26) | 0.21 (0.14, 0.31) |  | 105.17 | -0.32 (-0.52, -0.12) |
| Burundi | 9.39 (5.90, 14.29) | 0.42 (0.27, 0.65) |  | 15.16 (9.25, 24.64) | 0.37 (0.23, 0.60) |  | 61.51 | -0.90 (-1.08, -0.72) |
| Cambodia | 29.79 (20.23, 43.08) | 0.64 (0.42, 0.93) |  | 74.58 (48.94, 109.94) | 0.66 (0.43, 0.97) |  | 150.36 | -0.16 (-0.28, -0.04) |
| Cameroon | 2.53 (1.53, 4.01) | 0.06 (0.04, 0.10) |  | 7.84 (4.89, 12.24) | 0.07 (0.05, 0.11) |  | 209.64 | 0.26 (0.06, 0.46) |
| Canada | 66.19 (47.44, 92.93) | 0.20 (0.15, 0.28) |  | 354.58 (247.18, 499.12) | 0.50 (0.36, 0.70) |  | 435.67 | 3.53 (3.31, 3.76) |
| Cape Verde | 0.25 (0.17, 0.37) | 0.11 (0.07, 0.15) |  | 5.77 (4.03, 8.47) | 1.41 (0.96, 2.09) |  | 2165.84 | 7.47 (5.18, 9.82) |
| Central African Republic | 2.59 (1.57, 4.12) | 0.24 (0.14, 0.37) |  | 4.70 (2.70, 7.91) | 0.24 (0.14, 0.39) |  | 81.91 | -0.41 (-0.64, -0.19) |
| Chad | 13.39 (8.64, 19.79) | 0.49 (0.31, 0.72) |  | 24.88 (15.82, 37.03) | 0.48 (0.31, 0.72) |  | 85.77 | -0.21 (-0.29, -0.12) |
| Chile | 17.31 (12.01, 24.32) | 0.18 (0.13, 0.26) |  | 77.28 (52.93, 109.68) | 0.32 (0.22, 0.45) |  | 346.52 | 2.62 (2.38, 2.85) |
| China | 8866.13 (7044.40, 11079.87) | 1.06 (0.85, 1.31) |  | 9401.11 (7548.90, 11639.83) | 0.48 (0.39, 0.59) |  | 6.03 | -4.20 (-5.05, -3.35) |
| Colombia | 31.25 (22.02, 44.25) | 0.19 (0.13, 0.27) |  | 110.30 (70.06, 168.33) | 0.21 (0.13, 0.32) |  | 252.97 | 0.57 (0.09, 1.05) |
| Comoros | 0.92 (0.49, 1.60) | 0.44 (0.25, 0.75) |  | 2.12 (1.30, 3.37) | 0.46 (0.28, 0.74) |  | 129.69 | -0.04 (-0.18, 0.10) |
| Congo | 2.83 (1.75, 4.35) | 0.28 (0.18, 0.43) |  | 6.01 (3.63, 9.35) | 0.26 (0.16, 0.39) |  | 112.37 | -0.74 (-0.90, -0.58) |
| Cook Islands | 0.15 (0.11, 0.22) | 1.28 (0.88, 1.81) |  | 0.33 (0.22, 0.46) | 1.34 (0.91, 1.87) |  | 113.40 | 0.08 (-0.05, 0.20) |
| Costa Rica | 7.04 (4.85, 9.84) | 0.41 (0.28, 0.57) |  | 24.36 (15.48, 36.59) | 0.48 (0.31, 0.72) |  | 246.06 | 0.34 (-0.21, 0.89) |
| Cote d'Ivoire | 20.43 (13.35, 30.06) | 0.58 (0.37, 0.87) |  | 45.12 (29.08, 68.70) | 0.49 (0.32, 0.76) |  | 120.78 | -1.23 (-1.49, -0.98) |
| Croatia | 17.71 (12.21, 24.77) | 0.29 (0.20, 0.40) |  | 31.01 (19.84, 46.19) | 0.34 (0.22, 0.50) |  | 75.06 | 0.85 (0.29, 1.41) |
| Cuba | 61.49 (42.87, 85.27) | 0.59 (0.41, 0.82) |  | 48.86 (31.95, 74.23) | 0.25 (0.16, 0.38) |  | -20.54 | -3.31 (-4.60, -2.00) |
| Cyprus | 1.45 (0.92, 2.15) | 0.19 (0.13, 0.28) |  | 4.23 (2.68, 6.24) | 0.23 (0.15, 0.33) |  | 191.35 | 0.79 (0.49, 1.09) |
| Czech Republic | 40.75 (28.09, 59.15) | 0.29 (0.20, 0.42) |  | 48.21 (31.35, 71.83) | 0.22 (0.15, 0.33) |  | 18.31 | -1.20 (-1.42, -0.99) |
| Democratic Republic of the Congo | 23.88 (15.65, 35.48) | 0.16 (0.11, 0.24) |  | 55.44 (36.13, 85.50) | 0.16 (0.10, 0.24) |  | 132.14 | -0.25 (-0.33, -0.18) |
| Denmark | 8.94 (5.87, 13.18) | 0.11 (0.07, 0.15) |  | 23.77 (15.72, 34.78) | 0.20 (0.14, 0.29) |  | 166.07 | 2.62 (2.35, 2.89) |
| Djibouti | 0.50 (0.27, 0.86) | 0.40 (0.22, 0.70) |  | 2.45 (1.36, 4.35) | 0.48 (0.28, 0.80) |  | 391.47 | 0.52 (0.41, 0.63) |
| Dominica | 0.59 (0.40, 0.84) | 0.78 (0.54, 1.10) |  | 0.31 (0.21, 0.45) | 0.34 (0.23, 0.49) |  | -48.08 | -3.02 (-3.81, -2.23) |
| Dominican Republic | 11.13 (7.76, 15.80) | 0.31 (0.21, 0.44) |  | 40.63 (25.21, 63.05) | 0.45 (0.28, 0.70) |  | 265.11 | 1.69 (1.14, 2.25) |
| Ecuador | 16.13 (11.20, 22.53) | 0.33 (0.23, 0.46) |  | 68.07 (44.70, 98.73) | 0.49 (0.32, 0.71) |  | 322.02 | 1.75 (1.41, 2.10) |
| Egypt | 324.85 (212.05, 491.66) | 1.12 (0.72, 1.73) |  | 1232.68 (736.17, 1990.92) | 1.96 (1.18, 3.09) |  | 279.46 | 2.65 (2.32, 2.99) |
| El Salvador | 7.73 (5.40, 11.16) | 0.26 (0.18, 0.38) |  | 11.78 (7.36, 18.25) | 0.19 (0.12, 0.30) |  | 52.45 | -1.14 (-1.85, -0.43) |
| Equatorial Guinea | 0.37 (0.22, 0.60) | 0.20 (0.12, 0.32) |  | 1.40 (0.78, 2.28) | 0.32 (0.17, 0.52) |  | 276.37 | 1.73 (1.61, 1.86) |
| Eritrea | 3.48 (2.01, 5.85) | 0.40 (0.23, 0.68) |  | 10.22 (6.36, 16.52) | 0.45 (0.27, 0.71) |  | 194.05 | 0.12 (-0.08, 0.32) |
| Estonia | 3.22 (2.19, 4.66) | 0.16 (0.11, 0.22) |  | 8.37 (5.32, 12.76) | 0.30 (0.19, 0.45) |  | 160.21 | 1.46 (0.91, 2.01) |
| Eswatini | 1.63 (0.93, 3.08) | 0.59 (0.33, 1.07) |  | 10.62 (3.29, 19.92) | 1.87 (0.62, 3.44) |  | 552.34 | 4.79 (3.48, 6.12) |
| Ethiopia | 54.38 (35.81, 80.08) | 0.29 (0.20, 0.41) |  | 117.88 (91.26, 155.16) | 0.31 (0.24, 0.41) |  | 116.76 | -0.02 (-0.13, 0.09) |
| Federated States of Micronesia | 0.42 (0.26, 0.65) | 0.96 (0.61, 1.49) |  | 0.69 (0.39, 1.10) | 1.08 (0.65, 1.67) |  | 64.75 | 0.19 (0.09, 0.29) |
| Fiji | 1.71 (1.11, 2.52) | 0.51 (0.33, 0.75) |  | 4.59 (2.96, 6.83) | 0.67 (0.43, 0.98) |  | 168.78 | 1.22 (0.98, 1.46) |
| Finland | 12.81 (8.47, 18.86) | 0.17 (0.12, 0.25) |  | 35.66 (23.36, 52.69) | 0.27 (0.18, 0.40) |  | 178.45 | 1.81 (1.66, 1.96) |
| France | 202.74 (137.18, 302.18) | 0.24 (0.16, 0.36) |  | 508.59 (333.04, 744.28) | 0.36 (0.24, 0.53) |  | 150.86 | 1.08 (0.79, 1.37) |
| Gabon | 1.19 (0.75, 1.80) | 0.23 (0.15, 0.34) |  | 2.82 (1.70, 4.36) | 0.30 (0.17, 0.47) |  | 138.19 | 0.69 (0.49, 0.89) |
| Gambia | 7.50 (4.81, 11.08) | 2.21 (1.42, 3.22) |  | 32.52 (20.77, 48.81) | 3.43 (2.23, 5.16) |  | 333.39 | 1.23 (1.00, 1.47) |
| Georgia | 5.03 (3.39, 7.28) | 0.09 (0.06, 0.13) |  | 15.44 (10.51, 22.45) | 0.26 (0.18, 0.37) |  | 206.98 | 2.96 (1.85, 4.09) |
| Germany | 229.78 (159.87, 323.14) | 0.17 (0.12, 0.24) |  | 673.09 (458.32, 973.09) | 0.33 (0.23, 0.46) |  | 192.93 | 2.57 (2.08, 3.06) |
| Ghana | 24.39 (15.56, 37.37) | 0.43 (0.27, 0.65) |  | 76.17 (50.72, 111.86) | 0.51 (0.34, 0.76) |  | 212.27 | 0.33 (0.10, 0.57) |
| Greece | 25.45 (16.68, 37.08) | 0.17 (0.11, 0.24) |  | 65.31 (43.03, 95.92) | 0.24 (0.16, 0.34) |  | 156.62 | 1.46 (1.32, 1.59) |
| Greenland | 0.14 (0.09, 0.20) | 0.43 (0.30, 0.62) |  | 0.44 (0.28, 0.65) | 0.67 (0.44, 0.95) |  | 212.20 | 1.31 (1.12, 1.49) |
| Grenada | 0.54 (0.37, 0.77) | 0.71 (0.49, 1.01) |  | 0.30 (0.20, 0.43) | 0.29 (0.19, 0.41) |  | -44.75 | -3.36 (-4.36, -2.36) |
| Guam | 0.25 (0.17, 0.36) | 0.38 (0.26, 0.55) |  | 1.08 (0.72, 1.58) | 0.57 (0.39, 0.84) |  | 333.79 | 1.83 (1.63, 2.03) |
| Guatemala | 23.72 (16.63, 33.69) | 0.71 (0.48, 1.01) |  | 45.96 (29.97, 67.30) | 0.43 (0.28, 0.64) |  | 93.73 | -2.65 (-4.21, -1.08) |
| Guinea | 71.36 (47.40, 103.35) | 2.28 (1.53, 3.31) |  | 143.62 (91.34, 217.89) | 2.75 (1.73, 4.23) |  | 101.26 | 0.68 (0.63, 0.73) |
| Guinea-Bissau | 2.36 (1.45, 3.77) | 0.62 (0.38, 0.95) |  | 3.98 (2.44, 6.21) | 0.61 (0.37, 0.94) |  | 68.83 | -0.24 (-0.31, -0.17) |
| Guyana | 1.88 (1.30, 2.66) | 0.52 (0.36, 0.75) |  | 1.74 (1.15, 2.60) | 0.30 (0.20, 0.44) |  | -7.43 | -1.98 (-2.81, -1.14) |
| Haiti | 14.23 (7.75, 23.13) | 0.49 (0.27, 0.82) |  | 19.96 (10.18, 33.03) | 0.32 (0.17, 0.52) |  | 40.34 | -1.51 (-1.75, -1.26) |
| Honduras | 15.72 (5.06, 27.64) | 0.79 (0.24, 1.42) |  | 78.07 (29.88, 137.08) | 1.38 (0.52, 2.42) |  | 396.73 | 2.29 (2.09, 2.49) |
| Hungary | 71.90 (50.28, 101.91) | 0.49 (0.35, 0.69) |  | 44.29 (29.51, 63.70) | 0.22 (0.15, 0.31) |  | -38.40 | -2.16 (-2.97, -1.34) |
| Iceland | 0.37 (0.25, 0.53) | 0.13 (0.09, 0.18) |  | 1.15 (0.76, 1.68) | 0.20 (0.13, 0.30) |  | 213.85 | 1.64 (1.45, 1.82) |
| India | 1136.84 (897.70, 1403.75) | 0.29 (0.23, 0.36) |  | 3519.57 (2776.52, 4360.99) | 0.33 (0.26, 0.41) |  | 209.59 | 0.34 (0.23, 0.45) |
| Indonesia | 212.88 (170.46, 261.30) | 0.23 (0.18, 0.28) |  | 462.11 (372.18, 563.72) | 0.25 (0.20, 0.29) |  | 117.07 | 0.17 (0.11, 0.22) |
| Iran | 118.80 (89.80, 152.47) | 0.57 (0.42, 0.75) |  | 353.75 (296.66, 423.13) | 0.54 (0.45, 0.64) |  | 197.76 | -0.65 (-1.34, 0.04) |
| Iraq | 32.93 (21.65, 48.41) | 0.44 (0.29, 0.65) |  | 144.46 (92.75, 213.57) | 0.69 (0.45, 1.01) |  | 338.73 | 1.84 (1.34, 2.35) |
| Ireland | 3.95 (2.64, 5.79) | 0.09 (0.07, 0.14) |  | 19.28 (12.61, 28.28) | 0.25 (0.17, 0.37) |  | 388.17 | 4.18 (3.80, 4.55) |
| Israel | 9.92 (6.43, 14.72) | 0.20 (0.13, 0.29) |  | 27.01 (18.18, 38.92) | 0.23 (0.16, 0.33) |  | 172.22 | 0.30 (0.17, 0.44) |
| Italy | 270.40 (227.46, 321.75) | 0.30 (0.25, 0.35) |  | 341.45 (274.50, 414.21) | 0.22 (0.18, 0.27) |  | 26.27 | -1.47 (-1.76, -1.18) |
| Jamaica | 6.02 (4.18, 8.62) | 0.33 (0.23, 0.46) |  | 8.24 (5.49, 12.40) | 0.28 (0.18, 0.42) |  | 36.80 | 0.13 (-0.82, 1.08) |
| Japan | 805.71 (682.87, 945.98) | 0.47 (0.40, 0.55) |  | 1649.84 (1259.48, 2029.69) | 0.40 (0.32, 0.48) |  | 104.77 | -1.36 (-1.97, -0.74) |
| Jordan | 3.86 (2.52, 5.78) | 0.34 (0.21, 0.51) |  | 17.76 (11.71, 26.48) | 0.32 (0.21, 0.48) |  | 360.36 | -0.34 (-0.49, -0.18) |
| Kazakhstan | 26.34 (18.33, 37.72) | 0.22 (0.15, 0.31) |  | 85.05 (57.02, 124.51) | 0.52 (0.35, 0.77) |  | 222.87 | 0.19 (-0.84, 1.22) |
| Kenya | 29.21 (19.99, 49.76) | 0.38 (0.26, 0.64) |  | 106.68 (71.80, 157.95) | 0.53 (0.36, 0.77) |  | 265.26 | 0.78 (0.48, 1.09) |
| Kiribati | 0.41 (0.27, 0.62) | 1.13 (0.74, 1.69) |  | 0.77 (0.51, 1.16) | 1.17 (0.77, 1.76) |  | 87.43 | 0.01 (-0.14, 0.17) |
| Kuwait | 1.66 (1.14, 2.33) | 0.31 (0.21, 0.44) |  | 7.30 (4.79, 10.85) | 0.35 (0.23, 0.53) |  | 340.66 | 1.43 (1.06, 1.81) |
| Kyrgyzstan | 2.25 (1.56, 3.25) | 0.08 (0.05, 0.11) |  | 7.37 (5.03, 10.63) | 0.18 (0.12, 0.26) |  | 227.54 | 3.71 (3.39, 4.02) |
| Lao | 14.61 (8.46, 23.68) | 0.74 (0.43, 1.16) |  | 23.35 (14.55, 34.54) | 0.58 (0.37, 0.85) |  | 59.80 | -1.09 (-1.23, -0.95) |
| Latvia | 4.54 (3.12, 6.47) | 0.13 (0.09, 0.18) |  | 9.26 (6.03, 13.74) | 0.22 (0.15, 0.33) |  | 104.02 | 1.21 (0.47, 1.97) |
| Lebanon | 5.72 (3.75, 8.43) | 0.28 (0.18, 0.40) |  | 15.26 (9.62, 24.38) | 0.29 (0.18, 0.47) |  | 166.59 | 0.17 (0.05, 0.29) |
| Lesotho | 4.26 (2.30, 8.35) | 0.46 (0.25, 0.88) |  | 16.88 (7.11, 28.90) | 1.40 (0.62, 2.34) |  | 296.53 | 4.38 (3.75, 5.00) |
| Liberia | 5.94 (3.88, 8.89) | 0.56 (0.37, 0.84) |  | 10.12 (6.46, 15.59) | 0.55 (0.35, 0.85) |  | 70.31 | -0.17 (-0.35, 0.01) |
| Libya | 10.51 (6.65, 15.90) | 0.60 (0.38, 0.91) |  | 32.69 (21.67, 50.69) | 0.69 (0.44, 1.06) |  | 211.01 | 0.63 (0.44, 0.81) |
| Lithuania | 5.44 (3.79, 7.66) | 0.12 (0.08, 0.17) |  | 13.26 (8.54, 19.35) | 0.22 (0.14, 0.32) |  | 143.67 | 1.48 (1.11, 1.84) |
| Luxembourg | 0.95 (0.63, 1.41) | 0.17 (0.11, 0.25) |  | 2.45 (1.56, 3.67) | 0.24 (0.15, 0.35) |  | 157.21 | 1.20 (0.93, 1.47) |
| Macedonia | 11.77 (8.13, 16.47) | 0.67 (0.46, 0.95) |  | 24.36 (15.88, 36.06) | 0.79 (0.52, 1.16) |  | 107.00 | 0.60 (0.48, 0.72) |
| Madagascar | 17.14 (10.49, 27.98) | 0.35 (0.21, 0.60) |  | 33.38 (20.10, 52.97) | 0.34 (0.20, 0.54) |  | 94.80 | -0.24 (-0.35, -0.12) |
| Malawi | 14.27 (9.16, 22.10) | 0.40 (0.25, 0.60) |  | 26.04 (17.11, 37.98) | 0.40 (0.27, 0.58) |  | 82.51 | -0.67 (-1.02, -0.32) |
| Malaysia | 43.48 (29.65, 61.96) | 0.51 (0.34, 0.74) |  | 179.76 (116.83, 266.73) | 0.73 (0.48, 1.08) |  | 313.43 | 1.42 (1.02, 1.82) |
| Maldives | 0.39 (0.22, 0.69) | 0.53 (0.31, 0.91) |  | 1.51 (1.01, 2.23) | 0.56 (0.37, 0.86) |  | 284.80 | 0.34 (0.17, 0.51) |
| Mali | 37.64 (25.15, 55.10) | 0.91 (0.61, 1.33) |  | 91.16 (58.10, 139.75) | 1.06 (0.68, 1.59) |  | 142.20 | 0.41 (0.33, 0.49) |
| Malta | 0.44 (0.29, 0.65) | 0.10 (0.07, 0.15) |  | 1.52 (0.99, 2.24) | 0.16 (0.11, 0.23) |  | 245.64 | 1.47 (1.09, 1.86) |
| Marshall Islands | 0.14 (0.09, 0.21) | 0.89 (0.56, 1.36) |  | 0.29 (0.18, 0.45) | 0.92 (0.59, 1.40) |  | 109.62 | 0.01 (-0.09, 0.12) |
| Mauritania | 6.50 (4.36, 9.50) | 0.68 (0.46, 1.00) |  | 10.62 (6.75, 15.56) | 0.55 (0.35, 0.81) |  | 63.45 | -0.79 (-0.88, -0.70) |
| Mauritius | 1.22 (0.84, 1.73) | 0.18 (0.12, 0.25) |  | 3.95 (2.55, 6.01) | 0.24 (0.16, 0.36) |  | 224.22 | 2.35 (1.93, 2.78) |
| Mexico | 82.51 (70.96, 96.07) | 0.20 (0.18, 0.24) |  | 430.51 (348.57, 524.52) | 0.38 (0.31, 0.47) |  | 421.74 | 2.30 (2.14, 2.47) |
| Moldova | 3.58 (2.51, 5.12) | 0.09 (0.06, 0.12) |  | 9.37 (6.44, 13.46) | 0.16 (0.11, 0.23) |  | 161.69 | 0.57 (-0.85, 2.01) |
| Monaco | 0.18 (0.11, 0.27) | 0.23 (0.15, 0.36) |  | 0.63 (0.39, 0.97) | 0.63 (0.40, 0.96) |  | 257.35 | 4.13 (3.22, 5.05) |
| Mongolia | 34.85 (22.38, 52.51) | 3.51 (2.30, 5.19) |  | 154.50 (97.81, 231.79) | 8.72 (5.64, 12.87) |  | 343.38 | 4.23 (3.58, 4.88) |
| Montenegro | 2.70 (1.80, 3.87) | 0.45 (0.30, 0.64) |  | 5.07 (3.38, 7.45) | 0.52 (0.35, 0.75) |  | 87.50 | 0.44 (0.24, 0.65) |
| Morocco | 23.24 (14.27, 35.07) | 0.20 (0.12, 0.30) |  | 75.60 (48.96, 112.40) | 0.28 (0.18, 0.41) |  | 225.36 | 1.11 (0.83, 1.40) |
| Mozambique | 13.38 (8.34, 21.15) | 0.26 (0.16, 0.43) |  | 44.20 (28.81, 65.79) | 0.47 (0.31, 0.70) |  | 230.38 | 1.90 (1.71, 2.10) |
| Myanmar | 55.79 (34.45, 87.75) | 0.27 (0.17, 0.43) |  | 203.55 (136.56, 298.09) | 0.48 (0.33, 0.71) |  | 264.87 | 2.01 (1.80, 2.23) |
| Namibia | 1.21 (0.67, 2.23) | 0.18 (0.10, 0.32) |  | 4.33 (2.81, 6.46) | 0.32 (0.21, 0.47) |  | 257.13 | 2.01 (1.61, 2.40) |
| Nauru | 0.03 (0.02, 0.05) | 0.91 (0.59, 1.34) |  | 0.04 (0.02, 0.06) | 0.94 (0.60, 1.45) |  | 13.76 | -0.25 (-0.50, -0.01) |
| Nepal | 13.33 (8.87, 20.37) | 0.16 (0.10, 0.24) |  | 47.63 (30.16, 74.15) | 0.24 (0.15, 0.37) |  | 257.28 | 1.44 (1.25, 1.63) |
| Netherlands | 20.13 (13.76, 28.88) | 0.10 (0.07, 0.14) |  | 79.62 (54.33, 113.38) | 0.23 (0.16, 0.32) |  | 295.55 | 3.26 (3.08, 3.44) |
| New Zealand | 6.63 (5.51, 7.86) | 0.17 (0.14, 0.20) |  | 31.20 (26.37, 36.87) | 0.40 (0.34, 0.47) |  | 370.73 | 3.12 (2.86, 3.37) |
| Nicaragua | 3.27 (2.30, 4.59) | 0.21 (0.15, 0.31) |  | 14.43 (9.64, 20.72) | 0.35 (0.24, 0.51) |  | 341.85 | 1.94 (1.64, 2.24) |
| Niger | 1.22 (0.79, 1.85) | 0.05 (0.03, 0.07) |  | 3.64 (2.24, 5.52) | 0.05 (0.03, 0.08) |  | 198.93 | 0.08 (-0.01, 0.16) |
| Nigeria | 131.63 (93.94, 176.76) | 0.33 (0.23, 0.44) |  | 287.26 (217.60, 374.53) | 0.38 (0.30, 0.49) |  | 118.24 | 0.57 (0.51, 0.63) |
| Niue | 0.02 (0.01, 0.02) | 0.73 (0.48, 1.10) |  | 0.02 (0.01, 0.03) | 0.81 (0.52, 1.19) |  | 6.59 | 0.30 (0.18, 0.42) |
| North Korea | 110.28 (69.93, 169.48) | 0.71 (0.45, 1.08) |  | 161.65 (99.78, 250.07) | 0.51 (0.31, 0.79) |  | 46.59 | -1.39 (-1.51, -1.27) |
| Northern Mariana Islands | 0.12 (0.08, 0.17) | 0.70 (0.48, 1.02) |  | 0.39 (0.24, 0.59) | 0.80 (0.54, 1.15) |  | 235.61 | 0.40 (0.29, 0.51) |
| Norway | 6.73 (5.58, 8.03) | 0.10 (0.08, 0.11) |  | 16.37 (13.44, 19.89) | 0.17 (0.14, 0.20) |  | 143.09 | 2.41 (2.23, 2.58) |
| Oman | 2.02 (1.17, 3.24) | 0.34 (0.19, 0.54) |  | 8.80 (6.08, 12.72) | 0.59 (0.40, 0.87) |  | 335.32 | 2.92 (2.49, 3.36) |
| Pakistan | 142.46 (93.02, 203.21) | 0.25 (0.16, 0.36) |  | 314.07 (242.94, 410.57) | 0.29 (0.22, 0.38) |  | 120.47 | 0.46 (0.34, 0.57) |
| Palau | 0.06 (0.04, 0.10) | 0.68 (0.40, 1.07) |  | 0.18 (0.11, 0.28) | 0.88 (0.56, 1.30) |  | 178.47 | 0.82 (0.61, 1.03) |
| Palestine | 6.76 (4.16, 10.50) | 0.85 (0.52, 1.31) |  | 14.12 (9.61, 20.40) | 0.70 (0.48, 1.02) |  | 108.83 | -0.69 (-0.76, -0.63) |
| Panama | 3.36 (2.34, 4.79) | 0.23 (0.15, 0.33) |  | 10.44 (6.56, 15.55) | 0.25 (0.16, 0.38) |  | 210.92 | 0.69 (0.27, 1.10) |
| Papua New Guinea | 1.45 (0.90, 2.29) | 0.10 (0.06, 0.16) |  | 4.50 (2.87, 6.86) | 0.13 (0.08, 0.20) |  | 210.10 | 0.82 (0.76, 0.88) |
| Paraguay | 2.67 (1.80, 3.91) | 0.12 (0.08, 0.18) |  | 6.54 (4.07, 10.01) | 0.12 (0.08, 0.19) |  | 144.80 | 0.19 (-0.39, 0.79) |
| Peru | 54.19 (36.72, 78.03) | 0.48 (0.32, 0.69) |  | 87.25 (54.21, 127.34) | 0.27 (0.17, 0.40) |  | 60.99 | -2.83 (-3.41, -2.24) |
| Philippines | 199.25 (150.16, 257.02) | 0.71 (0.53, 0.91) |  | 406.23 (320.92, 506.90) | 0.55 (0.43, 0.68) |  | 103.88 | -1.18 (-1.49, -0.86) |
| Poland | 265.54 (225.20, 311.19) | 0.63 (0.53, 0.74) |  | 124.32 (98.52, 156.67) | 0.17 (0.14, 0.22) |  | -53.18 | -4.38 (-5.79, -2.95) |
| Portugal | 14.80 (10.29, 21.52) | 0.11 (0.08, 0.15) |  | 67.96 (45.54, 98.13) | 0.28 (0.19, 0.40) |  | 359.17 | 2.99 (2.59, 3.40) |
| Puerto Rico | 26.53 (18.44, 37.40) | 0.72 (0.51, 1.01) |  | 27.02 (17.65, 40.43) | 0.36 (0.23, 0.54) |  | 1.83 | -1.29 (-2.12, -0.45) |
| Qatar | 1.55 (0.99, 2.32) | 2.23 (1.38, 3.44) |  | 11.88 (7.06, 19.14) | 2.53 (1.56, 3.92) |  | 664.98 | 0.73 (0.53, 0.94) |
| Romania | 32.06 (22.39, 46.17) | 0.12 (0.09, 0.17) |  | 88.16 (58.68, 129.20) | 0.23 (0.16, 0.34) |  | 174.98 | 3.04 (2.59, 3.48) |
| Russia | 217.73 (185.26, 258.63) | 0.12 (0.11, 0.15) |  | 587.90 (478.18, 727.56) | 0.25 (0.21, 0.31) |  | 170.01 | 3.01 (2.67, 3.36) |
| Rwanda | 15.54 (9.36, 24.47) | 0.57 (0.36, 0.88) |  | 31.53 (20.31, 45.88) | 0.58 (0.38, 0.82) |  | 102.82 | -0.53 (-0.78, -0.29) |
| Saint Kitts and Nevis | 0.46 (0.31, 0.65) | 1.19 (0.84, 1.68) |  | 0.23 (0.16, 0.34) | 0.40 (0.28, 0.58) |  | -48.63 | -3.77 (-4.79, -2.74) |
| Saint Lucia | 0.42 (0.29, 0.59) | 0.49 (0.34, 0.69) |  | 0.44 (0.31, 0.63) | 0.21 (0.15, 0.30) |  | 4.74 | -3.36 (-4.45, -2.25) |
| Saint Vincent and the Grenadines | 0.43 (0.30, 0.61) | 0.60 (0.42, 0.85) |  | 0.38 (0.26, 0.54) | 0.29 (0.20, 0.41) |  | -12.47 | -2.32 (-3.27, -1.36) |
| Samoa | 0.42 (0.28, 0.64) | 0.50 (0.33, 0.75) |  | 0.65 (0.42, 0.96) | 0.46 (0.30, 0.68) |  | 54.34 | -0.50 (-0.58, -0.42) |
| San Marino | 0.04 (0.03, 0.07) | 0.13 (0.08, 0.19) |  | 0.13 (0.07, 0.22) | 0.19 (0.11, 0.32) |  | 194.21 | 1.93 (1.68, 2.19) |
| Sao Tome and Principe | 0.14 (0.09, 0.20) | 0.22 (0.15, 0.33) |  | 0.28 (0.17, 0.42) | 0.29 (0.18, 0.44) |  | 104.97 | 0.81 (0.69, 0.92) |
| Saudi Arabia | 31.99 (19.18, 48.41) | 0.62 (0.37, 0.94) |  | 102.13 (66.61, 154.39) | 0.76 (0.49, 1.15) |  | 219.23 | 0.41 (0.06, 0.76) |
| Senegal | 7.77 (5.11, 11.09) | 0.26 (0.17, 0.38) |  | 19.95 (12.82, 28.92) | 0.29 (0.19, 0.43) |  | 156.72 | 0.43 (0.30, 0.56) |
| Serbia | 40.51 (26.22, 60.35) | 0.39 (0.25, 0.56) |  | 64.88 (41.19, 96.65) | 0.40 (0.26, 0.58) |  | 60.14 | -0.04 (-0.28, 0.19) |
| Seychelles | 0.42 (0.30, 0.59) | 0.75 (0.52, 1.04) |  | 0.65 (0.45, 0.92) | 0.63 (0.43, 0.89) |  | 54.10 | -0.41 (-0.69, -0.12) |
| Sierra Leone | 8.59 (5.45, 12.81) | 0.47 (0.30, 0.69) |  | 15.70 (10.12, 23.36) | 0.47 (0.30, 0.71) |  | 82.88 | -0.01 (-0.08, 0.06) |
| Singapore | 6.51 (4.34, 9.70) | 0.33 (0.22, 0.49) |  | 32.31 (20.62, 48.62) | 0.44 (0.28, 0.67) |  | 395.95 | 1.19 (0.88, 1.51) |
| Slovakia | 17.93 (12.42, 24.73) | 0.30 (0.21, 0.41) |  | 24.40 (15.54, 36.96) | 0.26 (0.17, 0.40) |  | 36.05 | -1.07 (-1.44, -0.69) |
| Slovenia | 5.43 (3.45, 8.14) | 0.22 (0.14, 0.33) |  | 20.55 (12.46, 31.80) | 0.45 (0.28, 0.70) |  | 278.66 | 2.88 (2.51, 3.24) |
| Solomon Islands | 0.72 (0.45, 1.12) | 0.51 (0.33, 0.81) |  | 1.54 (1.03, 2.24) | 0.50 (0.34, 0.72) |  | 113.62 | -0.10 (-0.28, 0.09) |
| Somalia | 9.82 (5.34, 16.89) | 0.44 (0.24, 0.74) |  | 26.01 (15.09, 45.63) | 0.45 (0.26, 0.78) |  | 164.88 | 0.21 (0.12, 0.31) |
| South Africa | 131.86 (84.30, 217.10) | 0.63 (0.40, 1.06) |  | 311.38 (261.77, 372.78) | 0.73 (0.61, 0.87) |  | 136.15 | 0.04 (-0.57, 0.66) |
| South Korea | 149.09 (98.28, 214.15) | 0.56 (0.38, 0.81) |  | 837.29 (564.98, 1221.55) | 0.94 (0.64, 1.37) |  | 461.61 | 2.24 (1.06, 3.43) |
| South Sudan | 8.80 (4.98, 15.15) | 0.39 (0.22, 0.69) |  | 13.60 (7.22, 23.74) | 0.40 (0.22, 0.69) |  | 54.48 | 0.10 (0.06, 0.15) |
| Spain | 107.08 (71.21, 156.28) | 0.19 (0.13, 0.28) |  | 290.18 (191.33, 422.10) | 0.29 (0.19, 0.42) |  | 170.98 | 1.17 (0.78, 1.56) |
| Sri Lanka | 19.41 (13.32, 27.42) | 0.20 (0.14, 0.29) |  | 69.50 (42.79, 108.78) | 0.29 (0.18, 0.45) |  | 258.08 | 2.19 (1.88, 2.50) |
| Sudan | 33.63 (17.13, 55.53) | 0.39 (0.20, 0.64) |  | 89.09 (51.98, 144.47) | 0.54 (0.31, 0.86) |  | 164.93 | 1.19 (0.97, 1.42) |
| Suriname | 1.36 (0.94, 1.94) | 0.54 (0.38, 0.79) |  | 1.55 (1.03, 2.26) | 0.27 (0.18, 0.39) |  | 14.11 | -2.31 (-3.17, -1.45) |
| Sweden | 29.00 (23.91, 35.53) | 0.19 (0.15, 0.22) |  | 49.46 (40.23, 60.03) | 0.23 (0.19, 0.28) |  | 70.57 | 1.24 (0.63, 1.85) |
| Switzerland | 12.91 (8.66, 19.01) | 0.12 (0.08, 0.17) |  | 46.75 (30.43, 68.99) | 0.26 (0.17, 0.38) |  | 262.04 | 2.31 (1.78, 2.84) |
| Syrian Arab Republic | 25.16 (15.86, 38.04) | 0.53 (0.33, 0.83) |  | 59.77 (37.80, 91.48) | 0.55 (0.35, 0.83) |  | 137.58 | -0.05 (-0.23, 0.14) |
| Taiwan (Province of China) | 99.73 (69.01, 145.16) | 0.62 (0.43, 0.89) |  | 229.86 (147.66, 348.06) | 0.58 (0.38, 0.87) |  | 130.47 | -1.09 (-2.00, -0.17) |
| Tajikistan | 1.34 (0.93, 1.89) | 0.05 (0.03, 0.07) |  | 9.56 (6.41, 13.84) | 0.24 (0.15, 0.36) |  | 612.34 | 6.21 (5.85, 6.58) |
| Tanzania | 26.00 (18.11, 37.39) | 0.26 (0.18, 0.38) |  | 75.27 (51.90, 107.55) | 0.34 (0.23, 0.48) |  | 189.51 | 0.82 (0.67, 0.96) |
| Thailand | 536.06 (368.47, 758.16) | 1.61 (1.12, 2.29) |  | 2426.76 (1528.28, 3630.73) | 2.41 (1.52, 3.60) |  | 352.71 | 1.62 (1.45, 1.78) |
| Timor-Leste | 1.56 (0.93, 2.47) | 0.61 (0.36, 0.95) |  | 4.30 (2.56, 6.64) | 0.57 (0.34, 0.85) |  | 175.31 | -0.39 (-0.59, -0.19) |
| Togo | 6.11 (4.10, 9.00) | 0.53 (0.35, 0.78) |  | 17.09 (11.17, 26.43) | 0.53 (0.35, 0.81) |  | 179.68 | -0.48 (-0.63, -0.32) |
| Tokelau | 0.01 (0.01, 0.01) | 0.72 (0.47, 1.10) |  | 0.01 (0.01, 0.01) | 0.76 (0.49, 1.12) |  | -0.33 | -0.00 (-0.08, 0.08) |
| Tonga | 1.04 (0.63, 1.60) | 1.93 (1.17, 2.93) |  | 1.94 (1.23, 2.86) | 2.46 (1.56, 3.62) |  | 86.62 | 0.54 (0.15, 0.93) |
| Trinidad and Tobago | 5.27 (3.75, 7.47) | 0.65 (0.46, 0.92) |  | 5.34 (3.45, 8.11) | 0.30 (0.19, 0.45) |  | 1.44 | -2.85 (-3.93, -1.76) |
| Tunisia | 5.69 (3.63, 8.94) | 0.12 (0.08, 0.19) |  | 16.90 (9.88, 27.46) | 0.14 (0.08, 0.22) |  | 197.04 | 0.73 (0.60, 0.86) |
| Turkey | 109.77 (71.49, 163.72) | 0.33 (0.21, 0.50) |  | 285.79 (189.78, 413.75) | 0.34 (0.23, 0.49) |  | 160.36 | 0.13 (-0.18, 0.44) |
| Turkmenistan | 1.24 (0.86, 1.76) | 0.07 (0.05, 0.10) |  | 16.04 (10.37, 24.16) | 0.41 (0.27, 0.62) |  | 1197.26 | 6.93 (5.98, 7.89) |
| Tuvalu | 0.05 (0.03, 0.08) | 0.82 (0.51, 1.25) |  | 0.08 (0.05, 0.12) | 0.80 (0.51, 1.19) |  | 47.29 | -0.36 (-0.47, -0.25) |
| Uganda | 28.59 (19.20, 41.49) | 0.46 (0.31, 0.67) |  | 101.38 (69.00, 149.47) | 0.76 (0.50, 1.12) |  | 254.63 | 1.89 (1.67, 2.10) |
| Ukraine | 56.23 (47.00, 67.25) | 0.08 (0.07, 0.10) |  | 163.19 (128.75, 202.67) | 0.21 (0.17, 0.26) |  | 190.22 | 4.12 (3.67, 4.56) |
| United Arab Emirates | 1.58 (0.79, 2.94) | 0.47 (0.22, 0.95) |  | 20.16 (7.92, 50.97) | 0.64 (0.25, 1.62) |  | 1178.08 | 1.18 (0.80, 1.57) |
| United Kingdom | 110.58 (93.01, 131.31) | 0.12 (0.10, 0.14) |  | 397.88 (332.46, 471.42) | 0.30 (0.25, 0.35) |  | 259.83 | 3.88 (3.67, 4.09) |
| United States of America | 655.65 (563.61, 764.09) | 0.20 (0.17, 0.23) |  | 2533.76 (2080.78, 3046.75) | 0.45 (0.37, 0.54) |  | 286.45 | 2.96 (2.75, 3.17) |
| United States Virgin Islands | 0.35 (0.23, 0.50) | 0.44 (0.30, 0.63) |  | 0.61 (0.41, 0.89) | 0.33 (0.23, 0.47) |  | 74.43 | -1.12 (-1.64, -0.60) |
| Uruguay | 4.70 (3.18, 6.86) | 0.12 (0.08, 0.17) |  | 13.34 (9.15, 18.67) | 0.23 (0.16, 0.33) |  | 183.72 | 2.78 (2.61, 2.95) |
| Uzbekistan | 6.18 (4.26, 8.84) | 0.06 (0.04, 0.08) |  | 96.68 (63.49, 141.10) | 0.55 (0.37, 0.80) |  | 1463.99 | 10.13 (9.07, 11.20) |
| Vanuatu | 0.41 (0.23, 0.68) | 0.68 (0.37, 1.12) |  | 1.29 (0.77, 2.01) | 0.79 (0.47, 1.21) |  | 211.83 | 0.52 (0.41, 0.63) |
| Venezuela | 44.30 (30.90, 61.67) | 0.48 (0.33, 0.67) |  | 58.24 (37.72, 90.52) | 0.21 (0.13, 0.32) |  | 31.46 | -2.17 (-3.49, -0.82) |
| Viet Nam | 127.94 (79.08, 192.97) | 0.34 (0.21, 0.52) |  | 218.09 (139.41, 326.36) | 0.27 (0.17, 0.40) |  | 70.46 | -0.98 (-1.19, -0.77) |
| Yemen | 10.90 (5.72, 18.11) | 0.25 (0.13, 0.43) |  | 34.67 (21.44, 54.86) | 0.30 (0.18, 0.46) |  | 218.17 | 0.70 (0.58, 0.82) |
| Zambia | 8.20 (5.42, 12.53) | 0.32 (0.21, 0.50) |  | 24.22 (15.77, 36.16) | 0.41 (0.27, 0.60) |  | 195.21 | 0.23 (-0.09, 0.56) |
| Zimbabwe | 37.69 (23.79, 61.46) | 0.97 (0.62, 1.58) |  | 88.24 (56.50, 132.73) | 1.31 (0.84, 2.02) |  | 134.14 | 0.16 (-0.45, 0.77) |

ASMR: age-standardized mortality rate; CI: confidence interval; EAPC: estimated annual percentage change; NASH: nonalcoholic steatohepatitis; UI: uncertainty interval.

**
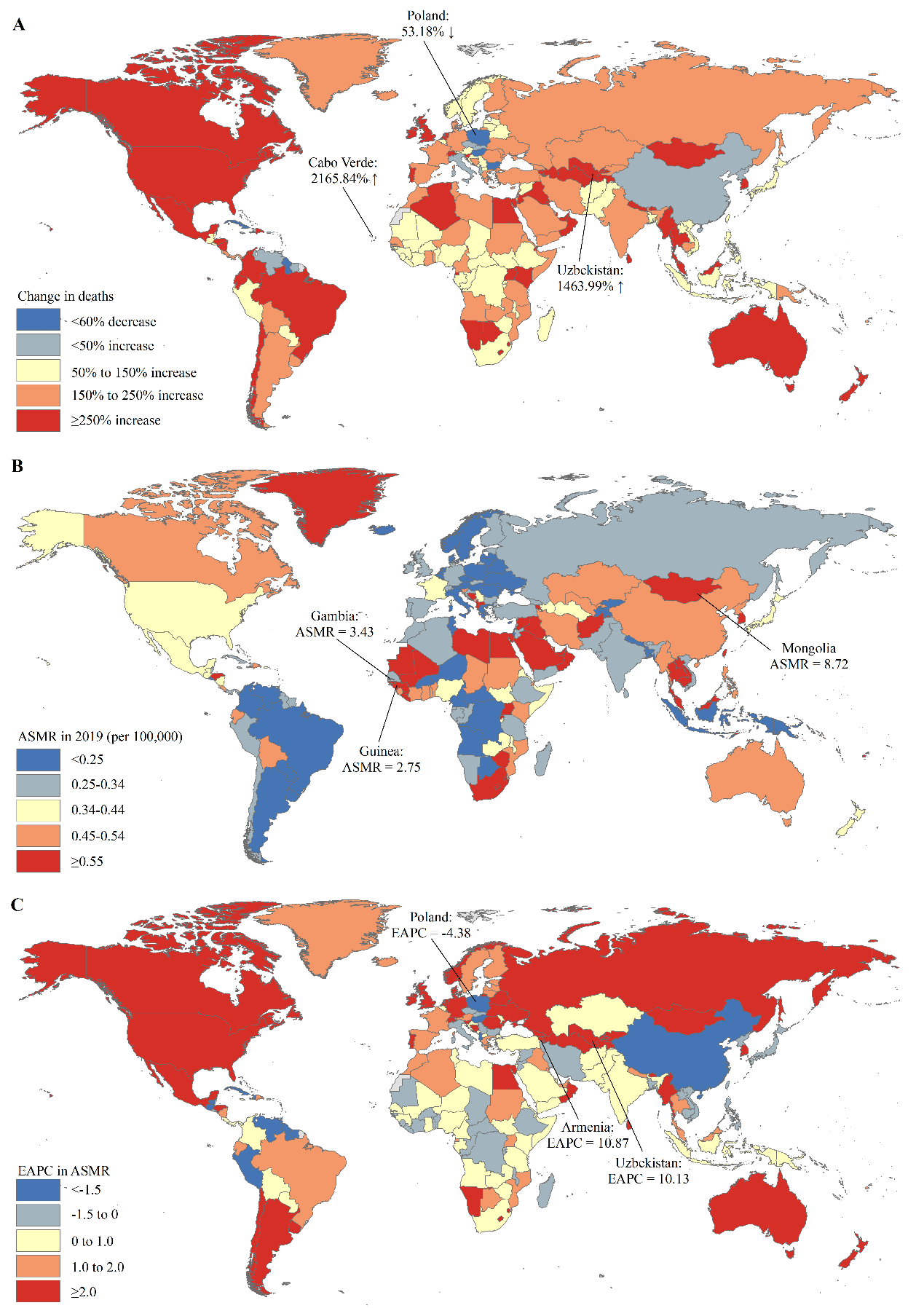
**

**Figure S9.** The global trends in the mortality of primary liver cancer due to NASH in 204 countries and territories. (A) The percentage change in deaths of primary liver cancer due to NASH between 1990 and 2019; (B) The ASMR of primary liver cancer due to NASH in 2019; (C) The EAPCs in ASMR of primary liver cancer due to NASH from 1990 to 2019.

Note: ASMR: age-standardized mortality rate; EAPC: estimated annual percentage change; NASH: nonalcoholic steatohepatitis.

**Table S23. The deaths and ASMR of primary liver cancer due to other causes in 1990 and 2019 and their change trends from 1990 to 2019 at national level.**

| Nation | 1990 | |  | 2019 | |  | 1990-2019 | |
| --- | --- | --- | --- | --- | --- | --- | --- | --- |
|  | Deaths  No. (95% UI) | ASMR per 100,000  No. (95% UI) |  | Deaths  No. x 10^3^ (95% UI) | ASMR per 100,000  No. (95% UI) |  | Percentage change in deaths  No. (%) | EAPC in ASMR  No. (95% CI) |
| Afghanistan | 67.12 (44.62, 97.02) | 0.83 (0.55, 1.22) |  | 136.37 (94.79, 194.06) | 0.75 (0.49, 1.10) |  | 103.16 | -0.37 (-0.49, -0.24) |
| Albania | 11.08 (8.08, 14.57) | 0.49 (0.34, 0.67) |  | 9.74 (5.90, 15.07) | 0.26 (0.17, 0.38) |  | -12.04 | -3.25 (-3.82, -2.69) |
| Algeria | 22.02 (16.58, 28.39) | 0.13 (0.09, 0.18) |  | 48.59 (33.52, 68.49) | 0.14 (0.10, 0.20) |  | 120.62 | 0.37 (0.24, 0.50) |
| American Samoa | 0.07 (0.05, 0.09) | 0.25 (0.16, 0.36) |  | 0.14 (0.09, 0.21) | 0.28 (0.19, 0.40) |  | 106.68 | 0.82 (0.59, 1.06) |
| Andorra | 0.22 (0.14, 0.34) | 0.43 (0.27, 0.65) |  | 0.57 (0.35, 0.87) | 0.43 (0.27, 0.66) |  | 152.84 | 0.12 (0.01, 0.23) |
| Angola | 21.80 (12.70, 37.46) | 0.18 (0.12, 0.27) |  | 46.88 (26.73, 77.32) | 0.16 (0.11, 0.24) |  | 115.06 | -0.46 (-0.58, -0.35) |
| Antigua and Barbuda | 0.27 (0.19, 0.37) | 0.50 (0.35, 0.68) |  | 0.16 (0.11, 0.23) | 0.17 (0.12, 0.24) |  | -39.96 | -3.89 (-4.84, -2.93) |
| Argentina | 33.15 (24.78, 43.65) | 0.10 (0.08, 0.13) |  | 64.66 (45.87, 88.70) | 0.13 (0.09, 0.17) |  | 95.04 | 1.35 (1.13, 1.56) |
| Armenia | 3.44 (2.54, 4.22) | 0.10 (0.08, 0.13) |  | 9.64 (6.40, 13.87) | 0.26 (0.17, 0.37) |  | 180.06 | 5.30 (4.24, 6.37) |
| Australia | 17.99 (13.49, 24.06) | 0.10 (0.08, 0.13) |  | 74.54 (51.65, 103.46) | 0.20 (0.14, 0.27) |  | 314.26 | 2.82 (2.60, 3.03) |
| Austria | 17.78 (12.74, 24.59) | 0.17 (0.13, 0.22) |  | 33.29 (22.03, 47.78) | 0.21 (0.14, 0.29) |  | 87.21 | 0.99 (0.74, 1.24) |
| Azerbaijan | 7.06 (5.86, 8.65) | 0.10 (0.08, 0.12) |  | 16.24 (11.21, 24.19) | 0.19 (0.13, 0.29) |  | 130.11 | 3.09 (1.75, 4.45) |
| Bahamas | 0.85 (0.63, 1.13) | 0.49 (0.35, 0.68) |  | 0.76 (0.52, 1.07) | 0.20 (0.14, 0.28) |  | -10.00 | -3.36 (-4.20, -2.51) |
| Bahrain | 0.60 (0.44, 0.83) | 0.33 (0.22, 0.48) |  | 2.27 (1.47, 3.45) | 0.27 (0.18, 0.42) |  | 277.50 | -1.34 (-1.81, -0.86) |
| Bangladesh | 218.54 (153.13, 312.76) | 0.19 (0.14, 0.26) |  | 224.75 (170.16, 299.50) | 0.15 (0.12, 0.20) |  | 2.84 | -1.28 (-1.51, -1.04) |
| Barbados | 0.61 (0.41, 0.88) | 0.23 (0.16, 0.32) |  | 0.78 (0.52, 1.10) | 0.20 (0.14, 0.27) |  | 26.75 | -0.49 (-0.64, -0.33) |
| Belarus | 10.88 (8.49, 13.46) | 0.11 (0.08, 0.13) |  | 14.69 (9.97, 21.05) | 0.13 (0.10, 0.18) |  | 35.04 | 1.43 (1.03, 1.82) |
| Belgium | 21.06 (14.32, 30.04) | 0.15 (0.11, 0.21) |  | 36.14 (23.36, 51.95) | 0.18 (0.12, 0.24) |  | 71.56 | 0.43 (0.07, 0.80) |
| Belize | 0.54 (0.42, 0.68) | 0.47 (0.34, 0.62) |  | 0.59 (0.43, 0.81) | 0.20 (0.14, 0.27) |  | 10.80 | -3.08 (-3.71, -2.45) |
| Benin | 12.20 (8.74, 16.71) | 0.35 (0.24, 0.48) |  | 21.40 (14.50, 31.08) | 0.28 (0.18, 0.41) |  | 75.40 | -1.03 (-1.20, -0.86) |
| Bermuda | 0.24 (0.17, 0.34) | 0.40 (0.28, 0.56) |  | 0.12 (0.08, 0.18) | 0.11 (0.07, 0.15) |  | -50.23 | -4.52 (-5.51, -3.51) |
| Bhutan | 0.51 (0.22, 0.85) | 0.10 (0.05, 0.16) |  | 0.85 (0.57, 1.27) | 0.14 (0.09, 0.21) |  | 66.71 | 1.19 (1.02, 1.37) |
| Bolivia | 17.58 (11.67, 26.92) | 0.43 (0.27, 0.69) |  | 34.64 (22.32, 51.36) | 0.38 (0.24, 0.58) |  | 97.10 | -0.45 (-0.53, -0.37) |
| Bosnia and Herzegovina | 9.04 (6.33, 12.78) | 0.22 (0.16, 0.31) |  | 16.68 (10.12, 25.49) | 0.30 (0.19, 0.44) |  | 84.52 | 1.59 (1.17, 2.00) |
| Botswana | 0.38 (0.21, 0.72) | 0.05 (0.03, 0.11) |  | 1.22 (0.77, 1.81) | 0.07 (0.05, 0.11) |  | 219.78 | 0.59 (0.01, 1.18) |
| Brazil | 146.69 (132.01, 162.28) | 0.13 (0.12, 0.15) |  | 294.21 (256.34, 337.24) | 0.13 (0.12, 0.15) |  | 100.56 | 0.48 (0.36, 0.61) |
| Brunei | 0.41 (0.28, 0.57) | 0.39 (0.25, 0.58) |  | 1.44 (0.93, 2.09) | 0.47 (0.30, 0.66) |  | 255.40 | 1.16 (0.68, 1.65) |
| Bulgaria | 26.47 (18.11, 37.01) | 0.23 (0.16, 0.31) |  | 18.84 (11.92, 27.93) | 0.16 (0.10, 0.22) |  | -28.84 | -0.78 (-1.30, -0.25) |
| Burkina Faso | 29.95 (18.35, 46.97) | 0.25 (0.17, 0.34) |  | 56.24 (35.03, 87.69) | 0.22 (0.15, 0.30) |  | 87.78 | -0.65 (-0.93, -0.37) |
| Burundi | 13.31 (8.47, 21.29) | 0.31 (0.20, 0.46) |  | 18.62 (12.53, 27.77) | 0.24 (0.16, 0.38) |  | 39.84 | -1.19 (-1.31, -1.06) |
| Cambodia | 177.86 (85.30, 357.01) | 1.11 (0.62, 2.00) |  | 117.99 (68.18, 200.58) | 0.73 (0.44, 1.19) |  | -33.66 | -1.75 (-1.94, -1.56) |
| Cameroon | 2.02 (1.39, 2.87) | 0.03 (0.02, 0.05) |  | 6.29 (4.18, 8.70) | 0.04 (0.02, 0.05) |  | 211.05 | 0.18 (0.03, 0.34) |
| Canada | 40.00 (29.74, 53.38) | 0.13 (0.10, 0.17) |  | 143.47 (100.57, 203.07) | 0.24 (0.17, 0.32) |  | 258.68 | 2.36 (2.17, 2.55) |
| Cape Verde | 0.19 (0.15, 0.26) | 0.06 (0.05, 0.09) |  | 2.62 (1.84, 3.73) | 0.60 (0.41, 0.86) |  | 1256.74 | 6.30 (4.10, 8.54) |
| Central African Republic | 6.80 (3.93, 10.72) | 0.24 (0.15, 0.36) |  | 8.86 (5.26, 14.35) | 0.20 (0.12, 0.32) |  | 30.34 | -0.98 (-1.12, -0.84) |
| Chad | 14.97 (10.09, 20.80) | 0.34 (0.23, 0.47) |  | 27.79 (18.99, 38.40) | 0.30 (0.20, 0.43) |  | 85.63 | -0.58 (-0.67, -0.49) |
| Chile | 15.75 (11.78, 21.16) | 0.15 (0.11, 0.20) |  | 40.83 (28.55, 57.60) | 0.18 (0.13, 0.25) |  | 159.29 | 1.34 (1.10, 1.59) |
| China | 16280.81 (13226.78, 20051.23) | 1.73 (1.41, 2.13) |  | 10786.11 (8660.98, 13162.79) | 0.57 (0.46, 0.69) |  | -33.75 | -5.34 (-6.17, -4.51) |
| Colombia | 42.14 (33.70, 52.05) | 0.18 (0.14, 0.23) |  | 73.29 (48.70, 105.44) | 0.15 (0.10, 0.21) |  | 73.95 | -0.41 (-0.82, 0.01) |
| Comoros | 0.96 (0.50, 1.49) | 0.28 (0.15, 0.45) |  | 1.38 (0.86, 2.07) | 0.25 (0.16, 0.38) |  | 43.13 | -0.59 (-0.76, -0.42) |
| Congo | 5.51 (3.48, 8.18) | 0.24 (0.17, 0.34) |  | 6.21 (4.09, 9.05) | 0.16 (0.10, 0.23) |  | 12.67 | -1.78 (-1.93, -1.63) |
| Cook Islands | 0.08 (0.05, 0.11) | 0.58 (0.39, 0.82) |  | 0.10 (0.06, 0.14) | 0.41 (0.26, 0.59) |  | 20.28 | -1.18 (-1.26, -1.10) |
| Costa Rica | 6.18 (4.71, 7.99) | 0.30 (0.22, 0.41) |  | 13.10 (8.54, 19.15) | 0.26 (0.17, 0.39) |  | 111.81 | -0.65 (-1.16, -0.13) |
| Cote d'Ivoire | 25.66 (17.93, 35.75) | 0.35 (0.24, 0.50) |  | 36.73 (23.99, 53.25) | 0.25 (0.16, 0.37) |  | 43.13 | -1.82 (-2.09, -1.55) |
| Croatia | 8.33 (5.68, 11.67) | 0.14 (0.10, 0.19) |  | 10.56 (6.56, 15.83) | 0.13 (0.09, 0.19) |  | 26.78 | 0.03 (-0.47, 0.54) |
| Cuba | 44.47 (31.96, 60.06) | 0.43 (0.31, 0.58) |  | 23.05 (14.66, 34.23) | 0.13 (0.09, 0.19) |  | -48.17 | -4.44 (-5.60, -3.26) |
| Cyprus | 1.38 (0.93, 1.99) | 0.18 (0.12, 0.25) |  | 2.96 (1.94, 4.30) | 0.17 (0.11, 0.24) |  | 114.08 | 0.06 (-0.17, 0.29) |
| Czech Republic | 19.29 (13.49, 26.81) | 0.15 (0.11, 0.21) |  | 17.14 (10.85, 25.18) | 0.09 (0.06, 0.13) |  | -11.11 | -1.80 (-1.98, -1.62) |
| Democratic Republic of the Congo | 106.57 (67.68, 168.95) | 0.21 (0.15, 0.30) |  | 136.51 (70.75, 240.11) | 0.16 (0.10, 0.25) |  | 28.09 | -0.86 (-0.92, -0.80) |
| Denmark | 7.42 (5.03, 10.56) | 0.10 (0.08, 0.14) |  | 16.51 (10.84, 23.77) | 0.16 (0.12, 0.23) |  | 122.56 | 1.83 (1.60, 2.05) |
| Djibouti | 0.85 (0.54, 1.35) | 0.28 (0.17, 0.45) |  | 2.18 (1.29, 3.50) | 0.27 (0.16, 0.43) |  | 155.90 | -0.21 (-0.32, -0.11) |
| Dominica | 0.43 (0.30, 0.59) | 0.60 (0.42, 0.82) |  | 0.17 (0.11, 0.25) | 0.20 (0.14, 0.29) |  | -60.21 | -3.77 (-4.58, -2.96) |
| Dominican Republic | 16.46 (13.17, 20.46) | 0.32 (0.24, 0.42) |  | 31.04 (20.62, 46.13) | 0.32 (0.21, 0.49) |  | 88.52 | 0.35 (-0.18, 0.88) |
| Ecuador | 16.01 (12.32, 20.51) | 0.25 (0.18, 0.33) |  | 40.14 (27.24, 57.00) | 0.27 (0.18, 0.39) |  | 150.75 | 0.78 (0.40, 1.15) |
| Egypt | 367.25 (268.64, 502.98) | 0.87 (0.62, 1.22) |  | 755.24 (473.87, 1169.74) | 1.03 (0.63, 1.62) |  | 105.65 | 1.45 (1.08, 1.83) |
| El Salvador | 9.34 (7.39, 11.80) | 0.24 (0.18, 0.33) |  | 6.84 (4.48, 10.11) | 0.11 (0.07, 0.17) |  | -26.84 | -2.70 (-3.51, -1.89) |
| Equatorial Guinea | 0.95 (0.62, 1.42) | 0.20 (0.14, 0.28) |  | 1.47 (0.81, 2.46) | 0.16 (0.09, 0.26) |  | 54.38 | -0.77 (-0.88, -0.66) |
| Eritrea | 5.32 (3.30, 8.21) | 0.30 (0.18, 0.47) |  | 11.00 (7.20, 16.47) | 0.29 (0.19, 0.43) |  | 106.77 | -0.24 (-0.37, -0.10) |
| Estonia | 2.00 (1.38, 2.73) | 0.12 (0.08, 0.16) |  | 2.86 (1.77, 4.36) | 0.13 (0.08, 0.18) |  | 43.08 | -0.38 (-0.83, 0.08) |
| Eswatini | 1.13 (0.70, 1.96) | 0.29 (0.17, 0.52) |  | 5.21 (1.74, 10.29) | 0.75 (0.26, 1.46) |  | 360.82 | 4.25 (3.16, 5.35) |
| Ethiopia | 125.82 (76.44, 218.12) | 0.26 (0.17, 0.41) |  | 148.18 (111.96, 196.11) | 0.20 (0.16, 0.26) |  | 17.76 | -1.10 (-1.18, -1.02) |
| Federated States of Micronesia | 0.27 (0.17, 0.39) | 0.50 (0.31, 0.74) |  | 0.34 (0.19, 0.57) | 0.44 (0.26, 0.72) |  | 24.35 | -0.44 (-0.54, -0.33) |
| Fiji | 1.23 (0.85, 1.74) | 0.28 (0.18, 0.40) |  | 2.24 (1.44, 3.34) | 0.28 (0.18, 0.42) |  | 82.65 | 0.55 (0.19, 0.91) |
| Finland | 10.32 (7.18, 14.39) | 0.15 (0.11, 0.21) |  | 21.50 (13.68, 31.68) | 0.19 (0.13, 0.27) |  | 108.38 | 1.04 (0.93, 1.14) |
| France | 164.45 (114.17, 236.02) | 0.22 (0.15, 0.30) |  | 324.66 (214.75, 468.56) | 0.27 (0.18, 0.37) |  | 97.42 | 0.47 (0.22, 0.72) |
| Gabon | 2.14 (1.26, 3.32) | 0.21 (0.13, 0.30) |  | 2.16 (1.38, 3.19) | 0.16 (0.10, 0.23) |  | 1.06 | -1.09 (-1.19, -0.99) |
| Gambia | 11.00 (7.27, 15.46) | 1.56 (1.02, 2.22) |  | 27.76 (19.12, 38.22) | 2.00 (1.34, 2.89) |  | 152.36 | 0.61 (0.40, 0.82) |
| Georgia | 4.54 (3.00, 6.25) | 0.09 (0.06, 0.12) |  | 6.40 (4.32, 9.13) | 0.13 (0.09, 0.18) |  | 41.20 | 0.88 (0.17, 1.59) |
| Germany | 191.09 (136.49, 265.55) | 0.16 (0.12, 0.22) |  | 414.97 (280.43, 582.28) | 0.24 (0.17, 0.32) |  | 117.16 | 1.47 (1.12, 1.83) |
| Ghana | 29.81 (21.86, 40.18) | 0.28 (0.20, 0.39) |  | 62.79 (43.64, 86.28) | 0.28 (0.19, 0.41) |  | 110.62 | -0.25 (-0.42, -0.07) |
| Greece | 18.04 (12.21, 25.21) | 0.13 (0.09, 0.17) |  | 36.07 (23.01, 52.59) | 0.16 (0.11, 0.22) |  | 99.96 | 0.97 (0.82, 1.13) |
| Greenland | 0.16 (0.12, 0.22) | 0.41 (0.29, 0.56) |  | 0.34 (0.21, 0.53) | 0.50 (0.32, 0.74) |  | 106.90 | 0.46 (0.30, 0.63) |
| Grenada | 0.47 (0.34, 0.64) | 0.63 (0.45, 0.86) |  | 0.20 (0.14, 0.28) | 0.19 (0.13, 0.26) |  | -57.66 | -4.30 (-5.31, -3.28) |
| Guam | 0.14 (0.10, 0.19) | 0.16 (0.11, 0.22) |  | 0.36 (0.24, 0.52) | 0.19 (0.13, 0.27) |  | 166.00 | 1.03 (0.88, 1.18) |
| Guatemala | 31.30 (24.23, 40.43) | 0.65 (0.48, 0.89) |  | 39.24 (27.31, 53.94) | 0.31 (0.21, 0.44) |  | 25.36 | -3.32 (-4.85, -1.76) |
| Guinea | 83.21 (59.37, 113.42) | 1.55 (1.12, 2.15) |  | 135.63 (91.64, 196.13) | 1.64 (1.08, 2.38) |  | 62.99 | 0.24 (0.19, 0.28) |
| Guinea-Bissau | 2.66 (1.72, 4.04) | 0.41 (0.26, 0.62) |  | 3.32 (2.22, 4.95) | 0.34 (0.21, 0.52) |  | 24.99 | -0.77 (-0.84, -0.71) |
| Guyana | 2.09 (1.54, 2.77) | 0.46 (0.32, 0.63) |  | 1.36 (0.90, 1.98) | 0.21 (0.14, 0.31) |  | -34.94 | -2.71 (-3.51, -1.90) |
| Haiti | 21.15 (11.74, 35.24) | 0.52 (0.29, 0.84) |  | 25.20 (14.35, 39.29) | 0.31 (0.17, 0.49) |  | 19.16 | -1.80 (-2.04, -1.55) |
| Honduras | 18.58 (8.77, 28.85) | 0.70 (0.27, 1.17) |  | 54.67 (22.49, 91.99) | 0.86 (0.34, 1.46) |  | 194.23 | 0.86 (0.69, 1.03) |
| Hungary | 31.21 (21.45, 43.30) | 0.23 (0.17, 0.31) |  | 14.80 (9.48, 21.49) | 0.09 (0.06, 0.12) |  | -52.59 | -2.68 (-3.40, -1.95) |
| Iceland | 0.31 (0.22, 0.43) | 0.11 (0.08, 0.15) |  | 0.72 (0.47, 1.04) | 0.14 (0.10, 0.20) |  | 131.39 | 0.71 (0.56, 0.85) |
| India | 885.43 (749.62, 1030.76) | 0.13 (0.11, 0.15) |  | 1537.37 (1259.01, 1864.44) | 0.13 (0.11, 0.16) |  | 73.63 | -0.12 (-0.22, -0.02) |
| Indonesia | 278.44 (206.70, 399.92) | 0.16 (0.13, 0.22) |  | 244.46 (201.89, 291.00) | 0.12 (0.10, 0.14) |  | -12.20 | -1.29 (-1.36, -1.22) |
| Iran | 109.63 (90.13, 132.53) | 0.37 (0.29, 0.47) |  | 182.64 (156.50, 214.71) | 0.26 (0.22, 0.30) |  | 66.60 | -1.51 (-2.19, -0.82) |
| Iraq | 32.55 (23.51, 43.43) | 0.31 (0.20, 0.43) |  | 96.27 (65.61, 141.99) | 0.38 (0.25, 0.56) |  | 195.75 | 0.99 (0.62, 1.37) |
| Ireland | 3.51 (2.50, 4.74) | 0.09 (0.07, 0.12) |  | 11.97 (7.96, 16.89) | 0.17 (0.12, 0.23) |  | 241.52 | 2.88 (2.58, 3.19) |
| Israel | 8.39 (5.79, 11.90) | 0.17 (0.12, 0.24) |  | 18.78 (12.80, 26.57) | 0.17 (0.12, 0.24) |  | 123.85 | -0.12 (-0.23, 0.00) |
| Italy | 154.42 (130.00, 182.59) | 0.20 (0.17, 0.23) |  | 166.94 (137.88, 203.04) | 0.14 (0.12, 0.16) |  | 8.11 | -1.61 (-1.85, -1.37) |
| Jamaica | 5.28 (3.89, 7.12) | 0.28 (0.20, 0.38) |  | 5.18 (3.51, 7.38) | 0.18 (0.12, 0.25) |  | -2.02 | -0.80 (-1.76, 0.16) |
| Japan | 595.62 (510.06, 689.32) | 0.36 (0.31, 0.42) |  | 883.05 (710.46, 1081.66) | 0.26 (0.22, 0.30) |  | 48.26 | -2.08 (-2.61, -1.54) |
| Jordan | 3.78 (2.83, 4.97) | 0.21 (0.14, 0.31) |  | 10.21 (7.20, 14.41) | 0.14 (0.10, 0.21) |  | 169.71 | -1.65 (-1.85, -1.44) |
| Kazakhstan | 24.42 (17.09, 32.23) | 0.17 (0.12, 0.22) |  | 41.60 (28.51, 57.34) | 0.24 (0.17, 0.33) |  | 70.32 | -1.30 (-2.23, -0.36) |
| Kenya | 29.40 (22.74, 45.05) | 0.23 (0.17, 0.38) |  | 72.18 (49.50, 104.86) | 0.27 (0.18, 0.39) |  | 145.53 | 0.21 (-0.10, 0.52) |
| Kiribati | 0.31 (0.21, 0.43) | 0.66 (0.44, 0.94) |  | 0.50 (0.34, 0.74) | 0.60 (0.40, 0.90) |  | 63.62 | -0.34 (-0.40, -0.28) |
| Kuwait | 1.53 (1.22, 1.91) | 0.17 (0.12, 0.23) |  | 3.29 (2.27, 4.60) | 0.13 (0.09, 0.19) |  | 114.90 | -0.20 (-0.52, 0.12) |
| Kyrgyzstan | 4.15 (2.45, 5.75) | 0.09 (0.06, 0.12) |  | 5.82 (4.29, 7.57) | 0.11 (0.08, 0.15) |  | 40.21 | 1.30 (1.04, 1.55) |
| Lao | 9.32 (5.29, 16.16) | 0.33 (0.19, 0.53) |  | 10.85 (7.08, 15.69) | 0.21 (0.14, 0.31) |  | 16.47 | -1.73 (-1.83, -1.62) |
| Latvia | 2.97 (2.01, 4.04) | 0.10 (0.07, 0.14) |  | 3.35 (2.18, 4.89) | 0.10 (0.07, 0.14) |  | 12.79 | -0.46 (-0.97, 0.06) |
| Lebanon | 4.58 (3.16, 6.46) | 0.18 (0.12, 0.26) |  | 7.58 (4.86, 11.63) | 0.15 (0.09, 0.22) |  | 65.62 | -0.77 (-0.83, -0.70) |
| Lesotho | 2.87 (1.69, 5.25) | 0.26 (0.15, 0.47) |  | 8.65 (3.67, 14.93) | 0.62 (0.27, 1.07) |  | 201.23 | 3.56 (3.04, 4.09) |
| Liberia | 5.56 (3.71, 8.17) | 0.35 (0.24, 0.49) |  | 6.82 (4.45, 9.98) | 0.26 (0.16, 0.39) |  | 22.49 | -1.24 (-1.43, -1.06) |
| Libya | 9.24 (6.29, 13.23) | 0.38 (0.25, 0.56) |  | 17.55 (11.54, 26.70) | 0.33 (0.22, 0.50) |  | 89.93 | -0.36 (-0.51, -0.22) |
| Lithuania | 3.66 (2.47, 4.93) | 0.10 (0.06, 0.13) |  | 5.02 (3.26, 7.34) | 0.11 (0.08, 0.15) |  | 37.43 | 0.08 (-0.13, 0.29) |
| Luxembourg | 0.76 (0.52, 1.05) | 0.15 (0.11, 0.20) |  | 1.64 (1.05, 2.39) | 0.18 (0.12, 0.25) |  | 116.68 | 0.61 (0.39, 0.82) |
| Macedonia | 6.77 (4.90, 9.34) | 0.36 (0.26, 0.50) |  | 9.59 (6.04, 14.98) | 0.32 (0.21, 0.47) |  | 41.73 | -0.56 (-0.69, -0.43) |
| Madagascar | 25.21 (16.04, 39.30) | 0.27 (0.18, 0.41) |  | 35.56 (23.78, 52.36) | 0.22 (0.14, 0.33) |  | 41.08 | -0.82 (-0.91, -0.73) |
| Malawi | 51.71 (29.12, 88.14) | 0.46 (0.30, 0.67) |  | 58.61 (41.46, 78.94) | 0.35 (0.26, 0.47) |  | 13.34 | -1.32 (-1.59, -1.06) |
| Malaysia | 16.81 (12.15, 22.51) | 0.15 (0.10, 0.22) |  | 39.66 (25.57, 59.31) | 0.15 (0.10, 0.22) |  | 135.98 | 0.06 (-0.23, 0.35) |
| Maldives | 0.24 (0.15, 0.42) | 0.20 (0.12, 0.36) |  | 0.48 (0.33, 0.69) | 0.15 (0.10, 0.22) |  | 96.32 | -1.03 (-1.17, -0.90) |
| Mali | 68.50 (47.55, 96.36) | 0.80 (0.57, 1.13) |  | 92.81 (62.46, 132.50) | 0.67 (0.44, 0.99) |  | 35.49 | -0.91 (-1.20, -0.62) |
| Malta | 0.41 (0.29, 0.56) | 0.10 (0.07, 0.14) |  | 0.95 (0.62, 1.38) | 0.12 (0.09, 0.17) |  | 132.87 | 0.66 (0.46, 0.87) |
| Marshall Islands | 0.10 (0.07, 0.15) | 0.52 (0.32, 0.76) |  | 0.18 (0.11, 0.27) | 0.45 (0.27, 0.67) |  | 68.41 | -0.63 (-0.69, -0.56) |
| Mauritania | 4.80 (3.43, 6.49) | 0.36 (0.24, 0.51) |  | 5.70 (3.66, 8.30) | 0.23 (0.15, 0.35) |  | 18.63 | -1.53 (-1.68, -1.38) |
| Mauritius | 0.59 (0.45, 0.76) | 0.07 (0.05, 0.09) |  | 0.97 (0.63, 1.42) | 0.07 (0.04, 0.09) |  | 63.64 | 0.75 (0.43, 1.08) |
| Mexico | 108.51 (98.28, 119.63) | 0.18 (0.16, 0.20) |  | 263.38 (218.56, 312.77) | 0.23 (0.19, 0.27) |  | 142.72 | 1.04 (0.87, 1.20) |
| Moldova | 3.64 (2.29, 5.01) | 0.08 (0.05, 0.12) |  | 3.94 (2.68, 5.54) | 0.08 (0.06, 0.11) |  | 8.21 | -1.48 (-2.76, -0.18) |
| Monaco | 0.10 (0.06, 0.16) | 0.18 (0.12, 0.26) |  | 0.32 (0.19, 0.48) | 0.37 (0.24, 0.54) |  | 207.78 | 3.13 (2.36, 3.90) |
| Mongolia | 22.99 (15.20, 34.57) | 2.10 (1.34, 3.17) |  | 80.00 (51.23, 121.28) | 3.78 (2.39, 5.74) |  | 247.95 | 2.87 (2.27, 3.47) |
| Montenegro | 1.44 (1.03, 2.04) | 0.23 (0.17, 0.33) |  | 1.90 (1.25, 2.77) | 0.21 (0.14, 0.30) |  | 31.30 | -0.50 (-0.76, -0.23) |
| Morocco | 18.79 (13.10, 26.01) | 0.13 (0.08, 0.18) |  | 39.57 (25.49, 58.68) | 0.13 (0.09, 0.20) |  | 110.58 | 0.09 (-0.10, 0.28) |
| Mozambique | 22.41 (13.76, 35.85) | 0.20 (0.13, 0.30) |  | 72.06 (45.31, 112.20) | 0.32 (0.22, 0.46) |  | 221.54 | 1.77 (1.63, 1.91) |
| Myanmar | 58.99 (35.27, 106.44) | 0.16 (0.10, 0.26) |  | 118.93 (81.60, 169.60) | 0.25 (0.17, 0.35) |  | 101.62 | 1.42 (1.13, 1.71) |
| Namibia | 0.97 (0.59, 1.61) | 0.11 (0.06, 0.18) |  | 2.86 (1.91, 4.09) | 0.17 (0.11, 0.25) |  | 195.23 | 1.71 (1.46, 1.95) |
| Nauru | 0.03 (0.02, 0.04) | 0.48 (0.31, 0.72) |  | 0.02 (0.02, 0.04) | 0.42 (0.26, 0.68) |  | -3.12 | -0.51 (-0.73, -0.30) |
| Nepal | 30.75 (18.76, 52.26) | 0.13 (0.09, 0.20) |  | 33.92 (23.79, 52.56) | 0.13 (0.09, 0.20) |  | 10.31 | 0.16 (-0.03, 0.35) |
| Netherlands | 15.95 (11.66, 21.66) | 0.09 (0.07, 0.12) |  | 46.20 (31.44, 64.19) | 0.16 (0.12, 0.21) |  | 189.61 | 2.10 (1.98, 2.23) |
| New Zealand | 4.46 (3.91, 5.12) | 0.12 (0.11, 0.14) |  | 13.10 (11.08, 15.42) | 0.19 (0.17, 0.22) |  | 193.68 | 1.77 (1.59, 1.95) |
| Nicaragua | 5.27 (4.29, 6.50) | 0.21 (0.16, 0.28) |  | 10.73 (7.58, 14.58) | 0.22 (0.15, 0.31) |  | 103.53 | 0.46 (0.04, 0.89) |
| Niger | 1.52 (1.12, 2.06) | 0.03 (0.02, 0.05) |  | 4.45 (3.07, 6.38) | 0.03 (0.02, 0.05) |  | 192.28 | -0.09 (-0.19, -0.00) |
| Nigeria | 239.57 (183.35, 311.89) | 0.26 (0.20, 0.33) |  | 281.75 (191.24, 376.55) | 0.21 (0.16, 0.27) |  | 17.61 | -0.64 (-1.03, -0.24) |
| Niue | 0.01 (0.01, 0.01) | 0.37 (0.24, 0.53) |  | 0.01 (0.00, 0.01) | 0.31 (0.19, 0.46) |  | -22.91 | -0.71 (-0.76, -0.65) |
| North Korea | 168.53 (108.16, 245.13) | 0.92 (0.60, 1.32) |  | 187.76 (116.03, 278.77) | 0.59 (0.38, 0.86) |  | 11.41 | -1.76 (-1.89, -1.63) |
| Northern Mariana Islands | 0.07 (0.05, 0.10) | 0.29 (0.19, 0.43) |  | 0.14 (0.09, 0.21) | 0.26 (0.17, 0.38) |  | 98.60 | -0.25 (-0.40, -0.10) |
| Norway | 5.77 (4.98, 6.63) | 0.10 (0.09, 0.11) |  | 11.65 (9.70, 13.98) | 0.14 (0.12, 0.16) |  | 102.00 | 1.52 (1.36, 1.67) |
| Oman | 2.69 (1.91, 3.64) | 0.26 (0.17, 0.38) |  | 6.23 (4.60, 8.54) | 0.29 (0.20, 0.40) |  | 131.48 | 1.06 (0.66, 1.45) |
| Pakistan | 192.20 (144.89, 263.33) | 0.17 (0.13, 0.23) |  | 354.61 (277.66, 448.80) | 0.18 (0.14, 0.23) |  | 84.50 | 0.15 (0.07, 0.23) |
| Palau | 0.04 (0.02, 0.06) | 0.33 (0.20, 0.51) |  | 0.07 (0.04, 0.11) | 0.31 (0.20, 0.47) |  | 91.88 | -0.03 (-0.13, 0.06) |
| Palestine | 5.83 (3.81, 8.60) | 0.57 (0.35, 0.89) |  | 9.89 (7.02, 13.67) | 0.39 (0.26, 0.55) |  | 69.59 | -1.27 (-1.35, -1.19) |
| Panama | 3.86 (3.03, 4.94) | 0.21 (0.16, 0.29) |  | 7.08 (4.76, 10.00) | 0.17 (0.11, 0.24) |  | 83.48 | -0.46 (-0.85, -0.06) |
| Papua New Guinea | 1.89 (1.43, 2.46) | 0.07 (0.05, 0.10) |  | 4.76 (3.48, 6.54) | 0.07 (0.05, 0.11) |  | 151.50 | 0.40 (0.35, 0.45) |
| Paraguay | 4.15 (3.23, 5.30) | 0.14 (0.10, 0.18) |  | 6.07 (3.95, 8.91) | 0.11 (0.07, 0.15) |  | 46.15 | -0.76 (-1.27, -0.25) |
| Peru | 64.81 (49.11, 84.06) | 0.45 (0.31, 0.62) |  | 64.43 (42.77, 92.94) | 0.20 (0.13, 0.29) |  | -0.59 | -3.48 (-3.96, -2.99) |
| Philippines | 125.89 (102.94, 150.91) | 0.31 (0.24, 0.38) |  | 187.42 (152.76, 230.04) | 0.21 (0.17, 0.26) |  | 48.88 | -1.63 (-1.89, -1.37) |
| Poland | 125.81 (108.47, 144.82) | 0.30 (0.26, 0.35) |  | 43.98 (35.05, 54.47) | 0.07 (0.06, 0.09) |  | -65.05 | -4.63 (-5.86, -3.39) |
| Portugal | 13.97 (10.27, 18.78) | 0.12 (0.09, 0.15) |  | 43.30 (28.87, 62.20) | 0.22 (0.15, 0.30) |  | 210.03 | 1.91 (1.62, 2.19) |
| Puerto Rico | 14.24 (9.89, 19.64) | 0.39 (0.27, 0.54) |  | 9.57 (6.02, 14.25) | 0.15 (0.10, 0.21) |  | -32.79 | -2.39 (-3.14, -1.62) |
| Qatar | 0.92 (0.62, 1.33) | 0.89 (0.54, 1.35) |  | 4.97 (3.02, 7.89) | 0.71 (0.42, 1.16) |  | 437.33 | -0.48 (-0.65, -0.30) |
| Romania | 19.90 (14.70, 26.21) | 0.08 (0.06, 0.10) |  | 32.65 (21.27, 46.92) | 0.11 (0.08, 0.15) |  | 64.07 | 1.65 (1.32, 1.99) |
| Russia | 203.69 (181.48, 228.92) | 0.14 (0.13, 0.16) |  | 271.49 (215.97, 335.94) | 0.15 (0.11, 0.19) |  | 33.29 | 0.33 (0.03, 0.64) |
| Rwanda | 20.93 (12.69, 34.08) | 0.41 (0.26, 0.64) |  | 30.37 (19.94, 45.18) | 0.36 (0.24, 0.52) |  | 45.08 | -0.90 (-1.09, -0.72) |
| Saint Kitts and Nevis | 0.33 (0.23, 0.46) | 0.89 (0.63, 1.23) |  | 0.14 (0.09, 0.20) | 0.22 (0.14, 0.32) |  | -58.71 | -4.63 (-5.67, -3.59) |
| Saint Lucia | 0.39 (0.29, 0.52) | 0.41 (0.30, 0.56) |  | 0.26 (0.17, 0.37) | 0.13 (0.09, 0.18) |  | -33.84 | -4.22 (-5.27, -3.16) |
| Saint Vincent and the Grenadines | 0.39 (0.29, 0.51) | 0.50 (0.37, 0.66) |  | 0.23 (0.16, 0.31) | 0.18 (0.12, 0.24) |  | -42.36 | -3.47 (-4.32, -2.61) |
| Samoa | 0.26 (0.18, 0.37) | 0.25 (0.16, 0.36) |  | 0.32 (0.21, 0.47) | 0.20 (0.13, 0.29) |  | 22.82 | -0.68 (-0.78, -0.58) |
| San Marino | 0.04 (0.02, 0.05) | 0.13 (0.09, 0.17) |  | 0.08 (0.05, 0.13) | 0.15 (0.10, 0.23) |  | 123.87 | 1.02 (0.85, 1.20) |
| Sao Tome and Principe | 0.12 (0.09, 0.17) | 0.14 (0.10, 0.18) |  | 0.18 (0.13, 0.26) | 0.14 (0.09, 0.20) |  | 47.90 | 0.04 (-0.13, 0.21) |
| Saudi Arabia | 20.25 (13.99, 28.36) | 0.28 (0.17, 0.42) |  | 37.85 (23.67, 58.37) | 0.22 (0.13, 0.34) |  | 86.88 | -1.14 (-1.37, -0.90) |
| Senegal | 8.96 (6.33, 12.38) | 0.16 (0.11, 0.22) |  | 15.66 (10.50, 22.20) | 0.16 (0.10, 0.23) |  | 74.78 | 0.12 (-0.07, 0.31) |
| Serbia | 21.57 (14.81, 31.57) | 0.20 (0.14, 0.29) |  | 23.38 (14.31, 35.59) | 0.16 (0.10, 0.23) |  | 8.39 | -1.14 (-1.39, -0.89) |
| Seychelles | 0.14 (0.09, 0.19) | 0.23 (0.16, 0.33) |  | 0.17 (0.11, 0.24) | 0.16 (0.11, 0.22) |  | 22.75 | -1.14 (-1.44, -0.85) |
| Sierra Leone | 9.61 (6.44, 14.36) | 0.32 (0.22, 0.45) |  | 15.05 (10.03, 21.99) | 0.28 (0.18, 0.42) |  | 56.65 | -0.48 (-0.61, -0.35) |
| Singapore | 5.27 (3.70, 7.19) | 0.24 (0.17, 0.33) |  | 17.61 (11.10, 26.37) | 0.25 (0.16, 0.36) |  | 234.26 | 0.15 (-0.13, 0.43) |
| Slovakia | 9.43 (6.75, 12.75) | 0.16 (0.12, 0.22) |  | 9.47 (5.97, 13.95) | 0.12 (0.08, 0.17) |  | 0.38 | -1.67 (-2.00, -1.35) |
| Slovenia | 2.60 (1.65, 3.86) | 0.11 (0.07, 0.16) |  | 6.50 (3.94, 9.89) | 0.16 (0.10, 0.24) |  | 149.52 | 1.55 (1.26, 1.85) |
| Solomon Islands | 1.05 (0.71, 1.49) | 0.41 (0.28, 0.58) |  | 1.93 (1.36, 2.55) | 0.36 (0.26, 0.49) |  | 83.51 | -0.38 (-0.50, -0.26) |
| Somalia | 14.23 (7.69, 23.44) | 0.31 (0.16, 0.51) |  | 36.05 (21.89, 58.08) | 0.30 (0.18, 0.50) |  | 153.29 | 0.04 (-0.02, 0.11) |
| South Africa | 82.68 (58.92, 128.19) | 0.32 (0.22, 0.51) |  | 130.23 (108.67, 155.36) | 0.27 (0.23, 0.32) |  | 57.50 | -0.96 (-1.55, -0.37) |
[truncated: 9,126 more chars]
